# The updated Consolidated Framework for Implementation Research based on user feedback

**DOI:** 10.1186/s13012-022-01245-0

**Published:** 2022-10-29

**Authors:** Laura J. Damschroder, Caitlin M. Reardon, Marilla A. Opra Widerquist, Julie Lowery

**Affiliations:** VA Center for Clinical Management Research, VA Ann Arbor Healthcare System, 2215 Fuller Road, MI 48105 Ann Arbor, USA

**Keywords:** Implementation science, Implementation framework, Implementation determinants, Implementation outcomes, Implementation evaluation, Consolidated Framework for Implementation Research, CFIR, Theory

## Abstract

**Background:**

Many implementation efforts fail, even with highly developed plans for execution, because contextual factors can be powerful forces working against implementation in the real world. The Consolidated Framework for Implementation Research (CFIR) is one of the most commonly used determinant frameworks to assess these contextual factors; however, it has been over 10 years since publication and there is a need for updates. The purpose of this project was to elicit feedback from experienced CFIR users to inform updates to the framework.

**Methods:**

User feedback was obtained from two sources: (1) a literature review with a systematic search; and (2) a survey of authors who used the CFIR in a published study. Data were combined across both sources and reviewed to identify themes; a consensus approach was used to finalize all CFIR updates. The VA Ann Arbor Healthcare System IRB declared this study exempt from the requirements of 38 CFR 16 based on category 2.

**Results:**

The systematic search yielded 376 articles that contained the CFIR in the title and/or abstract and 334 unique authors with contact information; 59 articles included feedback on the CFIR. Forty percent (*n* = 134/334) of authors completed the survey. The CFIR received positive ratings on most framework sensibility items (e.g., applicability, usability), but respondents also provided recommendations for changes. Overall, updates to the CFIR include revisions to existing domains and constructs as well as the addition, removal, or relocation of constructs. These changes address important critiques of the CFIR, including better centering innovation recipients and adding determinants to equity in implementation.

**Conclusion:**

The updates in the CFIR reflect feedback from a growing community of CFIR users. Although there are many updates, constructs can be mapped back to the original CFIR to ensure longitudinal consistency. We encourage users to continue critiquing the CFIR, facilitating the evolution of the framework as implementation science advances.

**Supplementary Information:**

The online version contains supplementary material available at 10.1186/s13012-022-01245-0.

Contributions to the literature The updated CFIR manuscript:

- Provides an update of the Consolidated Framework for Implementation Research (CFIR), one of the most highly cited frameworks in implementation science.
- Addresses important user critiques of the CFIR based on the literature and a survey, including better centering innovation recipients, and adding determinants to equity in implementation.
- Demonstrates how determinant frameworks must evolve as implementation science matures to advance the science.

## Background

Far too many efforts to implement evidence-based innovations (EBIs) fail [1, 2], even with highly developed plans for execution [3]. In randomized controlled trials, innovations are tested in an environment where many contextual factors are controlled. However, implementation science embraces the reality that contextual factors are active and dynamic forces working for and against implementation efforts in the real world [4–7].

Theories that guide conceptualization of contextual factors are often encapsulated within determinant frameworks [8, 9]; these frameworks delineate determinants (i.e., barriers or facilitators) that influence the outcome of implementation efforts. Determinant frameworks provide a base set of concepts, terms, and definitions by which to articulate dynamic complex contexts and develop much needed measures of context [10]. As a discipline, implementation science spans both generalized theory-building and development of practical approaches for successful implementation; both researchers and practitioners use and benefit from determinant frameworks [11].

The Consolidated Framework for Implementation Research (CFIR) is among the most highly cited [12] frameworks in implementation science and has been listed in the top five most accessed articles within *Implementation Science* since its publication in 2009. Kirk et al.’s 2015 review of 26 articles with meaningful use of the CFIR found that most users employed mixed (*n* = 13) or qualitative (*n* = 10) methods and used the CFIR in the post-implementation phase (*n* = 15) [13]. As a determinant framework, the overarching aim of the CFIR is to predict or explain barriers and facilitators (determinants, independent variables) to implementation effectiveness (the outcome, dependent variable) [8]. Determinant frameworks can thus be used to inform choice of implementation strategies that may best address contextual determinants [14], generate hypotheses to prospectively guide predictions of implementation outcomes, or retrospectively explain implementation outcomes by assessing differences in determinants across implementation settings [11, 13, 15]*.*

Implementation scientists have been called to engage in “theory-building” science where theory is improved with every application and theorizing becomes “an iterative and recursive process” [16]. This means that theory should not be seen as immutable, but as something that should be refined considering empirical findings. The original CFIR article invited critique in recognition of the need for the framework to evolve [17]; the aim of this project was to elicit feedback from experienced CFIR users to inform updates to the framework.

## Methods

The VA Ann Arbor Healthcare System IRB declared this study exempt from the requirements of 38 CFR 16 based on category 2.

### Data collection

Feedback was obtained from two sources: (1) articles identified in a literature review with a systematic search; and (2) a survey of authors who used the CFIR in a published study.

#### Literature review

We completed a literature review to identify articles with feedback on the CFIR. The most efficient search criteria for this purpose was the inclusion of the CFIR in the title and/or abstract (see Additional file 1). We searched SCOPUS and Web of Science from 2009 (the year the CFIR was published) to July 6, 2020. This search yielded 376 articles, including (1) original research; (2) systematic reviews; and (3) evaluation of the CFIR as a framework. Two reviewers (MOW, CMR) read the full text of approximately 10% (*n* = 40/376) of the included articles to independently abstract feedback on the CFIR; discrepancies with abstraction were discussed until consensus was reached. One reviewer (MOW) then read the remaining articles and abstracted relevant passages. Only 59 of the articles contained feedback on the CFIR.

#### Author survey

We surveyed unique corresponding authors of the articles included in the literature review (*n* = 334) in August 2020 to elicit in-depth feedback about their experience using the CFIR. First, the survey elicited information about the author’s use of the CFIR (e.g., the total number of projects completed using the CFIR) (see Table 1). Second, respondents were asked to rate the CFIR based on Flottorp et al.’s “sensibility” criteria for determinant frameworks (e.g., Applicability, Simplicity) [18] (see Table 2). Third, respondents were asked for open-ended feedback about the framework overall as well as existing domains and constructs. Finally, respondents were asked for recommendations to add or remove domains and constructs (see Additional file 2 for the full survey). Survey invitations were sent via email with an embedded link to the survey.

**Table 1:** Use of the original CFIR

**Use of the CFIR**	*N*(%)
Number of projects in which the CFIR was used:	*N* = 128
1	42 (32.8)
2	32 (25.0)
3	19 (14.8)
4	11 (8.6)
≥ 5	24 (18.8)
Settings in which the CFIR was used:	*N* = 130
Healthcare	108 (83.1)
Public Health	45 (34.6)
Education	10 (7.7)
Agriculture	1 (0.8)
Other	15 (11.5)
Use of the CFIR:	*N* = 130
Guide data collection	107 (82.3)
Guide data analysis	114 (87.7)
Guide data interpretation	109 (83.8)
Design implementation strategy	42 (32.3)
Other	4 (3.1)

**Table 2:** User ratings of Flottorp’s Criteria for the original CFIR

Flottorp Criteria	Survey Item	Yes *N* (%)	Partially *N* (%)	No *N* (%)	Uncertain *N* (%)	*N*
Applicability	Applicable across different settings	86 (67.2)	19 (14.8)	2 (1.6)	21 (16.4)	128
Applicability	Applicable for different types of innovations	104 (81.3)	13 (10.2)	1 (0.8)	10 (7.8)	128
Simplicity	More complicated than necessary	20 (15.7)	54 (42.5)	50 (39.4)	3 (2.4)	127
Logic	Organized in a logical way that is easy to understand	73 (57.5)	50 (39.4)	3 (2.4)	1 (0.8)	127
Usability	Easy for implementation researchers to use	64 (50.4)	57 (44.8)	2 (1.6)	4 (3.1)	127
Usability	Easy for non-implementation researchers to use	20 (15.9)	46 (36.5)	37 (29.4)	23 (18.3)	126
Suitability	Suitable to identify and prioritize determinants	75 (59.1)	35 (27.6)	9 (7.1)	8 (6.3)	127
Usefulness	Useful for designing implementation strategies	83 (65.4)	33 (25.9)	4 (3.1)	7 (5.5)	127
Usefulness	Useful for reporting determinants	96 (76.8)	21 (16.8)	1 (0.8)	7 (5.6)	125
Clarity	Labeled/explained in a way that is easy to understand^a^	87 (76.9)	–	10 (8.8)	16 (14.2)	113
Comprehensiveness	Recommendations to add domains/constructs^b^	44 (41.5)	–	62 (58.5)	–	106
Relevance	Recommendations to remove domains constructs^b^	27 (28.1)	–	69 (71.8)	–	96
N/A	Helps compare findings across studies	74 (58.7)	29 (23.0)	4 (3.2)	19 (15.1)	126
N/A	Helps to advance or build theory	66 (52.4)	40 (31.7)	6 (4.8)	14 (11.1)	126

^a^Responses included Yes, No, Uncertain

^b^Responses included Yes, No

### Data analysis

Responses to closed-ended survey questions were analyzed using descriptive statistics. Responses to open-ended survey questions were combined with passages abstracted from the published literature in Microsoft Excel; feedback was organized in individual matrices at the framework, domain (i.e., one matrix for each domain), and construct (i.e., one matrix for each construct) levels. Matrices contained a row for each individual feedback item (including the source of the feedback, i.e., survey or literature) with a column for each analyst (CMR, LJD, JCL) to add notes and provide a recommendation on how to address the feedback. Additional literature was reviewed (1) when a user recommended a specific citation or (2) when a user identified a high-level issue (e.g., a construct was too broad), but did not provide a solution (e.g., did not suggest specific subconstructs). The team independently reviewed all feedback items to add their notes and recommendations, and then met approximately 3 h a week from September 2020 to February 2022 to discuss and reach consensus on CFIR updates.

### Positionality

We are all white, cisgender women. We are researchers embedded within and employed by the United States (US) Veterans Health Administration (VHA), the largest integrated healthcare system in the USA. The VHA has over 1000 medical centers, community-based outpatient clinics, and other entities, and serves 9.6 million enrolled US military Veterans. LJD and JCL were developers of the original CFIR. JCL has worked in implementation science in the VHA’s Quality Enhancement Research Initiative (QUERI) program since 2006 and has a Health Services Organization and Policy doctoral degree. LJD worked in management consulting for 20 years prior to joining the VHA and has two master’s degrees (biometrics and public health); she joined the VHA QUERI program in 2007. CMR is a qualitative analyst with 10 years of experience using the CFIR to collect, analyze, and interpret qualitative data from implementation evaluations. MOW is a Limited License Master’s Social Worker (LLMSW) and a research associate. Although LJD and CMR have consulted on dozens of projects outside the VHA and trained hundreds of CFIR users, most have been in US healthcare settings.

## Results

### Overview

The systematic search yielded 376 articles and 334 unique authors with contact information; 59 articles included feedback on the CFIR. Most of the projects discussed in the 59 articles were conducted in US healthcare settings; 27% (*n* = 16) were conducted in non-healthcare settings (e.g., educational, agricultural, or community settings), and 8% (*n* = 5) were conducted in low- and/or middle-income countries (LMICs) (see Additional file 3).

While 47% (*n* = 157/334) of authors responded to the survey, only 40% (*n* = 134/334) of authors completed the survey. Nearly 20% of authors reported use of the CFIR on five or more projects, and over 65% reported use in at least two projects. Over 80% of authors reported use of the CFIR in healthcare settings and to guide data collection, analysis, and/or interpretation (see Table 1).

While 50% of respondents felt the CFIR was easy to use for implementation science researchers, only 16% felt it was easy to use for non-researchers. In addition, 58% felt the CFIR was more complicated than necessary. One respondent stated: the “CFIR is far too complicated and difficult to use. I have been learning about and trying to use CFIR for more than 5 years and the more I use it the more difficult and uninterpretable I find it to be” (survey response). However, another observed that, “Implementation research is challenging in itself, and I see that the complexity of CFIR gets blamed for the broader challenges” (survey response). In addition, while the number of constructs was often cited as the reason the CFIR was too complicated, many users identified missing themes in the framework; nearly all respondents provided qualitative feedback about revising existing domain(s)/construct(s) or adding domain(s)/construct(s).

The other sensibility criteria from Flottorp et al. received positive ratings from over half of the survey respondents; most respondents felt the CFIR was applicable across settings (67%) and innovations (81%), useful for reporting determinants (77%) and designing implementation strategies (65%), and that the domains and constructs were labeled in a way that was easy to understand (77%) (see Table 2).

### CFIR updates

Table 3 details the updated CFIR domain and construct names and definitions; it is also included in Additional file 6 for user convenience (see below). Word limits prohibit the ability to describe the updated CFIR in detail, but more detail is available in the Additional files:

- Additional file 4 contains a mapping of the original CFIR constructs to the updated CFIR constructs;
- Additional file 5 contains the mapping in Additional file 4 with the rationale for each update based on user feedback; and
- Additional file 6 contains both the short and detailed descriptions of updated CFIR constructs, drawing on the descriptions from the original CFIR, feedback from our literature review, and support from other published literature.

**Table 3:** Updated CFIR domain and construct definitions

***Framework guidance:*** The CFIR is intended to be used to collect data from individuals who have power and/or influence over implementation outcomes. See the CFIR Outcomes Addendum for guidance on identifying these individuals and selecting outcomes [19] The CFIR must be fully operationalized prior to use in a project: (1) Define the subject of each domain for the project (see guidance for each domain below) (2) Replace broad construct language with project-specific language if needed (3) Add constructs to capture salient themes not included in the updated CFIR	
**I. Innovation domain** ***Innovation:*** The “thing” being implemented [20], e.g., a new clinical treatment, educational program, or city service ***Project Innovation:*** [Document the innovation being implemented, e.g., innovation type, innovation core vs. adaptable components, using a published reporting guideline [21–24]. Distinguish the innovation (the “thing” that continues when implementation is complete) [20, 25] from the implementation process and strategies used to implement the innovation [26, 27] (activities that end after implementation is complete) [28].]	
**Construct name**	**Construct definition** *The degree to which:*
A. Innovation Source	The group that developed and/or visibly sponsored use of the innovation is reputable, credible, and/or trustable
B. Innovation Evidence Base	The innovation has robust evidence supporting its effectiveness
C. Innovation Relative Advantage	The innovation is better than other available innovations or current practice
D. Innovation Adaptability	The innovation can be modified, tailored, or refined to fit local context or needs
E. Innovation Trialability	The innovation can be tested or piloted on a small scale and undone
F. Innovation Complexity	The innovation is complicated, which may be reflected by its scope and/or the nature and number of connections and steps
G. Innovation Design	The innovation is well designed and packaged, including how it is assembled, bundled, and presented
H. Innovation Cost	The innovation purchase and operating costs are affordable
**II. Outer Setting domain** ***Outer Setting:*** The setting in which the Inner Setting exists, e.g., hospital system, school district, state. There may be multiple Outer Settings and/or multiple levels within the Outer Setting, e.g., community, system, state ***Project Outer Setting(s):*** [Document the actual Outer Setting in the project, e.g., type, location, and the boundary between the Outer Setting and the Inner Setting.]	
**Construct name**	**Construct definition** *The degree to which:*
A. Critical Incidents	Large-scale and/or unanticipated events disrupt implementation and/or delivery of the innovation
B. Local Attitudes	Sociocultural values (e.g., shared responsibility in helping recipients) and beliefs (e.g., convictions about the worthiness of recipients) encourage the Outer Setting to support implementation and/or delivery of the innovation
C. Local Conditions	Economic, environmental, political, and/or technological conditions enable the Outer Setting to support implementation and/or delivery of the innovation
D. Partnerships & Connections	The Inner Setting is networked with external entities, including referral networks, academic affiliations, and professional organization networks
E. Policies & Laws	Legislation, regulations, professional group guidelines and recommendations, or accreditation standards support implementation and/or delivery of the innovation
F. Financing	Funding from external entities (e.g., grants, reimbursement) is available to implement and/or deliver the innovation
G. External Pressure	External pressures drive implementation and/or delivery of the innovation *Use this construct to capture themes related to External Pressures that are not included in the subconstructs below*
1. Societal Pressure	Mass media campaigns, advocacy groups, or social movements or protests drive implementation and/or delivery of the innovation
2. Market Pressure	Competing with and/or imitating peer entities drives implementation and/or delivery of the innovation
3. Performance Measurement Pressure	Quality or benchmarking metrics or established service goals drive implementation and/or delivery of the innovation
**III. Inner Setting domain** ***Inner Setting:*** The setting in which the innovation is implemented, e.g., hospital, school, city. There may be multiple Inner Settings and/or multiple levels within the Inner Setting, e.g., unit, classroom, team ***Project Inner Setting(s):*** [Document the actual Inner Setting in the project, e.g., type, location, and the boundary between the Outer Setting and the Inner Setting.]	
**Construct name**	**Construct definition** *The degree to which:*
*Note:*	*Constructs A – D exist in the Inner Setting regardless of implementation and/or delivery of the innovation, i.e., they are persistent general characteristics of the Inner Setting*
A. Structural Characteristics	Infrastructure components support functional performance of the Inner Setting *Use this construct to capture themes related to Structural Characteristics that are not included in the subconstructs below*
1. Physical Infrastructure	Layout and configuration of space and other tangible material features support functional performance of the Inner Setting
2. Information Technology Infrastructure	Technological systems for tele-communication, electronic documentation, and data storage, management, reporting, and analysis support functional performance of the Inner Setting
3. Work Infrastructure	Organization of tasks and responsibilities within and between individuals and teams, and general staffing levels, support functional performance of the Inner Setting
B. Relational Connections	There are high quality formal and informal relationships, networks, and teams within and across Inner Setting boundaries (e.g., structural, professional)
C. Communications	There are high quality formal and informal information sharing practices within and across Inner Setting boundaries (e.g., structural, professional)
D. Culture	There are shared values, beliefs, and norms across the Inner Setting *Use this construct to capture themes related to Culture that are not included in the subconstructs below*
1. Human Equality-Centeredness	There are shared values, beliefs, and norms about the inherent equal worth and value of all human beings
2. Recipient-Centeredness	There are shared values, beliefs, and norms around caring, supporting, and addressing the needs and welfare of recipients
3. Deliverer-Centeredness	There are shared values, beliefs, and norms around caring, supporting, and addressing the needs and welfare of deliverers
4. Learning-Centeredness	There are shared values, beliefs, and norms around psychological safety, continual improvement, and using data to inform practice
*Note:*	*Constructs E – K are specific to the implementation and/or delivery of the innovation*
E. Tension for Change	The current situation is intolerable and needs to change
F. Compatibility	The innovation fits with workflows, systems, and processes
G. Relative Priority	Implementing and delivering the innovation is important compared to other initiatives
H. Incentive Systems	Tangible and/or intangible incentives and rewards and/or disincentives and punishments support implementation and delivery of the innovation
I. Mission Alignment	Implementing and delivering the innovation is in line with the overarching commitment, purpose, or goals in the Inner Setting
J. Available Resources	Resources are available to implement and deliver the innovation *Use this construct to capture themes related to Available Resources that are not included in the subconstructs below*
1. Funding	Funding is available to implement and deliver the innovation
2. Space	Physical space is available to implement and deliver the innovation
3. Materials & Equipment	Supplies are available to implement and deliver the innovation
K. Access to Knowledge & Information	Guidance and/or training is accessible to implement and deliver the innovation
**IV. Individuals domain** ***Individuals:*** The roles and characteristics of individuals	
**Roles subdomain** ***Project Roles:*** [Document the roles applicable to the project and their location in the Inner Setting or Outer Setting.]	
**Construct name**	**Construct definition**
A. High-level Leaders	Individuals with a high level of authority, including key decision-makers, executive leaders, or directors
B. Mid-level Leaders	Individuals with a moderate level of authority, including leaders supervised by a high-level leader and who supervise others
C. Opinion Leaders	Individuals with informal influence on the attitudes and behaviors of others
D. Implementation Facilitators	Individuals with subject matter expertise who assist, coach, or support implementation
E. Implementation Leads	Individuals who lead efforts to implement the innovation
F. Implementation Team Members	Individuals who collaborate with and support the Implementation Leads to implement the innovation, ideally including Innovation Deliverers and Recipients
G. Other Implementation Support	Individuals who support the Implementation Leads and/or Implementation Team Members to implement the innovation
H. Innovation Deliverers	Individuals who are directly or indirectly delivering the innovation
I. Innovation Recipients	Individuals who are directly or indirectly receiving the innovation
**Characteristics subdomain** ***Project Characteristics:*** [Document the characteristics applicable to the roles in the project based on the COM-B system [29] or role-specific theories.]	
**Construct name**	**Construct definition:** *The degree to which:*
A. Need	The individual(s) has deficits related to survival, well-being, or personal fulfillment, which will be addressed by implementation and/or delivery of the innovation
B. Capability	The individual(s) has interpersonal competence, knowledge, and skills to fulfill Role
C. Opportunity	The individual(s) has availability, scope, and power to fulfill Role
D. Motivation	The individual(s) is committed to fulfilling Role
**V. Implementation Process domain** ***Implementation Process:*** The activities and strategies used to implement the innovation ***Project Implementation Process:*** [Document the implementation process framework [8] and/or activities and strategies [26, 27] being used to implement the innovation. Distinguish the implementation process used to implement the innovation (activities that end after implementation is complete) from the innovation (the “thing” that continues when implementation is complete) [20, 25, 28].	
**Construct name**	**Construct definition:** *The degree to which individuals:*
A. Teaming	Join together, intentionally coordinating and collaborating on interdependent tasks, to implement the innovation
B. Assessing Needs	Collect information about priorities, preferences, and needs of people *Use this construct to capture themes related to Assessing Needs that are not included in the subconstructs below*
1. Innovation Deliverers	Collect information about the priorities, preferences, and needs of deliverers to guide implementation and delivery of the innovation
2. Innovation Recipients	Collect information about the priorities, preferences, and needs of recipients to guide implementation and delivery of the innovation
C. Assessing Context	Collect information to identify and appraise barriers and facilitators to implementation and delivery of the innovation
D. Planning	Identify roles and responsibilities, outline specific steps and milestones, and define goals and measures for implementation success in advance
E. Tailoring Strategies	Choose and operationalize implementation strategies to address barriers, leverage facilitators, and fit context
F. Engaging	Attract and encourage participation in implementation and/or the innovation *Use this construct to capture themes related to Engaging that are not included in the subconstructs below*
1. Innovation Deliverers	Attract and encourage deliverers to serve on the implementation team and/or to deliver the innovation
2. Innovation Recipients	Attract and encourage recipients to serve on the implementation team and/or participate in the innovation
G. Doing	Implement in small steps, tests, or cycles of change to trial and cumulatively optimize delivery of the innovation
H. Reflecting & Evaluating	Collect and discuss quantitative and qualitative information about the success of implementation and/or the innovation *Use this construct to capture themes related to Reflecting & Evaluating that are not included in the subconstructs below*
1. Implementation	Collect and discuss quantitative and qualitative information about the success of implementation
2. Innovation	Collect and discuss quantitative and qualitative information about the success of the innovation
I. Adapting	Modify the innovation and/or the Inner Setting for optimal fit and integration into work processes

In the sections below, we summarize key updates in the updated CFIR and refer readers to the additional files and CFIR Outcomes Addendum [19] for details.

### Overall framework

Construct names and definitions were updated in response to recommendations to make the framework more applicable across a range of innovations and settings [30–35]. This includes (1) using *innovation* (following Rogers that any “idea, practice, or object perceived as new” is an innovation) [36] rather than intervention; (2) using *recipients* (individuals intended to benefit from the innovation) rather than patients; and (3) using *deliverers* (individuals involved in delivering the innovation). In addition, we have removed all references to stakeholders and instead refer to people who “have influence and/or power over the outcome of implementation efforts” when discussing how to identify a sample for data collection. Overall, every domain and construct had at least minor revisions.

Some survey respondents were unclear whether the CFIR seeks to elicit perceptions or reality: “A difficult distinction here is whether these are PERCEPTIONS [*sic*] of the implementer, or actual features of the program; both seem important” (survey response). Underlying assessment theories are needed to fully explicate a response to this concern. However, we acknowledge that responses to questions related to CFIR constructs will likely reflect a blend of objective reality and subjective perceptions that arise out of experiences within the setting (see “Discussion”).

Constructs and subconstructs were added to address missing themes and further develop domains; the number of constructs and subconstructs increased in all domains except the *Innovation Domain*; the updated CFIR contains 48 constructs and 19 subconstructs across 5 domains (with one domain including two subdomains). Domain-specific changes are summarized in the sections below and reflect our consensus decisions based on published feedback (noted by citations) and survey responses.

### Innovation domain

#### Domain level

Survey respondents questioned whether the CFIR was intended to evaluate the innovation and/or the strategy being used to implement the innovation, and they found it difficult to differentiate between them. The literature has recognized that the lack of a clean boundary between the innovation and implementation strategies is a contributor to implementation complexity [22]; however, distinguishing between the innovation and implementation strategy is necessary for accurate attribution to implementation outcomes [28] and to identify appropriate areas for future intervention. As a result, the updated CFIR guides users to define the innovation (aka “the thing” [20, 25] being implemented), including the boundary between the innovation and implementation strategies. We encourage use of a reporting guideline to define the innovation (see Table 3).

#### Constructs and subconstructs

The word *Innovation* was added to the name of each construct in the *Innovation Domain* to orient users to the focus of this domain: the Innovation *itself*, independent of the implementation strategy. Major revisions were made to the definition of *Innovation Complexity*: the text “difficulty of implementation” was replaced with “the innovation is complicated” to focus attention on the innovation, not implementation.

### Outer Setting domain

#### Domain level

While some users recommended dividing the *Outer Setting* into multiple levels, others wanted to combine the *Outer and Inner Settings*, describing difficulty understanding boundaries between the two settings. In the original CFIR article's Additional file 1, the boundary between the *Inner and Outer Settings* was visually depicted using “overlapping, irregular, and thick grayed lines” to highlight that the line between them is not always clear [17]. Lengnick-Hall et al. expand on this reality and call for researchers to take an “open-systems” perspective “to highlight interdependence between outer and inner contexts and [to] view organizations as part of a broader interdependent system that may range from simple to complex, rigid to flexible, and loosely to tightly coupled” [37]. Although embracing an open-systems perspective may be challenging, conceptually differentiating internal and external influences on the performance of organizations has been a central tenet of organization science [38] and highlights the level at which to focus interventions. As a result, the updated CFIR retains the two domains and guides users to objectively define their *Outer vs. Inner Settings*, including defining multiple levels of the *Outer Setting* if appropriate.

#### Constructs and subconstructs

A few constructs were renamed because users felt the labels were unintuitive (e.g., *Cosmopolitanism*) or confusing (e.g., *Peer Pressure*). *Patient Needs and Resources* was separated into three constructs and relocated to the *Inner Setting* and *Individuals Domains* in response to comments that it captured two distinct themes: awareness of patient needs versus prioritization of patient needs [39].

Users remarked that the Outer Setting domain was underdeveloped [40, 41]. The updated CFIR adds constructs to capture the potential influence of *Local Attitudes*, i.e., sociocultural values and beliefs, and *Local Conditions*, i.e., economic, environmental, political, and/or technological conditions, on the willingness and ability of entities within the *Outer Setting* to support implementation and delivery of the innovation [42–47], which may influence equity in implementation. These constructs are especially important for innovations that require support from community entities, such as Housing First models of care [48], and for capturing common resource constraints in LMICs [42].

The original CFIR’s broad construct, *External Policies and Incentives*, was separated into several new constructs, including, for example, the key role of *Financing* [46, 49–51]. The updated CFIR also better captures diverse sources of *External Pressures* [46], including *Societal Pressure* (e.g., pressure from social movements and protests) [45], *Market Pressure* (e.g., pressure to compete with and/or imitate peer entities), and *Performance Measurement Pressure* (e.g., pressure to meet publicly reported goals).

### Inner Setting domain

#### Domain level

Some users recommended dividing the *Inner Setting* into multiple levels [52] to account for teams or units [53, 54]. We added guidance for users to objectively define their *Inner Setting* and to add additional levels as needed. For example, Safaeinili et al. adapted the CFIR to accommodate three embedded levels: (1) pilot clinics, (2) peer clinics, and (3) the larger health system [54].

#### Constructs and subconstructs

New constructs and subconstructs were added to the *Inner Setting* to address several critiques. For example, *Culture* was felt to be too broad, with one survey respondent stating, it “ends up becoming my ‘I don’t know where else this fits’ bucket” (survey response). Additionally, users noted the absence of equity considerations [40, 42], including “more specifically racism, patriarchy and misogyny, that [are] so much a part of the care that we provide” (survey response). As a result, four subconstructs were added to Culture, including *Human Equality-Centeredness*,* Recipient-Centeredness*,* Deliverer-Centeredness*, and *Learning-Centeredness*, which serve to orient users to determinants that may influence equity in implementation.

In addition, as described in our companion article, The CFIR Outcomes Addendum [19], *Implementation Climate* and *Readiness for Implementation* were removed from the updated CFIR. Though few users commented on these constructs, some questioned their meaning and “nesting” of subconstructs within each in the framework (e.g., *Leadership Engagement, Available Resources,* and *Access to Knowledge and Information* were all nested within *Readiness for Implementation*). Though there is broad recognition that implementation climate and readiness are a function of multiple implementation determinants, there is no consensus on precisely which determinants. Therefore, we have reclassified these constructs to more appropriately position them as antecedent assessments [55], on the pathway between implementation determinants and outcomes in the CFIR Outcomes Addendum [19].

### Individuals domain

#### Domain level

Many users felt the CFIR did not provide “sufficient individual-level constructs” [45] and were unclear about which individuals were included [45–47, 56–59]. Furthermore, they felt that constructs in this domain overlapped with constructs in other domains and failed to capture more important individual-level characteristics. One user summarized this feedback well: “[The CFIR needs to focus] more on who the individuals are and their underlying characteristics” (survey response). As a result, the *Individuals Domain* was significantly reorganized and now includes two subdomains: *Roles* and *Characteristics*.

#### Roles subdomain

In the original CFIR, roles were spread across three different domains: *Patient Needs and Resources* was listed in the *Outer Setting*, *Leadership Engagement* was listed in the *Inner Setting*, and multiple implementation-specific roles were listed in the *Process Domain* (e.g., *Formally Appointed Internal Implementation Leaders*). All roles have been relocated to this new subdomain, and additional roles were added, including *Implementation Team Members* [60]. In addition, the *Formally Appointed Internal Implementation Leader* and *Champion* constructs were combined into the *Implementation Leads* role because of the inability of users to distinguish between the two roles [61], and as affirmed in a review of champions [62].

#### Characteristics subdomain

Users felt that the existing *Characteristics* constructs overlapped with constructs in other domains, e.g., *Knowledge and Beliefs* overlapped with all constructs in the *Innovation Domain*. In addition, they thought the domain failed to capture more relevant characteristics related to professional roles and identities, skills and capabilities, autonomy, and level of involvement [46, 47, 59]. Some CFIR users combine this domain with the Theoretical Domains Framework (TDF), which was developed with the intent “to simplify and integrate a plethora of behavior change theories and make theory more accessible to, and usable by, other disciplines” [63]. The COM-B system was developed as an even more simplified system by which to acknowledge key domains related to behavior change based on US consensus of behavioral theorists and a principle of criminal law defining specific prerequisites for volitional behavior [29]. As a result, the original CFIR *Characteristics* constructs were replaced with constructs based on Michie et al.’s COM-B system [29]. The COM-B posits that broad categories of *Capability* (e.g., skills), *Opportunity* (e.g., autonomy), and *Motivation* (e.g., commitment) shape behavior.

The COM-B constructs are each mapped to 14 domains in the TDF, which provides CFIR users a wide portal into a repository of 84 behavior-change theoretical constructs. In addition, we encourage users to add additional constructs and map them to the COM-B as appropriate. For example, theories, models, and frameworks related to:

- Behavior change, e.g., the TDF [63, 64], the Theory of Planned Behavior [65], or the Social Ecological Theory [66] may provide constructs relevant for *Innovation Recipients* and *Innovation Deliverers.*
- Facilitation [67, 68] and project management [69, 70] may provide constructs relevant for *Implementation Facilitators* and *Implementation Leads.*
- Leadership [67, 68] may provide constructs relevant for *High- and Mid-Level Leaders.*

We also added the *Need* construct, based on feedback about its importance for all constituents [56], and to capture facets of the original CFIR *Patient Needs and Resources* construct.

### Implementation Process

#### Domain level

We added guidance to encourage users to describe their overall approach or implementation process framework to guide implementation, e.g., the Interactive Systems Framework [71]. Doing so helps distinguish the *Innovation* from the *Implementation Process* and accompanying implementation strategies.

Some users questioned the inclusion of the *Implementation Process Domain* in the CFIR because it appears to include strategies, not contextual factors. We clarify that the goal of this domain is to capture “the degree to which” each of these processes occur during implementation and influence implementation outcomes. Additional constructs were added in the updated CFIR to acknowledge scientific advancement since 2009 that are common across many process frameworks [8] and collective-level change theories [72]. Depending on the process framework used for a particular project and the implementation strategies used [26, 27], there may be other components of the implementation process that users should add.

#### Constructs and subconstructs

The updated CFIR has expanded the number of constructs within the *Implementation Process Domain* in response to critiques that key processes and strategies were missing. Though it is outside the scope of the CFIR to include all 73 implementation strategies from the Expert Recommendations for Implementing Change (ERIC) [26, 27], a few best practices have been added: *Teaming* [42, 46, 73],* Assessing Needs* [46, 47], *Assessing Context*,* Tailoring Strategies* [14], and *Adapting* [45, 74, 75]. Published guidance highlights the importance of *Adapting* the innovation [76], and the updated CFIR notes the importance of adapting the setting as well [77]. The addition of *Assessing Needs: Innovation Recipients* and *Engaging: Innovation Recipients* serves to better center *Innovation Recipients* in the updated CFIR and orient users to these as important determinants to equity in implementation.

## Discussion

In the original CFIR article, we called for users to publish their reflections on three key questions: (1) Is the framework’s terminology and language coherent? (2) Does the framework promote comparison of results across contexts and studies over time? (3) Does the framework stimulate new theoretical development? This feedback was used to evolve the CFIR. However, only 59 of 376 (15.7%) articles in the literature review contained feedback to improve the CFIR. The survey expanded this rate 2.5-fold: 40% of 334 authors provided feedback in our follow-on survey. We echo Kislov et al.’s call to move into theorizing as “an iterative and recursive process [where] theory is no longer seen as ‘fixed and immutable’” but rather as a living, evolving, improving set of propositions, principles, and hypotheses [16], to which we contribute as a component of every application of theory in every study.

The addition of constructs better aligns the updated CFIR with other published frameworks. For example, Nilsen and Bernhardsson evaluated 17 determinant frameworks with clearly distinguishable dimensions. They concluded that the original CFIR only addressed 10 of 12 identified dimensions; the updated CFIR now addresses all 12 dimensions with the addition of the *Characteristics: Opportunity* construct in the *Individuals Domain*, to capture dedicated time, and the *Structural Characteristics: Physical Infrastructure* subconstruct in the *Inner Setting Domain*, to capture the physical environment [41]. Expansion of the *Outer Setting* also brings the updated CFIR into closer alignment with other implementation and policy frameworks [18, 78–80].

### Framework scope and purpose

As detailed in our companion article, the CFIR Outcomes Addendum, several users suggested that the CFIR be expanded to include (1) implementation and innovation outcomes and (2) determinants to innovation outcomes collected from recipients [19]. However, these are both outside the scope of the CFIR, which is an implementation determinant framework [8] designed to describe barriers and facilitators to implementation outcomes [17]. The CFIR Outcomes Addendum provides high-level guidance for identifying implementation outcomes by drawing on other frameworks [81, 82]. It also clarifies that data on CFIR constructs should be collected from those who have power and/or influence over *implementation* outcomes; data collected from recipients not involved in implementation should be a source of information for *innovation* determinants and outcomes.

### Construct operationalization

The CFIR is a generalized framework, but adaptations have been developed for diverse innovations and settings, e.g., educational, agricultural, community, and low- and middle-income contexts [42, 46, 47, 54] (see Additional file 3). Though the CFIR provides relatively detailed definitions for each construct [9], it is essential for users to fully operationalize constructs by adapting and using language that is meaningful for the context and individuals involved in implementing and delivering the innovation.

### Construct selection

The updated CFIR significantly expands the number of constructs. It is often not feasible to assess every construct in the framework; nor will every construct apply within every project. In order to purposefully select a subset of constructs for assessment, users can rely on (1) consensus discussions and/or surveys with experts, (2) empirical studies or prior work, or [3] policy or change theories, including theories of organizational- [40] and individual-level [63, 83] change. For example, an implementation model developed by Klein et al. [84], comprising only seven constructs, was used to focus the evaluation in one study [85]. In all cases, it is important to elicit and analyze data from open-ended questions to explore the possibility of themes not captured by existing constructs. In addition, even if only a subset of constructs is used to guide data collection, data should be coded to additional CFIR constructs during the analysis, interpretation, or reporting phases as appropriate.

### Construct measurement

The majority of CFIR users employ qualitative methods to assess constructs [13]. However, more users are employing quantitative assessment approaches, including established methods to quantify qualitative data by applying ratings of valence (positive to negative manifestation) and strength (weak to strong manifestation) to qualitative data for each construct [86, 87]. A key challenge for measurement is the focus on *what* is being measured. Some CFIR users wanted more explanation about whether constructs were intended to capture perceptions or objective reality. At the construct level, we explicitly guide CFIR users to elicit “the degree to which” each construct manifests as defined. Perceptions and shared meanings, arising through social interactions among individuals in the workplace [88], are an important influence on how people respond to this question, along with objective consideration of presence or absence of specific factors related to each construct. Thus, assessing the “degree to which” each construct manifests will likely elicit responses based on a blend of subjective judgements combined with objective fact; for example, *Structural Characteristics: IT Infrastructure* may capture the factual presence of an electronic health record system (EHR) as well as subjective perceptions about the degree to which that EHR supports functional performance in the *Inner Setting*.

Systematic reviews of implementation context measures have been conducted, all of which have found significant gaps and signal the continued need for measure validation and development [89–95]. Development of quantitative measures must be intimately linked to underlying theory; validation of measures relies on establishing empirical validity of underlying theories encapsulated in constructs including use of appropriate response scales [96]. Lewis et al.’s measurement reviews on the original CFIR constructs [10] focused specifically on assessing questionnaires administered to healthcare professionals or leaders within behavioral health settings [97] using multi-dimensional criteria for validity and pragmatism [98]. The most highly rated measures typically elicit self-ratings using Likert-type scales. In the updated CFIR, the addition of the definition stem “the degree to which” to constructs that have clear labels and definitions provides a strong starting point to assess construct and content validity for quantitative measures.

### Equity in implementation

Although the updated CFIR includes new constructs to better assess determinants related to equity in implementation, we urge users to collaborate with equity experts [99] to combine use of equity, justice, or non-discrimination theories with the CFIR as a lens through which to view *all* facets of implementation [100]. For example, Allen et al. adapted the CFIR using the Public Health Critical Race Praxis to understand the ways structural racism influence implementation of an equity program across all constructs [101]. Researchers have produced decades of findings focused on the role of individual (e.g., race) and structural (e.g., access to care) determinants of health in highlighting inequities in services and outcomes [102]. We must move upstream, past spurious individual-level determinants [99] to recognize the role of racism and other systems of oppression as the source of these outcomes [103–105]. Lett et al. challenge us to center equity by asking ourselves: Who is represented in the study? How can this work cause harm [99]? This requires understanding our own positionality, i.e., who we are, relative to who *should* have influence and/or power over implementation, being deliberate in collaborating with communities and deeply knowledgeable equity researchers, and prioritizing sustainability over urgency in research [99]. Our own team’s lack of equity expertise and our narrow positionality disallow us from addressing the urgent need to adequately center equity within the CFIR.

Notwithstanding our personal limitations, implementation researchers are uniquely positioned to address oppression by seeking to understand how it manifests across all domains as a determinant to equitable implementation. Approaches to build competency and ingrain collaborative critique and reflexive methods into professional practice do exist and could help teams center equity and make meaningful impact [106]. We must seek opportunities to subvert established systems of oppression by including and sharing power with members of historically excluded groups in implementation and evaluation. When first planning implementation of an innovation, researchers should use a multilevel approach to identify implementation strategies that will address equity [100], e.g., including recipients and other community members in choosing and adapting the innovation and implementation strategies. When evaluating implementation, researchers should combine use of an equity-focused framework (e.g., the HEIF [107]) and a broader theoretical lens (e.g., critical race theory [101, 108]) with the CFIR to identify potential determinants and implementation outcomes [109], and deliberately include historically excluded recipients and deliverers in this process [99].

### Limitations

Our survey was only sent to authors identified via our literature search, and our literature search was limited to articles that included the CFIR in the title and/or abstract published before July 2020. As a result, we may have missed valuable feedback from (1) implementation scientists and practitioners who used other determinant frameworks, (2) authors who used the CFIR but did not include it in the title and/or abstract of their article, and (3) authors who included the CFIR in the title and/or abstract but published after July 2020. Including non-users or users with less experience could potentially broaden the tenets and design of the CFIR. However, we purposefully focused on feedback from individuals with experience using the CFIR; these individuals have applied the framework, providing them with firsthand knowledge of issues with the CFIR. While it was not feasible to update our search after July 2020, we added the *Outer Setting: Critical Incidents* construct to capture the influence of large-scale events (e.g., the COVID 19 pandemic), which may disrupt implementation and/or delivery of an innovation. In addition, many survey respondents asked for more clarification about how to apply the CFIR and differentiate between specific dyads or clusters of constructs. While we were not able to address this in the current manuscript, we plan to publish a practical application guide for users in the future. Despite limitations, gaps, and the need for further evolution, the updated CFIR offers much needed updates for the field.

#### Future research

The updated CFIR represents an incremental change from the original based on feedback from CFIR users, approximately two-thirds of whom have applied the CFIR in more than one study. The CFIR (and technical assistance website [110]) is a public resource and common good—free and open to all—and it must continue to evolve. We call on implementation scientists to collaborate with researchers in other disciplines (e.g., equity and justice, business, organizational science) to continue developing the CFIR, building on feedback from an ever-larger community of users. Our call for critique and reflection is echoed by others [16, 111].

Our team can help support these advances, but we do not own the framework, and we represent a narrow slice of the world. We extend an open invitation for others within alternative spheres to move the CFIR into the next generation. Further development is needed to: operationalize the framework to address equity; adapt the framework for a series of specific scenarios such as LMIC settings; map the framework to other determinant frameworks to identify gaps and resolve synonymy and polysemy issues (i.e., construct fallacies) [112]; develop qualitative, mixed, and quantitative methods for application; continue development of validated measures; establish foundations for iterative evolution and strengthen the theories encapsulated in the updated CFIR, including understanding *relationships* between constructs and with outcomes; and further exploring and establishing semantic identity for each construct [112]. Systematic reviews of empirical findings are needed to further inform or refine theoretical concepts encapsulated within and across constructs and middle-range theories need to be developed to understand interrelationships between constructs [16]. Our team and others have explored the use of causation [113] and relationship coding to highlight how determinants may interact within an implementation project [114]. For example, Kerins et al. developed an adapted version of the CFIR that included construct relationships based on a systematic review of menu labeling implementation projects [45]. Coincidence analysis and other novel analysis methods can be applied to explore clusters of constructs that lead to desired outcomes [115].

## Conclusion

The updates in the CFIR reflect feedback from a growing community of CFIR users. Although there are many updates, constructs can be mapped back to the original CFIR to ensure longitudinal consistency. We have provided resources for users to apply the updated CFIR via several additional files, and the technical assistance website will be updated to support the CFIR [110]. We are deeply grateful for past users who provided feedback and encourage future users to continue the critique and development of the CFIR as a common good.

## Supplementary Information

**Additional file 1. **Literature Review Methods.

**Additional file 2. **Consolidated Framework for Implementation Research User Survey.

**Additional file 3. **Literature Review Articles.

**Additional file 4. **Original CFIR (2009) to Updated CFIR (2022): Construct Mapping.

**Additional file 5. **User Feedback & CFIR Updates.

**Additional file 6. **Updated CFIR Domains and Constructs: Short Definitions and Detailed Descriptions.

## Data Availability

The datasets used and/or analyzed during the current study are available from the corresponding author on reasonable request.

## Abbreviations

EBI: Evidence-based innovation
LMIC: Low- and/or middle-income country
QUERI: Quality Enhancement Research Initiative
US: United States
VHA: Veterans Health Administration
