# Supplementary material for: The updated Consolidated Framework for Implementation Research based on user feedback
Source: Implement Sci. 2022 Oct 29;17:75. doi: 10.1186/s13012-022-01245-0 (PMC9617234; doi:10.1186/s13012-022-01245-0)

# Additional File 1: Literature Review Methods

Our goal was to identify studies with “meaningful use” of the CFIR, i.e., studies that demonstrated use of the CFIR via data collection, analysis, interpretation and/or to organize presentation of findings. In Phase 1, we searched SCOPUS and Web of Science (WOS) from CFIR’s initial publication year of 2009 through January 7, 2020 (see search terms below). We identified all original peer-reviewed research in English that cited the 2009 article (a forward citation tracking method) or that contained “Consolidated Framework for Implementation Research” or “CFIR” in the title and/or abstract. Settings in SCOPUS and WOS were used to exclude articles tagged as commentaries, syntheses, or protocols. The articles were imported to a citation database tool (EndNote 10) to remove duplicate articles and retrieve abstracts for screening.

A sample of articles (n=150/2600) was evaluated for meaningful use; all articles with meaningful use included “CFIR” and/or “Consolidated Framework for Implementation Research” in the title and/or abstract and were 1) Original research; 2) Systematic reviews; and/or 3) Evaluation of the CFIR as a framework. Thus, we simplified our search criteria, manually searching and removing articles that did not meet these criteria. In Phase 2, we used these criteria to search for articles in the first 6 months of 2020 in SCOPUS and WOS.

Two reviewers (MOW, CMR) read the full text of approximately 10% (n=40/376) of the included articles to independently abstract feedback on the CFIR; discrepancies with abstraction were discussed until consensus was reached. One reviewer (MOW) then read the remaining articles and abstracted all relevant passages that captured author feedback. Only 59 of 376 articles contained feedback on the CFIR (see Figure 1).

## Phase 1 Initial Search Criteria (conducted 1/7/2020)

### SCOPUS

( REF ( "fostering implementation of health services research findings into practice: a consolidated framework for advancing implementation science" ) )  OR  ( ( TITLE ( "Consolidated Framework for Implementation Research" ) OR  ABS ( "Consolidated Framework for Implementation Research" ) ) ) OR ( (TITLE ( “CFIR” ) OR ABS ( “CFIR” ) ) )  AND NOT  ( TITLE ( "Synthesis" ) )  AND NOT  ( TITLE ( "Protocol" ) )  AND NOT  ( TITLE ( "Commentary" ) )  AND  ( LIMIT-TO ( LANGUAGE ,  "English" ) )

### Web of Science

(TS=(Consolidated Framework for Implementation Research) OR TS=("fostering implementation of health services research findings into practice: a consolidated framework for advancing implementation science") OR AU=(Damschroder L.J) OR ALL=(CFIR) NOT TI=("Protocol") NOT TI=("synthesis") NOT TI=("commentary")) AND LANGUAGE: (English)
Timespan: 2009-2019

## Phase 2 Search Criteria (conducted 7/6/2020)

### SCOPUS

TITLE-ABS (“Consolidated Framework for Implementation Research” OR  CFIR )  AND  ( LIMIT-TO ( PUBYEAR ,  2020 ) )

### Web of Science

TS=("Consolidated Framework for Implementation Research" OR CFIR)

Timespan: 2020

## Figure 1: Article Identification Diagram

**Article Identification**


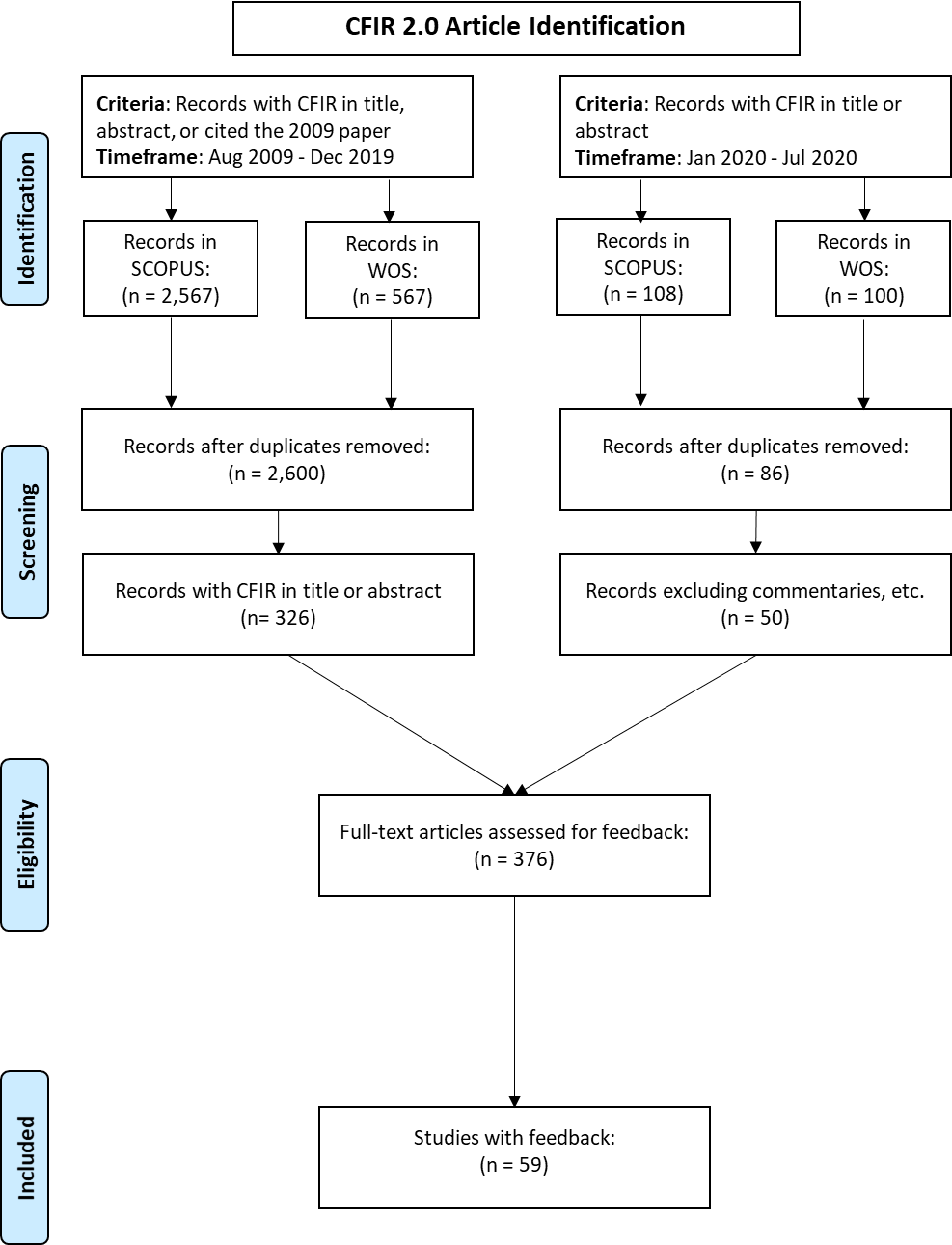

Supplement: Supplementary file 1 — Additional file 1. Literature Review Methods. [file 13012_2022_1245_MOESM1_ESM.docx]
