# Supplementary material for: The updated Consolidated Framework for Implementation Research based on user feedback
Source: Implement Sci. 2022 Oct 29;17:75. doi: 10.1186/s13012-022-01245-0 (PMC9617234; doi:10.1186/s13012-022-01245-0)
Supplement: Supplementary file 2 — Additional file 2. Consolidated Framework for Implementation Research User Survey. [file 13012_2022_1245_MOESM2_ESM.pdf]

Consolidated Framework for Implementation Research User Survey

Research Information Sheet

Please complete the survey below.

Thank you!

Please enter your name: ______________________

Please enter your email address: ______________________

The first part of the survey is about how you have used the CFIR in your work:

How many projects (e.g., research studies, quality improvement projects) have you done that used the CFIR? ______________________________

Please list publication titles: ______________________________________

Which settings have you used the CFIR in? Please check all that apply:
- ☐ Healthcare
- ☐ Public Health
- ☐ Education
- ☐ Agriculture
- ☐ Other

Please describe: ______________________________________

How have you used the CFIR? Please check all that apply:
- ☐ To guide data collection (e.g., developing an interview guide or survey items)
- ☐ To guide data analysis (e.g., coding data)
- ☐ To guide interpretation (e.g., translating findings to align with CFIR contructs)
- ☐ To design an implementation strategy
- ☐ Other

Please describe: ______________________________________

Have you used and/or developed quantitative measures based on the CFIR?
- ◯ Yes
- ◯ No

Please list publication titles: ______________________________________

Please list the name(s) of quantitative measures used: ______________________________________

Have you used the CFIR in conjunction with another theory, model, or framework?
- ◯ Yes
- ◯ No

What frameworks have you used with the CFIR? ______________________________________

Why did you use other frameworks with the CFIR? ______________________________________

The second part of the survey is about your overall assessment of the CFIR based on your own experiences with its use:

	Yes	Partially	No	Uncertain
Is the CFIR applicable across different settings (e.g., healthcare and non healthcare settings)?	○	○	○	○
Is the CFIR applicable for different types of innovations (e.g., clinical interventions, process changes, new guidelines)?	○	○	○	○
Is the CFIR more complicated than necessary?	○	○	○	○
Is the CFIR organized in a logical way that is easy to understand?	○	○	○	○
Is it easy for implementation researchers to use the CFIR?	○	○	○	○
Is it easy for people who are not implementation researchers to use the CFIR?	○	○	○	○
Is the CFIR suitable for helping people to identify and prioritize determinants of change in practice that should be considered when designing implementation strategies?	○	○	○	○
Is the CFIR likely to be useful to people designing implementation strategies?	○	○	○	○
Is the CFIR likely to be useful for reporting determinants of practice in research?	○	○	○	○
Does the CFIR help to advance or build theory?	○	○	○	○
Does the CFIR help to compare findings across studies?	○	○	○	○

Please share any comments: ____________________________

The third part of the survey is about your recommendations for existing domains and/or constructs in the CFIR.

Are the constructs and domains (groups of constructs) labeled and explained in a way that is easy to understand?

- ◯ Yes
- ◯ No
- ◯ Uncertain

Please describe any recommendations you have to revise existing domain and/or constructs names and/or definitions below.

To review the CFIR Domains and Constructs visit: https://cfirguide.org/constructs/ or download the attachment below.

[Attachment: "cfir_guide.pdf"]

I. INTERVENTION CHARACTERISTICS

A. Intervention Source ________________________

B. Evidence Strength and Quality ________________________

C. Relative Advantage ________________________

D. Adaptability ________________________

E. Trialability ________________________

F. Complexity ________________________

G. Design Quality and Packaging ________________________

H. Cost ________________________

II. OUTER SETTING

A. Patient Needs and Resources

____

B. Cosmopolitanism

____

C. Peer Pressure

____

D. External Policies and Incentives

____

III. INNER SETTING

A. Structural Characteristics

____

B. Networks and Communications

____

C. Culture

____

D. Implementation Climate

____

1. Tension for Change

____

2. Compatibility

____

3. Relative Priority

____

4. Organizational Incentives and Rewards

____

5. Goals and Feedback

____

6. Learning Climate

____

E. Readiness for Implementation

____

1. Leadership Engagement

____

2. Available Resources

____

3. Access to Knowledge and Information

____

IV. CHARACTERISTICS OF INDIVIDUALS

A. Knowledge and Beliefs about the Intervention

____

B. Self-Efficacy

____

C. Individual Stage of Change

____

D. Individual Identification with Organization

____

E. Other Personal Attributes

____

V. PROCESS

A. Planning

____

B. Engaging

1. Opinion Leaders

2. Formally Appointed Internal Implementation Leaders

3. Champions

4. External Change Agents

C. Executing

D. Reflecting and Evaluating

The final part of the survey is about your recommendations to add or remove any domains and/or constructs in the CFIR.

Are potentially important domains and/or constructs missing from the CFIR?

◯ Yes
◯ No
◯ Uncertain

Please describe any recommendations you have to ADD any domains and/or constructs to the CFIR:

____

Are domains and/or constructs included in the CFIR that should not be?

◯ Yes
◯ No
◯ Uncertain

Please describe any recommendations you have to REMOVE any domains and/or constructs from the CFIR:

____

Do you have any other recommendations for updating the CFIR or other insights you would like to share?

____
