# Supplementary material for: The updated Consolidated Framework for Implementation Research based on user feedback
Source: Implement Sci. 2022 Oct 29;17:75. doi: 10.1186/s13012-022-01245-0 (PMC9617234; doi:10.1186/s13012-022-01245-0)
Supplement: Supplementary file 3 — Additional file 3. Literature Review Articles. [file 13012_2022_1245_MOESM3_ESM.docx]

# Additional File 3: Literature Review Articles

## Published Articles with Feedback

This additional file contains a table with the articles that contained feedback on the CFIR (n=59/376) as well as a list of the articles that did not contain feedback (n=317/376). In the table, rows highlighted in blue represent work conducted in low- and/or middle-income countries (LMIC) (n=5/59) while rows highlighted in orange represent work conducted outside of traditional health systems (n=16/59).

|  | **Year** | **Author** | **Title** | **Setting** | **Country** |
| --- | --- | --- | --- | --- | --- |
|  | 2014 | Abbott, P A; Foster, J; Marin, H D F; Dykes, P C | Complexity and the science of implementation in health IT-knowledge gaps and future visions | Health Information Technology | N/A |
|  | 2019 | Acharya, S; Werts, N | Toward the Design of an Engagement Tool for Effective Electronic Health Record Adoption | Trauma Center | US |
|  | 2017 | Ament, S M C; Gillissen, F; Moser, A; Maessen, J M C; Dirksen, C D; von Meyenfeldt, M F; van der Weijden, T | Factors associated with sustainability of 2 quality improvement programs after achieving early implementation success. A qualitative case study | Hospitals | Netherlands |
|  | 2018 | Ashok, M; Hung, D; Rojas-Smith, L; Halpern, M T; Harrison, M | Framework for Research on Implementation of Process Redesigns | Primary Care | US |
|  | 2015 | Breimaier, H E; Heckemann, B; Halfens, R J G; Lohrmann, C | The Consolidated Framework for Implementation Research (CFIR): A useful theoretical framework for guiding and evaluating a guideline implementation process in a hospital-based nursing practice | Hospitals | Australia |
|  | 2020 | Callaghan-Koru J.A., Islam M., Khan M., Sowe A., Islam J., Mannan I.I., George J., Bangladesh Chlorhexidine Scale Up Study Group | Factors that influence the scale up of new interventions in low-income settings: a qualitative case study of the introduction of chlorhexidine cleansing of the umbilical cord in Bangladesh | LMIC Health Facility | Bangladesh |
|  | 2018 | Cole, C B; Pacca, J; Mehl, A; Tomasulo, A; Van Der Veken, L; Viola, A; Ridde, V | Toward communities as systems: A sequential mixed methods study to understand factors enabling implementation of a skilled birth attendance intervention in Nampula Province, Mozambique | LMIC Primary Care | Mozambique |
|  | 2015 | Dy, S M; Ashok, M; Wines, R C; Smith, L R | A framework to guide implementation Research for care transitions interventions | Hospital  to ambulatory care transitions | N/A |
|  | 2018 | Fernandez, M E; Walker, T J; Weiner, B J; Calo, W A; Liang, S; Risendal, B; Friedman, D B; Tu, S P; Williams, R S; Jacobs, S; Herrmann, A K; Kegler, M C | Developing measures to assess constructs from the Inner Setting domain of the Consolidated Framework for Implementation Research | Federally Qualified Health Centers (FQHC) | US |
|  | 2016 | Forman-Hoffman VL, Middleton JC, McKeeman JL, Stambaugh LF, Christian RB, Gaynes BN | Selected treatment strategies for emotional and behavioural disorders in children and adolescents | Pediatric Mental Health | N/A |
|  | 2020 | Godbee, K., Gunn, J., Lautenschlager, N. T., & Palmer, V. J. | Refined conceptual model for implementing dementia risk reduction: Incorporating perspectives from Australian general practice | Primary Care | Australia |
|  | 2018 | Green, H D | A Community-Based Evaluation of Screening, Brief Intervention, and Referral to Treatment (SBIRT) for the Black Community | Community | US |
|  | 2019 | Hendricks-Sturrup, R M; Mazor, K M; Sturm, A C; Lu, C Y | Barriers and facilitators to genetic testing for familial hypercholesterolemia in the United States: A review | Familial Hypercholesterolemia | US |
|  | 2016 | Henrich, N; Holmes, B; Isaac-Renton, J; Prystajecky, N | Exploring readiness for the adoption of new molecular water quality tests: Insights from interviews with policy makers, laboratory managers and watershed managers | Public Health | Canada |
|  | 2018 | Hill, J N; Locatelli, S M; Bokhour, B G; Fix, G M; Solomon, J; Mueller, N; Lavela, S L | Evaluating broad-scale system change using the Consolidated Framework for Implementation Research: Challenges and strategies to overcome them | Veterans Health Administration (VHA) | US |
|  | 2020 | Hitch D., Leech K., Neale S., Malcolm A. | Evaluating the implementation of an early supported discharge (ESD) program for stroke survivors: A mixed methods longitudinal case study | Hospital | Australia |
|  | 2019 | Ho, M; Livingston, P; Bould, M D; Nyandwi, J D; Nizeyimana, F; Uwineza, J B; Urquart, R | Barriers and facilitators to implementing a regional anesthesia service in a low-income country: a qualitative study | LMIC Healthcare System | Rwanda |
|  | 2017 | Hung, D; Gray, C; Martinez, M; Schmittdiel, J; Harrison, M I | Acceptance of lean redesigns in primary care: A contextual analysis | Veterans Health Administration (VHA) Primary Care | US |
|  | 2013 | Ilott, I; Gerrish, K; Booth, A; Field, B | Testing the Consolidated Framework for Implementation Research on health care innovations from South Yorkshire | Healthcare Settings | England |
|  | 2020 | Kerins C., McHugh S., McSharry J., Reardon C.M., Hayes C., Perry I.J., Geaney F., Seery S., Kelly C. | Barriers and facilitators to implementation of menu labelling interventions from a food service industry perspective: A mixed methods systematic review | Food Service | Ireland |
|  | 2019 | King, E S; Moore, C J; Wilson, H K; Harden, S M; Davis, M; Berg, A C | Mixed methods evaluation of implementation and outcomes in a community-based cancer prevention intervention | Community | US |
|  | 2017 | Klafke, N; Mahler, C; Von Hagens, C; Wensing, M; Schneeweiss, A; Müller, A; Szecsenyi, J; Joos, S | How the consolidated framework for implementation research can strengthen findings and improve translation of research into practice: A case study | Outpatient Cancer Clinics | Germany |
|  | 2018 | Lander, B; Wilcox, E; McAlpine, J N; Finlayson, S J; Huntsman, D G; Miller, D; Hanley, G E | Changing Clinical Practice: Evaluation of Implementing Recommendations for Opportunistic Salpingectomy in British Columbia and Ontario | Gynecology | Canada |
|  | 2019 | Leeman, J; Baquero, B; Bender, M; Choy-Brown, M; Ko, L K; Nilsen, P; Wangen, M; Birken, S A | Advancing the use of organization theory in implementation science | FQHCs | US |
|  | 2018 | Leeman, J; Wiecha, J L; Vu, M; Blitstein, J L; Allgood, S; Lee, S; Merlo, C | School health implementation tools: A mixed methods evaluation of factors influencing their use | Health Departments and School Districts | US |
|  | 2019 | Leppanen, Anne; Ekblad, Solvig; Tomson, Tanja | Tobacco Cessation on Prescription as a primary health care intervention targeting a context with socioeconomically disadvantaged groups in Sweden: A qualitative study of perceived implementation barriers and facilitators among providers | Primary Care | Sweden |
|  | 2016 | Lord, Sarah; Moore, Sarah K; Ramsey, Alex; Dinauer, Susan; Johnson, Kimberly | Implementation of a Substance Use Recovery Support Mobile Phone App in Community Settings: Qualitative Study of Clinician and Staff Perspectives of Facilitators and Barriers | Community Agencies and Centers | US |
|  | 2019 | McEachern, B M; Jackson, J; Yungblut, S; Tomasone, J R | Barriers and Facilitators to Implementing Exercise is Medicine Canada on Campus Groups | College Campuses | Canada |
|  | 2020 | Means A.R., Kemp C.G., Gwayi-Chore M.-C., Gimbel S., Soi C., Sherr K., Wagenaar B.H., Wasserheit J.N., Weiner B.J. | Evaluating and optimizing the consolidated framework for implementation research (CFIR) for use in low- And middle-income countries: A systematic review | LMICs | Review |
|  | 2019 | Merlo, Gregory; Page, Katie; Zardo, Pauline; Graves, Nicholas | Applying an Implementation Framework to the Use of Evidence from Economic Evaluations in Making Healthcare Decisions | Healthcare Decision-making | N/A |
|  | 2020 | Miake-Lye I.M., Delevan D.M., Ganz D.A., Mittman B.S., Finley E.P. | Unpacking organizational readiness for change: An updated systematic review and content analysis of assessments | Readiness Assessment | Review |
|  | 2019 | Moretto, N; Comans, T A; Chang, A T; O'Leary, S P; Osborne, S; Carter, H E; Smith, D; Cavanagh, T; Blond, D; Raymer, M | Implementation of simulation modelling to improve service planning in specialist orthopaedic and neurosurgical outpatient services | Orthopaedic and Neurosurgical Outpatient Clinics | Australia |
|  | 2019 | Morgan, D; Kosteniuk, J; O'Connell, M E; Kirk, A; Stewart, N J; Seitz, D; Bayly, M; Froehlich Chow, A; Elliot, V; Daku, J; Hack, T; Hoium, F; Kennett-Russill, D; Sauter, K | Barriers and facilitators to development and implementation of a rural primary health care intervention for dementia: A process evaluation | Rural Primary Care | Canada |
|  | 2016 | Moullin, J C; Sabater-Hernández, D; Benrimoj, S I | Qualitative study on the implementation of professional pharmacy services in Australian community pharmacies using framework analysis | Community Pharmacies | Australia |
|  | 2018 | Orlando, L A; Sperber, N R; Voils, C; Nichols, M; Myers, R A; Wu, R R; Rakhra-Burris, T; Levy, K D; Levy, M; Pollin, T I; Guan, Y; Horowitz, C R; Ramos, M; Kimmel, S E; McDonough, C W; Madden, E B; Damschroder, L J | Developing a common framework for evaluating the implementation of genomic medicine interventions in clinical care: The IGNITE Network's Common Measures Working Group | Healthcare Settings | US |
|  | 2020 | Pithara C., Farr M., Sullivan S.A., Edwards H.B., Hall W., Gadd C., Walker J., Hebden N., Horwood J. | Implementing a digital tool to support shared care planning in community-based mental health services: Qualitative evaluation | Community Mental Health | England |
|  | 2016 | Rosen, C S; Matthieu, M M; Wiltsey Stirman, S; Cook, J M; Landes, S; Bernardy, N C; Chard, K M; Crowley, J; Eftekhari, A; Finley, E P; Hamblen, J L; Harik, J M; Kehle-Forbes, S M; Meis, L A; Osei-Bonsu, P E; Rodriguez, A L; Ruggiero, K J; Ruzek, J I; Smith, B N; Trent, L; Watts, B V | A Review of Studies on the System-Wide Implementation of Evidence-Based Psychotherapies for Posttraumatic Stress Disorder in the Veterans Health Administration | Veterans Health Administration (VHA) | US |
|  | 2018 | Ruble, Lisa; McGrew, John H; Snell-Rood, Claire; Adams, Medina; Kleinert, Harold | Adapting COMPASS for Youth with ASD to Improve Transition Outcomes Using Implementation Science | School Community | US |
|  | 2016 | Sabus, C; Spake, E | Innovative physical therapy practice: A qualitative verification of factors that support diffusion of innovation in outpatient physical therapy practice | Private Outpatient PT Clinics | US |
|  | 2020 | Safaeinili, N; Brown-Johnson, C; Shaw, J G; Mahoney, M; Winget, M | CFIR simplified: Pragmatic application of and adaptations to the Consolidated Framework for Implementation Research (CFIR) for evaluation of a patient-centered care transformation within a learning health system | Learning Health Systems | US |
|  | 2019 | Santens, T; Hannes, K; Levy, S; Diamond, G; Bosmans, G | Barriers and Facilitators to Implementing Attachment-based Family Therapy into a Child Welfare Setting: A Qualitative Process Evaluation | Child Welfare | Belgium |
|  | 2018 | Serhal, E; Arena, A; Sockalingam, S; Mohri, L; Crawford, A | Adapting the consolidated framework for implementation research to create organizational readiness and implementation tools for project ECHO | Mental Health Care | Canada |
|  | 2016 | Sopcak, N; Aguilar, C; O'Brien, M A; Nykiforuk, C; Aubrey-Bassler, K; Cullen, R; Grunfeld, E; Manca, D P | Implementation of the BETTER 2 program: A qualitative study exploring barriers and facilitators of a novel way to improve chronic disease prevention and screening in primary care | Primary Care Clinics | Canada |
|  | 2011 | Sorensen, J L; Kosten, T | Developing the Tools of Implementation Science in Substance Use Disorders Treatment: Applications of the Consolidated Framework for Implementation Research | Substance Use Disorders | N/A |
|  | 2019 | Strehlenert, H; Hansson, J; Nyström, M E; Hasson, H | Implementation of a national policy for improving health and social care: A comparative case study using the Consolidated Framework for Implementation Research | Geriatric Healthcare | Sweden |
|  | 2020 | Tiderington E., Ikeda J., Lovell A. | Stakeholder Perspectives on Implementation Challenges and Strategies for Moving on Initiatives in Permanent Supportive Housing | Communities | US |
|  | 2018 | Tinc, P J; Gadomski, A; Sorensen, J A; Weinehall, L; Jenkins, P; Lindvall, K | Applying the Consolidated Framework for implementation research to agricultural safety and health: Barriers, facilitators, and evaluation opportunities | Farming | US |
|  | 2017 | Vamos, C A; Thompson, E L; Cantor, A; Detman, L; Bronson, E; Phelps, A; Louis, J M; Gregg, A R; Curran, J S; Sappenfield, W | Contextual factors influencing the implementation of the obstetrics hemorrhage initiative in Florida | Hospitals | US |
|  | 2019 | Van Deinse, T B; Bunger, A; Burgin, S; Wilson, A B; Cuddeback, G S | Using the Consolidated Framework for Implementation Research to examine implementation determinants of specialty mental health probation | Communities | US |
|  | 2015 | Varsi, C; Ekstedt, M; Gammon, D; Ruland, C M | Using the consolidated framework for implementation research to identify barriers and facilitators for the implementation of an internet-based patient-provider communication service in five settings: A qualitative study | Hospital | Norway |
|  | 2018 | Vidgen, H A; Love, P V; Wutzke, S E; Daniels, L A; Rissel, C E; Innes-Hughes, C; Baur, L A | A description of health care system factors in the implementation of universal weight management services for children with overweight or obesity: case studies from Queensland and New South Wales, Australia | Primary Care Clinics | South Wales and Queensland, Australia |
|  | 2017 | Wagner, D J; Durbin, J; Barnsley, J; Ivers, N M | Beyond quality improvement: Exploring why primary care teams engage in a voluntary audit and feedback program | Primary Care | Canada |
|  | 2019 | Wakefield, B J; Drwal, K; Paez, M; Grover, S; Franciscus, C; Reisinger, H S; Kaboli, P J; El Accaoui, R | Creating and disseminating a home-based cardiac rehabilitation program: Experience from the Veterans Health Administration | Veterans Health Administration (VHA) | US |
|  | 2018 | Ware, P; Ross, H J; Cafazzo, J A; Laporte, A; Gordon, K; Seto, E | Evaluating the implementation of a mobile phone–based telemonitoring program: Longitudinal study guided by the consolidated framework for implementation research | Specialty Clinic | Canada |
|  | 2018 | Warner, G; Lawson, B; Sampalli, T; Burge, F; Gibson, R; Wood, S | Applying the consolidated framework for implementation research to identify barriers affecting implementation of an online frailty tool into primary health care: A qualitative study | Primary Healthcare | Canada |
|  | 2020 | Wells R., Breckenridge E.D., Linder S.H. | Wellness project implementation within Houston's Faith and Diabetes initiative: a mixed methods study | Faith Communities | US |
|  | 2011 | Williams, E C; Johnson, M L; Lapham, G T; Caldeiro, R M; Chew, L; Fletcher, G S; McCormick, K A; Weppner, W G; Bradley, K A | Strategies to Implement Alcohol Screening and Brief Intervention in Primary Care Settings: A Structured Literature Review | Primary Care | N/A |
|  | 2019 | Yuan, S; Wang, F; Li, X; Jia, M; Tian, M | Facilitators and barriers to implement the family doctor contracting services in China: Findings from a qualitative study | Family Doctor Contracting Services | China |
|  | 2019 | Zitti, T; Gautier, L; Coulibaly, A; Ridde, V | Stakeholder perceptions and context of the implementation of performance-based financing in district hospitals in Mali | LMIC Hospital | Mali |

## Published Articles without Feedback

1. 2015. Abou-Malham, Sabina; Hatem, Marie; Leduc, Nicole. Analyzing barriers and facilitators to the implementation of an action plan to strengthen the midwifery professional role: a Moroccan case study
2. 2015. Abrahamson, K; De Crane, S; Mueller, C; Davila, H W; Arling, G. Implementation of a nursing home quality improvement project to reduce resident pain: A qualitative case study
3. 2020. Adamu, A A; Uthman, O A; Gadanya, M A; Wiysonge, C S. Using the consolidated framework for implementation research (CFIR) to assess the implementation context of a quality improvement program to reduce missed opportunities for vaccination in Kano, Nigeria: a mixed methods study
4. 2016. Alanis-Hirsch, K; Croff, R; Ford, J H; Johnson, K; Chalk, M; Schmidt, L; McCarty, D. Extended-Release Naltrexone: A Qualitative Analysis of Barriers to Routine Use
5. 2020. Allen P., Pilar M., Walsh-Bailey C., Hooley C., Mazzucca S., Lewis C.C., Mettert K.D., Dorsey C.N., Purtle J., Kepper M.M., Baumann A.A., Brownson R.C.. Quantitative measures of health policy implementation determinants and outcomes: A systematic review
6. 2020. Atkins D.L., Wagner A.D., Zhang J., Njuguna I.N., Neary J., Omondi V.O., Otieno V.A., Ondengʼe K., Wamalwa D.C., John-Stewart G., Slyker J.A., Beima-Sofie K.. Brief Report: Use of the Consolidated Framework for Implementation Research (CFIR) to Characterize Health Care Workers' Perspectives on Financial Incentives to Increase Pediatric HIV Testing
7. 2017. Bahr, S J; Siclovan, D M; Opper, K; Beiler, J; Bobay, K L; Weiss, M E. Interprofessional Health Team Communication about Hospital Discharge: An Implementation Science Evaluation Study
8. 2015. Baker, A M; Riekert, K A; Sawicki, G S; Eakin, M N. CF RISE: Implementing a Clinic-Based Transition Program
9. 2020. Baldwin L.-M., Schneider J.L., Schwartz M., Rivelli J.S., Green B.B., Petrik A.F., Coronado G.D.. First-year implementation of mailed FIT colorectal cancer screening programs in two Medicaid/Medicare health insurance plans: qualitative learnings from health plan quality improvement staff and leaders
10. 2018. Barac, R; Als, D; Radhakrishnan, A; Gaffey, M F; Bhutta, Z A; Barwick, M. Implementation of interventions for the control of typhoid fever in low- and middle-income countries
11. 2017. Bardosh, K L; Murray, M; Khaemba, A M; Smillie, K; Lester, R. Operationalizing mHealth to improve patient care: A qualitative implementation science evaluation of the WelTel texting intervention in Canada and Kenya
12. 2020. Baumgartner, A D; Clark, C M; LaValley, S A; Monte, S V; Wahler Jr., R G; Singh, R. Interventions to deprescribe potentially inappropriate medications in the elderly: Lost in translation?
13. 2019. Beckers, L.W.M.E.; Rameckers, E A A; Smeets, R.J.E.M.; van der Burg, J J W; Aarts, P B M; Schnackers, M.L.A.P.; Janssen-Potten, Y J M. Barriers to recruitment of children with cerebral palsy in a trial of home-based training
14. 2016. Bendall, C L; Wilson, D M; Frison, K R; Inskip, J A; Camp, P G. A partnership for Indigenous knowledge translation: Implementation of a First Nations community COPD screening day
15. 2016. Bender, A K. Using the Consolidated Framework for Implementation Research to Increase Provider Screening for Intimate Partner Violence in Rural Health Clinics
16. 2018. Benjamin Wolk, C; Van Pelt, Amelia E; Jager-Hyman, Shari; Ahmedani, Brian K; Zeber, John E; Fein, Joel A; Brown, Gregory K; Gregor, Courtney A; Lieberman, Adina; Beidas, Rinad S; Wolk, Courtney Benjamin; Van Pelt, Amelia E; Jager-Hyman, Shari; Ahmedani, Brian K; Zeber, John E; Fein, Joel A; Brown, Gregory K; Gregor, Courtney A; Lieberman, Adina; Beidas, Rinad S. Stakeholder Perspectives on Implementing a Firearm Safety Intervention in Pediatric Primary Care as a Universal Suicide Prevention Strategy A Qualitative Study
17. 2018. Bergmark, M; Bejerholm, U; Markström, U. Critical Components in Implementing Evidence-based Practice: A Multiple Case Study of Individual Placement and Support for People with Psychiatric Disabilities
18. 2020. Bergström H., Sundblom E., Elinder L.S., Norman Å., Nyberg G.. Managing Implementation of a Parental Support Programme for Obesity Prevention in the School Context: The Importance of Creating Commitment in an Overburdened Work Situation, a Qualitative Study
19. 2018. Björk Brämberg, E; Jensen, I; Kwak, L. Nationwide implementation of a national policy for evidence-based rehabilitation with focus on facilitating return to work: a survey of perceived use, facilitators, and barriers
20. 2018. Blonigen, D M; Manfredi, L; Heinz, A; Bi, X; Suarez, P; Nevedal, A L; Vashi, A A; Timko, C; Wagner, T. Reducing frequent utilization of psychiatric emergency services among veterans while maintaining quality of care
21. 2019. Boeckmann, M; Warsi, S; Noor, M; Dogar, O; Mustagfira, E H; Firoze, F; Zahid, R; Readshaw, A; Siddiqi, K; Kotz, D; Keding, A; Gabe, R; Marshall, A; Parrott, S; Swami, S; Khan, A; Raja, S; Sohail, S; Huque, R; Barua, D; Huque, S; Jahan, I; Fatima, R; Qadeer, E; Sheikh, A; Elsey, H; Karki, J; Králíková, E; Nohavova, I; Zvolska, K; Pankova, A; Baral, S; Regmi, S; Shrestha, P; Khanal, S; Joshi, B; Consortium, the T B & Tobacco. Health worker and patient views on implementation of smoking cessation in routine tuberculosis care
22. 2020. Bogren M., Banu A., Parvin S., Chowdhury M., Erlandsson K.. Implementation of a context-specific accreditation assessment tool for affirming quality midwifery education in Bangladesh: a qualitative research study
23. 2018. Bokhour, B G; Fix, G M; Mueller, N M; Barker, A M; LaVela, S L; Hill, J N; Solomon, J L; Lukas, C V. How can healthcare organizations implement patient-centered care? Examining a large-scale cultural transformation
24. 2018. Bozsik, F; Berman, M; Shook, R; Summar, S; Dewit, E; Carlson, J. Implementation contextual factors related to youth advocacy for healthy eating and active living
25. 2015. Breimaier, H E; Halfens, R J G; Lohrmann, C. Effectiveness of multifaceted and tailored strategies to implement a fall-prevention guideline into acute care nursing practice: A before-and-after, mixed-method study using a participatory action research approach
26. 2016. Breland, J Y; Asch, S M; Slightam, C; Wong, A; Zulman, D M. Key ingredients for implementing intensive outpatient programs within patient-centered medical homes: A literature review and qualitative analysis
27. 2019. Breman, R B; Low, L K; Paul, J; Johantgen, M. Promoting active labor admission: Early labor lounge implementation barriers and facilitators from the clinician perspective
28. 2014. Briand, C; Menear, M. Implementing a continuum of evidence-based psychosocial interventions for people with severe mental illness: Part 2 - Review of critical implementation issues
29. 2018. Brook, J; McGraw, C. Multidisciplinary perspectives: Application of the Consolidated Framework for Implementation Research to evaluate a health coaching initiative
30. 2020. Brown-Johnson, Cati; Safaeinili, Nadia; Zionts, Dani; Holdsworth, Laura M; Shaw, Jonathan G; Asch, Steven M; Mahoney, Megan; Winget, Marcy. The Stanford Lightning Report Method: A comparison of rapid qualitative synthesis results across four implementation evaluations
31. 2019. Burton, W; Twiddy, M; Sahota, P; Brown, J; Bryant, M. Participant engagement with a UK community-based preschool childhood obesity prevention programme: A focused ethnography study
32. 2015. Cabassa, L J; Gomes, A P; Lewis-Fernández, R. What would it take? Stakeholders' views and preferences for implementing a health care manager program in community mental health clinics under health care reform
33. 2019. Cannon, J S; Gilbert, M; Ebener, P; Malone, P S; Reardon, C M; Acosta, J; Chinman, M. Influence of an Implementation Support Intervention on Barriers and Facilitators to Delivery of a Substance Use Prevention Program
34. 2020. Carbonneau M., Carbonneau M., Eboreime E.A., Hyde A., Campbell-Scherer D., Campbell-Scherer D., Faris P., Gramlich L., Tsuyuki R.T., Tsuyuki R.T., Congly S.E., Congly S.E., Shaheen A.A., Shaheen A.A., Sadler M., Zeman M., Spiers J., Abraldes J.G., Abraldes J.G., Sugars B., Sia W., Green L., Abdellatif D., Schaefer J.P., Selvarajah V., Marr K., Ryan D., Westra Y., Bakshi N., Varghese J.C., Tandon P., Tandon P.. The cirrhosis care Alberta (CCAB) protocol: Implementing an evidence-based best practice order set for the management of liver cirrhosis - A hybrid type i effectiveness-implementation trial
35. 2018. Carter, J E; Pyati, S; Kanach, F A; Maxwell, A M W; Belden, C M; Shea, C M; Van De Ven, T; Thompson, J; Hoenig, H; Raghunathan, K. Implementation of perioperative music using the consolidated framework for implementation research
36. 2020. Cason, M; Sessions, L C; Nemeth, L; Catchpole, K; Bundy, D G. Components of team science—What contributes to success?
37. 2019. Charlson, Fiona; Chang, Odille; Kubuabola, Ilisapeci; Schess, Jaclyn; Latu, Catherine; Hunter, Ernest; Tukana, Isimeli; Qaloewai, Sefanaia; Shidhaye, Rahul. Implementation of the mental health Gap Action Programme (mhGAP) within the Fijian Healthcare System: a mixed-methods evaluation
38. 2014. Chin, M H; Goddu, A P; Ferguson, M J; Peek, M E. Expanding and Sustaining Integrated Health Care–Community Efforts to Reduce Diabetes Disparities
39. 2017. Chinman, M; Woodward, E N; Curran, G M; Hausmann, L R M. Harnessing Implementation Science to Increase the Impact of Health Equity Research
40. 2020. Chirwa E., Kapito E., Jere D.L., Kafulafula U., Chodzaza E., Chorwe-Sungani G., Gresh A., Liu L., Abrams E.T., Klima C.S., McCreary L.L., Norr K.F., Patil C.L.. An effectiveness-implementation hybrid type 1 trial assessing the impact of group versus individual antenatal care on maternal and infant outcomes in Malawi
41. 2016. Chu, C E; Wu, F; He, X; Zhou, K; Cheng, Y; Cai, W; Geng, E; Volberding, P; Tucker, J D. Hepatitis C virus treatment access among human immunodeficiency virus and hepatitis C virus (HCV)-coinfected people who inject drugs in Guangzhou, China: Implications for HCV treatment expansion
42. 2015. Cole, A M; Esplin, A; Baldwin, L.-M.. Adaptation of an evidence-based colorectal cancer screening program using the consolidated framework for implementation research
43. 2018. Colldén, C; Hellström, A; Collden, Christian; Hellstrom, Andreas. Value-based healthcare translated: A complementary view of implementation
44. 2020. Collins D., Laatikainen T., Farrington J.. Implementing essential interventions for cardiovascular disease risk management in primary healthcare: Lessons from Eastern Europe and Central Asia
45. 2018. Connell, L A; Smith, M.-C.; Byblow, W D; Stinear, C M. Implementing biomarkers to predict motor recovery after stroke
46. 2013. Cook, C; Rockwood, K. Knowledge translation: An overview and recommendations in relation to the Fourth Canadian Consensus Conference on the Diagnosis and Treatment of Dementia
47. 2019. Cooke, Alexis; Saleem, Haneefa; Hassan, Saria; Mushi, Dorothy; Mbwambo, Jessie; Lambdin, Barrot. Patient and provider perspectives on implementation barriers and facilitators of an integrated opioid treatment and HIV care intervention
48. 2016. Coronado, G D; Retecki, S; Schneider, J; Taplin, S H; Burdick, T; Green, B B. Recruiting community health centers into pragmatic research: Findings from STOP CRC
49. 2017. Coronado, G D; Schneider, J L; Petrik, A; Rivelli, J; Taplin, S; Green, B B. Implementation successes and challenges in participating in a pragmatic study to improve colon cancer screening: perspectives of health center leaders
50. 2019. Coughlin, Julia M; Shallcross, Meagan L; Schafer, Willemijn L A; Buckley, Barbara A; Stulberg, Jonah J; Holl, Jane L; Bilimoria, Karl Y; Johnson, Julie K. Minimizing Opioid Prescribing in Surgery (MOPiS) Initiative: An Analysis of Implementation Barriers
51. 2020. Coulibaly A., Coulibaly A., Coulibaly A., Gautier L., Gautier L., Zitti T., Zitti T., Zitti T., Ridde V.. Implementing performance-based financing in peripheral health centres in Mali: What can we learn from it?
52. 2019. Creason, Alia H; Ruscio, Aimee C; Tate, Karyn E; McGraw, Kate L. Accelerating Psychological Health Research Findings into Clinical Practice Through the Practice-Based Implementation Network Model
53. 2019. Crowley, K; Mishra, A; Cruz-Cano, R; Gold, R; Kleinman, D; Agarwal, R. Electronic Health Record Implementation Findings at a Large, Suburban Health and Human Services Department
54. 2020. Czosnek L., Rankin N., Zopf E., Richards J., Rosenbaum S., Cormie P.. Implementing Exercise in Healthcare Settings: The Potential of Implementation Science
55. 2018. Daaleman, T P; Brock, D; Gwynne, M; Weir, S; Dickinson, I; Willis, B; Reid, A. Implementing Lean in Academic Primary Care
56. 2011. Damschroder, L J; Hagedorn, H J. A Guiding Framework and Approach for Implementation Research in Substance Use Disorders Treatment
57. 2013. Damschroder, L J; Lowery, J C. Evaluation of a large-scale weight management program using the consolidated framework for implementation research (CFIR)
58. 2017. Damschroder, L J; Reardon, C M; Sperber, N; Robinson, C H; Fickel, J J; Oddone, E Z. Implementation evaluation of the Telephone Lifestyle Coaching (TLC) program: organizational factors associated with successful implementation
59. 2017. Dansereau, E; Miangotar, Y; Squires, E; Mimche, H; El Bcheraoui, C. Challenges to implementing Gavi's health system strengthening support in Chad and Cameroon: Results from a mixed-methods evaluation
60. 2019. Davidson, Alexandra R; Braham, Sarah; Dasey, Lauren; Reidlinger, Dianne P. Physicians' perspectives on the treatment of patients with eating disorders in the acute setting
61. 2019. Davis, Brock T; Thoma, Lynn M; Meredith, Ashley H. Provider and health care team member insights for implementing a transitions of care clinic in a federally qualified health center
62. 2020. Delaforce A., Duff J., Munday J., Hardy J.. Overcoming barriers to evidence-based patient blood management: A restricted review
63. 2019. DeSisto, Carla L; Kroelinger, Charlan D; Estrich, Cameron; Velonis, Alisa; Uesugi, Keriann; Goodman, David A; Pliska, Ellen; Akbarali, Sanaa; Rankin, Kristin M. Application of an Implementation Science Framework to Policies on Immediate Postpartum Long-Acting Reversible Contraception
64. 2017. Desveaux, L; Saragosa, M; Rogers, J; Bevan, L; Loshak, H; Moser, A; Feldman, S; Regier, L; Jeffs, L; Ivers, N M. Improving the appropriateness of antipsychotic prescribing in nursing homes: A mixed-methods process evaluation of an academic detailing intervention
65. 2015. Ditty, M S; Landes, S J; Doyle, A; Beidas, R S. It Takes a Village: A Mixed Method Analysis of Inner Setting Variables and Dialectical Behavior Therapy Implementation
66. 2016. Dogar, O; Elsey, H; Khanal, S; Siddiqi, K. Challenges of Integrating Tobacco Cessation Interventions in TB Programmes: Case Studies from Nepal and Pakistan
67. 2016. Douglas, N F. Organizational context associated with time spent evaluating language and cognitive-communicative impairments in skilled nursing facilities: Survey results within an implementation science framework
68. 2018. Drake, C; Meade, C; Hull, S K; Price, A; Snyderman, R. Integration of Personalized Health Planning and Shared Medical Appointments for Patients with Type 2 Diabetes Mellitus
69. 2019. Drew, Sarah; Judge, Andrew; Cohen, Rachel; Fitzpatrick, Raymond; Barker, Karen; Gooberman-Hill, Rachael. Enhanced Recovery After Surgery implementation in practice: an ethnographic study of services for hip and knee replacement
70. 2017. Dy, S M; Al Hamayel, N A; Hannum, S M; Sharma, R; Isenberg, S R; Kuchinad, K; Zhu, J; Smith, K; Lorenz, K A; Kamal, A H; Walling, A M; Weaver, S J. A Survey to Evaluate Facilitators and Barriers to Quality Measurement and Improvement: Adapting Tools for Implementation Research in Palliative Care Programs
71. 2020. Dzinamarira T., Kamanzi C., Mashamba-Thompson T.P.. Key stakeholders' perspectives on implementation and scale up of HIV self-testing in Rwanda
72. 2016. Edelman, E J; Hansen, N B; Cutter, C J; Danton, C; Fiellin, L E; O'Connor, P G; Williams, E C; Maisto, S A; Bryant, K J; Fiellin, D A. Implementation of integrated stepped care for unhealthy alcohol use in HIV clinics
73. 2015. Edelman, E; Hansen, N; Cutter, C; Danton, C; Fiellin, L; O'Connor, P; Williams, E; Maisto, S; Bryant, K; Fiellin, D. IMPLEMENTING INTEGRATED STEPPED CARE FOR UNHEALTHY ALCOHOL USE IN HIV TREATMENT SETTINGS: A QUALITATIVE STUDY USING THE CFIR CONSTRUCTS
74. 2019. Egholm, Cecilie Lindstrom; Helmark, Charlotte; Doherty, Patrick; Nilsen, Per; Zwisler, Ann-Dorthe; Bunkenborg, Gitte. "Struggling with practices" - a qualitative study of factors influencing the implementation of clinical quality registries for cardiac rehabilitation in England and Denmark
75. 2015. Eldh, A C; Fredriksson, M; Vengberg, S; Halford, C; Wallin, L; Dahlström, T; Winblad, U. Depicting the interplay between organisational tiers in the use of a national quality registry to develop quality of care in Sweden
76. 2020. Ellis J., Band R., Kinsella K., Cheetham-Blake T., James E., Ewings S., Rogers A.. Optimising and profiling pre-implementation contexts to create and implement a public health network intervention for tackling loneliness
77. 2020. Emerson A., Allison M., Kelly P.J., Ramaswamy M.. Barriers and facilitators of implementing a collaborative HPV vaccine program in an incarcerated population: A case study
78. 2019. Escoffery, Cam; Riehman, Kara; Watson, Lesley; Priess, A Sandy; Borne, Marcie Fisher; Halpin, Sean Nathaniel; Rhiness, Carlie; Wiggins, Emily; Kegler, Michelle C. Facilitators and Barriers to the Implementation of the HPV VACs (Vaccinate Adolescents Against Cancers) Program: A Consolidated Framework for Implementation Research Analysis
79. 2020. Esponda, G M; Hartman, S; Qureshi, O; Sadler, E; Cohen, A; Kakuma, R. Barriers and facilitators of mental health programmes in primary care in low-income and middle-income countries
80. 2018. Fernald, Douglas H; Simpson, Matthew J; Nease, Donald E; Hahn, David L; Hoffmann, Amanda E; Michaels, Leann C; Fagnan, Lyle J; Daly, Jeanette M; Levy, Barcey T. Implementing Community-Created Self-Management Support Tools in Primary Care Practices: Multimethod Analysis From the INSTTEPP Study
81. 2017. Finlay, A K; Ellerbe, L S; Wong, J J; Timko, C; Rubinsky, A D; Gupta, S; Bowe, T R; Burden, J L; Harris, A H S. Barriers to and facilitators of pharmacotherapy for alcohol use disorder in VA residential treatment programs
82. 2016. Fish, J. Co-producing knowledge about lesbian and bisexual women with breast cancer: Messages for nursing professionals from a knowledge exchange project
83. 2020. Flynn D., Joyce M., Gillespie C., Kells M., Swales M., Spillane A., Hurley J., Hayes A., Gallagher E., Arensman E., Weihrauch M.. Evaluating the national multisite implementation of dialectical behaviour therapy in a community setting: A mixed methods approach
84. 2020. Flynn D., Kells M., Joyce M., Corcoran P., Hurley J., Gillespie C., Suarez C., Swales M., Arensman E.. Multisite implementation and evaluation of 12-month standard dialectical behavior therapy in a public community setting
85. 2019. Flynn, Daniel; Kells, Mary; Joyce, Mary; Corcoran, Paul; Gillespie, Conall; Suarez, Catalina; Swales, Michaela; Arensman, Ella. Innovations in Practice: Dialectical behaviour therapy for adolescents: multisite implementation and evaluation of a 16-week programme in a public community mental health setting
86. 2014. Forman, J; Harrod, M; Robinson, C; Annis-Emeott, A; Ott, J; Saffar, D; Krein, S L; Greenstone, C L. First things first: Foundational requirements for a medical home in an academic medical center
87. 2014. Fredriksson, M; Eldh, A C; Vengberg, S; Dahlström, T; Halford, C; Wallin, L; Winblad, U. Local politico-administrative perspectives on quality improvement based on national registry data in Sweden: a qualitative study using the Consolidated Framework for Implementation Research
88. 2019. Freedman, Tamlyn; Taddio, Anna; McMurtry, C Meghan; Wong, Horace; MacDonald, Noni; McDowall, Tori; DeVlaming-Kot, Christene; Alderman, Leslie; Alfieri-Maiolo, Angela; Bucci, Lucie M; Halpert, Christine; Robson, Kate; Wilson, Evelyn; Cortes, Jocelyn; Hirji, M Mustafa; Badali, Melanie; Ilersich, Anthony N T; Ilersich, Angelo L T; Schmidt, Cathryn; Filipovic, Srdjana. Involving stakeholders in informing the development of a Knowledge Translation (KT) intervention to improve the vaccination experience at school
89. 2019. Gäbler, G; Coenen, Michaela; Fohringer, Katrin; Trauner, Michael; Stamm, Tanja A; Gaebler, Gabriele; Coenen, Michaela; Fohringer, Katrin; Trauner, Michael; Stamm, Tanja A. Towards a nationwide implementation of a standardized nutrition and dietetics terminology in clinical practice: a pre-implementation focus group study including a pretest and using the consolidated framework for implementation research
90. 2019. Gale, Randall C; Wu, Justina; Erhardt, Taryn; Bounthavong, Mark; Reardon, Caitlin M; Damschroder, Laura J; Midboe, Amanda M. Comparison of rapid vs in-depth qualitative analytic methods from a process evaluation of academic detailing in the Veterans Health Administration
91. 2018. Garbutt, J M; Dodd, S; Walling, E; Lee, A A; Kulka, K; Lobb, R. Barriers and facilitators to HPV vaccination in primary care practices: A mixed methods study using the Consolidated Framework for Implementation Research
92. 2018. Garbutt, J M; Dodd, S; Walling, E; Lee, A A; Kulka, K; Lobb, R. Theory-based development of an implementation intervention to increase HPV vaccination in pediatric primary care practices
93. 2018. Garcia, K M; Martin, E; Garney, W R; Primm, K M. Qualitative Analysis of Partnerships' Effect on Implementation of a Nationally Led Community-Based Initiative
94. 2016. Garg, Sachin K; Lyles, Courtney R; Ackerman, Sara; Handley, Margaret A; Schillinger, Dean; Gourley, Gato; Aulakh, Veenu; Sarkar, Urmimala. Qualitative analysis of programmatic initiatives to text patients with mobile devices in resource-limited health systems Healthcare Information Systems
95. 2018. Gesell, S B; Golden, S L; Limkakeng Jr., A T; Carr, C M; Matuskowitz, A; Smith, L M; Mahler, S A. Implementation of the HEART Pathway: Using the consolidated framework for implementation research
96. 2020. Gimbel S., Mocumbi A.O., Ásbjörnsdóttir K., Coutinho J., Andela L., Cebola B., Craine H., Crocker J., Hicks L., Holte S., Hossieke R., Itai E., Levin C., Manaca N., Murgorgo F., Nhumba M., Pfeiffer J., Ramiro I., Ronen K., Sotoodehnia N., Uetela O., Wagner A., Weiner B.J., Sherr K.. Systems analysis and improvement approach to optimize the hypertension diagnosis and case cascade for PLHIV individuals (SAIA-HTN): A hybrid type III cluster randomized trial
97. 2017. Gimbel, S; Mwanza, M; Nisingizwe, M P; Michel, C; Hirschhorn, L; Hingora, A; Mboya, D; Exavery, A; Tani, K; Manzi, F; Pemba, S; Phillips, J; Kante, A M; Ramsey, K; Baynes, C; Awoonor-Williams, J K; Bawah, A; Nimako, B A; Kanlisi, N; Jackson, E F; Sheff, M C; Kyei, P; Asuming, P O; Biney, A; Chilengi, R; Ayles, H; Chirwa, C; Stringer, J; Mulenga, M; Musatwe, D; Chisala, M; Lemba, M; Mutale, W; Drobac, P; Rwabukwisi, F C; Hirschhorn, L R; Binagwaho, A; Gupta, N; Nkikabahizi, F; Manzi, A; Condo, J; Farmer, D B; Hedt-Gauthier, B; Sherr, K; Cuembelo, F; Michel, C; Wagenaar, B; Henley, C; Kariaganis, M; Manuel, J L; Napua, M; Pio, A. Improving data quality across 3 sub-Saharan African countries using the Consolidated Framework for Implementation Research (CFIR): Results from the African Health Initiative
98. 2016. Gimbel, S; Rustagi, A S; Robinson, J; Kouyate, S; Coutinho, J; Nduati, R; Pfeiffer, J; Gloyd, S; Sherr, K; Adam Granato, S; Kone, A; Cruz, E; Manuel, J L; Zucule, J; Napua, M; Mbatia, G; Wariua, G; Maina, M. Evaluation of a systems analysis and improvement approach to optimize prevention of mother-to-child transmission of HIV using the consolidated framework for implementation research
99. 2019. Godbee, K; Gunn, J; Lautenschlager, N T; Curran, E; Palmer, V J. Implementing dementia risk reduction in primary care: a preliminary conceptual model based on a scoping review of practitioners' views
100. 2019. Goff, Sarah L; Unruh, Mark L; Klingensmith, Jamie; Eneanya, Nwamaka D; Garvey, Casey; Germain, Michael J; Cohen, Lewis M. Advance care planning with patients on hemodialysis: an implementation study
101. 2020. Gore R., Brown A., Wong G., Sherman S., Schwartz M., Islam N.. Integrating Community Health Workers into Safety-Net Primary Care for Diabetes Prevention: Qualitative Analysis of Clinicians’ Perspectives
102. 2018. Gransjøen, A M; Wiig, S; Lysdahl, K B; Hofmann, B M. Development and conduction of an active re-implementation of the Norwegian musculoskeletal guidelines
103. 2017. Green, S A; Bell, D; Mays, N. Identification of factors that support successful implementation of care bundles in the acute medical setting: a qualitative study
104. 2020. Gyamfi J., Allegrante J.P., Iwelunmor J., Williams O., Plange-Rhule J., Blackstone S., Ntim M., Apusiga K., Peprah E., Ogedegbe G.. Application of the Consolidated Framework for Implementation Research to examine nurses' perception of the task shifting strategy for hypertension control trial in Ghana
105. 2017. Hadjistavropoulos, H D; Nugent, M M; Dirkse, D; Pugh, N. Implementation of internet-delivered cognitive behavior therapy within community mental health clinics: A process evaluation using the consolidated framework for implementation research
106. 2019. Hagedorn, Hildi J; Wisdom, Jennifer P; Gerould, Heather; Pinsker, Erika; Brown, Randall; Dawes, Michael; Dieperink, Eric; Myrick, Donald Hugh; Oliva, Elizabeth M; Wagner, Todd H; Harris, Alex H S. Implementing alcohol use disorder pharmacotherapy in primary care settings: a qualitative analysis of provider-identified barriers and impact on implementation outcomes
107. 2015. Hagg\aard, U; Trolldal, Bjorn; Kvillemo, Pia; Guldbrandsson, Karin; Haggard, Ulrika; Trolldal, Bjorn; Kvillemo, Pia; Guldbrandsson, Karin; Hagg\aard, U; Trolldal, Bjorn; Kvillemo, Pia; Guldbrandsson, Karin. Implementation of a multicomponent Responsible Beverage Service programme in Sweden - A qualitative study of promoting and hindering factors
108. 2019. Hahn, Erin E; Munoz-Plaza, Corrine E; Schottinger, Joanne E; Brasfield, Farah M; Gould, Michael K; Parry, Carla. Developing innovative models of care for cancer survivors: use of implementation science to guide evaluation of appropriateness and feasibility
109. 2019. Haines, K J; McPeake, J; Hibbert, E; Boehm, L M; Aparanji, K; Bakhru, R N; Bastin, A J; Beesley, S J; Beveridge, L; Butcher, B W; Drumright, K; Eaton, T L; Farley, T; Firshman, P; Fritschle, A; Holdsworth, C; Hope, A A; Johnson, A; Kenes, M T; Khan, B A; Kloos, J A; Kross, E K; Mactavish, P; Meyer, J; Montgomery-Yates, A; Quasim, T; Saft, H L; Slack, A; Stollings, J; Weinhouse, G; Whitten, J; Netzer, G; Hopkins, R O; Mikkelsen, M E; Iwashyna, T J; Sevin, C M. Enablers and Barriers to Implementing ICU Follow-Up Clinics and Peer Support Groups Following Critical Illness: The Thrive Collaboratives
110. 2020. Hamovitch, E K; Acri, M; Bornheimer, L A; Falek, I; Lambert, K; Galler, M. Providers' Perspectives on Implementing a Multiple Family Group for Children with Disruptive Behavior
111. 2020. Hanna, J; Kubiak, S; Pasman, E; Gaba, A; Andre, M; Smelson, D; Pinals, D A. Evaluating the implementation of a prisoner re-entry initiative for individuals with opioid use and mental health disorders: Application of the consolidated framework for implementation research in a cross-system initiative
112. 2018. Haroon, S; Wooldridge, D; Hoogewerf, J; Nirantharakumar, K; Williams, J; Martino, L; Bhala, N. Information standards for recording alcohol use in electronic health records: Findings from a national consultation
113. 2020. Harry M.L., Saman D.M., Truitt A.R., Allen C.I., Walton K.M., O'Connor P.J., Ekstrom H.L., Sperl-Hillen J.M., Bianco J.A., Elliott T.E.. Pre-implementation adaptation of primary care cancer prevention clinical decision support in a predominantly rural healthcare system
114. 2019. Harry, Melissa L; Truitt, Anjali R; Saman, Daniel M; Henzler-Buckingham, Hillary A; Allen, Clayton I; Walton, Kayla M; Ekstrom, Heidi L; O'Connor, Patrick J; Sperl-Hillen, JoAnn M; Bianco, Joseph A; Elliott, Thomas E. Barriers and facilitators to implementing cancer prevention clinical decision support in primary care: a qualitative study
115. 2012. Hartzler, B; Lash, S J; Roll, J M. Contingency management in substance abuse treatment: A structured review of the evidence for its transportability
116. 2020. Hatch B., Tillotson C., Huguet N., Marino M., Baron A., Nelson J., Sumic A., Cohen D., E DeVoe J.. Implementation and adoption of a health insurance support tool in the electronic health record: a mixed methods analysis within a randomized trial
117. 2015. Haverhals, L M; Sayre, G; Helfrich, C D; Battaglia, C; Aron, D; Stevenson, L D; Kirsh, S; Ho, P M; Lowery, J. E-consult implementation: Lessons learned using consolidated framework for implementation research
118. 2019. Havers, S M; Martin, E; Wilson, A; Hall, L. Implementation of government-directed policy in the hospital setting: A modified Delphi study
119. 2019. Havers, Sally M; Russo, Philip L; Page, Katie; Wilson, Andrew; Hall, Lisa. Clinician perspectives of policy implementation: A qualitative study of the implementation of a national infection prevention policy in Australian hospitals
120. 2018. Helseth, S A; Janssen, T; Scott, K; Squires, D D; Becker, S J. Training community-based treatment providers to implement contingency management for opioid addiction: Time to and frequency of adoption
121. 2020. Henderson J., Hess M., Mehra K., Hawke L.D.. From Planning to Implementation of the YouthCan IMPACT Project: a Formative Evaluation
122. 2020. Henry E.G., Ngoma T., Kaiser J.L., Fong R.M., Vian T., Hamer D.H., Rockers P.C., Biemba G., Scott N.A. Evaluating implementation effectiveness and sustainability of a maternity waiting homes intervention to improve access to safe delivery in rural Zambia: A mixed-methods protocol
123. 2015. Highfield, L; Rajan, S S; Valerio, M A; Walton, G; Fernandez, M E; Bartholomew, L K. A non-randomized controlled stepped wedge trial to evaluate the effectiveness of a multi-level mammography intervention in improving appointment adherence in underserved women
124. 2018. Highfield, L; Valerio, M A; Fernandez, M E; Eldridge-Bartholomew, L K. Development of an implementation intervention using intervention mapping to increase mammography among low income women
125. 2019. Hohmeier, K C; Wheeler, J S; Turner, K; Vick, J S; Marchetti, M L; Crain, J; Brookhart, A. Targeting adaptability to improve Medication Therapy Management (MTM) implementation in community pharmacy
126. 2018. Holloway, E; Sturrock, B; Lamoureux, E; Keeffe, J; Hegel, M; Casten, R; Mellor, D; Rees, G. Can we address depression in vision rehabilitation settings? Professionals' perspectives on the barriers to integrating problem-solving treatment
127. 2019. Holt, J M; Cusatis, R; Asan, O; Williams, J; Nukuna, S; Flynn, K E; Moore, J; Crotty, B H. Incorporating patient-generated contextual data into care: Clinician perspectives using the Consolidated Framework for Implementation Science
128. 2019. Hower, Kira Isabel; Vennedey, Vera; Hillen, Hendrik Ansgar; Kuntz, Ludwig; Stock, Stephanie; Pfaff, Holger; Ansmann, Lena. Implementation of patient-centred care: which organisational determinants matter from decision maker's perspective? Results from a qualitative interview study across various health and social care organisations
129. 2020. Hu J., Ruan H., Li Q., Gifford W., Zhou Y., Yu L., Harrison D.. Barriers and facilitators to effective procedural pain treatments for pediatric patients in the Chinese context: A qualitative descriptive study
130. 2017. Huang, K.-Y.; Nakigudde, J; Rhule, D; Gumikiriza-Onoria, J L; Abura, G; Kolawole, B; Ndyanabangi, S; Kim, S; Seidman, E; Ogedegbe, G; Brotman, L M. Transportability of an Evidence-Based Early Childhood Intervention in a Low-Income African Country: Results of a Cluster Randomized Controlled Study
131. 2020. Hudson K.G., Lawton R., Hugh-Jones S.. Factors affecting the implementation of a whole school mindfulness program: A qualitative study using the consolidated framework for implementation research
132. 2015. Hunter, S B; Han, B; Slaughter, M E; Godley, S H; Garner, B R. Associations between implementation characteristics and evidence-based practice sustainment: A study of the Adolescent Community Reinforcement Approach
133. 2019. Ide, N; Hardy, V; Chirambo, G; Heavin, C; O'connor, Y; O'donoghue, J; Mastellos, N; Dharmayat, K; Andersson, B; Carlsson, S; Muula, A; Thompson, M. People welcomed this innovation with two hands: A qualitative report of an mhealth intervention for community case management in Malawi
134. 2018. Islam, N; Patel, S. Best practices for partnering with ethnic minority-serving religious organizations on health promotion and prevention
135. 2019. Jebara, T; Cunningham, S; MacLure, K; Pallivalapila, A; Awaisu, A; Al Hail, M; Stewart, D. Key stakeholders' views on the potential implementation of pharmacist prescribing: A qualitative investigation
136. 2020. Jebara, T; Cunningham, S; MacLure, K; Pallivalapila, A; Awaisu, A; Al Hail, M; Stewart, D. A modified-Delphi study of a framework to support the potential implementation of pharmacist prescribing
137. 2020. Johanson S., Markström U., Larsson M.E., Bejerholm U.. Implementation of a novel return-to-work approach for persons with affective disorders in a traditional vocational rehabilitation context: A case study
138. 2015. Jones, C M; Stewart, C; Roszell, S S. Beyond best practice implementing a unit-based CLABSI project
139. 2018. Jones, D L; Rodriguez, V J; Butts, S A; Arheart, K; Zulu, R; Chitalu, N; Weiss, S M. Increasing acceptability and uptake of voluntary male medical circumcision in Zambia: Implementing and disseminating an evidence-based intervention
140. 2020. Joudrey P.J., Oldfield B.J., Yonkers K.A., O'Connor P.G., Berland G., Edelman E.J.. Inpatient adoption of medications for alcohol use disorder: A mixed-methods formative evaluation involving key stakeholders
141. 2017. Kairy, D; Poissant, L; Rushton, P; Messier, F; Vincent, C; Zidarov, D; Ahmed, S; Fillion, B; Lavoie, V. Telerehabilitation implementation and routine clinical use: Preliminary findings from a case study across three rehabilitation centers
142. 2014. Kalkan, A; Roback, K; Hallert, E; Carlsson, P. Factors influencing rheumatologists' prescription of biological treatment in rheumatoid arthritis: an interview study
143. 2018. Kegler, M C; Beasley, D D; Liang, S; Cotter, M; Phillips, E; Hermstad, A; Williams, R; Martinez, J; Riehman, K. Using the consolidated framework for implementation research to understand safety net health system efforts to increase colorectal cancer screening rates
144. 2018. Kegler, M C; Liang, S; Weiner, B J; Tu, S P; Friedman, D B; Glenn, B A; Herrmann, A K; Risendal, B; Fernandez, M E. Measuring Constructs of the Consolidated Framework for Implementation Research in the Context of Increasing Colorectal Cancer Screening in Federally Qualified Health Center
145. 2017. Keith, R E; Crosson, J C; O'Malley, A S; Cromp, D A; Taylor, E F. Using the Consolidated Framework for Implementation Research (CFIR) to produce actionable findings: A rapid-cycle evaluation approach to improving implementation
146. 2018. Kellom, K S; Matone, M; Adejare, A; Barg, F K; Rubin, D M; Cronholm, P F. A Qualitative Exploration of Co-location as an Intervention to Strengthen Home Visiting Implementation in Addressing Maternal Child Health
147. 2020. Kempen T.G.H., Kälvemark A., Sawires M., Stewart D., Gillespie U.. Facilitators and barriers for performing comprehensive medication reviews and follow-up by multiprofessional teams in older hospitalised patients
148. 2018. Khoong, E C; Cherian, R; Smith, D E; Schillinger, D; Wolf, M S; Sarkar, U. Implementation of patient-centered prescription labeling in a safety-net ambulatory care network
149. 2018. Kien, C; Grillich, L; Nussbaumer-Streit, B; Schoberberger, R. Pathways leading to success and non-success: A process evaluation of a cluster randomized physical activity health promotion program applying fuzzy-set qualitative comparative analysis
150. 2020. King D.K., Shoup J.A., Raebel M.A., Anderson C.B., Wagner N.M., Ritzwoller D.P., Bender B.G.. Planning for Implementation Success Using RE-AIM and CFIR Frameworks: A Qualitative Study
151. 2018. King, J C; Hibbs, R; Saville, C W N; Swales, M A. The survivability of dialectical behaviour therapy programmes: A mixed methods analysis of barriers and facilitators to implementation within UK healthcare settings
152. 2018. Knobloch, M J; Chewning, B; Musuuza, J; Rees, S; Green, C; Patterson, E; Safdar, N. Leadership rounds to reduce health care–associated infections
153. 2020. Knox M., Murphy E.J., Leslie T., Wick R., Tuot D.S.. E-consult implementation success: Lessons from 5 county-based delivery systems
154. 2018. Kowalski, C P; Veeser, M; Heisler, M. Formative evaluation and adaptation of pre-and early implementation of diabetes shared medical appointments to maximize sustainability and adoption
155. 2017. Kramer, B J; Cote, S D; Lee, D I; Creekmur, B; Saliba, D. Barriers and facilitators to implementation of VA home-based primary care on American Indian reservations: A qualitative multi-case study
156. 2019. Krustev, Eugene; Dubrowski, Adam. Development and Implementation of a Three-dimensional Printed Knee Joint Simulation Model Using the Consolidated Framework for Implementation Research: Addressing Local Simulation Needs
157. 2020. Lake J.D., Rosenberg-Yunger Z.R.S., Dainty K.N., Rolf Von Den Baumen T., Everall A.C., Guilcher S.J.T.. Understanding perceptions of involving community pharmacy within an integrated care model: A qualitative study
158. 2019. Lambert-Kerzner, A C; Aasen, D M; Overbey, D M; Damschroder, L J; Henderson, W G; Hammermeister, K E; Bronsert, M R; Meguid, R A. Use of the consolidated framework for implementation research to guide dissemination and implementation of new technologies in surgery
159. 2019. Lamontagne, M.-E.; Best, K L; Clarke, T; Dumont, F S; Noreau, L. Implementation Evaluation of an Online Peer-Mentor Training Program for Individuals With Spinal Cord Injury
160. 2011. Lash, S J; Timko, C; Curran, G M; McKay, J R; Burden, J L. Implementation of Evidence-Based Substance Use Disorder Continuing Care Interventions
161. 2016. Latif, A; Waring, J; Watmough, D; Barber, N; Chuter, A; Davies, J; Salema, N.-E.; Boyd, M J; Elliott, R A. Examination of England's New Medicine Service (NMS) of complex health care interventions in community pharmacy
162. 2018. Latif, Asam. Community pharmacy Medicines Use Review: current challenges
163. 2017. Lawson, B; Sampalli, T; Wood, S; Warner, G; Moorhouse, P; Gibson, R; Mallery, L; Burge, F; Bedford, L G. Evaluating the implementation and feasibility of a web-based tool to support timely identification and care for the frail population in primary healthcare settings
164. 2018. Lee, R M; Ramanadhan, S; Kruse, G R; Deutsch, C. A mixed methods approach to evaluate partnerships and implementation of the Massachusetts prevention and wellness trust fund
165. 2020. Leeman J., Askelson N., Ko L.K., Rohweder C.L., Avelis J., Best A., Friedman D., Glanz K., Seegmiller L., Stradtman L., Vanderpool R.C.. Understanding the processes that Federally Qualified Health Centers use to select and implement colorectal cancer screening interventions: a qualitative study
166. 2017. Leeman, J; Birken, S A; Powell, B J; Rohweder, C; Shea, C M. Beyond "implementation strategies": Classifying the full range of strategies used in implementation science and practice
167. 2020. Leung E., Wanner K.J., Senter L., Brown A., Middleton D.. What will it take? Using an implementation research framework to identify facilitators and barriers in implementing a school-based referral system for sexual health services
168. 2018. Li, S.-A.; Jeffs, L; Barwick, M; Stevens, B. Organizational contextual features that influence the implementation of evidence-based practices across healthcare settings: A systematic integrative review
169. 2016. Liang, S; Kegler, M C; Cotter, M; Emily, P; Beasley, D; Hermstad, A; Morton, R; Martinez, J; Riehman, K; Phillips, E; Beasley, D; Hermstad, A; Morton, R; Martinez, J; Riehman, K. Integrating evidence-based practices for increasing cancer screenings in safety net health systems: A multiple case study using the Consolidated Framework for Implementation Research
170. 2017. Lind, S; Wallin, L; Brytting, T; Fürst, C J; Sandberg, J. Implementation of national palliative care guidelines in Swedish acute care hospitals: A qualitative content analysis of stakeholders' perceptions
171. 2015. Lindholm, L H; Koivukangas, A; Lassila, A; Kampman, O. Early assessment of implementing evidence-based brief therapy interventions among secondary service psychiatric therapists
172. 2017. Liu, S S; Meyerson, B; King, J; Yih, Y; Ostovari, M. Drivers and Barriers for Adopting Accreditation at Local Health Departments for Their Performance Improvement Effort
173. 2017. Lodge, A C; Kaufman, L; Stevens Manser, S. Barriers to Implementing Person-Centered Recovery Planning in Public Mental Health Organizations in Texas: Results from Nine Focus Groups
174. 2020. Louie E., Barrett E.L., Baillie A., Haber P., Morley K.C.. Implementation of evidence-based practice for alcohol and substance use disorders: Protocol for systematic review
175. 2019. Louie, D L; Assefa, M T; McGovern, M P. Attitudes of primary care physicians toward prescribing buprenorphine: A narrative review
176. 2017. Lovelock, Kirsten; Martin, Greg; Gauld, Robin; MacRae, Jayden. Better, Sooner, More Convenient? The reality of pursuing greater integration between primary and secondary healthcare providers in New Zealand
177. 2019. Low, Lee Lan; Ab Rahim, Fathullah Iqbal; Johari, Mohammad Zabri; Abdullah, Zalilah; Aziz, Siti Hajar Abdul; Suhaimi, Nur Ajeerah; Jaafar, Norrafizah; Hanafiah, Ainul Nadziha Mohd; Kong, Yuke Lin; Mahmud, Siti Haniza; Zulkepli, Mohamad Zaidan; Perialathan, Komathi; Muharam, Norazlin; Zainudin, Nur Hani; Zin, Zaikiah Mohd; Roslan, Norazilah Mohd; Aris, Tahir; Murad, Shahnaz. Assessing receptiveness to change among primary healthcare providers by adopting the consolidated framework for implementation research (CFIR)
178. 2019. Lowenstein, M; Feuerstein-Simon, R; Sheni, R; Dupuis, R; Kinsey, E W; Luna Marti, X; Cannuscio, C. Public libraries as partners in confronting the overdose crisis: A qualitative analysis
179. 2020. Luc, M; Corriveau, H; Boire, G; Filiatrault, J; Beaulieu, M.-C.; Dagenais, P; Gaboury, I. Implementing a fracture follow-up liaison service: perspective of key stakeholders
180. 2014. Luck, J; Bowman, C; York, L; Midboe, A; Taylor, T; Gale, R; Asch, S. Multimethod evaluation of the VA's peer-to-peer toolkit for patient-centered medical home implementation
181. 2016. Maguire, E M; Bokhour, B G; Wagner, T H; Asch, S M; Gifford, A L; Gallagher, T H; Durfee, J M; Martinello, R A; Elwy, A R. Evaluating the implementation of a national disclosure policy for large-scale adverse events in an integrated health care system: identification of gaps and successes
182. 2019. Malham, S A; Smithman, M.-A.; Touati, N; Brousselle, A; Loignon, C; Dubois, C.-A.; Nour, K; Boivin, A; Breton, M. Explaining variation of implementation outcomes of centralized waiting lists for unattached patients: A comparative multiple case study in Quebec
183. 2020. Margolis K.L., Crain A.L., Bergdall A.R., Beran M., Anderson J.P., Solberg L.I., O'Connor P.J., Sperl-Hillen J.M., Pawloski P.A., Ziegenfuss J.Y., Rehrauer D., Norton C., Haugen P., Green B.B., McKinney Z., Kodet A., Appana D., Sharma R., Trower N.K., Williams R., Crabtree B.F.. Design of a pragmatic cluster-randomized trial comparing telehealth care and best practice clinic-based care for uncontrolled high blood pressure
184. 2019. Marhefka, Stephanie L; Sharma, Vinita; Schafer, Ellen J; Turner, DeAnne; Falope, Oluyemisi; Louis-Jacques, Adetola; Wachira, Mary M; Livingston, Taylor; Roig-Romero, Regina Maria. Why do we need a policy?' Administrators' perceptions on breast-feeding-friendly childcare
185. 2016. Marques, L; Dixon, L; Valentine, S E; Borba, C P C; Simon, N M; Stirman, S W. Providers' perspectives of factors influencing implementation of evidence-based treatments in a community mental health setting: A qualitative investigation of the training-practice gap
186. 2017. Martinez, C; Bacigalupe, G; Cortada, J M; Grandes, G; Sanchez, A; Pombo, H; Bully, P. The implementation of health promotion in primary and community care: a qualitative analysis of the ‘Prescribe Vida Saludable' strategy
187. 2019. Maxwell, Annette E; Santifer, Rhonda; Chang, L Cindy; Gatson, Juana; Crespi, Catherine M; Lucas-Wright, Aziza. Organizational readiness for wellness promotion - a survey of 100 African American church leaders in South Los Angeles
188. 2019. Mc Sween-Cadieux, E; Dagenais, C; Somé, D T; Ridde, V. A health knowledge brokering intervention in a district of Burkina Faso: A qualitative retrospective implementation analysis
189. 2017. McClung, L; Obasi, C; Knobloch, M J; Safdar, N. Health care worker perspectives of their motivation to reduce health care–associated infections
190. 2019. McKay, H; Naylor, P.-J.; Lau, E; Gray, S M; Wolfenden, L; Milat, A; Bauman, A; Race, D; Nettlefold, L; Sims-Gould, J. Implementation and scale-up of physical activity and behavioural nutrition interventions: An evaluation roadmap
191. 2017. McKee, G; Codd, M; Dempsey, O; Gallagher, P; Comiskey, C. Describing the implementation of an innovative intervention and evaluating its effectiveness in increasing research capacity of advanced clinical nurses: Using the consolidated framework for implementation research
192. 2019. Messing, Barbara Pisano; Ward, Elizabeth C; Lazarus, Cathy; Ryniak, Keri; Kim, Melissa; Silinonte, Jessica; Gold, Dorothy; Thompson, Carol B; Pitman, Karen T; Blanco, Ray; Sobel, Ryan; Harrer, Karen; Ulmer, Karen; Neuner, Geoffrey; Patel, Kruti; Tang, Mei; Lee, Gregory. Establishing a Multidisciplinary Head and Neck Clinical Pathway: An Implementation Evaluation and Audit of Dysphagia-Related Services and Outcomes
193. 2020. Meyerson B.E., Agley J.D., Jayawardene W., Eldridge L.A., Arora P., Smith C., Vadiei N., Kennedy A., Moehling T., PharmNet Research Team. Feasibility and acceptability of a proposed pharmacy-based harm reduction intervention to reduce opioid overdose, HIV and hepatitis C
194. 2011. Midboe, A M; Cucciare, M A; Trafton, J A; Ketroser, N; Chardos, J F. Implementing motivational interviewing in primary care: The role of provider characteristics
195. 2018. Midboe, A M; Wu, J; Erhardt, T; Carmichael, J M; Bounthavong, M; Christopher, M L D; Gale, R C. Academic detailing to improve opioid safety: Implementation lessons from a qualitative evaluation
196. 2020. Morrow A., Morrow A., Tucker K.M., Tucker K.M., Shaw T.J., Parkinson B., Abraham C., Wolfenden L., Taylor N., Taylor N.. Understanding implementation success: Protocol for an in-depth, mixed-methods process evaluation of a cluster randomised controlled trial testing methods to improve detection of Lynch syndrome in Australian hospitals
197. 2018. Mosson, R; Von Thiele Schwarz, U; Richter, A; Hasson, H. The impact of inner and outer context on line managers' implementation leadership
198. 2017. Mudge, S; Rewi, D; Channon, A. Identifying an outcome measure to assess the impact of Mobility Dogs
199. 2014. Murphy, A L; Gardner, D M; Kutcher, S P; Martin-Misener, R. A theory-informed approach to mental health care capacity building for pharmacists
200. 2019. Musinguzi, Geofrey; Wanyenze, Rhoda K; Ndejjo, Rawlance; Ssinabulya, Isaac; van Marwijk, Harm; Ddumba, Isaac; Bastiaens, Hilde; Nuwaha, Fred. An implementation science study to enhance cardiovascular disease prevention in Mukono and Buikwe districts in Uganda: a stepped-wedge design
201. 2020. Mwangi N., Bascaran C., Ramke J., Kipturgo M., Kim M., Ng'Ang'A M., Gichuhi S., Mutie D., Moorman C., Muthami L., Foster A.. Peer-support to increase uptake of screening for diabetic retinopathy: Process evaluation of the DURE cluster randomized trial
202. 2018. Naidoo, N; Zuma, N; Khosa, N S; Marincowitz, G; Railton, J; Matlakala, N; Jobson, G A; Igumbor, J O; McIntyre, J A; Struthers, H E; Peters, R P H. Qualitative assessment of facilitators and barriers to HIV programme implementation by community health workers in Mopani district, South Africa
203. 2017. Nathan, N; Wiggers, J; Wyse, R; Williams, C M; Sutherland, R; Yoong, S L; Lecathelinais, C; Wolfenden, L. Factors associated with the implementation of a vegetable and fruit program in a population of Australian elementary schools
204. 2017. Nolan, M B; Warner, D O. Perioperative tobacco use treatments: Putting them into practice
205. 2016. Norman, \AA; Nyberg, G; Elinder, L S; Berlin, A. One size does not fit all-qualitative process evaluation of the Healthy School Start parental support programme to prevent overweight and obesity among children in disadvantaged areas in Sweden Energy balance-related behaviors
206. 2016. Northridge, M E; Birenz, S; Gomes, D M; Golembeski, C A; Greenblatt, A P; Shelley, D; Russell, S L. Views of Dental Providers on Primary Care Coordination at Chairside: A Pilot Study
207. 2017. Northridge, M E; Kavathe, R; Zanowiak, J; Wyatt, L; Singh, H; Islam, N. Implementation and dissemination of the Sikh American Families Oral Health Promotion Program
208. 2016. Northridge, M E; Kum, S S; Chakraborty, B; Greenblatt, A P; Marshall, S E; Wang, H; Kunzel, C; Metcalf, S S. Third Places for Health Promotion with Older Adults: Using the Consolidated Framework for Implementation Research to Enhance Program Implementation and Evaluation
209. 2017. Northridge, M E; Shedlin, M; Schrimshaw, E W; Estrada, I; De La Cruz, L; Peralta, R; Birdsall, S; Metcalf, S S; Chakraborty, B; Kunzel, C. Recruitment of racial/ethnic minority older adults through community sites for focus group discussions
210. 2015. Nouwens, E; Van Lieshout, J; Wensing, M. Determinants of impact of a practice accreditation program in primary care: A qualitative study
211. 2019. Nyame, Solomon; Iwelunmor, Juliet; Ogedegbe, Gbenga; Adjei, Kezia Gladys Amaning; Adjei, Kwame; Apusiga, Kingsley; Gyamfi, Joyce; Asante, Kwaku Poku; Plange-Rhule, Jacob. Capacity and Readiness for Implementing Evidence-Based Task-Strengthening Strategies for Hypertension Control in Ghana A Cross-Sectional Study
212. 2020. Okamoto, S K; Helm, S; Chin, S K; Hata, J; Hata, E; Okamura, K H. The implementation of a culturally grounded, school-based, drug prevention curriculum in rural Hawai‘i
213. 2019. Oswald, Jennifer M; Boswell, James F; Smith, Melanie; Thompson-Brenner, Heather; Brooks, Gayle. Practice-Research Integration in the Residential Treatment of Patients With Severe Eating and Comorbid Disorders
214. 2018. Padwa, H; Kaplan, C D. Translating Science to Practice: Lessons Learned Implementing Evidence-Based Treatments in US Substance Use Disorder Treatment Programs
215. 2014. Palacio, Ana; Keller, Vaughn F; Chen, Jessica; Tamariz, Leonardo; Carrasquillo, Olveen; Tanio, Craig. Can Physicians Deliver Chronic Medications at the Point of Care?
216. 2020. Palm H.C., Degnan J.H., Biefeld S.D., Reese A.L., Espey E., Hofler L.G.. An initiative to implement immediate postpartum long-acting reversible contraception in rural New Mexico
217. 2019. Palmer, Jennifer A; Parker, Victoria A; Mor, Vincent; Volandes, Angelo E; Barre, Lacey R; Belanger, Emmanuelle; Carter, Phoebe; Loomer, Lacey; McCreedy, Ellen; Mitchell, Susan L. Barriers and facilitators to implementing a pragmatic trial to improve advance care planning in the nursing home setting
218. 2020. Pascoe S.J.S., Scott N.A., Fong R.M., Murphy J., Huber A.N., Moolla A., Phokojoe M., Gorgens M., Rosen S., Wilson D., Pillay Y., Fox M.P., Fraser-Hurt N.. “Patients are not the same, so we cannot treat them the same” – A qualitative content analysis of provider, patient and implementer perspectives on differentiated service delivery models for HIV treatment in South Africa
219. 2018. Patel, Sapana R; Margolies, Paul J; Covell, Nancy H; Lipscomb, Cristine; Dixon, Lisa B. Using Instructional Design, Analyze, Design, Develop, Implement, and Evaluate, to Develop e-Learning Modules to Disseminate Supported Employment for Community Behavioral Health Treatment Programs in New York State
220. 2018. Patey, Chris; Norman, Paul; Bishop, Nicole; Bartellas, Michael; Dubrowski, Adam. Development, Evaluation, and Implementation of a New 3D Printed Tongue Depressor Dispenser
221. 2017. Patterson, J; Holdford, D. Understanding the dissemination of appointment-based synchronization models using the CFIR framework
222. 2020. Pauly B., Wallace B., Pagan F., Phillips J., Wilson M., Hobbs H., Connolly J.. Impact of overdose prevention sites during a public health emergency in Victoria, Canada
223. 2020. Percy J.N., Crain J., Rein L., Hohmeier K.C.. The impact of a pharmacist-extender training program to improve pneumococcal vaccination rates within a community chain pharmacy
224. 2017. Perez, C; Kaizer, F; Archambault, P; Fung, J. A novel approach to integrate VR exer-games for stroke rehabilitation: Evaluating the implementation of a 'games room'
225. 2019. Petersen, Inge; van Rensburg, Andre; Kigozi, Fred; Semrau, Maya; Hanlon, Charlotte; Abdulmalik, Jibnl; Kola, Lola; Fekadu, Abebaw; Gureje, Oye; Gurung, Dristy; Jordans, Mark; Mntambo, Ntokozo; Mugisha, James; Muke, Shital; Petrus, Ruwayda; Shidhaye, Rahul; Ssebunnya, Joshua; Tekola, Bethlehem; Upadhaya, Nawaraj; Patel, Vikram; Lund, Crick; Thornicroft, Graham. Scaling up integrated primary mental health in six low- and middle-income countries: obstacles, synergies and implications for systems reform
226. 2018. Pinto, C; Bristowe, K; Witt, J; Davies, J M; de Wolf-Linder, S; Dawkins, M; Guo, P; Higginson, I J; Daveson, B; Murtagh, F E M. Perspectives of patients, family caregivers and health professionals on the use of outcome measures in palliative care and lessons for implementation: a multi-method qualitative study
227. 2019. Poncette, A.-S.; Meske, C; Mosch, L; Balzer, F. How to Overcome Barriers for the Implementation of New Information Technologies in Intensive Care Medicine
228. 2017. Possemato, K; Kuhn, E; Johnson, E M; Hoffman, J E; Brooks, E. Development and refinement of a clinician intervention to facilitate primary care patient use of the PTSD Coach app
229. 2020. Pratt-Chapman M.L.. Implementation of sexual and gender minority health curricula in health care professional schools: A qualitative study
230. 2019. Radovic, A; Odenthal, K; Flores, A T; Miller, E; Stein, B D. Prescribing Technology to Increase Uptake of Depression Treatment in Primary Care: A Pre-implementation Focus Group Study of SOVA (Supporting Our Valued Adolescents)
231. 2019. Rafferty, M R; MacDonald, J; Byskosh, A; Sloan, L; Toledo, S; Marciniak, C; Simuni, T. Using Implementation Frameworks to Provide Proactive Physical Therapy for People With Parkinson Disease: Case Report
232. 2019. Ralph, N; Chambers, S; Pomery, A; Oliffe, J; Dunn, J. Nurse-led supportive care intervention for men with advanced prostate cancer
233. 2020. Ramaswamy, M; Allison, M; Musser, B; Satterwhite, C; Armstrong, R; Kelly, P J. Local Health Department Interest in Implementation of a Jail-Based Human Papillomavirus Vaccination Program in Kansas, Iowa, Missouri, and Nebraska
234. 2016. Ramsey, A; Lord, S; Torrey, J; Marsch, L; Lardiere, M. Paving the Way to Successful Implementation: Identifying Key Barriers to Use of Technology-Based Therapeutic Tools for Behavioral Health Care
235. 2016. Rankin, K M; Kroelinger, C D; DeSisto, C L; Pliska, E; Akbarali, S; Mackie, C N; Goodman, D A. Application of Implementation Science Methodology to Immediate Postpartum Long-Acting Reversible Contraception Policy Roll-Out Across States
236. 2017. Rattray, N A; Damush, T M; Luckhurst, C; Bauer-Martinez, C J; Homoya, B J; Miech, E J. Prime movers: Advanced practice professionals in the role of stroke coordinator
237. 2020. Richmond S.A., Donaldson A., Macpherson A., Bridel W., van den Berg C., Finch C.F., Hagel B., Emery C.A.. Facilitators and Barriers to the Implementation of iSPRINT: A Sport Injury Prevention Program in Junior High Schools
238. 2016. Riley, M; Laurie, A R; Plegue, M A; Richarson, C R. The Adolescent "Expanded Medical Home": School-Based Health Centers Partner with a Primary Care Clinic to Improve Population Health and Mitigate Social Determinants of Health
239. 2016. Ritchie, C; Andersen, R; Eng, J; Garrigues, S K; Intinarelli, G; Kao, H; Kawahara, S; Patel, K; Sapiro, L; Thibault, A; Tunick, E; Barnes, D E. Implementation of an interdisciplinary, team-based complex Care Support health Care model at an academic medical center: Impact on health care utilization and quality of life
240. 2013. Robins, L S; Jackson, J E; Green, B B; Korngiebel, D; Force, R W; Baldwin, L.-M.. Barriers and facilitators to evidence-based blood pressure control in community practice
241. 2020. Rogers L., De Brún A., McAuliffe E.. Development of an integrative coding framework for evaluating context within implementation science
242. 2019. Rogers, E; Aidasani, S R; Friedes, R; Hu, L; Langford, A T; Moloney, D N; Orzeck-Byrnes, N; Sevick, M A; Levy, N. Barriers and facilitators to the implementation of a mobile insulin titration intervention for patients with uncontrolled diabetes: A qualitative analysis
243. 2019. Rogers, L Q; Goncalves, L; Martin, M Y; Pisu, M; Smith, T L; Hessong, D; Oster, R A; Qu, H; Shewchuk, R; Iqbal, F; Sheffield, M E; Minter, A; Baumann, A A. Beyond efficacy: a qualitative organizational perspective on key implementation science constructs important to physical activity intervention translation to rural community cancer care sites
244. 2019. Rosenstein, M G. Application of implementation science to OB/GYN quality improvement efforts
245. 2012. Ruffolo, M C; Capobianco, J. Moving an Evidence-Based Intervention Into Routine Mental Health Care: A Multifaceted Case Example
246. 2015. Russell, S L; Greenblatt, A P; Gomes, D; Birenz, S; Golembeski, C A; Shelley, D; McGuirk, M; Eisenberg, E; Northridge, M E. Toward Implementing Primary Care at Chairside: Developing a Clinical Decision Support System for Dental Hygienists
247. 2017. Saluja, S; Silverstein, A; Mukhopadhyay, S; Lin, Y; Raykar, N; Keshavjee, S; Samad, L; Meara, J G. Using the consolidated framework for implementation research to implement and evaluate national surgical planning
248. 2014. Sanchez, S H; Sethi, S S; Santos, S L; Boockvar, K. Implementing medication reconciliation from the planner's perspective: A qualitative study
249. 2020. Sarker M., Saha A., Matin M., Mehjabeen S., Tamim M.A., Sharkey A.B., Kim M., Nyankesha E.U., Widiati Y., Shahabuddin A.S.M.. Effective maternal, newborn and child health programming among Rohingya refugees in Cox's Bazar, Bangladesh: Implementation challenges and potential solutions
250. 2020. Sebastian S., Thomas D.P., Brimblecombe J., Majoni V., Cunningham F.C.. Factors impacting on development and implementation of training programs for health professionals to deliver brief interventions, with a focus on programs developed for indigenous clients: A literature review
251. 2018. Selick, Avra; Durbin, Janet; Casson, Ian; Lee, Jacques; Lunsky, Yona. Barriers and facilitators to improving health care for adults with intellectual and developmental disabilities: what do staff tell us?
252. 2017. Selove, R; Foster, M; Mack, R; Sanderson, M; Hull, P C. Using an implementation research framework to identify potential facilitators and barriers of an intervention to increase HPV vaccine uptake
253. 2014. Selove, Rebecca; Foster, Maya; Sanderson, Maureen; Hull, Pamela. Using the Consolidated Framework for Implementation Research (CFIR) to identify potential facilitators and barriers of an intervention to increase HPV vaccine uptake
254. 2019. Shade, L; Ludden, T; Dolor, R J; Halladay, J; Reeves, K; Rees, J; Hendrickson, L; Bray, P; Tapp, H. Using the Consolidated Framework for Implementation Research (CFIR) to evaluate implementation effectiveness of a facilitated approach to an asthma shared decision making intervention
255. 2018. Shea, C M; Tabriz, A A; Turner, K; North, S; Reiter, K L. Telestroke Adoption Among Community Hospitals in North Carolina: A Cross-Sectional Study
256. 2014. Sherr, K; Gimbel, S; Rustagi, A; Nduati, R; Cuembelo, F; Farquhar, C; Wasserheit, J; Gloyd, S; Koné, A; Robinson, J; Granato, A; Kouyate, S; Mbatia, G; Wariua, G; Maina, M; Njuguna, P M; Coutinho, J; Cruz, E; Jamnadas, M; Zucule, J; Michel, C; Wagenaar H., B H; Pfeiffer, J. Systems analysis and improvement to optimize pMTCT (SAIA): A cluster randomized trial
257. 2019. Sherr, Kenneth; Asbjornsdottir, Kristjana; Crocker, Jonny; Coutinho, Joana; Cuembelo, Maria de Fatima; Tavede, Esperanca; Manaca, Nelia; Ronen, Keshet; Murgorgo, Felipe; Barnabas, Ruanne; John-Stewart, Grace; Holte, Sarah; Weiner, Bryan J; Pfeiffer, James; Gimbel, Sarah. Scaling-up the Systems Analysis and Improvement Approach for prevention of mother-to-child HIV transmission in Mozambique (SAIA-SCALE): a stepped-wedge cluster randomized trial
258. 2016. Shi, J; Jiang, C; Tan, D; Yu, D; Lu, Y; Sun, P; Pan, Y; Zhang, H; Wang, Z; Yang, B. Advancing Implementation of Evidence-Based Public Health in China: An Assessment of the Current Situation and Suggestions for Developing Regions
259. 2017. Shoemaker, S J; Curran, G M; Swan, H; Teeter, B S; Thomas, J. Application of the Consolidated Framework for Implementation Research to community pharmacy: A framework for implementation research on pharmacy services
260. 2019. Shue, Sarah A; McGuire, Alan B; Matthias, Marianne S. Facilitators and Barriers to Implementation of a Peer Support Intervention for Patients with Chronic Pain: A Qualitative Study
261. 2016. Siantz, E; Henwood, B; Gilmer, T. Implementation of peer providers in integrated mental health and primary care settings
262. 2020. Sico I.P., Hall B.J., Aguilar-González A., Orozco M., Ramirez C., Baumgartner J.N., Boyd D., Bolaños J., Calgua E., Lou-Meda R., Rice H.E.. Implementation Analysis of a Perioperative Patient Safety Program in Guatemala
263. 2016. Smith, S G; Side, L; Meisel, S F; Horne, R; Cuzick, J; Wardle, J. Clinician-reported barriers to implementing breast cancer chemoprevention in the UK: A qualitative investigation
264. 2015. Sohlberg, M M; Kucheria, P; Fickas, S; Wade, S L. Developing brain injury interventions on both ends of the treatment continuum depends upon early research partnerships and feasibility studies
265. 2018. Soi, C; Gimbel, S; Chilundo, B; Muchanga, V; Matsinhe, L; Sherr, K. Human papillomavirus vaccine delivery in Mozambique: Identification of implementation performance drivers using the Consolidated Framework for Implementation Research (CFIR)
266. 2020. Sombié I., Degroote S., Somé P.A., Ridde V.. Analysis of the implementation of a community-based intervention to control dengue fever in Burkina Faso
267. 2019. Standiford, T; Conte, M L; Billi, J E; Sales, A; Barnes, G D. Integrating Lean Thinking and Implementation Science Determinants Checklists for Quality Improvement: A Scoping Review
268. 2018. Stanhope, V; Manuel, J I; Jessell, L; Halliday, T M. Implementing SBIRT for adolescents within community mental health organizations: A mixed methods study
269. 2017. Steffen, K; Doctor, A; Hoerr, J; Gill, J; Markham, C; Brown, S M; Cohen, D; Hansen, R; Kryzer, E; Richards, J; Small, S; Valentine, S; York, J L; Proctor, E K; Spinella, P C. Controlling phlebotomy volume diminishes PICU transfusion: Implementation processes and impact
270. 2018. Stephan, A.-J.; Kovacs, E; Phillips, A; Schelling, J; Ulrich, S M; Grill, E. Barriers and facilitators for the management of vertigo: A qualitative study with primary care providers
271. 2018. Stevenson, L; Ball, S; Haverhals, L M; Aron, D C; Lowery, J. Evaluation of a national telemedicine initiative in the Veterans Health Administration: Factors associated with successful implementation
272. 2016. Stirman, S W; Gutner, C A; Langdon, K; Graham, J R. Bridging the Gap Between Research and Practice in Mental Health Service Settings: An Overview of Developments in Implementation Theory and Research
273. 2018. Stokes, T; Tumilty, E; Doolan-Noble, F; Gauld, R. HealthPathways implementation in a New Zealand health region: A qualitative study using the Consolidated Framework for Implementation Research
274. 2020. Sturesson M., Bylund S.H., Edlund C., Falkdal A.H., Bernspång B.. Collaboration in work ability assessment for sick-listed persons in primary healthcare
275. 2015. Tabak, Rachel G; Moreland-Russell, Sarah. Food Service Perspectives on National School Lunch Program Implementation
276. 2018. Tabak, Rachel G; Schwarz, Cynthia D; Carter, Ebony; Haire-Joshu, Debra. Context for Implementing a Gestational Weight Gain Program Nationally
277. 2018. Talbot, E; Bird, Y; Russell, J; Sahota, K; Schneider, J; Khalifa, N. Implementation of individual placement and support (IPS) into community forensic mental health settings: Lessons learned
278. 2018. Teeters, L A; Heerman, W J; Schlundt, D; Harris, D; Barkin, S L. Community readiness assessment for obesity research: Pilot implementation of the Healthier Families programme
279. 2019. Thompson, Tess; Kreuter, Matthew W; Caito, Nicole; Williams, Rebecca S; Escoffery, Cam; Fernandez, Maria E; Kegler, Michelle C. Implementing an Evidence-based Tobacco Control Program at Five 2-1-1 Call Centers: An Evaluation Using the Consolidated Framework for Implementation Research
280. 2020. Tinc P.J., Jenkins P., Sorensen J.A., Weinehall L., Gadomski A., Lindvall K.. Key factors for successful implementation of the national rollover protection structure rebate program: A correlation analysis using the consolidated framework for implementation research
281. 2020. Tinc P.J., Sorensen J.A., Lindvall K.. Stakeholder Experiences Implementing a National ROPS Rebate Program: A Grounded Theory Situational Analysis
282. 2020. Tong E.K., Wolf T., Cooke D.T., Fairman N., Chen M.S., Jr.. The emergence of a sustainable tobacco treatment program across the cancer care continuum: A systems approach for implementation at the university of california davis comprehensive cancer center
283. 2020. Tonkin E., Calzone K.A., Badzek L., Benjamin C., Middleton A., Patch C., Kirk M.. A Roadmap for Global Acceleration of Genomics Integration Across Nursing
284. 2019. Trumbo, Silas P; Iams, Wade T; Limper, Heather M; Goggins, Kathryn; Gibson, Jayme; Oliver, Lauren; Leverenz, David L; Samuels, Lauren R; Brady, Donald W; Kripalani, Sunil. Deimplementation of Routine Chest X-rays in Adult Intensive Care Units
285. 2019. Tucker, Sharon; Sheikholeslami, Deborah; Farrington, Michele; Picone, Debra; Johnson, Janis; Matthews, Grace; Evans, Rhonda; Gould, Renee; Bohlken, Deborah; Comried, Lynn; Petrulevich, Kelly; Perkhounkova, Elena; Cullen, Laura. Patient, Nurse, and Organizational Factors That Influence Evidence-Based Fall Prevention for Hospitalized Oncology Patients: An Exploratory Study
286. 2020. Turner D., Lockhart E., Wang W., Shore R., Daley E.M., Marhefka S.L.. PrEP Implementation Behaviors of Community-Based HIV Testing Staff: A Mixed-Methods Approach Using Latent Class Analysis
287. 2018. Valverde, P A; Calhoun, E; Esparza, A; Wells, K J; Risendal, B C. The early dissemination of patient navigation interventions: Results of a respondent-driven sample survey
288. 2018/2020. Vamos C.A., Green S.M., Griner S., Daley E., DeBate R., Jacobs T., Christiansen S.. Identifying Implementation Science Characteristics for a Prenatal Oral Health eHealth Application
289. 2016. Vamos, C A; Cantor, A; Thompson, E L; Detman, L A; Bronson, E A; Phelps, A; Louis, J M; Gregg, A R; Curran, J S; Sappenfield, W M. The Obstetric Hemorrhage Initiative (OHI) in Florida: The Role of Intervention Characteristics in Influencing Implementation Experiences among Multidisciplinary Hospital Staff
290. 2019. Vamos, C A; Griner, S B; Kirchharr, C; Green, S M; Debate, R; Daley, E M; Quinonez, R B; Boggess, K A; Jacobs, T; Christiansen, S. The development of a theory-based eHealth app prototype to promote oral health during prenatal care visits
291. 2019. van der Vaart, R; Worm-Smeitink, M; Bos, Y; Wensing, M; Evers, A; Knoop, H. Implementing guided ICBT for chronic pain and fatigue: A qualitative evaluation among therapists and managers
292. 2019. Van Rinsum, C; Gerards, S; Rutten, G; Johannesma, M; Van De Goor, I; Kremers, S. The implementation of the coaching on lifestyle (CooL) intervention: Lessons learnt
293. 2020. van Rooijen M., Lenzen S., Dalemans R., Moser A., Beurskens A.. Implementation of a Patient Reported Experience Measure in a Dutch disability care organisation: a qualitative study
294. 2016. Vanderlee, L; Vine, M M; Fenton, N E; Hammond, D. Stakeholder perspectives on implementing menu labeling in a cafeteria setting
295. 2017. VanDevanter, N; Kumar, P; Nguyen, N; Nguyen, L; Nguyen, T; Stillman, F; Weiner, B; Shelley, D. Application of the Consolidated Framework for Implementation Research to assess factors that may influence implementation of tobacco use treatment guidelines in the Viet Nam public health care delivery system
296. 2020. Varley, A L; Lappan, S; Jackson, J; Goodin, B R; Cherrington, A L; Copes, H; Hendricks, P S. Understanding Barriers and Facilitators to the Uptake of Best Practices for the Treatment of Co-Occurring Chronic Pain and Opioid Use Disorder
297. 2020. Vasudevan L., Schroeder K., Raveendran Y., Goel K., Makarushka C., Masalu N., Zullig L.L.. Using digital health to facilitate compliance with standardized pediatric cancer treatment guidelines in Tanzania: Protocol for an early-stage effectiveness-implementation hybrid study
298. 2020. Verjans-Janssen S.R.B., Gerards S.M.P.L., Verhees A.H., Kremers S.P.J., Vos S.B., Jansen M.W.J., Van Kann D.H.H.. Implementation of KEIGAAF in primary schools: A mutual adaptation physical activity and nutrition intervention
299. 2019. Wagner, Daniel J; Durbin, Janet; Barnsley, Jan; Ivers, Noah M. Measurement without management: qualitative evaluation of a voluntary audit & feedback intervention for primary care teams
300. 2019. Walker, Sarah Cusworth; Sedlar, Georganna; Berliner, Lucy; Rodriguez, Felix I; Davis, Paul A; Johnson, Savannah; Leith, Jessica. Advancing the state-level tracking of evidence-based practices: a case study
301. 2019. Walker, Timothy J; Rodriguez, Serena A; Vernon, Sally W; Savas, Lara S; Frost, Erica L; Fernandez, Maria E. Validity and reliability of measures to assess constructs from the inner setting domain of the consolidated framework for implementation research in a pediatric clinic network implementing HPV programs
302. 2020. Wallace B., Van Roode T., Pagan F., Phillips P., Wagner H., Calder S., Aasen J., Pauly B., Hore D.. What is needed for implementing drug checking services in the context of the overdose crisis? A qualitative study to explore perspectives of potential service users
303. 2019. Wallace, Bruce; Pagan, Flora; Pauly, Bernadette. The implementation of overdose prevention sites as a novel and nimble response during an illegal drug overdose public health emergency
304. 2019. Waltz, Thomas J; Powell, Byron J; Fernandez, Maria E; Abadie, Brenton; Damschroder, Laura J. Choosing implementation strategies to address contextual barriers: diversity in recommendations and future directions
305. 2017. Warren, C E; Ndwiga, C; Sripad, P; Medich, M; Njeru, A; Maranga, A; Odhiambo, G; Abuya, T. Sowing the seeds of transformative practice to actualize women's rights to respectful maternity care: Reflections from Kenya using the consolidated framework for implementation research
306. 2019. Weintraub, J A; Birken, S A; Burgette, J M; Lewis, T A; White, B A. Use of the consolidated framework for implementation research to assess determinants of silver diamine fluoride implementation in safety net dental clinics
307. 2020. Weir A., Kitto S., Smith J., Presseau J., Colman I., Hatcher S.. Barriers and enablers to conducting cluster randomized control trials in hospitals: A theory-informed scoping review
308. 2017. Weiss, C H. Why do we fail to deliver evidence-based practice in critical care medicine?
309. 2019. Werts, N; Acharya, S. A Framework for Enhancing Health Information Data Security: Application of the Consolidated Framework for Implementation Research to Breach Analysis
310. 2020. Westafer L.M., Kunz A., Bugajska P., Hughes A., Mazor K.M., Schoenfeld E.M., Stefan M.S., Lindenauer P.K.. Provider Perspectives on the Use of Evidence-based Risk Stratification Tools in the Evaluation of Pulmonary Embolism: A Qualitative Study
311. 2019. White, M C; Randall, K; Capo-Chichi, N F E; Sodogas, F; Quenum, S; Wright, K; Close, K L; Russ, S; Sevdalis, N; Leather, A J M. Implementation and evaluation of nationwide scale-up of the Surgical Safety Checklist
312. 2017. Williams, K M; Kirsh, S; Aron, D; Au, D; Helfrich, C; Lambert-Kerzner, A; Lowery, J; Battaglia, C; Graham, G D; Doukas, M; Jain, R; Ho, P M. Evaluation of the Veterans Health Administration's Specialty Care Transformational Initiatives to Promote Patient-Centered Delivery of Specialty Care: A Mixed-Methods Approach
313. 2018. Williams-Roberts, H; Neudorf, C; Abonyi, S; Cushon, J; Muhajarine, N. Facilitators and barriers of sociodemographic data collection in Canadian health care settings: A multisite case study evaluation
314. 2019. Witwer, E; Baldwin, L.-M.; Cole, A. Qualitative Assessment of Washington State Medicaid Health Plan Readiness to Implement Systems-Based Approaches to Colorectal Cancer Screening
315. 2015.Wolfenden, L; Finch, M; Nathan, N; Weaver, N; Wiggers, J; Yoong, S L; Jones, J; Dodds, P; Wyse, R; Sutherland, R; Gillham, K.Factors associated with early childhood education and care service implementation of healthy eating and physical activity policies and practices in Australia: a cross-sectional study
316. 2019. Zebrowski, Alexis M; Ellis, Darcy E; Barg, Frances K; Sperber, Nina R; Bernhardt, Barbara A; Denny, Joshua C; Dexter, Paul R; Ginsburg, Geoffrey S; Horowitz, Carol R; Johnson, Julie A; Levy, Mia A; Orlando, Lori A; Pollin, Toni I; Skaar, Todd C; Kimmel, Stephen E. Qualitative study of system-level factors related to genomic implementation
317. 2017. Zidarov, D; Poissant, L; Sicotte, C. Use of comparative performance indicators in rehabilitation
