# Supplementary material for: The updated Consolidated Framework for Implementation Research based on user feedback
Source: Implement Sci. 2022 Oct 29;17:75. doi: 10.1186/s13012-022-01245-0 (PMC9617234; doi:10.1186/s13012-022-01245-0)
Supplement: Supplementary file 4 — Additional file 4. Original CFIR (2009) to Updated CFIR (2022): Construct Mapping. [file 13012_2022_1245_MOESM4_ESM.docx]

# Additional File 4: Original CFIR (2009) to Updated CFIR (2022): Construct Mapping

This additional file maps the original CFIR (published in 2009) constructs to the updated CFIR (published in 2022) constructs.

| **Original CFIR**  *No specific guidance provided at the framework-level in the original CFIR* | | **Updated CFIR**  ***Framework Guidance:***  The CFIR is intended to be used to collect data from individuals who have power and/or influence over implementation outcomes. See the CFIR Outcomes Addendum for guidance on identifying these individuals and selecting outcomes [1].  The CFIR must be fully operationalized prior to use in a project:  1) Define the subject of each domain for the project (see guidance for each domain below).  2) Replace broad construct language with project-specific language if needed.  3) Add constructs to capture salient themes not included in the updated CFIR. | |
| --- | --- | --- | --- |
| **I. INTERVENTION CHARACTERISTICS DOMAIN**  *No specific guidance provided at the domain-level in the original CFIR.* | | **I. INNOVATION DOMAIN**  ***Innovation:*** The “thing” being implemented [2], e.g., a new clinical treatment, educational program, or city service.  ***Project Innovation***: [Document the innovation being implemented, e.g., innovation type, innovation core vs. adaptable components, using a published reporting guideline [3–6]. Distinguish the innovation (the “thing” that continues when implementation is complete) [2,7] from the implementation process and strategies used to implement the innovation [8,9] (activities that end after implementation is complete) [10].] | |
| **Old Construct Name** | **Old Construct Definition** | **Construct Name** | **Construct Definition** *The degree to which:* |
| Intervention Source | Perception of key stakeholders about whether the intervention is externally or internally developed. | A. Innovation Source | The group that developed and/or visibly sponsored use of the innovation is reputable, credible, and/or trustable. |
| Evidence Strength & Quality | Stakeholders’ perceptions of the quality and validity of evidence supporting the belief that the intervention will have desired outcomes. | B. Innovation Evidence-Base | The innovation has robust evidence supporting its effectiveness. |
| Relative Advantage | Stakeholders’ perception of the advantage of implementing the intervention versus an alternative solution. | C. Innovation Relative Advantage | The innovation is better than other available innovations or current practice. |
| Adaptability | The degree to which an intervention can be adapted, tailored, refined, or reinvented to meet local needs. | D. Innovation Adaptability | The innovation can be modified, tailored, or refined to fit local context or needs. |
| Trialability | The ability to test the intervention on a small scale in the organization, and to be able to reverse course (undo implementation) if warranted. | E. Innovation Trialability | The innovation can be tested or piloted on a small scale and undone. |
| Complexity | Perceived difficulty of implementation, reflected by duration, scope, radicalness, disruptiveness, centrality, and intricacy and number of steps required to implement. | F. Innovation Complexity | The innovation is complicated, which may be reflected by its scope and/or the nature and number of connections and steps. |
| Design Quality and Packaging | Perceived excellence in how the intervention is bundled, presented, and assembled. | G. Innovation Design | The innovation is well designed and packaged, including how it is assembled, bundled, and presented. |
| Cost | Costs of the intervention and costs associated with implementing that intervention including investment, supply, and opportunity costs. | H. Innovation Cost | The innovation purchase and operating costs are affordable. |
| **II. OUTER SETTING DOMAIN**  *No specific guidance provided at the domain-level in the original CFIR.* | | **II. OUTER SETTING DOMAIN**  ***Outer Setting:*** The setting in which the Inner Setting exists, e.g., hospital system, school district, state. There may be multiple Outer Settings and/or multiple levels within the Outer Setting (e.g., community, system, state).   ***Project Outer Setting(s):*** [Document the actual Outer Setting in the project, e.g., type, location, and the boundary between the Outer Setting and the Inner Setting.] | |
| **Old Construct Name** | **Old Construct Definition** | **Construct Name** | **Construct Definition** *The degree to which:* |
| Patient Needs & Resources | The extent to which patient needs, as well as barriers and facilitators to meet those needs, are accurately known and prioritized by the organization. | *None* | *Construct separated and relocated; see Roles Subdomain: Innovation Recipients; Characteristics Subdomain: Need; and Inner Setting Domain: Culture: Recipient-Centeredness.* |
| *None* | *Construct added in the updated CFIR.* | A. Critical Incidents | Large-scale and/or unanticipated events disrupt implementation and/or delivery of the innovation. |
| *None* | *Construct added in the updated CFIR.* | B. Local Attitudes | Sociocultural values (e.g., shared responsibility in helping recipients) and beliefs (e.g., convictions about the worthiness of recipients) encourage the Outer Setting to support implementation and/or delivery of the innovation. |
| *None* | *Construct added in the updated CFIR.* | C. Local Conditions | Economic, environmental, political, and/or technological conditions enable the Outer Setting to support implementation and/or delivery of the innovation. |
| Cosmopolitanism | The degree to which an organization is networked with other external organizations. | D. Partnerships & Connections | The Inner Setting is networked with external entities, including referral networks, academic affiliations, and professional organization networks. |
| External Policies & Incentives | A broad construct that includes external strategies to spread interventions including policy and regulations (governmental or other central entity), external mandates, recommendations and guidelines, pay-for-performance, collaboratives, and public or benchmark reporting. | E. Policies & Laws | Legislation, regulations, professional group guidelines and recommendations, or accreditation standards support implementation and/or delivery of the innovation. |
| *None* | *Construct added in the updated CFIR.* | F. Financing | Funding from external entities (e.g., grants, reimbursement) is available to implement and/or deliver the innovation. |
| *None* | *Construct added in the updated CFIR.* | G. External Pressure | External pressures drive implementation and/or delivery of the innovation.  *Use this construct to capture themes related to External Pressures that are not included in the subconstructs below.* |
| *None* | *Subconstruct added in the updated CFIR.* | 1. Societal Pressure | Mass media campaigns, advocacy groups, or social movements or protests drive implementation and/or delivery of the innovation. |
| Peer Pressure | Mimetic or competitive pressure to implement an intervention; typically, because most or other key peer or competing organizations have already implemented or in a bid for a competitive edge. | 2. Market Pressure | Competing with and/or imitating peer entities drives implementation and/or delivery of the innovation. |
| *None* | *See Outer Setting: External Policies & Incentives construct.* | 3. Performance-Measurement Pressure | Quality or benchmarking metrics or established service goals drive implementation and/or delivery of the innovation. |
| **III. INNER SETTING DOMAIN**  *No specific guidance provided at the domain-level in the original CFIR.* | | **III. INNER SETTING DOMAIN**  ***Inner Setting:*** The setting in which the innovation is implemented, e.g., hospital, school, city. There may be multiple Inner Settings and/or multiple levels within the Inner Setting, e.g., unit, classroom, team.   ***Project Inner Setting(s):*** [Document the actual Inner Setting in the project, e.g., type, location, and the boundary between the Outer Setting and the Inner Setting.] | |
| **Old Construct Name** | **Old Construct Definition** | **Construct Name** | **Construct Definition** *The degree to which:* |
| *None* | *No specific guidance provided at the domain-level in the original CFIR.* | *Note:* | *Constructs A – D exist in the Inner Setting regardless of implementation and/or delivery of the innovation, i.e., they are persistent general characteristics of the Inner Setting.* |
| Structural Characteristics | The social architecture, age, maturity, and size of an organization. | A. Structural Characteristics | Infrastructure components support functional performance of the Inner Setting.  *Use this construct to capture themes related to Structural Characteristics that are not included in the subconstructs below.* |
| *None* | *Subconstruct added in the updated CFIR.* | 1. Physical Infrastructure | Layout and configuration of space and other tangible material features support functional performance of the Inner Setting. |
| *None* | *Subconstruct added in the updated CFIR.* | 2. Information Technology Infrastructure | Technological systems for tele-communication, electronic documentation, and data storage, management, reporting, and analysis support functional performance of the Inner Setting. |
| *None* | *Subconstruct added in the updated CFIR.* | 3. Work Infrastructure | Organization of tasks and responsibilities within and between individuals and teams, and general staffing levels, support functional performance of the Inner Setting. |
| Networks & Communications | The nature and quality of webs of social networks and the nature and quality of formal and informal communications within an organization. | B. Relational Connections | There are high quality formal and informal relationships, networks, and teams within and across Inner Setting boundaries (e.g., structural, professional). |
|  |  | C. Communications | There are high quality formal and informal information sharing practices within and across Inner Setting boundaries (e.g., structural, professional). |
| Culture | Norms, values, and basic assumptions of a given organization. | D. Culture | There are shared values, beliefs, and norms across the Inner Setting.  *Use this construct to capture themes related to Culture that are not included in the subconstructs below.* |
| *None* | *Subconstruct added in the updated CFIR.* | 1. Human Equality-Centeredness | There are shared values, beliefs, and norms about the inherent equal worth and value of all human beings. |
| *None* | *Subconstruct added in the updated CFIR.* | 2. Recipient-Centeredness | There are shared values, beliefs, and norms around caring, supporting, and addressing the needs and welfare of recipients. |
| *None* | *Subconstruct added in the updated CFIR.* | 3. Deliverer-Centeredness | There are shared values, beliefs, and norms around caring, supporting, and addressing the needs and welfare of deliverers. |
| *None* | *See Inner Setting: Learning Climate construct.* | 4. Learning-Centeredness | There are shared values, beliefs, and norms around psychological safety, continual improvement, and using data to inform practice. |
| *None* | *No specific guidance provided at the domain-level in the original CFIR.* | *Note:* | *Constructs E – K are specific to the implementation and/or delivery of the innovation****.*** |
| Implementation Climate | The absorptive capacity for change, shared receptivity of involved individuals to an intervention and the extent to which use of that intervention will be rewarded, supported, and expected within their organization. | *None* | *Construct removed from the updated CFIR; reclassified as an antecedent assessment in the CFIR Outcomes Addendum* [1]*.* |
| Tension for Change | The degree to which stakeholders perceive the current situation as intolerable or needing change. | E. Tension for Change | The current situation is intolerable and needs to change. |
| Compatibility | The degree of tangible fit between meaning and values attached to the intervention by involved individuals, how those align with individuals’ own norms, values, and perceived risks and needs, and how the intervention fits with existing workflows and systems. | F. Compatibility | The innovation fits with workflows, systems, and processes. |
| Relative Priority | Individuals’ shared perception of the importance of the implementation within the organization. | G. Relative Priority | Implementing and delivering the innovation is important compared to other initiatives. |
| Organizational Incentives & Rewards | Extrinsic incentives such as goal-sharing awards, performance reviews, promotions, and raises in salary and less tangible incentives such as increased stature or respect. | H. Incentive Systems | Tangible and/or intangible incentives and rewards and/or disincentives and punishments support implementation and delivery of the innovation. |
| Goals & Feedback | The degree to which goals are clearly communicated, acted upon, and fed back to staff, and alignment of that feedback with goals. | I. Mission Alignment | Implementing and delivering the innovation is in line with the overarching commitment, purpose, or goals in the Inner Setting. |
| Learning Climate | A climate in which: a) leaders express their own fallibility and need for team members’ assistance and input; b) team members feel that they are essential, valued, and knowledgeable partners in the change process; c) individuals feel psychologically safe to try new methods; and d) there is sufficient time and space for reflective thinking and evaluation. | *None* | *Construct renamed and relocated; see Inner Setting: Culture: Learning-Centeredness.* |
| Readiness for Implementation | Tangible and immediate indicators of organizational commitment to its decision to implement an intervention. | *None* | *Construct removed from the updated CFIR; reclassified as an antecedent assessment in the CFIR Outcomes Addendum* [1]*.* |
| Leadership Engagement | Commitment, involvement, and accountability of leaders and managers with the implementation. | *None* | *Construct separated, renamed, and relocated; see Individuals Domain: Roles Subdomain: High-Level & Mid-Level Leaders; and Characteristics Subdomain: Motivation.* |
| Available Resources | The level of resources dedicated for implementation and on-going operations including money, training, education, physical space, and time. | J. Available Resources | Resources are available to implement and deliver the innovation.  *Use this construct to capture themes related to Available Resources that are not included in the subconstructs below.* |
| *None* | *Subconstruct added in the updated CFIR.* | 1. Funding | Funding is available to implement and deliver the innovation. |
| *None* | *Subconstruct added in the updated CFIR.* | 2. Space | Physical space is available to implement and deliver the innovation. |
| *None* | *Subconstruct added in the updated CFIR.* | 3. Materials & Equipment | Supplies are available to implement and deliver the innovation. |
| Access to knowledge and information | Ease of access to digestible information and knowledge about the intervention and how to incorporate it into work tasks. | K. Access to Knowledge & Information | Guidance and/or training is accessible to implement and deliver the innovation. |
| **IV. CHARACTERISTICS OF INDIVIDUALS**  *No specific guidance provided at the domain-level in the original CFIR.* | | **IV. INDIVIDUALS DOMAIN**  ***Individuals:*** The roles and characteristics of individuals. | |
| *None: Roles Subdomain added in the updated CFIR.* | | **ROLES SUBDOMAIN**  ***Project Roles:*** [Document the roles applicable to the project and their location in the Inner or Outer Setting.] | |
| **Old Construct Name** | **Old Construct Definition** | **Construct Name** | **Construct Definition** |
| *None* | *See Inner Setting: Leadership Engagement.* | A. High-level Leaders | Individuals with a high level of authority, including key decision-makers, executive leaders, or directors. |
| *None* | *See Inner Setting: Leadership Engagement* | B. Mid-level Leaders | Individuals with a moderate level of authority, including leaders supervised by a high-level leader and who supervise others. |
| *None* | *See Process: Engaging: Opinion Leaders.* | C. Opinion Leaders | Individuals with informal influence on the attitudes and behaviors of others. |
| *None* | *See Process: Engaging: External Change Agents.* | D. Implementation Facilitators | Individuals with subject matter expertise who assist, coach, or support implementation. |
| *None* | *See Process: Engaging: Formally Appointed Internal Implementation Leaders & Champions.* | E. Implementation Leads | Individuals who lead efforts to implement the innovation. |
| *None* | *Construct added in the updated CFIR.* | F. Implementation Team Members | Individuals who collaborate with and support the Implementation Leads to implement the innovation, ideally including Innovation Deliverers and Recipients. |
| *None* | *Construct added in the updated CFIR.* | G. Other Implementation Support | Individuals who support the Implementation Leads and/or Implementation Team Members to implement the innovation. |
| *None* | *Construct added in the updated CFIR.* | H. Innovation Deliverers | Individuals who are directly or indirectly delivering the innovation. |
| *None* | *See Outer Setting: Patient Needs & Resources.* | I. Innovation Recipients | Individuals who are directly or indirectly receiving the innovation. |
| *None: Characteristics Subdomain added in the updated CFIR.* | | **CHARACTERISTICS SUBDOMAIN**  ***Project Characteristics:*** [Document the characteristics applicable to the roles in the project based on the COM-B system [11] or role-specific theories.] | |
| **Old Construct Name** | **Old Construct Definition** | **Construct Name** | **Construct Definition:**  *The degree to which:* |
| Knowledge & Beliefs about the Intervention | Individuals’ attitudes toward and value placed on the intervention as well as familiarity with facts, truths, and principles related to the intervention. | *None* | *Construct removed from the updated CFIR.* |
| Self-efficacy | Individual belief in their own capabilities to execute courses of action to achieve implementation goals. | *None* | *Construct removed from the updated CFIR.* |
| Individual Stage of Change | Characterization of the phase an individual is in, as he or she progresses toward skilled, enthusiastic, and sustained use of the intervention. | *None* | *Construct removed from the updated CFIR.* |
| Individual Identification with Organization | A broad construct related to how individuals perceive the organization and their relationship and degree of commitment with that organization. | *None* | *Construct removed from the updated CFIR.* |
| Other Personal Attributes | A broad construct to include other personal traits such as tolerance of ambiguity, intellectual ability, motivation, values, competence, capacity, and learning style. | *None* | *Construct removed from the updated CFIR.* |
| *None* | *Construct added in the updated CFIR.* | A. Need | The individual(s) has deficits related to survival, well-being, or personal fulfillment, which will be addressed by implementation and/or delivery of the innovation. |
| *None* | *Construct added in the updated CFIR.* | B. Capability | The individual(s) has interpersonal competence, knowledge, and skills to fulfill Role. |
| *None* | *Construct added in the updated CFIR.* | C. Opportunity | The individual(s) has availability, scope, and power to fulfill Role. |
| *None* | *Construct added in the updated CFIR.* | D. Motivation | The individual(s) is committed to fulfilling Role. |
| **V. PROCESS**  *No specific guidance provided at the domain-level in the original CFIR.* | | **V. IMPLEMENTATION PROCESS DOMAIN**  ***Implementation Process:*** The activities and strategies used to implement the innovation.  ***Project Implementation Process:*** [Document the implementation process framework [12] and/or activities and strategies [8,9] being used to implement the innovation. Distinguish the implementation process used to implement the innovation (activities that end after implementation is complete) from the innovation (the “thing” that continues when implementation is complete) [2,7,10].] | |
| **Old Construct Name** | **Old Construct Definition** | **Construct Name** | **Construct Definition:** *The degree to which individuals:* |
| *None* | *Construct added in the updated CFIR.* | A. Teaming | Join together, intentionally coordinating and collaborating on interdependent tasks, to implement the innovation. |
| *None* | *Construct added in the updated CFIR.* | B. Assessing Needs | Collect information about priorities, preferences, and needs of people.  *Use this construct to capture themes related to Assessing Needs that are not included in the subconstructs below.* |
| *None* | *Subconstruct added in the updated CFIR.* | 1. Innovation Deliverers | Collect information about the priorities, preferences, and needs of deliverers to guide implementation and delivery of the innovation. |
| *None* | *Subconstruct added in the updated CFIR.* | 2. Innovation Recipients | Collect information about the priorities, preferences, and needs of recipients to guide implementation and delivery of the innovation. |
| *None* | *Construct added in the updated CFIR.* | C. Assessing Context | Collect information to identify and appraise barriers and facilitators to implementation and delivery of the innovation. |
| Planning | The degree to which a scheme or method of behavior and tasks for implementing an intervention are developed in advance and the quality of those schemes or methods. | D. Planning | Identify roles and responsibilities, outline specific steps and milestones, and define goals and measures for implementation success in advance. |
| *None* | *Construct added in the updated CFIR.* | E. Tailoring Strategies | Choose and operationalize implementation strategies to address barriers, leverage facilitators, and fit context. |
| Engaging | Attracting and involving appropriate individuals in the implementation and use of the intervention through a combined strategy of social marketing, education, role modeling, training, and other similar activities. | F. Engaging | Attract and encourage participation in implementation and/or the innovation.  *Use this construct to capture themes related to Engaging that are not included in the subconstructs below.* |
| *None* | *Subconstruct added in the updated CFIR.* | 1. Innovation Deliverers | Attract and encourage deliverers to serve on the implementation team and/or to deliver the innovation. |
| *None* | *Subconstruct added in the updated CFIR.* | 2. Innovation Recipients | Attract and encourage recipients to serve on the implementation team and/or participate in the innovation. |
| Opinion Leaders | Individuals in an organization who have formal or informal influence on the attitudes and beliefs of their colleagues with respect to implementing the intervention. | *None* | *Subconstruct relocated; see Individuals Domain: Roles Subdomain: Opinion Leaders.* |
| Formally appointed internal implementation leaders | Individuals from within the organization who have been formally appointed with responsibility for implementing an intervention as coordinator, project manager, team leader, or other similar role. | *None* | *Subconstructs combined, renamed, and relocated; see Individuals Domain: Roles Subdomain: Implementation Leads.* |
| Champions | “Individuals who dedicate themselves to supporting, marketing, and ‘driving through’ an [implementation]”, overcoming indifference or resistance that the intervention may provoke in an organization. |  |  |
| External Change Agents | Individuals who are affiliated with an outside entity who formally influence or facilitate intervention decisions in a desirable direction. | *None* | *Subconstruct renamed and relocated; see Individuals Domain: Roles Subdomain: Implementation Facilitators.* |
| Executing | Carrying out or accomplishing the implementation according to plan. | G. Doing | Implement in small steps, tests, or cycles of change to trial and cumulatively optimize delivery of the innovation. |
| Reflecting & Evaluating | Quantitative and qualitative feedback about the progress and quality of implementation accompanied with regular personal and team debriefing about progress and experience. | H. Reflecting & Evaluating | Collect and discuss quantitative and qualitative information about the success of implementation and/or the innovation.  *Use this construct to capture themes related to Reflecting & Evaluating that are not included in the subconstructs below.* |
| *None* | *Subconstruct added in the updated CFIR.* | 1. Implementation | Collect and discuss quantitative and qualitive information about the success of implementation. |
| *None* | *Subconstruct added in the updated CFIR.* | 2. Innovation | Collect and discuss quantitative and qualitative information about the success of the innovation. |
| *None* | *Construct added in the updated CFIR.* | I. Adapting | Modify the innovation and/or the Inner Setting for optimal fit and integration into work processes. |

# References

[1] Damschroder LJ, Reardon CM, Opra Widerquist MA, Lowery J. Conceptualizing outcomes for use with the Consolidated Framework for Implementation Research (CFIR): the CFIR Outcomes Addendum. Implementation Sci 2022;17:7. https://doi.org/10.1186/s13012-021-01181-5.

[2] Curran GM. Implementation science made too simple: a teaching tool. Implement Sci Commun 2020;1:27. https://doi.org/10.1186/s43058-020-00001-z.

[3] Albrecht L, Archibald M, Arseneau D, Scott SD. Development of a checklist to assess the quality of reporting of knowledge translation interventions using the Workgroup for Intervention Development and Evaluation Research (WIDER) recommendations. Implementation Sci 2013;8:52. https://doi.org/10.1186/1748-5908-8-52.

[4] Butler M, Epstein RA, Totten A, Whitlock EP, Ansari MT, Damschroder LJ, et al. AHRQ series on complex intervention systematic reviews—paper 3: adapting frameworks to develop protocols. Journal of Clinical Epidemiology 2017;90:19–27. https://doi.org/10.1016/j.jclinepi.2017.06.013.

[5] The AIMD Writing/Working Group, Bragge P, Grimshaw JM, Lokker C, Colquhoun H. AIMD - a validated, simplified framework of interventions to promote and integrate evidence into health practices, systems, and policies. BMC Med Res Methodol 2017;17:38. https://doi.org/10.1186/s12874-017-0314-8.

[6] Hoffmann TC, Glasziou PP, Boutron I, Milne R, Perera R, Moher D, et al. Better reporting of interventions: template for intervention description and replication (TIDieR) checklist and guide. BMJ 2014;348:g1687. https://doi.org/10.1136/bmj.g1687.

[7] Lengnick-Hall R, Gerke DR, Proctor EK, Bunger AC, Phillips RJ, Martin JK, et al. Six practical recommendations for improved implementation outcomes reporting. Implementation Sci 2022;17:16. https://doi.org/10.1186/s13012-021-01183-3.

[8] Powell BJ, McMillen JC, Proctor EK, Carpenter CR, Griffey RT, Bunger AC, et al. A compilation of strategies for implementing clinical innovations in health and mental health. Med Care Res Rev 2012;69:123–57. https://doi.org/10.1177/1077558711430690.

[9] Powell BJ, Waltz TJ, Chinman MJ, Damschroder LJ, Smith JL, Matthieu MM, et al. A refined compilation of implementation strategies: results from the Expert Recommendations for Implementing Change (ERIC) project. Implementation Science 2015;10:21.

[10] Pinnock H, Barwick M, Carpenter CR, Eldridge S, Grandes G, Griffiths CJ, et al. Standards for Reporting Implementation Studies (StaRI) Statement. BMJ 2017:i6795. https://doi.org/10.1136/bmj.i6795.

[11] Michie S, van Stralen MM, West R. The behaviour change wheel: A new method for characterising and designing behaviour change interventions. Implement Sci 2011;6:42. https://doi.org/10.1186/1748-5908-6-42.

[12] Nilsen P. Making sense of implementation theories, models and frameworks. Implementation Science 2015;10:53.
