# Supplementary material for: The updated Consolidated Framework for Implementation Research based on user feedback
Source: Implement Sci. 2022 Oct 29;17:75. doi: 10.1186/s13012-022-01245-0 (PMC9617234; doi:10.1186/s13012-022-01245-0)
Supplement: Supplementary file 5 — Additional file 5. User Feedback & CFIR Updates. [file 13012_2022_1245_MOESM5_ESM.docx]

# Additional File 5: User Feedback & CFIR Updates

This additional file maps the original CFIR (published in 2009) to the updated CFIR (published in 2022) and provides a rationale from user feedback for each update made in the framework. Updates were made at three levels in the CFIR: 1) the framework level, i.e., changes to the CFIR overall; 2) the domain level, i.e., changes to the domain overall; and 3) the construct level, i.e., changes to the specific construct. The types of changes made include:

Addition of guidance at the framework-level and domain-level

Minor revisions to domain/construct names and definitions to clarify information from the original CFIR

Major revisions to domain/construct names and definitions to correct inconsistencies from the original CFIR

Removal of existing constructs/subconstructs

Addition of new constructs/subconstructs

Reorganization of constructs and domains, including relocating constructs, separating single constructs into multiple constructs, and combining multiple constructs into single constructs

# Framework

## Framework-Level User Feedback & CFIR Updates

| **Original CFIR** | *No specific guidance provided at the framework-level in the original CFIR.* | **Updated CFIR** | ***Framework Guidance:***  The CFIR is intended to be used to collect data from individuals who have power and/or influence over implementation outcomes. See the CFIR Outcomes Addendum for guidance on identifying these individuals and selecting outcomes [1].  The CFIR must be fully operationalized prior to use in a project:  1) Define the subject of each domain for the project (see guidance for each domain below).  2) Replace broad construct language with project-specific language if needed.  3) Add constructs to capture salient themes not included in the updated CFIR. |
| --- | --- | --- | --- |
| ***User Feedback***  Users were unclear if CFIR constructs were intended to capture perceptions or reality; one stated: “A difficult distinction here is whether these are PERCEPTIONS of the implementer, or actual features of the program; both seem important, and we have tried to capture both, but this can get confusing” (survey response).  ***Addition of Framework-Level Guidance***  As a result, we added guidance in the updated CFIR to clarify that the perceptions of those with influence and/or power are important determinants of implementation outcomes; responses to questions related to CFIR constructs will likely reflect a blend of objective reality and perceptions that arise out of experiences within the setting. However, we also added an area in each domain for users to document an a priori factual definition of the subject of each domain.  ***User Feedback***  Users felt the CFIR was too narrow; they felt it was hard to apply beyond healthcare settings and/or with non-clinical innovations, e.g., dynamic technological innovations [2,3], consultative innovations [4], or innovations that contained multiple steps [5,6] or spanned multiple settings [7]. In addition, they felt the language in the CFIR should be simpler and more consistent across constructs.  ***Minor Revisions to Construct Names & Definitions***  As a result, we made several changes to clarify language throughout the CFIR. We updated domain/construct names and definitions to broaden applicability of the updated CFIR (e.g., replacing the term patient with recipient) and revised verbiage to be simpler and more consistent across the framework. *Note: Given that all constructs contain minor revisions to make language broader, simpler, and/or more consistent, these types of updates are not explicitly detailed below.* In addition, we encourage users to operationalize the CFIR for their project, which may include adopting language more suitable to the project. | | | |

# Innovation

## Domain-Level User Feedback & CFIR Updates

| **I. INTERVENTION CHARACTERISTICS DOMAIN** | *No specific guidance provided at the domain-level in the original CFIR.* | **I. INNOVATION DOMAIN** | ***Innovation:*** The “thing” being implemented [8], e.g., a new clinical treatment, educational program, or city service.  ***Project Innovation:*** [Document the innovation being implemented, e.g., innovation type, innovation core vs. adaptable components, using a published reporting guideline [9–12]. Distinguish the innovation (the “thing” that continues when implementation is complete) [8,13] from the implementation process and strategies used to implement the innovation [14,15] (activities that end after implementation is complete) [16].] |
| --- | --- | --- | --- |
| ***User Feedback***  Users were unclear if the CFIR was intended to evaluate the innovation and/or the strategy being used to implement the innovation. While the CFIR is most often used to evaluate the implementation of innovations, it can be used to evaluate an implementation strategy if that *is* the innovation being evaluated.  ***Addition of Domain-Level Guidance***  Users must determine the goal of the evaluation and define the innovation and implementation strategy appropriately; distinguishing between the innovation and implementation strategy is necessary for accurate attribution to implementation outcomes [16] and to identify appropriate areas for future intervention, e.g., did implementation fail due to negative perceptions of the innovation *itself* or of the implementation strategy being used to implement the innovation? As a result, domain-level guidance was added to advise users to document the innovation being evaluated. In addition, the word “Innovation” was added to each construct name to further orient users to the focus of this domain. | | | |

## Construct-Level User Feedback & CFIR Updates

| **Old Construct Name** | **Old Construct Definition** | **Construct Name** | **Construct Definition** *The degree to which:* |
| --- | --- | --- | --- |
| Intervention Source | Perception of key stakeholders about whether the intervention is externally or internally developed. | A. Innovation Source | The group that developed and/or visibly sponsored use of the innovation is reputable, credible, and/or trustable. |
| ***User Feedback***  Users cited challenges using the Intervention Source construct because the detailed description implied that internal sources may facilitate implementation while external sources may hinder implementation (see Additional File 4 from the 2009 publication) [17]. They did not feel this was the case in all contexts, e.g., the Inner Setting may lack the necessary “experience for developing an intervention” (survey response) and in “resource-poor settings” an external source may “demonstrate the intervention value and increase likelihood of future local investment” [18].  ***Major Revision of Construct Definition***  The original CFIR detailed description of this construct also highlighted the “legitimacy of the source” as an important implementation determinant [17]. As a result, the construct definition was updated to focus on the trustworthiness of the source regardless of its location. | | | |
| Evidence Strength & Quality | Stakeholders’ perceptions of the quality and validity of evidence supporting the belief that the intervention will have desired outcomes. | B. Innovation Evidence-Base | The innovation has robust evidence supporting its effectiveness. |
| ***User Feedback***  Users were unclear about the difference between the Evidence Strength & Quality construct and innovation outcomes. For example, one user used this construct to code statements about the innovation working for their patients while another renamed this construct “effectiveness” (survey response).  ***Minor Revision of Construct Name***  Both uses above relate to effectiveness (an innovation outcome) instead of the underlying evidence-base (an implementation determinant). As a result, the construct was renamed to explicitly refer to the existing evidence-*base.* | | | |
| Relative Advantage | Stakeholders’ perception of the advantage of implementing the intervention versus an alternative solution. | C. Innovation Relative Advantage | The innovation is better than other available innovations or current practice. |
| ***User Feedback***  Users questioned if the Relative Advantage construct also referred to the advantage of the innovation compared to the status quo.  ***Minor Revision of Definition***  Comparing the innovation to the status quo is consistent with the original definition of this construct, which compares the innovation with the “idea it supersedes” [19]. As a result, the construct definition was updated to include comparison to the status quo. | | | |
| Adaptability | The degree to which an intervention can be adapted, tailored, refined, or reinvented to meet local needs. | D. Innovation Adaptability | The innovation can be modified, tailored, or refined to fit local context or needs. |
| ***User Feedback***  Users were unclear if the Adaptability construct was intended to capture the inherent adaptability of the innovation or the process of adapting the innovation, both of which are important implementation determinants. In addition, users questioned how to use this construct when the core vs. adaptable components of the innovation were not defined.  ***Addition of Domain-Level Guidance & Addition of New Adapting Construct*** *(see Implementation Process Domain)*  This construct refers to the inherent adaptability of the innovation; as a result, we added a new construct to the Implementation Process Domain (Adapting) to capture the process of adapting the innovation or Inner Setting as needed. In addition, in the domain-level guidance, users are strongly encouraged to define the innovation being implemented, including the core components and adaptable periphery of the innovation. | | | |
| Trialability | The ability to test the intervention on a small scale in the organization, and to be able to reverse course (undo implementation) if warranted. | E. Innovation Trialability | The innovation can be tested or piloted on a small scale and undone. |
| ***User Feedback***  Users were unclear if the Trialability construct was intended to capture the inherent trialability of the innovation or the process of trialing the innovation, both of which are important implementation determinants.  ***Minor Revision of Executing Construct Name and Definition*** *(see Implementation Process Domain)*  This construct refers to the inherent trialability of the innovation; as a result, the process of trialing has been added to the Doing construct (previously named Executing) in the Implementation Process domain. | | | |
| Complexity | Perceived difficulty of implementation, reflected by duration, scope, radicalness, disruptiveness, centrality, and intricacy and number of steps required to implement. | F. Innovation Complexity | The innovation is complicated, which may be reflected by its scope and/or the nature and number of connections and steps. |
| ***User Feedback***  Users were unclear if the Complexity construct was intended to assess innovation or implementation complexity. In addition, users noted the overlap of this construct with the Inner Setting: Compatibility construct due to the inclusion of “radicalness” and “disruptiveness” in the definition.  ***Major Revision of Construct Definition***  While complexity of the innovation is an important implementation determinant, complexity of implementation is a function of any number of CFIR constructs and is closely related to the concept of “Implementability,” which is included as an Anticipated Implementation Outcome in the CFIR Outcomes Addendum [1]. As a result, the definition was updated to focus on facets of the innovation itself, not the implementation process, and without regard to how it interfaces with the Inner Setting. This definition is aligned with other published conceptualizations of complex innovations [10,20,21]. | | | |
| Design Quality and Packaging | Perceived excellence in how the intervention is bundled, presented, and assembled. | G. Innovation Design | The innovation is well designed and packaged, including how it is assembled, bundled, and presented. |
| *No additional changes (See Framework-Level User Feedback & CFIR updates above).* | | | |
| Cost | Costs of the intervention and costs associated with implementing that intervention including investment, supply, and opportunity costs. | H. Innovation Cost | The innovation purchase and operating costs are affordable. |
| ***User Feedback***  Users were unclear if the Cost construct was intended to assess innovation and/or implementation cost.  ***Major Revision of Construct Definition***  As a result, we have updated the definition to only include innovation cost. | | | |

# Outer Setting

## Domain-Level User Feedback & CFIR Updates

| **II. OUTER SETTING DOMAIN** | *No specific guidance provided at the domain-level in the original CFIR.* | **II. OUTER SETTING DOMAIN** | ***Outer Setting:*** The setting in which the Inner Setting exists, e.g., hospital system, school district, state. There may be multiple Outer Settings and/or multiple levels within the Outer Setting (e.g., community, system, state).   ***Project Outer Setting(s):*** [Document the actual Outer Setting in the project, e.g., type, location, and the boundary between the Outer Setting and the Inner Setting.] |
| --- | --- | --- | --- |
| ***User Feedback***  Users found it difficult to operationalize the Inner vs. Outer Setting in their projects; they found the domain labels “unintuitive” and gave diverse recommendations from combining them into a single domain (because it was challenging to define a boundary between them) to separating each of them into multiple levels, e.g., to account for multiple spheres of influence (national, regional, local) within the Outer Setting.  Users also felt this domain needed to be expanded, with one commenting that it “would be great to see this domain developed further” and another that it “needs the most improvement. It is the most under-developed in terms of the framework” (survey response).  ***Addition of Domain-Level Guidance and Addition of New Constructs***  As a result, domain-level guidance was added to help users delineate and define the Outer Setting for their project and several new constructs were added (see below). While it can be challenging to differentiate the Inner and Outer Settings, it is nonetheless important to define and delineate the boundary between the two domains for accurate attribution to implementation outcomes and to identify appropriate levels of future intervention. | | | |

## Construct-Level User Feedback & CFIR Updates

| **Old Construct Name** | **Old Construct Definition** | **Construct Name** | **Construct Definition** *The degree to which:* |
| --- | --- | --- | --- |
| Patient Needs & Resources | The extent to which patient needs, as well as barriers and facilitators to meet those needs, are accurately known and prioritized by the organization. | *None* | *Construct separated and relocated; see Roles Subdomain: Innovation Recipients; Characteristics Subdomain: Need; and Inner Setting Domain: Culture: Recipient-Centeredness.* |
| **Construct Separated & Relocated**  See Roles Subdomain: Innovation Recipients; Characteristics Subdomain: Need; and Inner Setting Domain: Culture: Recipient-Centeredness. | | | |
| *None* | *Construct added in the updated CFIR.* | A. Critical Incidents | Large-scale and/or unanticipated events disrupt implementation and/or delivery of the innovation. |
| ***User Feedback***  Users suggested the addition of a construct to cover unanticipated events; work conducted during the COVID 19 pandemic has highlighted the significant effects that such incidents can have on implementation efforts and their success.  ***Addition of New Construct***  As a result, this construct was added. | | | |
| *None* | *Construct added in the updated CFIR.* | B. Local Attitudes | Sociocultural values (e.g., shared responsibility in helping recipients) and beliefs (e.g., convictions about the worthiness of recipients) encourage the Outer Setting to support implementation and/or delivery of the innovation. |
| *None* | *Construct added in the updated CFIR.* | C. Local Conditions | Economic, environmental, political, and/or technological conditions enable the Outer Setting to support implementation and/or delivery of the innovation. |
| ***User Feedback***  Users expressed a need for additional constructs related to the local Outer Setting, e.g., the local community, including characteristics of the community that encourage and/or enable the Outer Setting to support implementation and/or delivery of the innovation [22,23]. While not all innovations require support from the Outer Setting, these themes are important when the innovation is community-based or relies on the community for support or resources, because they will directly influence equity in implementation and implementation success. These themes are especially needed to capture common resource constraints, e.g. in low- and middle- income countries (LMICs) [22].  Users identified specific gaps related to 1) values [24], including equity, and beliefs (e.g., white supremacy, racial bias) [25], and 2) economic (e.g., recession) [26], environmental (e.g., built environment), political, and technological conditions (e.g., IT infrastructure) [23,27,28].  ***Addition of New Constructs***  As a result, two broad constructs were added to capture the influence of these factors on implementation and/or delivery of the innovation; users may wish to add subconstructs to specify which themes are relevant to their project. | | | |
| Cosmopolitanism | The degree to which an organization is networked with other external organizations. | D. Partnerships & Connections | The Inner Setting is networked with external entities, including referral networks, academic affiliations, and professional organization networks. |
| ***User Feedback***  Many users felt the name of the Cosmopolitanism construct was confusing; they felt it was “not intuitive” and suggested language around network connections and size, regional cohesion, and partnerships.  ***Minor Revision of Construct Name***  As a result, the name of this construct was updated. | | | |
| External Policies & Incentives | A broad construct that includes external strategies to spread interventions including policy and regulations (governmental or other central entity), external mandates, recommendations and guidelines, pay-for-performance, collaboratives, and public or benchmark reporting. | E. Policies & Laws | Legislation, regulations, professional group guidelines and recommendations, or accreditation standards support implementation and/or delivery of the innovation. |
| *None* | *Construct added in the updated CFIR.* | F. Financing | Funding from external entities (e.g., grants, reimbursement) is available to implement and/or deliver the innovation. |
| ***User Feedback***  Users felt the External Policies & Incentives construct needed to be separated into multiple constructs or given subconstructs, and others expanded the definition or added constructs to capture payment schemes [23], reimbursement [29], and remuneration [30]. These additions are supported by a recent scoping review that highlights the importance of financing and the variety of financial strategies [31].  ***Separation of Construct***  As a result, this construct was separated into three constructs: Policies & Laws, Financing, and External Pressure: Performance-Measurement Pressure (see below for the last construct). | | | |
| *None* | *Construct added in the updated CFIR.* | G. External Pressure | External pressures drive implementation and/or delivery of the innovation.  *Use this construct to capture themes related to External Pressures that are not included in the subconstructs below.* |
| ***User Feedback***  Users identified a broad theme related to external pressure [23], which may emanate from multiple entities in the Outer Setting.  ***Addition of New Construct***  As a result, this construct was added, and one new construct and two previous constructs were included as subconstructs. | | | |
| *None* | *Subconstruct added in the updated CFIR.* | 1. Societal Pressure | Mass media campaigns, advocacy groups, or social movements or protests drive implementation and/or delivery of the innovation. |
| ***User Feedback***  Users identified gaps related to societal and/or mass media pressure [26].  ***Addition of New Subconstruct***  As a result, this subconstruct was added under the new External Pressure construct. | | | |
| Peer Pressure | Mimetic or competitive pressure to implement an intervention; typically, because most or other key peer or competing organizations have already implemented or in a bid for a competitive edge. | 2. Market Pressure | Competing with and/or imitating peer entities drives implementation and/or delivery of the innovation. |
| ***User Feedback***  Users found the name of the Peer Pressure construct confusing; they thought peer referred to an individual peer in the Inner Setting vs. a peer entity in the Outer Setting. One user suggested renaming the construct “Competition” while another suggested “Market Forces” (survey responses).  ***Major Revision of Construct Name & Relocation of Construct***  As a result, following review of these concepts, the name of this construct was updated, and it was added as a subconstruct under the new External Pressure construct. | | | |
| *None* | *See Outer Setting: External Policies & Incentives construct.* | 3. Performance-Measurement Pressure | Quality or benchmarking metrics or established service goals drive implementation and/or delivery of the innovation. |

# Inner Setting

## Domain-Level User Feedback & CFIR Updates

| **III. INNER SETTING DOMAIN** | *No specific guidance provided at the domain-level in the original CFIR.* | **III. INNER SETTING DOMAIN** | ***Inner Setting:*** The setting in which the innovation is implemented, e.g., hospital, school, city. There may be multiple Inner Settings and/or multiple levels within the Inner Setting, e.g., unit, classroom, team.   ***Project Inner Setting(s):*** [Document the actual Inner Setting in the project, e.g., type, location, and the boundary between the Outer Setting and the Inner Setting.] |
| --- | --- | --- | --- |
| ***User Feedback***  Users found it difficult to operationalize the Inner vs. Outer Setting in their projects; they found the domain labels “unintuitive” and gave diverse recommendations from combining them into a single domain (because it was challenging to define a boundary between them) to separating each of them into multiple levels [32], e.g., to account for teams and units within the Inner Setting [33,34].  ***Addition of Domain-Level Guidance***  As a result, domain-level guidance was added to help users delineate and define the Inner Setting for their project. While it can be challenging to differentiate the Inner and Outer Settings, it is nonetheless important to define and delineate the boundary between the two domains for accurate attribution to implementation outcomes and to identify appropriate levels of future intervention.  ***User Feedback***  In addition, users found it difficult to distinguish between some Inner Setting constructs, e.g., Access to Knowledge & Information vs. Networks & Communications.  ***Addition of Domain-Level Guidance***  As a result, text was added to help distinguish between seemingly similar constructs, by clarifying that Constructs A – D are persistent general characteristics of the Inner Setting (e.g., Networks & Communication), while Constructs E – K are specific to implementation and/or delivery of the innovation (e.g., Access to Knowledge & Information). | | | |

## Construct-Level User Feedback & CFIR Updates

| **Old Construct Name** | **Old Construct Definition** | **Construct Name** | **Construct Definition** *The degree to which:* |
| --- | --- | --- | --- |
| *None* | *No specific guidance provided at the domain-level in the original CFIR.* | *Note:* | *Constructs A – D exist in the Inner Setting regardless of implementation and/or delivery of the innovation, i.e., they are persistent general characteristics of the Inner Setting.* |
| Structural Characteristics | The social architecture, age, maturity, and size of an organization. | A. Structural Characteristics | Infrastructure components support functional performance of the Inner Setting.  *Use this construct to capture themes related to Structural Characteristics that are not included in the subconstructs below.* |
| *None* | *Subconstruct added in the updated CFIR.* | 1. Physical Infrastructure | Layout and configuration of space and other tangible material features support functional performance of the Inner Setting. |
| *None* | *Subconstruct added in the updated CFIR.* | 2. Information Technology Infrastructure | Technological systems for tele-communication, electronic documentation, and data storage, management, reporting, and analysis support functional performance of the Inner Setting. |
| *None* | *Subconstruct added in the updated CFIR.* | 3. Work Infrastructure | Organization of tasks and responsibilities within and between individuals and teams, and general staffing levels, support functional performance of the Inner Setting. |
| ***User Feedback***  Users felt the Structural Characteristics construct was too broad and requested more guidance and/or recommended dividing into subconstructs; users recommended constructs related to physical infrastructure [23,27] and technological infrastructure [23,27,28]. In addition, users recommended reviewing the recent multi-country analysis of contextual features by Squires et. al., which includes a construct specific to work structures [35].  ***Addition of New Subconstructs***  As a result, we added the Physical Infrastructure, Information Technology Infrastructure, and Work Infrastructure subconstructs. | | | |
| Networks & Communications | The nature and quality of webs of social networks and the nature and quality of formal and informal communications within an organization. | B. Relational Connections | There are high quality formal and informal relationships, networks, and teams within and across Inner Setting boundaries (e.g., structural, professional). |
|  |  | C. Communications | There are high quality formal and informal information sharing practices within and across Inner Setting boundaries (e.g., structural, professional). |
| ***User Feedback***  Users stated that the Networks & Communications construct included two distinct themes: networks and communications. In addition, many users noted the absence of teams in the original CFIR [22,23].  ***Separation of Construct***  As a result, this construct was separated into the Relational Connections construct, which highlights the role of teams in the CFIR, and the Communications construct. | | | |
| Culture | Norms, values, and basic assumptions of a given organization. | D. Culture | There are shared values, beliefs, and norms across the Inner Setting.  *Use this construct to capture themes related to Culture that are not included in the subconstructs below.* |
| *None* | *Subconstruct added in the updated CFIR.* | 1. Human Equality-Centeredness | There are shared values, beliefs, and norms about the inherent equal worth and value of all human beings. |
| *None* | *Subconstruct added in the updated CFIR.* | 2. Recipient-Centeredness | There are shared values, beliefs, and norms around caring, supporting, and addressing the needs and welfare of recipients. |
| *None* | *Subconstruct added in the updated CFIR.* | 3. Deliverer-Centeredness | There are shared values, beliefs, and norms around caring, supporting, and addressing the needs and welfare of deliverers. |
| *None* | *See Inner Setting: Learning Climate construct.* | 4. Learning-Centeredness | There are shared values, beliefs, and norms around psychological safety, continual improvement, and using data to inform practice. |
| ***User Feedback***  Users felt the Culture construct was too broad, with one stating, it “ends up becoming my ‘I don't know where else this fits’ bucket” (survey response). In addition, many users noted the absence of equity considerations in the original CFIR [22,36].  ***Addition of New Subconstructs***  As noted in the original CFIR, there are many ways to conceptualize and define culture, with little consensus in the literature. However, our team and other users noted gaps in the CFIR related to several aspects of culture that are relevant in implementation work. As a result, four subconstructs were added to reinforce the importance of identifying and addressing these issues at a cultural and system-level.  Human Equality-Centeredness captures the importance of assessing “specific context elements, more specifically racism, patriarchy and misogyny, that [are] so much a part of the care that we provide” (survey response).  Recipient-Centeredness captures the concept of patient safety culture [37] and reflects one component of the original Patient Needs & Resources construct about the importance of centering recipients in the Inner Setting (the other component, awareness of patient needs, is captured by the Roles Subdomain: Innovation Recipients and Characteristics Subdomain: Need constructs below).    Deliverer-Centeredness captures the importance of recognizing and addressing the needs of deliverers, and aligns with the evolution of the “Triple Aim” (enhancing patient experience, improving population health, reducing costs) into the “Quadruple Aim,” which aims to improve the work-life and well-being of clinicians and staff [38].  Learning-Centeredness replaces the original Learning Climate subconstruct (previously under Implementation Climate). Users found it challenging to distinguish between Implementation Climate: Learning Climate versus Culture, but noted the importance of this concept, including psychological safety. An adapted version of the original CFIR includes this construct [23], reinforcing its importance; however, we have moved the concept of “learning” to Culture for two reasons. First, we have removed Implementation Climate because of indistinct definitions of culture versus climate (see more details below). Second, we recognize the important role of learning culture as a prominent theory in healthcare, especially as systems press forward to become “learning systems” [39,40]. Ideally, continuous learning is occurring throughout the Inner Setting with visible evidence of engaged process improvement (including historical evidence of previous change initiatives [41]), use of data to inform change, and the necessary relational environment [33,42,43]. | | | |
| *None* | *No specific guidance provided at the domain-level in the original CFIR.* | *Note:* | *Constructs E – K are specific to the implementation and/or delivery of the innovation****.*** |
| Implementation Climate | The absorptive capacity for change, shared receptivity of involved individuals to an intervention and the extent to which use of that intervention will be rewarded, supported, and expected within their organization. | *None* | *Construct removed from the updated CFIR; reclassified as an antecedent assessment in the CFIR Outcomes Addendum* [1]*.* |
| ***Removal of Construct: Construct Reclassified as Antecedent Assessment***  Since publication of the original CFIR, there has been continued conceptual and measurement development of Implementation Climate as a potential predictor of implementation outcomes, but there is little consensus in the literature on how it should be defined [44]. In the CFIR Outcomes Addendum [1], we provide more detailed rationale for conceptualizing Implementation Climate as an “Antecedent Assessment” [45]. These measures are a function of multiple CFIR determinants and potential antecedents to implementation outcomes. As a result, Implementation Climate was removed from the CFIR and its subconstructs have been elevated to the construct level. | | | |
| Tension for Change | The degree to which stakeholders perceive the current situation as intolerable or needing change. | E. Tension for Change | The current situation is intolerable and needs to change. |
| *No additional changes (See Framework-Level User Feedback & CFIR updates above).* | | | |
| Compatibility | The degree of tangible fit between meaning and values attached to the intervention by involved individuals, how those align with individuals’ own norms, values, and perceived risks and needs, and how the intervention fits with existing workflows and systems. | F. Compatibility | The innovation fits with workflows, systems, and processes. |
| ***User Feedback***  Users stated that the Compatibility construct included two distinct themes: compatibility with work processes and compatibility with values, with the latter overlapping with the original Goals & Feedback construct.  ***Major Revision of Construct Definition***  As a result, the definition was updated to only include the former; facets of compatibility with values are captured in the Individuals Domain at the individual level and by the new Mission Alignment construct at the collective level.  ***User Feedback***  Users commented that this construct should be in the Innovation Domain to align with Roger’s Diffusion of Innovations [36].  ***Addition of Domain-Level Guidance***  While we acknowledge the lack of agreement between frameworks, we conceptualize Compatibility as a boundary spanning construct between the Innovation Domain and the Inner Setting Domain; whether the innovation is compatible depends on its fit *within the Inner Setting*. In contrast, the other constructs within the Innovation Domain apply to inherent characteristics of the innovation regardless of where it is implemented. | | | |
| Relative Priority | Individuals’ shared perception of the importance of the implementation within the organization. | G. Relative Priority | Implementing and delivering the innovation is important compared to other initiatives. |
| ***User Feedback***  Users questioned if Relative Priority included priority of delivery of the innovation as well as initial implementation.  ***Major Revision of Construct Definition***  As a result, the definition was expanded to include delivery of the innovation. | | | |
| Organizational Incentives & Rewards | Extrinsic incentives such as goal-sharing awards, performance reviews, promotions, and raises in salary and less tangible incentives such as increased stature or respect. | H. Incentive Systems | Tangible and/or intangible incentives and rewards and/or disincentives and punishments support implementation and delivery of the innovation. |
| ***User Feedback***  Users were unclear if the Organizational Incentives & Rewards construct included disincentives (or punishments) as well as incentives (or rewards).  ***Minor Revision of Construct Name & Definition***  The construct name and definition were updated to incorporate the broader concept of incentive systems, which includes multiple techniques for behavior modification [46,47]. | | | |
| Goals & Feedback | The degree to which goals are clearly communicated, acted upon, and fed back to staff, and alignment of that feedback with goals. | I. Mission Alignment | Implementing and delivering the innovation is in line with the overarching commitment, purpose, or goals in the Inner Setting. |
| ***User Feedback***  Users were unclear about the difference between the Goals & Feedback construct and the Reflecting & Evaluating construct; they noted that setting goals was an important part of the implementation process and did not understand the placement of this construct in the Inner Setting. In addition, users noted that there was overlap between this construct and Learning Climate due to “the general quality improvement themes of both” (survey response).  ***Major Revision of Construct Name & Definition***  This construct was intended to capture the general nature of setting goals and providing feedback in the Inner Setting (i.e., is the Inner Setting goal driven? Is the Inner Setting data driven?) *and* the alignment of Inner Setting goals with innovation goals (i.e., Does the innovation align with accomplishing Inner Setting goals/mission?). Given the overlap of the first theme and Learning Climate (Culture: Learning-Centeredness in the updated CFIR), the name and definition of this construct were updated to highlight the importance of aligning the innovation with the Inner Setting mission; “alignment to achieve consistency of organization goals with resource allocation and actions at all levels of the organization” is an important implementation determinant [48]. | | | |
| Learning Climate | A climate in which: a) leaders express their own fallibility and need for team members’ assistance and input; b) team members feel that they are essential, valued, and knowledgeable partners in the change process; c) individuals feel psychologically safe to try new methods; and d) there is sufficient time and space for reflective thinking and evaluation. | *None* | *Construct renamed and relocated; see Inner Setting: Culture: Learning-Centeredness.* |
| ***Construct Renamed & Relocated***  *See Inner Setting: Culture: Learning-Centeredness for user feedback and CFIR updates.* | | | |
| Readiness for Implementation | Tangible and immediate indicators of organizational commitment to its decision to implement an intervention. | *None* | *Construct removed from the updated CFIR; reclassified as an antecedent assessment in the CFIR Outcomes Addendum* [1]*.* |
| ***Removal of Construct: Construct Reclassified as Antecedent Assessment***  Since publication of the original CFIR, there has been continued conceptual and measurement development of Readiness for Implementation as a potential predictor of implementation outcomes, but there is little consensus in the literature on how it should be defined, and it comprises more than the three subconstructs in the original CFIR of Leadership Engagement, Available Resources, and Access to Knowledge and Information [33,49]. In the CFIR Outcomes Addendum [1], we provide more detailed rationale for conceptualizing Readiness for Implementation as an “Antecedent Assessment” [45]. These measures are a function of multiple CFIR determinants and potential antecedents to implementation outcomes. As a result, Readiness for Implementation was removed from the CFIR and the subconstructs have been elevated to the construct level. | | | |
| Leadership Engagement | Commitment, involvement, and accountability of leaders and managers with the implementation. | *None* | *Construct separated, renamed, and relocated; see Individuals Domain: Roles Subdomain: High-Level & Mid-Level Leaders; and Characteristics Subdomain: Motivation.* |
| ***Construct Separated, Renamed & Relocated***  *See Individuals Domain: Roles Subdomain: High-Level & Mid-Level Leaders and Characteristics Subdomain: Motivation for user feedback and CFIR updates.* | | | |
| Available Resources | The level of resources dedicated for implementation and on-going operations including money, training, education, physical space, and time. | J. Available Resources | Resources are available to implement and deliver the innovation.  *Use this construct to capture themes related to Available Resources that are not included in the subconstructs below.* |
| *None* | *Subconstruct added in the updated CFIR.* | 1. Funding | Funding is available to implement and deliver the innovation. |
| *None* | *Subconstruct added in the updated CFIR.* | 2. Space | Physical space is available to implement and deliver the innovation. |
| *None* | *Subconstruct added in the updated CFIR.* | 3. Materials & Equipment | Supplies are available to implement and deliver the innovation. |
| ***User Feedback***  Users noted the overlap of this construct with Access to Knowledge & Information (in terms of training and education) and were unclear if staffing and/or staff time should be included here as a resource, e.g., “available staff,” or under Engaging as related to the roles important to implementation. In addition, users felt that distinctions between the types of resources included in this construct were important.  ***Addition of New Subconstructs***  As result, three subconstructs were added, and did not include availability of training, which is part of Access to Knowledge & Information, nor availability of staffing to implement and/or deliver the innovation, which is part of the Individuals Domain: Characteristics Subdomain: Opportunity construct. | | | |
| Access to knowledge and information | Ease of access to digestible information and knowledge about the intervention and how to incorporate it into work tasks. | K. Access to Knowledge & Information | Guidance and/or training is accessible to implement and deliver the innovation. |
| *No additional changes (See Framework-Level User Feedback & CFIR updates above).* | | | |

# Individuals: Roles & Characteristics

## Domain-Level User Feedback & CFIR Updates

| **IV. CHARACTERISTICS OF INDIVIDUALS** | *No specific guidance provided at the domain-level in the original CFIR.* | **IV. INDIVIDUALS DOMAIN** | ***Individuals:*** The roles and characteristics of individuals. |
| --- | --- | --- | --- |
| ***User Feedback***  Users noted the “CFIR’s predominant focus on determinants of implementation at organisation and system levels rather than individual level” [26] and felt the framework did not provide nor operationalize “sufficient individual-level constructs,” [26] [23,27,41,50–52]. As a result, they found it difficult to operationalize this domain; they were unclear which individuals were included and felt that the existing constructs overlapped with constructs in other domains while failing to capture more relevant characteristics. One user summarized this feedback well: “[The CFIR needs to focus] more on who the individuals are and their underlying characteristics” (survey response).  ***Reorganization of Domain & Minor Revision of Domain Name***  As a result, this domain was restructured to include a Role Subdomain and a Characteristics Subdomain. See below for additional detail. | | | |

# Roles

## Subdomain User Feedback & CFIR Updates

| *None* | *Roles Subdomain added in the updated CFIR.* | **ROLES SUBDOMAIN** | ***Project Roles:*** [Document the roles applicable to the project and their location in the Inner or Outer Setting.] |
| --- | --- | --- | --- |
| ***User Feedback***  Users were not clear which individuals to include in this domain; specifically, whether this domain included deliverers and/or recipients. This confusion was understandable given that in the original CFIR, roles were spread across three different domains: Patient Needs and Resources was listed in the Outer Setting, Leadership Engagement was listed in the Inner Setting, and implementation-specific roles were listed in the Process Domain (e.g., Formally Appointed Internal Implementation Leaders).  ***Relocation of Constructs & Addition of Domain-Level Guidance***  As a result, all roles have been consolidated within the Individuals Domain in the updated CFIR. Capturing additional detail about each role (e.g., level of engagement), requires coding the Role and relevant Characteristic construct. Furthermore, users have flexibility in specifying whether each role resides in the Inner or Outer Setting (e.g., an Implementation Facilitator affiliated with an Outer Setting entity versus an Implementation Lead employed by the Inner Setting). | | | |

## Construct-Level User Feedback & CFIR Updates

| **Old Construct Name** | **Old Construct Definition** | **Construct Name** | **Construct Definition** |
| --- | --- | --- | --- |
| *None* | *See Inner Setting: Leadership Engagement.* | A. High-level Leaders | Individuals with a high level of authority, including key decision-makers, executive leaders, or directors. |
| *None* | *See Inner Setting: Leadership Engagement* | B. Mid-level Leaders | Individuals with a moderate level of authority, including leaders supervised by a high-level leader and who supervise others. |
| ***User Feedback***  Users were unclear how the Inner Setting: Leadership Engagement construct differed from the types of leadership roles listed in the Process Domain. In addition, some users recommended distinguishing between multiple levels of leadership, including “clinical and management leadership” [53].  ***Separation of Construct***  As a result, this construct was separated into two constructs. | | | |
| *None* | *See Process: Engaging: Opinion Leaders.* | C. Opinion Leaders | Individuals with informal influence on the attitudes and behaviors of others. |
| ***User Feedback***  Users were confused about the difference between the Opinion Leader role and other roles, e.g., Champion.  ***Minor Revision of Construct Definition***  As a result, the definition was updated to include only informal (not formal) influence. | | | |
| *None* | *See Process: Engaging: External Change Agents.* | D. Implementation Facilitators | Individuals with subject matter expertise who assist, coach, or support implementation. |
| ***User Feedback***  Users found the External Change Agent role confusing and were unclear why it was limited to the Outer Setting.  ***Minor Revision of Construct Name & Major Revision of Construct Definition***  As a result, this construct was renamed; this role can exist in either the Inner or Outer setting, as it includes any individual who provides guidance to the Implementation Leads [54,55]. | | | |
| *None* | *See Process: Engaging: Formally Appointed Internal Implementation Leaders & Champions.* | E. Implementation Leads | Individuals who lead efforts to implement the innovation. |
| ***User Feedback***  Users noted confusion between the Formally Appointed Internal Implementation Leader and Champion role. It is difficult to provide a distinct definition for the Champion role, because any of the listed roles can champion implementation. For example, Ilot et al. found that “none of the instigators” of implementation were formally appointed, though some ultimately assumed that role, as well as the role of Champion and Opinion Leader [53]. Furthermore, published studies often use Champion to describe the person leading implementation [56].  ***Combination of Constructs***  As a result, we have combined the Champion and Formally Appointed Internal Implementation Leader constructs. | | | |
| *None* | *Construct added in the updated CFIR.* | F. Implementation Team Members | Individuals who collaborate with and support the Implementation Leads to implement the innovation, ideally including Innovation Deliverers and Recipients. |
| *None* | *Construct added in the updated CFIR.* | G. Other Implementation Support | Individuals who support the Implementation Leads and/or Implementation Team Members to implement the innovation. |
| ***User Feedback***  Users noted a gap in key roles related to the individuals who assist the Implementation Leads with implementation. In addition, many users noted the absence of teams and team characteristics in the original CFIR [22,23].  ***Addition of New Constructs***  As a result, two roles were added. The first role includes all individuals serving on the implementation team, and ideally includes Innovation Deliverers and Recipients. The second role was added to accommodate the diversity of implementation models used by researchers and implementers. Users are encouraged to fully describe the individuals, functions, and expectations for each type of Other Implementation Support Role. | | | |
| *None* | *Construct added in the updated CFIR.* | H. Innovation Deliverers | Individuals who are directly or indirectly delivering the innovation. |
| ***User Feedback***  Users recommended adding this role; although the Characteristics of Individuals Domain was intended to be used to capture information about the Innovation Deliverers, this role was not explicitly included in the original CFIR.  ***Addition of New Construct***  As a result, this role was added. | | | |
| *None* | *See Outer Setting: Patient Needs & Resources.* | I. Innovation Recipients | Individuals who are directly or indirectly receiving the innovation. |
| ***User Feedback***  Users questioned the placement of recipients (patients) in the Outer Setting and felt that the CFIR needed additional constructs to better center patients. In addition, users stated that the Patient Needs & Resources construct included two distinct themes; Godbee et al. separated this construct into knowledge of recipient needs and prioritization of recipient needs [57], while Dy et al. added patient-centered care to the Inner Setting to capture the latter theme [23].  ***Construct Separated***  As a result, the Patient Needs & Resources construct was separated into the Inner Setting Domain: Culture: Recipient-Centeredness construct (to capture the prioritization of recipient needs and the concept of patient-centered care) and the Individuals Domain: Innovation Recipients and Need constructs (to capture knowledge of recipient needs). In addition, constructs were added to the Implementation Process Domain (Assessing Needs: Innovation Recipients; Engaging: Innovation Recipients) to reinforce the importance of including Recipients in the implementation process*.* | | | |

# Characteristics

## Subdomain User Feedback & CFIR Updates

| *None* | *Characteristics Subdomain added in the updated CFIR.* | **CHARACTERISTICS SUBDOMAIN** | ***Project Characteristics:*** [Document the characteristics applicable to the roles in the project based on the COM-B system [58] or role-specific theories.] |
| --- | --- | --- | --- |
| ***User Feedback***  Users felt the constructs in the Characteristics Domain overlapped with constructs in other domains, e.g., Knowledge and Beliefs overlapped with all constructs in the Innovation Domain. In addition, they felt this domain failed to capture more relevant characteristics related to professional roles and identities, skills and capabilities, autonomy, and level of involvement [23,27,52]. Some CFIR users combine this domain with the Theoretical Domains Framework (TDF), which was developed with the intent “to simplify and integrate a plethora of behavior change theories and make theory more accessible to, and usable by, other disciplines” [59]. The COM-B system was developed as a simplified system by which to acknowledge key domains related to behavior change based on US consensus of behavioral theorists and a principle of criminal law defining specific prerequisites for volitional behavior [58].  ***Removal of Existing Constructs & Addition of New Constructs***  As a result, we replaced the original constructs in this domain with constructs based on the COM-B system [58]. The COM-B posits that broad categories of Capability, Opportunity, and Motivation will lead to the desired behavior. We also added the Need construct, given that the needs of all constituencies are important determinants to implementation outcomes.  We encourage users to add additional constructs as appropriate. For example, theories, models, and frameworks related to:   - Behavior change, e.g., the Theoretical Domains Framework [59,60], the Theory of Planned Behavior [61] or the Social Ecological Theory [62] may provide constructs relevant for Innovation Recipients and Innovation Deliverers. - Facilitation [63,64] and project management [65,66] may provide constructs relevant for Implementation Facilitators and Implementation Leads. - Leadership [63,64] may provide constructs relevant for High- and Mid-Level Leaders.   These role-specific constructs may be mapped to the broader COM-B constructs; for example, all 14 domains of the Theoretical Domains Framework (TDF) map to the COM-B system [58]. | | | |

## Construct-Level User Feedback & CFIR Updates

| **Old Construct Name** | **Old Construct Definition** | **Construct Name** | **Construct Definition:**  *The degree to which:* |
| --- | --- | --- | --- |
| Knowledge & Beliefs about the Intervention | Individuals’ attitudes toward and value placed on the intervention as well as familiarity with facts, truths, and principles related to the intervention. | *None* | *Construct removed from the updated CFIR.* |
| Self-efficacy | Individual belief in their own capabilities to execute courses of action to achieve implementation goals. | *None* | *Construct removed from the updated CFIR.* |
| Individual Stage of Change | Characterization of the phase an individual is in, as he or she progresses toward skilled, enthusiastic, and sustained use of the intervention. | *None* | *Construct removed from the updated CFIR.* |
| Individual Identification with Organization | A broad construct related to how individuals perceive the organization and their relationship and degree of commitment with that organization. | *None* | *Construct removed from the updated CFIR.* |
| Other Personal Attributes | A broad construct to include other personal traits such as tolerance of ambiguity, intellectual ability, motivation, values, competence, capacity, and learning style. | *None* | *Construct removed from the updated CFIR.* |
| ***User Feedback***  Users felt these constructs overlapped with existing constructs.  ***Removal of Existing Constructs***  As a result, these constructs were removed, but may be mapped to the new constructs in this domain as indicated in each construct listed below. | | | |
| *None* | *Construct added in the updated CFIR.* | A. Need | The individual(s) has deficits related to survival, well-being, or personal fulfillment, which will be addressed by implementation and/or delivery of the innovation. |
| ***User Feedback***  Users noted that the “aims” and “wishes/needs” of individuals were lacking in the original CFIR [41]. Although perceptions of patient needs were captured in the Outer Setting: Patient Needs & Resources construct in the original CFIR, it is important to capture this theme for all individuals.    ***Addition of New Construct***  As a result, the Need construct was added to this domain in addition to the COM-B constructs. | | | |
| *None* | *Construct added in the updated CFIR.* | B. Capability | The individual(s) has interpersonal competence, knowledge, and skills to fulfill Role. |
| ***User Feedback***  Users noted that the “skills and competencies" of individuals should be included in this domain, and this was included as a construct in adaptations of the CFIR [23,27].  ***Addition of New Construct***  As a result, the Capability construct was added to capture these themes. The original Knowledge & Beliefs, Self-Efficacy, and Other Personal Attributes: Intellectual Ability, Competence constructs may map to this construct. | | | |
| *None* | *Construct added in the updated CFIR.* | C. Opportunity | The individual(s) has availability, scope, and power to fulfill Role. |
| ***User Feedback***  Users were unclear where to capture themes related to staff availability for implementation and/or delivery of the innovation, e.g., available staffing, staff hours, as well as themes related to the degree of “autonomy and control” of individuals [52]; these themes were included as constructs in adaptations of the CFIR [23,27].  ***Addition of New Construct***  As a result, the Opportunity construct was included to capture characteristics that are conferred onto individuals by broader organizations or systems, e.g., the time and authority granted to the Implementation Lead to fulfill their role. The original Other Personal Attributes: Capacity construct may map to this construct. | | | |
| *None* | *Construct added in the updated CFIR.* | D. Motivation | The individual(s) is committed to fulfilling Role. |
| ***User Feedback***  Users noted that the CFIR failed to capture the overall motivation and commitment of individuals to fulfill their role, and this theme was included as a construct in adaptations of the CFIR [23,27]. Although perceptions of the commitment of leaders was captured in the original Leadership Engagement construct, it is important to capture this theme for all individuals.  ***Addition of New Construct***  As a result, the Motivation construct was added. The original Individual Stage of Change, Individual Identification with Organization, and Other Personal Attributes: Motivation, Values constructs may map to this construct. | | | |

# Implementation Process

## Domain-Level User Feedback & CFIR Updates

| **V. PROCESS** | *No specific guidance provided at the domain-level in the original CFIR.* | **V. IMPLEMENTATION PROCESS DOMAIN** | ***Implementation Process:*** The activities and strategies used to implement the innovation.  ***Project Implementation Process:*** [Document the implementation process framework [67] and/or activities and strategies [14,15] being used to implement the innovation. Distinguish the implementation process used to implement the innovation (activities that end after implementation is complete) from the innovation (the “thing” that continues when implementation is complete) [8,13,16].] |
| --- | --- | --- | --- |
| ***User Feedback***  Users were unclear if the CFIR was intended to evaluate the innovation and/or the strategy being used to implement the innovation. While the CFIR is most often used to evaluate the implementation of innovations, it can be used to evaluate an implementation strategy if that *is* the innovation being evaluated.  ***Addition of Domain-Level Guidance***  Users must determine the goal of the evaluation and define the innovation and implementation strategy appropriately; distinguishing between the innovation and implementation strategy is necessary for accurate attribution to implementation outcomes and to identify appropriate areas for future intervention, e.g., did implementation fail due to negative perceptions of the innovation *itself* or of the implementation strategy being used to implement the innovation? As a result, domain-level guidance was added to advise users to document the implementation process.  ***User Feedback***  Users questioned the inclusion of the Implementation Process Domain because it includes strategies not contextual factors. In addition, users referred to the Expert Recommendations for Implementation Change strategies [15,68], noting the limited number of processes contained in this domain and the significant advances that have occurred in this area since the publication of the original CFIR.  ***Addition of New Constructs***  As a determinant framework, the CFIR includes determinants related to several spheres of influence: the innovation being implemented, the individuals involved, the settings, *and* the implementation process. The goal of this domain is to capture the use and quality of these implementation processes; the addition of the construct definition stem “the degree to which” works to highlight how these processes are determinants to implementation. In addition, this domain was expanded to include several new constructs based on recommendations from users, however, the updated CFIR is not designed to include the complete list of ERIC strategies, only activities that are common across approaches. | | | |

## Construct-Level User Feedback & CFIR Updates

| **Old Construct Name** | **Old Construct Definition** | **Construct Name** | **Construct Definition:** *The degree to which individuals:* |
| --- | --- | --- | --- |
| *None* | *Construct added in the updated CFIR.* | A. Teaming | Join together, intentionally coordinating and collaborating on interdependent tasks, to implement the innovation. |
| ***User Feedback***  Users noted the absence of the role of teams in the original CFIR [22,23]. In addition, Edmonson’s work has highlighted the importance of teaming, especially as part of organizational learning [42].  ***Addition of New Construct***  As a result, this construct was added. | | | |
| *None* | *Construct added in the updated CFIR.* | B. Assessing Needs | Collect information about priorities, preferences, and needs of people.  *Use this construct to capture themes related to Assessing Needs that are not included in the subconstructs below.* |
| *None* | *Subconstruct added in the updated CFIR.* | 1. Innovation Deliverers | Collect information about the priorities, preferences, and needs of deliverers to guide implementation and delivery of the innovation. |
| *None* | *Subconstruct added in the updated CFIR.* | 2. Innovation Recipients | Collect information about the priorities, preferences, and needs of recipients to guide implementation and delivery of the innovation. |
| ***User Feedback***  Users noted the absence of assessing needs in the original CFIR, and this was included as a construct in adaptations of the CFIR [23,27]. In addition, users felt that the CFIR needed additional constructs to center patients.  ***Addition of New Construct***  Assessing the needs of both recipients and deliverers to guide the implementation process is an important determinant to implementation success as well as equity in implementation. Assessing recipient needs facilitates patient-centered care and patient safety culture [37] while assessing deliverer needs facilitates the “Quadruple Aim,” which includes improving the work-life and well-being of clinicians and staff [38]. As a result, this construct and subconstructs were added. | | | |
| *None* | *Construct added in the updated CFIR.* | C. Assessing Context | Collect information to identify and appraise barriers and facilitators to implementation and delivery of the innovation. |
| ***User Feedback***  Users noted the absence of assessing context in the original CFIR, and this was included as a construct in an adaptation of the CFIR [23].  ***Addition of New Construct***  As a result, this construct was added. Note: The CFIR is designed to assess context; it can be used as part of the implementation process and to conduct the evaluation. | | | |
| Planning | The degree to which a scheme or method of behavior and tasks for implementing an intervention are developed in advance and the quality of those schemes or methods. | D. Planning | Identify roles and responsibilities, outline specific steps and milestones, and define goals and measures for implementation success in advance. |
| ***User Feedback***  While some users noted the absence of setting goals as an important implementation process and determinant, others confused the Inner Setting: Goals & Feedback construct with setting goals and combined it with the Process: Reflecting & Evaluating construct [28,34]. However, it is important to distinguish between the process of setting goals and the process of evaluating progress towards those goals.  ***Major Revision to Construct***  As a result, the definition of Planning was updated to explicitly include defining goals and measures; evaluating progress remains under Reflecting & Evaluating. | | | |
| *None* | *Construct added in the updated CFIR.* | E. Tailoring Strategies | Choose and operationalize implementation strategies to address barriers, leverage facilitators, and fit context. |
| ***User Feedback***  Users noted the absence of choosing and using strategies in the original CFIR, especially following the publication of the Expert Recommendations for Implementing Change (ERIC) [15,68].  ***Addition of New Construct***  As a result, this construct was added to highlight the importance of this step in the implementation process. Although all the constructs in this domain can be conceptualized as implementation strategies, the ERIC provides a more comprehensive and detailed list that can be used for specific situations. | | | |
| Engaging | Attracting and involving appropriate individuals in the implementation and use of the intervention through a combined strategy of social marketing, education, role modeling, training, and other similar activities. | F. Engaging | Attract and encourage participation in implementation and/or the innovation.  *Use this construct to capture themes related to Engaging that are not included in the subconstructs below.* |
| *None* | *Subconstruct added in the updated CFIR.* | 1. Innovation Deliverers | Attract and encourage deliverers to serve on the implementation team and/or to deliver the innovation. |
| ***User Feedback***  Users noted the absence of the engaging deliverers in the original CFIR.  ***Addition of New Subconstruct***  Engaging deliverers to serve on the implementation team and/or deliver the innovation is an important determinant to implementation; the inclusion of deliverers also serves the “Quadruple Aim,” which aims to improve the work-life and well-being of clinicians and staff [38]. As a result, this role was added as a subconstruct under Engaging. | | | |
| *None* | *Subconstruct added in the updated CFIR.* | 2. Innovation Recipients | Attract and encourage recipients to serve on the implementation team and/or participate in the innovation. |
| ***User Feedback***  Users noted the absence of engaging recipients in the original CFIR and felt that the CFIR needed additional constructs to center patients.  ***Addition of New Subconstruct***  Engaging recipients to serve on the implementation team and/or participate in the innovation is an important determinant to implementation and equity in implementation; the inclusion of recipients facilitates patient centered care and patient safety culture [37]. As a result, this role was added as a subconstruct under Engaging. | | | |
| Opinion Leaders | Individuals in an organization who have formal or informal influence on the attitudes and beliefs of their colleagues with respect to implementing the intervention. | *None* | *Subconstruct relocated; see Individuals Domain: Roles Subdomain: Opinion Leaders.* |
| ***Subconstruct Relocated***  See Individuals Domain: Roles Subdomain: Opinions Leaders for user feedback and CFIR updates. | | | |
| Formally appointed internal implementation leaders | Individuals from within the organization who have been formally appointed with responsibility for implementing an intervention as coordinator, project manager, team leader, or other similar role. | *None* | *Subconstructs combined, renamed, and relocated; see Individuals Domain: Roles Subdomain: Implementation Leads.* |
| Champions | “Individuals who dedicate themselves to supporting, marketing, and ‘driving through’ an [implementation]”, overcoming indifference or resistance that the intervention may provoke in an organization. |  |  |
| ***Subconstructs Combined, Renamed, & Relocated***  See Individuals Domain: Roles Subdomain: Implementation Leads for user feedback and CFIR updates. | | | |
| External Change Agents | Individuals who are affiliated with an outside entity who formally influence or facilitate intervention decisions in a desirable direction. | *None* | *Subconstruct renamed and relocated; see Individuals Domain: Roles Subdomain: Implementation Facilitators.* |
| ***Subconstruct Renamed & Relocated***  See Individuals Domain: Roles Subdomain: Implementation Facilitators for user feedback and CFIR updates. | | | |
| Executing | Carrying out or accomplishing the implementation according to plan. | G. Doing | Implement in small steps, tests, or cycles of change to trial and cumulatively optimize delivery of the innovation. |
| ***User Feedback***  Users noted that the Executing construct overlapped with fidelity, which is an implementation outcome, and commented on the importance of taking incremental approaches or using PDSA (Plan, Do, Study, Act) cycles in the process of implementation. This idea was included as a construct in adaptations of the CFIR [23,27]. In addition, users were unclear if the Innovation Domain: Trialability construct was intended to capture the inherent trialability of the innovation or the process of trialing.  ***Minor Revision of Construct Name & Definition***  As a result, we have updated the name and definition of this construct to focus on the importance of iterating and trialing during implementation. | | | |
| Reflecting & Evaluating | Quantitative and qualitative feedback about the progress and quality of implementation accompanied with regular personal and team debriefing about progress and experience. | H. Reflecting & Evaluating | Collect and discuss quantitative and qualitative information about the success of implementation and/or the innovation.  *Use this construct to capture themes related to Reflecting & Evaluating that are not included in the subconstructs below.* |
| *None* | *Subconstruct added in the updated CFIR.* | 1. Implementation | Collect and discuss quantitative and qualitive information about the success of implementation. |
| *None* | *Subconstruct added in the updated CFIR.* | 2. Innovation | Collect and discuss quantitative and qualitative information about the success of the innovation. |
| ***User Feedback***  Users felt this construct was too broad; one user noted that teams reflected on both the progress of implementation, e.g., reaching implementation milestones, as well as the progress of the innovation, e.g., success rates for patients.  ***Addition of New Subconstructs***  These two processes align with the types of outcomes highlighted in the CFIR Outcomes Addendum: Implementation Outcomes and Innovation Outcomes [1]. As a result, these were added as subconstructs under Reflecting & Evaluating. | | | |
| *None* | *Construct added in the updated CFIR.* | I. Adapting | Modify the innovation and/or the Inner Setting for optimal fit and integration into work processes. |
| ***User Feedback***  Users noted the absence of adapting in the original CFIR. In addition, adaptations were made to both innovations *and* the Inner Setting to be “compatible with setting constraints, reimbursement policies, patient needs, or therapeutic style” [26,69,70].  ***Addition of New Construct***  As a result, this construct was added. | | | |

# References

[1] Damschroder LJ, Reardon CM, Opra Widerquist MA, Lowery J. Conceptualizing outcomes for use with the Consolidated Framework for Implementation Research (CFIR): the CFIR Outcomes Addendum. Implementation Sci 2022;17:7. https://doi.org/10.1186/s13012-021-01181-5.

[2] Pithara C, Farr M, Sullivan SA, Edwards HB, Hall W, Gadd C, et al. Implementing a Digital Tool to Support Shared Care Planning in Community-Based Mental Health Services: Qualitative Evaluation. J Med Internet Res 2020;22:e14868. https://doi.org/10.2196/14868.

[3] Ware P, Ross HJ, Cafazzo JA, Laporte A, Gordon K, Seto E. Evaluating the Implementation of a Mobile Phone–Based Telemonitoring Program: Longitudinal Study Guided by the Consolidated Framework for Implementation Research. JMIR Mhealth Uhealth 2018;6:e10768. https://doi.org/10.2196/10768.

[4] Ruble L, McGrew JH, Snell-Rood C, Adams M, Kleinert H. Adapting COMPASS for youth with ASD to improve transition outcomes using implementation science. School Psychology 2019;34:187–200. https://doi.org/10.1037/spq0000281.

[5] Williams EC, Johnson ML, Lapham GT, Caldeiro RM, Chew L, Fletcher GS, et al. Strategies to implement alcohol screening and brief intervention in primary care settings: A structured literature review. Psychology of Addictive Behaviors 2011;25:206–14. https://doi.org/10.1037/a0022102.

[6] Sorensen JL, Kosten T. Developing the tools of implementation science in substance use disorders treatment: Applications of the consolidated framework for implementation research. Psychology of Addictive Behaviors 2011;25:262–8. https://doi.org/10.1037/a0022765.

[7] Cole CB, Pacca J, Mehl A, Tomasulo A, van der Veken L, Viola A, et al. Toward communities as systems: a sequential mixed methods study to understand factors enabling implementation of a skilled birth attendance intervention in Nampula Province, Mozambique. Reprod Health 2018;15:132. https://doi.org/10.1186/s12978-018-0574-8.

[8] Curran GM. Implementation science made too simple: a teaching tool. Implement Sci Commun 2020;1:27. https://doi.org/10.1186/s43058-020-00001-z.

[9] Albrecht L, Archibald M, Arseneau D, Scott SD. Development of a checklist to assess the quality of reporting of knowledge translation interventions using the Workgroup for Intervention Development and Evaluation Research (WIDER) recommendations. Implementation Sci 2013;8:52. https://doi.org/10.1186/1748-5908-8-52.

[10] Butler M, Epstein RA, Totten A, Whitlock EP, Ansari MT, Damschroder LJ, et al. AHRQ series on complex intervention systematic reviews—paper 3: adapting frameworks to develop protocols. Journal of Clinical Epidemiology 2017;90:19–27. https://doi.org/10.1016/j.jclinepi.2017.06.013.

[11] The AIMD Writing/Working Group, Bragge P, Grimshaw JM, Lokker C, Colquhoun H. AIMD - a validated, simplified framework of interventions to promote and integrate evidence into health practices, systems, and policies. BMC Med Res Methodol 2017;17:38. https://doi.org/10.1186/s12874-017-0314-8.

[12] Hoffmann TC, Glasziou PP, Boutron I, Milne R, Perera R, Moher D, et al. Better reporting of interventions: template for intervention description and replication (TIDieR) checklist and guide. BMJ 2014;348:g1687. https://doi.org/10.1136/bmj.g1687.

[13] Lengnick-Hall R, Gerke DR, Proctor EK, Bunger AC, Phillips RJ, Martin JK, et al. Six practical recommendations for improved implementation outcomes reporting. Implementation Sci 2022;17:16. https://doi.org/10.1186/s13012-021-01183-3.

[14] Powell BJ, McMillen JC, Proctor EK, Carpenter CR, Griffey RT, Bunger AC, et al. A compilation of strategies for implementing clinical innovations in health and mental health. Med Care Res Rev 2012;69:123–57. https://doi.org/10.1177/1077558711430690.

[15] Powell BJ, Waltz TJ, Chinman MJ, Damschroder LJ, Smith JL, Matthieu MM, et al. A refined compilation of implementation strategies: results from the Expert Recommendations for Implementing Change (ERIC) project. Implementation Science 2015;10:21.

[16] Pinnock H, Barwick M, Carpenter CR, Eldridge S, Grandes G, Griffiths CJ, et al. Standards for Reporting Implementation Studies (StaRI) Statement. BMJ 2017:i6795. https://doi.org/10.1136/bmj.i6795.

[17] Damschroder LJ, Aron DC, Keith RE, Kirsh SR, Alexander JA, Lowery JC. Fostering implementation of health services research findings into practice: a consolidated framework for advancing implementation science. Implement Sci 2009;4:50. https://doi.org/10.1186/1748-5908-4-50.

[18] Ho M, Livingston P, Bould MD, Nyandwi JD, Nizeyimana F, Uwineza JB, et al. Barriers and facilitators to implementing a regional anesthesia service in a low-income country: a qualitative study. Pan Afr Med J 2019;32. https://doi.org/10.11604/pamj.2019.32.152.17246.

[19] Rogers E. Diffusion of Innovations. 5th ed. New York, NY: Free Press; 2003.

[20] Moecker R, Terstegen T, Haefeli WE, Seidling HM. The influence of intervention complexity on barriers and facilitators in the implementation of professional pharmacy services – A systematic review. Research in Social and Administrative Pharmacy 2021;17:1651–62. https://doi.org/10.1016/j.sapharm.2021.01.013.

[21] Lewin S, Hendry M, Chandler J, Oxman AD, Michie S, Shepperd S, et al. Assessing the complexity of interventions within systematic reviews: development, content and use of a new tool (iCAT_SR). BMC Med Res Methodol 2017;17:76. https://doi.org/10.1186/s12874-017-0349-x.

[22] Means AR, Kemp CG, Gwayi-Chore M-C, Gimbel S, Soi C, Sherr K, et al. Evaluating and optimizing the consolidated framework for implementation research (CFIR) for use in low-and middle-income countries: a systematic review. Implementation Science 2020;15:1–19. https://doi.org/10.1001/jamasurg.2017.5565.

[23] Dy SM, Ashok M, Wines RC, Rojas Smith L. A Framework to Guide Implementation Research for Care Transitions Interventions: Journal for Healthcare Quality 2015;37:41–54. https://doi.org/10.1097/01.JHQ.0000460121.06309.f9.

[24] Merlo G, Page K, Zardo P, Graves N. Applying an Implementation Framework to the Use of Evidence from Economic Evaluations in Making Healthcare Decisions. Appl Health Econ Health Policy 2019;17:533–43. https://doi.org/10.1007/s40258-019-00477-4.

[25] Tiderington E, Ikeda J, Lovell A. Stakeholder Perspectives on Implementation Challenges and Strategies for Moving On Initiatives in Permanent Supportive Housing. J Behav Health Serv Res 2020;47:346–64. https://doi.org/10.1007/s11414-019-09680-6.

[26] Kerins C, McHugh S, McSharry J, Reardon CM, Hayes C, Perry IJ, et al. Barriers and facilitators to implementation of menu labelling interventions from a food service industry perspective: a mixed methods systematic review. Int J Behav Nutr Phys Act 2020;17:48. https://doi.org/10.1186/s12966-020-00948-1.

[27] Ashok M, Hung D, Rojas-Smith L, Halpern MT, Harrison M. Framework for Research on Implementation of Process Redesigns. Quality Management in Health Care 2018;27:17–23. https://doi.org/10.1097/QMH.0000000000000158.

[28] Yuan S, Wang F, Li X, Jia M, Tian M. Facilitators and barriers to implement the family doctor contracting services in China: findings from a qualitative study. BMJ Open 2019;9:e032444. https://doi.org/10.1136/bmjopen-2019-032444.

[29] Hohmeier KC, Wheeler JS, Turner K, Vick JS, Marchetti ML, Crain J, et al. Targeting adaptability to improve Medication Therapy Management (MTM) implementation in community pharmacy. Implementation Sci 2019;14:99. https://doi.org/10.1186/s13012-019-0946-7.

[30] Moullin JC, Sabater-Hernández D, Benrimoj SI. Qualitative study on the implementation of professional pharmacy services in Australian community pharmacies using framework analysis. BMC Health Serv Res 2016;16:439. https://doi.org/10.1186/s12913-016-1689-7.

[31] Dopp AR, Narcisse M-R, Mundey P, Silovsky JF, Smith AB, Mandell D, et al. A scoping review of strategies for financing the implementation of evidence-based practices in behavioral health systems: State of the literature and future directions. Implementation Research and Practice 2020;1:263348952093998. https://doi.org/10.1177/2633489520939980.

[32] McEachern BM, Jackson J, Yungblut S, Tomasone JR. Barriers and Facilitators to Implementing Exercise is Medicine Canada on Campus Groups. Health Promotion Practice 2019;20:751–9. https://doi.org/10.1177/1524839919830923.

[33] Miake-Lye IM, Delevan DM, Ganz DA, Mittman BS, Finley EP. Unpacking organizational readiness for change: an updated systematic review and content analysis of assessments. BMC Health Serv Res 2020;20:106. https://doi.org/10.1186/s12913-020-4926-z.

[34] Safaeinili N, Brown‐Johnson C, Shaw JG, Mahoney M, Winget M. CFIR simplified: Pragmatic application of and adaptations to the Consolidated Framework for Implementation Research (CFIR) for evaluation of a patient‐centered care transformation within a learning health system. Learn Health Sys 2020;4. https://doi.org/10.1002/lrh2.10201.

[35] Squires JE, Aloisio LD, Grimshaw JM, Bashir K, Dorrance K, Coughlin M, et al. Attributes of context relevant to healthcare professionals’ use of research evidence in clinical practice: a multi-study analysis. Implementation Sci 2019;14:52. https://doi.org/10.1186/s13012-019-0900-8.

[36] Leeman J, Baquero B, Bender M, Choy-Brown M, Ko LK, Nilsen P, et al. Advancing the use of organization theory in implementation science. Preventive Medicine 2019;129:105832. https://doi.org/10.1016/j.ypmed.2019.105832.

[37] Nieva VF, Sorra J. Safety culture assessment: a tool for improving patient safety in healthcare organizations. Qual Saf Health Care 2003;12 Suppl 2:ii17-23. https://doi.org/10.1136/qhc.12.suppl_2.ii17.

[38] Bodenheimer T, Sinsky C. From Triple to Quadruple Aim: Care of the Patient Requires Care of the Provider. The Annals of Family Medicine 2014;12:573–6. https://doi.org/10.1370/afm.1713.

[39] Harrison MI, Shortell SM. Multi‐level analysis of the learning health system: Integrating contributions from research on organizations and implementation. Learn Health Sys 2021;5. https://doi.org/10.1002/lrh2.10226.

[40] Institute of Medicine (IOM). Best Care at Lower Cost: The Path to Continuously Learning Health Care in America. Washington, D.C.: National Academies Press; 2013. https://doi.org/10.17226/13444.

[41] Breimaier HE, Heckemann B, Halfens RJG, Lohrmann C. The Consolidated Framework for Implementation Research (CFIR): a useful theoretical framework for guiding and evaluating a guideline implementation process in a hospital-based nursing practice. BMC Nurs 2015;14:43. https://doi.org/10.1186/s12912-015-0088-4.

[42] Edmondson AC. Teaming: How organizations learn, innovate, and compete in the knowledge economy. Jossey-Bass; 2012.

[43] Lapré MA, Nembhard IM. Inside the Organizational Learning Curve: Understanding the Organizational Learning Process. Foundations and Trends® in Technology, Information and Operations Management 2011;4:1–103. https://doi.org/10.1561/0200000023.

[44] Powell BJ, Mettert KD, Dorsey CN, Weiner BJ, Stanick CF, Lengnick-Hall R, et al. Measures of organizational culture, organizational climate, and implementation climate in behavioral health: A systematic review. Implementation Research and Practice 2021;2:263348952110188. https://doi.org/10.1177/26334895211018862.

[45] Reilly KL, Kennedy S, Porter G, Estabrooks P. Comparing, Contrasting, and Integrating Dissemination and Implementation Outcomes Included in the RE-AIM and Implementation Outcomes Frameworks. Front Public Health 2020;8:430. https://doi.org/10.3389/fpubh.2020.00430.

[46] Balliet D, Mulder LB, Van Lange PAM. Reward, punishment, and cooperation: A meta-analysis. Psychological Bulletin 2011;137:594–615. https://doi.org/10.1037/a0023489.

[47] Clark PB, Wilson JQ. Incentive Systems: A Theory of Organizations. Administrative Science Quarterly 1961;6:129. https://doi.org/10.2307/2390752.

[48] VanDeusen Lukas C, Holmes SK, Cohen AB, Restuccia J, Cramer IE, Shwartz M, et al. Transformational change in health care systems: an organizational model. Health Care Manage Rev 2007;32:309–20. https://doi.org/10.1097/01.HMR.0000296785.29718.5d.

[49] Weiner BJ, Mettert KD, Dorsey CN, Nolen EA, Stanick C, Powell BJ, et al. Measuring readiness for implementation: A systematic review of measures’ psychometric and pragmatic properties. Implementation Research and Practice 2020;1:263348952093389. https://doi.org/10.1177/2633489520933896.

[50] Varsi C, Ekstedt M, Gammon D, Ruland CM. Using the Consolidated Framework for Implementation Research to Identify Barriers and Facilitators for the Implementation of an Internet-Based Patient-Provider Communication Service in Five Settings: A Qualitative Study. J Med Internet Res 2015;17:e262. https://doi.org/10.2196/jmir.5091.

[51] Barwick M, Barac R, Kimber M, Akrong L, Johnson SN, Cunningham CE, et al. Advancing implementation frameworks with a mixed methods case study in child behavioral health. Translational Behavioral Medicine 2020;10:685–704. https://doi.org/10.1093/tbm/ibz005.

[52] Moretto N, Comans TA, Chang AT, O’Leary SP, Osborne S, Carter HE, et al. Implementation of simulation modelling to improve service planning in specialist orthopaedic and neurosurgical outpatient services. Implementation Sci 2019;14:78. https://doi.org/10.1186/s13012-019-0923-1.

[53] Ilott I, Gerrish K, Booth A, Field B. Testing the Consolidated Framework for Implementation Research on health care innovations from South Yorkshire: Testing the CFIR on health care innovations. J Eval Clin Pract 2012:n/a-n/a. https://doi.org/10.1111/j.1365-2753.2012.01876.x.

[54] Ritchie MJ, Parker LE, Kirchner JE. From novice to expert: a qualitative study of implementation facilitation skills. Implement Sci Commun 2020;1:25. https://doi.org/10.1186/s43058-020-00006-8.

[55] Solberg LI, Kuzel A, Parchman ML, Shelley DR, Dickinson WP, Walunas TL, et al. A Taxonomy for External Support for Practice Transformation. J Am Board Fam Med 2021;34:32–9. https://doi.org/10.3122/jabfm.2021.01.200225.

[56] Miech EJ, Rattray NA, Flanagan ME, Damschroder L, Schmid AA, Damush TM. Inside help: An integrative review of champions in healthcare-related implementation. SAGE Open Medicine 2018;6:205031211877326. https://doi.org/10.1177/2050312118773261.

[57] Godbee K, Gunn J, Lautenschlager NT, Palmer VJ. Refined conceptual model for implementing dementia risk reduction: incorporating perspectives from Australian general practice. Aust J Prim Health 2020;26:247. https://doi.org/10.1071/PY19249.

[58] Michie S, van Stralen MM, West R. The behaviour change wheel: A new method for characterising and designing behaviour change interventions. Implement Sci 2011;6:42. https://doi.org/10.1186/1748-5908-6-42.

[59] Cane J, O’Connor D, Michie S. Validation of the theoretical domains framework for use in behaviour change and implementation research. Implementation Sci 2012;7:37. https://doi.org/10.1186/1748-5908-7-37.

[60] Michie S, Johnston M, Abraham C, Lawton R, Parker D, Walker A, et al. Making psychological theory useful for implementing evidence based practice: a consensus approach. Qual Saf Health Care 2005;14:26–33. https://doi.org/10.1136/qshc.2004.011155.

[61] Ajzen I. The theory of planned behaviour: Reactions and reflections. Psychology & Health 2011;26:1113–27. https://doi.org/10.1080/08870446.2011.613995.

[62] Stokols D. Translating Social Ecological Theory into Guidelines for Community Health Promotion. Am J Health Promot 1996;10:282–98. https://doi.org/10.4278/0890-1171-10.4.282.

[63] Metz A, Louison L, Burke K, Ward C. Implementation Support Practitioner Profile. National Implementation Research Network; 2020.

[64] Albers B, Metz A, Burke K. Implementation support practitioners – a proposal for consolidating a diverse evidence base. BMC Health Serv Res 2020;20:368. https://doi.org/10.1186/s12913-020-05145-1.

[65] Barron M, Barron A. Project Management Areas of Expertise. Project Management, n.d.

[66] Müller R, Turner R. Leadership competency profiles of successful project managers. International Journal of Project Management 2010;28:437–48. https://doi.org/10.1016/j.ijproman.2009.09.003.

[67] Nilsen P. Making sense of implementation theories, models and frameworks. Implementation Science 2015;10:53.

[68] Waltz TJ, Powell BJ, Matthieu MM, Damschroder LJ, Chinman MJ, Smith JL, et al. Use of concept mapping to characterize relationships among implementation strategies and assess their feasibility and importance: results from the Expert Recommendations for Implementing Change (ERIC) study. Implementation Sci 2015;10:109. https://doi.org/10.1186/s13012-015-0295-0.

[69] Hill JN, Locatelli SM, Bokhour BG, Fix GM, Solomon J, Mueller N, et al. Evaluating broad-scale system change using the Consolidated Framework for Implementation Research: challenges and strategies to overcome them. BMC Res Notes 2018;11:560. https://doi.org/10.1186/s13104-018-3650-9.

[70] Wells R, Breckenridge ED, Linder SH. Wellness project implementation within Houston’s Faith and Diabetes initiative: a mixed methods study. BMC Public Health 2020;20:1050. https://doi.org/10.1186/s12889-020-09167-6.
