# Supplementary material for: The updated Consolidated Framework for Implementation Research based on user feedback
Source: Implement Sci. 2022 Oct 29;17:75. doi: 10.1186/s13012-022-01245-0 (PMC9617234; doi:10.1186/s13012-022-01245-0)
Supplement: Supplementary file 6 — Additional file 6. Updated CFIR Domains and Constructs: Short Definitions and Detailed Descriptions. [file 13012_2022_1245_MOESM6_ESM.docx]

# Additional File 6: Updated CFIR Domains and Constructs: Short Definitions and Detailed Descriptions

This additional file provides the short definitions and detailed descriptions for each domain and construct in the updated CFIR. In addition to reviewing the main manuscript and other additional files, CFIR users are strongly encouraged to be familiar with the following source articles and resources:

1. [*Fostering implementation of health services research findings into practice: a consolidated framework for advancing implementation science*](https://doi.org/10.1186/1748-5908-4-50): This is the original CFIR manuscript (Damschroder, Aron, et al., 2009)
2. [*Conceptualizing outcomes for use with the Consolidated Framework for Implementation Research (CFIR): the CFIR Outcomes Addendum*](https://doi.org/10.1186/s13012-021-01181-5)*:* This is the CFIR Outcomes Addendum, which conceptualizes the types of outcomes for use with the CFIR (Damschroder et al., 2022)
3. [*CFIRguide.org*](https://cfirguide.org/): This is the CFIR technical assistance website, which includes additional resources, and will be updated to reflect the updated CFIR

This file compiles information from the original CFIR (the main manuscript and its Additional File 4) with new information from the updated CFIR.

# Updated CFIR Short Domain and Construct Definitions

Note: This table is from the main manuscript and is included in this document for convenience.

| ***Framework Guidance:***  The CFIR is intended to be used to collect data from individuals who have power and/or influence over implementation outcomes. See the CFIR Outcomes Addendum for guidance on identifying these individuals and selecting outcomes (19).  The CFIR must be fully operationalized prior to use in a project:  1) Define the subject of each domain for the project (see guidance for each domain below).  2) Replace broad construct language with project-specific language if needed.  3) Add constructs to capture salient themes not included in the updated CFIR. | |
| --- | --- |
| **I. INNOVATION DOMAIN**  ***Innovation:*** The “thing” being implemented (20), e.g., a new clinical treatment, educational program, or city service.  ***Project Innovation:*** [Document the innovation being implemented, e.g., innovation type, innovation core vs. adaptable components, using a published reporting guideline (21–24). Distinguish the innovation (the “thing” that continues when implementation is complete) (20,25) from the implementation process and strategies used to implement the innovation (26,27) (activities that end after implementation is complete) (28).] | |
| **Construct Name** | **Construct Definition** *The degree to which:* |
| A. Innovation Source | The group that developed and/or visibly sponsored use of the innovation is reputable, credible, and/or trustable. |
| B. Innovation Evidence-Base | The innovation has robust evidence supporting its effectiveness. |
| C. Innovation Relative Advantage | The innovation is better than other available innovations or current practice. |
| D. Innovation Adaptability | The innovation can be modified, tailored, or refined to fit local context or needs. |
| E. Innovation Trialability | The innovation can be tested or piloted on a small scale and undone. |
| F. Innovation Complexity | The innovation is complicated, which may be reflected by its scope and/or the nature and number of connections and steps. |
| G. Innovation Design | The innovation is well designed and packaged, including how it is assembled, bundled, and presented. |
| H. Innovation Cost | The innovation purchase and operating costs are affordable. |
| **II. OUTER SETTING DOMAIN**  ***Outer Setting:*** The setting in which the Inner Setting exists, e.g., hospital system, school district, state. There may be multiple Outer Settings and/or multiple levels within the Outer Setting (e.g., community, system, state).   ***Project Outer Setting(s):*** [Document the actual Outer Setting in the project, e.g., type, location, and the boundary between the Outer Setting and the Inner Setting.] | |
| **Construct Name** | **Construct Definition** *The degree to which:* |
| A. Critical Incidents | Large-scale and/or unanticipated events disrupt implementation and/or delivery of the innovation. |
| B. Local Attitudes | Sociocultural values (e.g., shared responsibility in helping recipients) and beliefs (e.g., convictions about the worthiness of recipients) encourage the Outer Setting to support implementation and/or delivery of the innovation. |
| C. Local Conditions | Economic, environmental, political, and/or technological conditions enable the Outer Setting to support implementation and/or delivery of the innovation. |
| D. Partnerships & Connections | The Inner Setting is networked with external entities, including referral networks, academic affiliations, and professional organization networks. |
| E. Policies & Laws | Legislation, regulations, professional group guidelines and recommendations, or accreditation standards support implementation and/or delivery of the innovation. |
| F. Financing | Funding from external entities (e.g., grants, reimbursement) is available to implement and/or deliver the innovation. |
| G. External Pressure | External pressures drive implementation and/or delivery of the innovation.  *Use this construct to capture themes related to External Pressures that are not included in the subconstructs below.* |
| 1. Societal Pressure | Mass media campaigns, advocacy groups, or social movements or protests drive implementation and/or delivery of the innovation. |
| 2. Market Pressure | Competing with and/or imitating peer entities drives implementation and/or delivery of the innovation. |
| 3. Performance-Measurement Pressure | Quality or benchmarking metrics or established service goals drive implementation and/or delivery of the innovation. |
| **III. INNER SETTING DOMAIN**  ***Inner Setting:*** The setting in which the innovation is implemented, e.g., hospital, school, city. There may be multiple Inner Settings and/or multiple levels within the Inner Setting, e.g., unit, classroom, team.   ***Project Inner Setting(s):*** [Document the actual Inner Setting in the project, e.g., type, location, and the boundary between the Outer Setting and the Inner Setting.] | |
| **Construct Name** | **Construct Definition** *The degree to which:* |
| *Note:* | *Constructs A – D exist in the Inner Setting regardless of implementation and/or delivery of the innovation, i.e., they are persistent general characteristics of the Inner Setting.* |
| A. Structural Characteristics | Infrastructure components support functional performance of the Inner Setting.  *Use this construct to capture themes related to Structural Characteristics that are not included in the subconstructs below.* |
| 1. Physical Infrastructure | Layout and configuration of space and other tangible material features support functional performance of the Inner Setting. |
| 2. Information Technology Infrastructure | Technological systems for tele-communication, electronic documentation, and data storage, management, reporting, and analysis support functional performance of the Inner Setting. |
| 3. Work Infrastructure | Organization of tasks and responsibilities within and between individuals and teams, and general staffing levels, support functional performance of the Inner Setting. |
| B. Relational Connections | There are high quality formal and informal relationships, networks, and teams within and across Inner Setting boundaries (e.g., structural, professional). |
| C. Communications | There are high quality formal and informal information sharing practices within and across Inner Setting boundaries (e.g., structural, professional). |
| D. Culture | There are shared values, beliefs, and norms across the Inner Setting.  *Use this construct to capture themes related to Culture that are not included in the subconstructs below.* |
| 1. Human Equality-Centeredness | There are shared values, beliefs, and norms about the inherent equal worth and value of all human beings. |
| 2. Recipient-Centeredness | There are shared values, beliefs, and norms around caring, supporting, and addressing the needs and welfare of recipients. |
| 3. Deliverer-Centeredness | There are shared values, beliefs, and norms around caring, supporting, and addressing the needs and welfare of deliverers. |
| 4. Learning-Centeredness | There are shared values, beliefs, and norms around psychological safety, continual improvement, and using data to inform practice. |
| *Note:* | *Constructs E – K are specific to the implementation and/or delivery of the innovation****.*** |
| E. Tension for Change | The current situation is intolerable and needs to change. |
| F. Compatibility | The innovation fits with workflows, systems, and processes. |
| G. Relative Priority | Implementing and delivering the innovation is important compared to other initiatives. |
| H. Incentive Systems | Tangible and/or intangible incentives and rewards and/or disincentives and punishments support implementation and delivery of the innovation. |
| I. Mission Alignment | Implementing and delivering the innovation is in line with the overarching commitment, purpose, or goals in the Inner Setting. |
| J. Available Resources | Resources are available to implement and deliver the innovation.  *Use this construct to capture themes related to Available Resources that are not included in the subconstructs below.* |
| 1. Funding | Funding is available to implement and deliver the innovation. |
| 2. Space | Physical space is available to implement and deliver the innovation. |
| 3. Materials & Equipment | Supplies are available to implement and deliver the innovation. |
| K. Access to Knowledge & Information | Guidance and/or training is accessible to implement and deliver the innovation. |
| **IV. INDIVIDUALS DOMAIN**  ***Individuals:*** The roles and characteristics of individuals. | |
| **ROLES SUBDOMAIN**  ***Project Roles:*** [Document the roles applicable to the project and their location in the Inner or Outer Setting.] | |
| **Construct Name** | **Construct Definition** |
| A. High-level Leaders | Individuals with a high level of authority, including key decision-makers, executive leaders, or directors. |
| B. Mid-level Leaders | Individuals with a moderate level of authority, including leaders supervised by a high-level leader and who supervise others. |
| C. Opinion Leaders | Individuals with informal influence on the attitudes and behaviors of others. |
| D. Implementation Facilitators | Individuals with subject matter expertise who assist, coach, or support implementation. |
| E. Implementation Leads | Individuals who lead efforts to implement the innovation. |
| F. Implementation Team Members | Individuals who collaborate with and support the Implementation Leads to implement the innovation, ideally including Innovation Deliverers and Recipients. |
| G. Other Implementation Support | Individuals who support the Implementation Leads and/or Implementation Team Members to implement the innovation. |
| H. Innovation Deliverers | Individuals who are directly or indirectly delivering the innovation. |
| I. Innovation Recipients | Individuals who are directly or indirectly receiving the innovation. |
| **CHARACTERISTICS SUBDOMAIN**  ***Project Characteristics:*** [Document the characteristics applicable to the roles in the project based on the COM-B system (29) or role-specific theories.] | |
| **Construct Name** | **Construct Definition:**  *The degree to which:* |
| A. Need | The individual(s) has deficits related to survival, well-being, or personal fulfillment, which will be addressed by implementation and/or delivery of the innovation. |
| B. Capability | The individual(s) has interpersonal competence, knowledge, and skills to fulfill Role. |
| C. Opportunity | The individual(s) has availability, scope, and power to fulfill Role. |
| D. Motivation | The individual(s) is committed to fulfilling Role. |
| **V. IMPLEMENTATION PROCESS DOMAIN**  ***Implementation Process:*** The activities and strategies used to implement the innovation.  ***Project Implementation Process:*** [Document the implementation process framework (8) and/or activities and strategies (26,27) being used to implement the innovation. Distinguish the implementation process used to implement the innovation (activities that end after implementation is complete) from the innovation (the “thing” that continues when implementation is complete) (20,25,28).] | |
| **Construct Name** | **Construct Definition:** *The degree to which individuals:* |
| A. Teaming | Join together, intentionally coordinating and collaborating on interdependent tasks, to implement the innovation. |
| B. Assessing Needs | Collect information about priorities, preferences, and needs of people.  *Use this construct to capture themes related to Assessing Needs that are not included in the subconstructs below.* |
| 1. Innovation Deliverers | Collect information about the priorities, preferences, and needs of deliverers to guide implementation and delivery of the innovation. |
| 2. Innovation Recipients | Collect information about the priorities, preferences, and needs of recipients to guide implementation and delivery of the innovation. |
| C. Assessing Context | Collect information to identify and appraise barriers and facilitators to implementation and delivery of the innovation. |
| D. Planning | Identify roles and responsibilities, outline specific steps and milestones, and define goals and measures for implementation success in advance. |
| E. Tailoring Strategies | Choose and operationalize implementation strategies to address barriers, leverage facilitators, and fit context. |
| F. Engaging | Attract and encourage participation in implementation and/or the innovation.  *Use this construct to capture themes related to Engaging that are not included in the subconstructs below.* |
| 1. Innovation Deliverers | Attract and encourage deliverers to serve on the implementation team and/or to deliver the innovation. |
| 2. Innovation Recipients | Attract and encourage recipients to serve on the implementation team and/or participate in the innovation. |
| G. Doing | Implement in small steps, tests, or cycles of change to trial and cumulatively optimize delivery of the innovation. |
| H. Reflecting & Evaluating | Collect and discuss quantitative and qualitative information about the success of implementation.  *Use this construct to capture themes related to Reflecting & Evaluating that are not included in the subconstructs below.* |
| 1. Implementation | Collect and discuss quantitative and qualitive information about the success of implementation. |
| 2. Innovation | Collect and discuss quantitative and qualitative information about the success of the innovation. |
| I. Adapting | Modify the innovation and/or the Inner Setting for optimal fit and integration into work processes. |

# Updated CFIR Detailed Domain and Construct Descriptions

## INNOVATION DOMAIN

***Innovation:*** *The “thing” being implemented* (Curran, 2020)*, e.g., a new clinical treatment, educational program, or city service.*

The original CFIR elaborated on this domain, stating that innovations usually come to a setting as a poor fit, requiring an active process to adapt the innovation and engage individuals to accomplish implementation. The innovation is often complex and multi-faceted, with many interacting components (Butler et al., 2017). Innovations can be conceptualized as having ‘core components’ (the essential and indispensable elements of the innovation) and an ‘adaptable periphery’ (adaptable elements, structures, and systems related to the innovation and setting into which it is being implemented) (Fixsen, 2007; Greenhalgh, Robert, et al., 2004). For example, a clinical reminder to screen for obesity has an alert that pops up on the computer screen at the appropriate time for the appropriate patient. This feature is part of the core of the innovation. However, depending on the work processes at individual clinics, the clinical reminder may pop up during the patient assessment by a nurse manager or during the visit with the primary care provider. This feature is part of the adaptable periphery of the innovation; these components can be modified to a particular setting and vice versa in a co-evolving and co-adaptive way (Kirsh et al., 2008; Plsek & Greenhalgh, 2001).

### Innovation Source

*The group that developed and/or visibly sponsored use of the innovation is reputable, credible, and/or trustable.*

An innovation may be internally developed as a good idea, solution to a problem, or other grass-roots effort, or may be developed by an external entity (e.g., vendor or research group) (Greenhalgh, Robert, et al., 2004). However, whether the source is internal or external does not, in and of itself, determine implementation outcomes; it is the legitimacy or trustworthiness of the source that is an important implementation determinant (Damschroder, Aron, et al., 2009). This was highlighted by Ho et al. who found that in “resource-poor settings” an external source may “demonstrate the intervention value and increase likelihood of future local investment” (Ho et al., 2019).

The original CFIR elaborated further on this construct, stating that an externally developed innovation coupled with lack of transparency in the decision-making process may undermine legitimacy and lead to implementation failure (A. Kitson et al., 1998; Rycroft-Malone, Kitson, et al., 2002). Dissemination, “whose main mechanism of spread is centrally driven and controlled,” is negatively associated with implementation (Greenhalgh, Robert, et al., 2004), page 604; emphasis added). Though there is empirical evidence of a positive association with an authoritative decision to use the innovation, there is a negative relationship with fully implementing or routinizing the innovation (Greenhalgh, Robert, et al., 2004). If the decision to adopt and implement is made by leaders higher in the hierarchy who edict change with little user input in the decision to implement an innovation, implementation is less likely to be effective (Helfrich, Weiner, et al., 2007; Klein et al., 2001). In effect, how the Innovation Source engages individuals that will be implementing, delivering, and/or receiving the innovation, influences implementation outcomes (see Implementation Process: Engaging).

### Innovation Evidence-Base

*The innovation has robust evidence supporting its effectiveness.*

Sources of evidence may be internal or external and include published literature, guidelines, anecdotal stories from colleagues, information from a competitor, previous experiences with recipients, results from a local pilot, and other sources (Harvey & Kitson, 2015; Rycroft-Malone, Harvey, et al., 2002; Stetler, 2001).

The original CFIR elaborated further on this construct, stating that though there is no agreed upon measure of “strong evidence,” there is empirical support for a positive association with dissemination, though evidence is mixed (Dopson et al., 2010). Though strong evidence is important, it is not always dominant in individual decisions to adopt nor is it ever sufficient (L. Fitzgerald & Dopson, 2006). The PARiHS model lists three sources of evidence as being key for uptake: research studies, clinical experience, and previous patient experience (Rycroft-Malone, Harvey, et al., 2002), and Stetler adds the possibility of other sources that appear to be credible (Stetler, 2001). Externally and internally generated evidence, including experience through piloting (see Implementation Process: Doing), may be combined to build a case for implementing an innovation (Stetler, 2001). The more sources of evidence used, the more likely innovations will be taken up (A. Kitson et al., 1998; Rycroft-Malone, Kitson, et al., 2002).

Regarding quantitative measurement of this construct: In a systematic review of quantitative measures related to implementation, Lewis et al. identified one measure (Lewis, Mettert, & Lyon, 2021). Using PAPERS measurement quality criteria with an aggregate scale ranging from -9 to +36 (Lewis, Mettert, Stanick, et al., 2021), the single measure had a score of 2, indicating the need for continued development of high-quality measures.

### Innovation Relative Advantage

*The innovation is better than other available innovations or current practice.*

Alternatives to the innovation can include the status quo, e.g., the practice, treatment, or program that the innovation supersedes (E. Rogers, 2003). The original CFIR elaborated further on this construct, stating that relative advantage must be recognized and acknowledged by all individuals for effective implementation (Greenhalgh, Robert, et al., 2004). If users perceive a clear, unambiguous advantage in effectiveness or efficiency of the innovation, it is more likely the implementation will be successful. In fact, relative advantage is sine qua non for adoption and implementation (Greenhalgh, Robert, et al., 2004).

Benefits of the innovation must be clearly visible (observable) to assess relative advantage; efforts to demonstrate benefits of the innovation clearly will help implementation (Denis et al., 2002; Dopson et al., 2010; Greenhalgh, Robert, et al., 2004; R. P. Grol et al., 2007; Meyer & Goes, 1988). Although Greenhalgh et al., the PRISM model, and Grol et al.’s implementation model include observability as a separate construct (Feldstein & Glasgow, 2008; Greenhalgh, Robert, et al., 2004; R. P. Grol et al., 2007), observability (or visibility) of benefits is tightly coupled with relative advantage and it is challenging to measure them separately in the real world. As a result, we consolidated the two factors.

The extent to which the innovation is codifiable may also influence relative advantage; many innovations contain significant tacit components and may have significant benefits that are more difficult to understand or discern (Berta & Baker, 2004; Tucker et al., 2007) and thus evaluate for relative advantage.

Regarding quantitative measurement of this construct: In a systematic review of quantitative measures related to implementation, Lewis et al. identified nine measures (Lewis, Mettert, & Lyon, 2021). Using PAPERS measurement quality criteria with an aggregate scale ranging from -9 to +36 (Lewis, Mettert, Stanick, et al., 2021), the highest score was 4, indicating the need for continued development of high-quality measures.

### Innovation Adaptability

*The innovation can be modified, tailored, or refined to fit local context or needs.*

Adaptability relies on a definition of the core components (elements that cannot be changed) versus the adaptable periphery (elements that can be changed) of the innovation itself (Fixsen, 2007; Greenhalgh, Robert, et al., 2004). A component analysis can be performed to identify the core components versus adaptable periphery (Carroll et al., 2007), but often the distinction is one that can only be discerned through trial and error as the innovation is disseminated more widely and adapted for a variety of contexts (Mendel et al., 2008). The tension between the need to achieve full and consistent implementation across multiple contexts while providing flexibility to adapt the innovation as needed is real and must be balanced, which is no small challenge (Perrin et al., 2006; von Thiele Schwarz et al., 2019).

The original CFIR elaborated further on this construct, stating that information about the hard core and soft periphery can be used to assess “fidelity” as an implementation outcome (Denis et al., 2002). The hard core may be defined by a research protocol or “black-box” packaging, while the soft periphery consists of factors that vary from setting to setting. For example, a computerized report system may have a hard core that users cannot change but these core components might be accessed from different launch points, depending on workflows of local settings. Greenhalgh et al. describe aspects of adaptability under “fuzzy boundaries” and “potential for reinvention” (Greenhalgh, Robert, et al., 2004 p 596-597). An innovation that can be easily modified to adapt to the setting is positively associated with implementation (Gustafson et al., 2003; Leeman et al., 2007; E. Rogers, 2003).

Regarding quantitative measurement of this construct: In a systematic review of quantitative measures related to implementation, Lewis et al. identified five measures (Lewis, Mettert, & Lyon, 2021). Using PAPERS measurement quality criteria with an aggregate scale ranging from -9 to +36 (Lewis, Mettert, Stanick, et al., 2021), the highest score was 5, indicating the need for continued development of high-quality measures.

### Innovation Trialability

*The innovation can be tested or piloted on a small scale and undone.*

The original CFIR elaborated further on this construct, stating that the ability to test the innovation on a small scale, (Greenhalgh, Robert, et al., 2004), and be able to reverse course (undo implementation) if warranted (Feldstein & Glasgow, 2008), are important potential determinants of implementation outcomes. The ability to trial is a key feature of the plan-do-study-act quality improvement cycle that allows users to find ways to increase coordination to manage interdependence (Leeman et al., 2007; Rabin et al., 2008). Piloting allows for individuals and groups to build experience and time to reflect upon and test the innovation (Rycroft-Malone, Kitson, et al., 2002). Usability testing (with deliverers and recipients) promotes successful adaptation of the innovation (Feldstein & Glasgow, 2008) (See Implementation Process: Engaging and Doing.)

Regarding quantitative measurement of this construct: In a systematic review of quantitative measures related to implementation, Lewis et al. identified six measures (Lewis, Mettert, & Lyon, 2021). Using PAPERS measurement quality criteria with an aggregate scale ranging from -9 to +36 (Lewis, Mettert, Stanick, et al., 2021), the highest score was 3, indicating the need for continued development of high-quality measures.

### Innovation Complexity

*The innovation is complicated, which may be reflected by its scope and/or the nature and number of connections and steps.*

Complexity may be related to length (the number of sequential sub-processes or steps for using an innovation) and breadth (the number of choices presented at decision points) (Kochevar & Yano, 2006). Complexity is increased with higher numbers of potential target units (teams, clinics, departments) or types of people in the Inner Setting (Kochevar & Yano, 2006). The updated definition of complexity aligns with other published conceptualizations of complex innovations (Butler et al., 2017; Lewin et al., 2017; Moecker et al., 2021).

The original CFIR elaborated further on this construct, stating that appropriately diagnosing and assessing complexity is thought to benefit implementation by avoiding unintended consequences (Kochevar & Yano, 2006). Simple innovations are more likely to be effective (Greenhalgh, Robert, et al., 2004; Gustafson et al., 2003) because they increase user satisfaction and the speed required to be competent in using the innovations (Klein et al., 2001).

The type of innovation, whether a technical (e.g., a new computer module) or administrative (behavioral change) change, can contribute to perceptions of complexity. Technical innovations may include a purchased product, packaged service, or an automated production process (e.g., computerized order entry). Administrative innovations primarily affect social structures or processes within settings. Most innovations are a hybrid of both. Technical innovations tend to be more tangible and administrative innovations tend to be more complex and difficult to implement (Greenhalgh, Robert, et al., 2004). On the other hand, complex behavioral change innovations can generate heightened commitment when they are viewed as a welcomed fundamental change compared to settings that regard the innovation as a simple “plug-in” (Edmondson et al., 2001). Edmondson et al. describe a "technological frame" of thinking that influences implementation effectiveness. In their study of a new cardiac surgical approach involving behavior change and teaming, the sites with less successful implementation viewed the innovation as an (oversimplified) “plug-in technology” while those with better implementation effectiveness regarded the innovation "as fundamental change for the [operating] team," (Edmondson et al., 2001) despite its complexity.

Regarding quantitative measurement of this construct: In a systematic review of quantitative measures related to implementation, Lewis et al. identified nine measures (Lewis, Mettert, & Lyon, 2021). Using PAPERS measurement quality criteria with an aggregate scale ranging from -9 to +36 (Lewis, Mettert, Stanick, et al., 2021), the highest score was 4, indicating the need for continued development of high-quality measures.

### Innovation Design

*The innovation is well designed and packaged, including how it is assembled, bundled, and presented.*

The original CFIR elaborated further on this construct, stating that how an innovation is designed, including how it is assembled, bundled, and presented, can have an important effect on implementation outcomes (Klein et al., 2001). Innovation design includes how well the components of the innovation are defined, the effectiveness of branding (Evans & Hastings, 2008), and the quality of the materials associated with the innovation, e.g., marketing and training materials. This construct was not included in Greenhalgh et al.’s model (Greenhalgh, Robert, et al., 2004), but is included in Grol and Wensing’s list of innovation characteristics (R. P. Grol et al., 2007). When innovation components are designed to be easily accessible to users, it promotes use of the new procedures (Graham & Logan, 2004). An unreliable or poorly designed innovation will undermine success (Klein et al., 2001). When innovation quality is perceived to be poor by users, there are negative consequences for satisfaction and innovation use (Helfrich, Weiner, et al., 2007; Klein et al., 2001). More recent literature highlights that innovations that incorporate user-centered design principles, i.e., that were developed with user involvement, may be more effective than other innovations (Dopp et al., 2019; Greenhalgh et al., 2016).

Regarding quantitative measurement of this construct: In a systematic review of quantitative measures related to implementation, Lewis et al. identified two measures (Lewis, Mettert, & Lyon, 2021). Using PAPERS measurement quality criteria with an aggregate scale ranging from -9 to +36 (Lewis, Mettert, Stanick, et al., 2021), the highest score was 3, indicating the need for continued development of high-quality measures.

### Innovation Cost

*The innovation purchase and operating costs are affordable.*

Innovation costs, including transaction costs (Leeman et al., 2019), can be a significant barrier to implementation if they are perceived to be too expensive or unaffordable. The original CFIR elaborated further on this construct, stating that the cost to purchase, subscribe, or use the innovation, including training costs associated with the innovation, is an important adoption and implementation determinant (Graham & Logan, 2004; Teplensky et al., 1995).

## OUTER SETTING DOMAIN

***Outer Setting:*** *The setting in which the Inner Setting exists, e.g., hospital system, school district, state.* *There may be multiple Outer Settings and/or multiple levels within the Outer Setting (e.g., community, system, state).*

Lengnick-Hall et al. call for taking an “open-systems” perspective when conceptualizing boundaries between Outer and Inner Settings “to highlight interdependence between outer and inner contexts and [to] view organizations as part of a broader interdependent system that may range from simple to complex, rigid to flexible, and loosely to tightly coupled” (Lengnick-Hall et al., 2020 p3). Although embracing an open-systems perspective may be challenging, conceptually differentiating internal and external influences on the performance of organizations has been a central tenet of organization science (Katz & Kahn, 1966), and highlights the level at which to focus interventions.

The original CFIR elaborated further on this domain, stating that the Outer Setting is designed to capture macro-level factors that emanate from outside the Inner Setting. The specific factors considered ‘in’ or ‘out’ will depend on the context of the implementation effort (Damschroder, Aron, et al., 2009). For example, outlying clinics may be part of the Outer Setting in one study, but part of the Inner Setting in another study. A social-ecological perspective that recognizes the complex interplay between macro-level factors (e.g., community, economic), meso-level factors (e.g., health systems, clinics), and microsystem-level factors (e.g., units, teams), with individuals is widely applied within implementation. Changes in the Outer Setting can have positive or negative influences on implementation; these constructs are boundary spanning because they are mediated through changes in the Inner Setting (Institute of Medicine, 2001).

### Critical Incidents

*Large-scale and/or unanticipated events disrupt implementation and/or delivery of the innovation.*

Large-scale and/or unanticipated events can disrupt implementation and/or delivery of the innovation (Rosenthal et al., 2021; e.g., Wensing et al., 2020) and may include pandemics, weather-related disasters, or political disruptions.

### Local Attitudes

*Sociocultural values (e.g., shared responsibility in helping recipients) and beliefs (e.g., convictions about the worthiness of recipients) encourage the Outer Setting to support implementation and/or delivery of the innovation.*

Attitudes in the local community can encourage the Outer Setting to support implementation and/or delivery of the innovation (Dy et al., 2015; Means et al., 2020). Local attitudes can be related to values (Merlo et al., 2019) (e.g., equity) and beliefs (e.g., white supremacy, racial bias) (Tiderington et al., 2020); examples include widespread attitudes about organ donation (Squires et al., 2019) and bias against tenants with housing choice vouchers (Tiderington et al., 2020). While not all innovations require support from the Outer Setting, these themes are important when the innovation is community-based or relies on the community for support or resources; with these innovations, local attitudes may influence implementation outcomes and equity in implementation.

### Local Conditions

*Economic, environmental, political, and/or technological conditions enable the Outer Setting to support implementation and/or delivery of the innovation.*

Conditions of the local community can enable the Outer Setting to support implementation and/or delivery of the innovation (Dy et al., 2015; Means et al., 2020). Conditions can include economic (e.g., recession) (Kerins et al., 2020), environmental (e.g., built environment), political (e.g., instability or corruption) (Flottorp et al., 2013), and/or technological (e.g., IT infrastructure) factors (Ashok et al., 2018; Dy et al., 2015; Means et al., 2020; Yuan et al., 2019). While not all innovations require support from the Outer Setting, these themes are important when the innovation is community-based or relies on the community for support or resources, especially in low- to middle- income countries, where resource constraints are common (Means et al., 2020); with these innovations, local conditions may influence implementation outcomes and equity in implementation.

### Partnerships & Connections

*The Inner Setting is networked with external entities, including referral networks, academic affiliations, and professional organization networks.*

This construct captures linkages between the Inner Setting and entities in the Outer Setting, including partnerships, collaboratives, professional societies (Aarons et al., 2011; Moullin et al., 2019), referral networks between health and social services (Flottorp et al., 2013), community-academic partnerships, advocacy groups (Moullin et al., 2019), contracts, technical assistance organizations (Aarons et al., 2011; Raghavan et al., 2008), and access to regional data warehouses or membership in systems (e.g., integrated healthcare system, school district) (Aarons et al., 2011; Nilsen & Bernhardsson, 2019).

The original CFIR elaborated further, recognizing that Inner Settings that support and promote external boundary-spanning roles are more likely to implement new practices quickly (Aiken et al., 1980; Baldridge & Burnham, 1975; Barnsley et al., 1998; Greenhalgh, Robert, et al., 2004; Kimberly & Evanisko, 1981). Professional knowledge can arise from increased boundary-spanning activities (Damanpour, 1991) and participation in professional group(s), as well as external training, are associated with implementation success (Simpson & Dansereau, 2007).

There is a negative relationship between linkages with external entities and implementation until clear advantages of the innovation become apparent (Burns & Wholey, 1993; Greenhalgh, Robert, et al., 2004). However, the relationship is positive once the innovation is accepted as the norm by others in the in/formal network (see also External Pressure) (Greenhalgh, Robert, et al., 2004).

In addition, the collective relationships of individuals in a setting with outer entities represent the social capital of the setting (Brehem & Rahn, 1997; Gittell & Vidal, 1998; Gladwell, 2006; Leana & Pil, 2006); increased bridging between the Inner Setting and the Outer Setting builds social capital (Greenhalgh, Robert, et al., 2004; Nilsen & Bernhardsson, 2019).

Regarding quantitative measurement of this construct: In a systematic review of quantitative measures related to implementation, McHugh et al. identified seven measures (McHugh et al., 2020). Using PAPERS criteria of measurement quality with an aggregate scale ranging from -9 to +36 (Lewis, Mettert, Stanick, et al., 2021), five measures (71.43%) had sufficient information for assessment and scores ranged from -2 to +6. Results indicate the need for continued development of high-quality measures.

### Policies & Laws

*Legislation, regulations, professional group guidelines and recommendations, or accreditation standards support implementation and/or delivery of the innovation.*

This construct includes externally promulgated (governmental or other external entity) policies, regulations, rules, codes, mandates, recommendations, guidelines, directives, or accreditation requirements (Aarons et al., 2011; Flottorp et al., 2013; Greenhalgh et al., 2017; Leeman et al., 2019; Mendel et al., 2008; Nilsen & Bernhardsson, 2019; Squires et al., 2019), and alignment of implementation of the innovation with these policies and laws (Means et al., 2020). This construct also includes externally generated malpractice liability (Flottorp et al., 2013) and continuing education requirements (Raghavan et al., 2008).

The original CFIR expanded more on this construct to include “political directives,” “policy push,” and “external mandates.” Greenhalgh et al. cite strong evidence of the direct effects of these concepts; they may also have indirect effects by increasing motivation (but not capacity) of settings to implement innovations (Greenhalgh, Robert, et al., 2004 p610).

### Financing

*Funding from external entities (e.g., grants, reimbursement) is available to implement and/or deliver the innovation.*

A scoping review highlights the importance of financing and recognizes a wide variety of financial strategies (Dopp et al., 2020). This construct captures payment schemes (Dy et al., 2015; Klein & Sorra, 1996), reimbursement (Greenhalgh et al., 2017; Hohmeier et al., 2019; Moullin et al., 2019; Raghavan et al., 2008), remuneration (Kirsh et al., 2008; Moullin et al., 2016), grants, and donations. It also includes overall funding constraints regionally or nationally (Aarons et al., 2011; Greenhalgh et al., 2017; Nilsen & Bernhardsson, 2019), distinct from local economic conditions (see Local Conditions). The original CFIR further elaborated by saying that reimbursement systems and other monetary incentives that are controlled outside the Inner Setting can influence implementation (R. P. Grol et al., 2007).

### External Pressure

*External pressures drive implementation and/or delivery of the innovation. Use this construct to capture themes related to External Pressures that are not included in the subconstructs below.*

There is strong direct evidence that the pressure to adopt an innovation, independent of perceptions of whether recipients need it or in response to a perceived problem, influences adoption and implementation, particularly in settings with strong external connections (see Partnerships and Connections) (Greenhalgh, Robert, et al., 2004). External pressure may emanate from multiple entities in the Outer Setting (Dy et al., 2015), including the subconstructs below.

#### 1. Societal Pressure

*Mass media campaigns, advocacy groups, or social movements or protests drive implementation and/or delivery of the innovation.*

Societal and/or mass media pressure can influence implementation and/or delivery of the innovation (Kerins et al., 2020; Pettigrew et al., 2001; Raghavan et al., 2008), as can normative pressure through, e.g., professional networks (Flottorp et al., 2013; Leeman et al., 2019). This also includes community advocacy, pressures from class action lawsuits, and consumer groups (Aarons et al., 2011; Moullin et al., 2019).

#### 2. Market Pressure

*Competing with and/or imitating peer entities drives implementation and/or delivery of the innovation.*

The original CFIR elaborated on this construct, recognizing that the need to compete with and/or imitate external peer entities can drive implementation and/or delivery of an innovation (Greenhalgh, Robert, et al., 2004); peer entities refers to any outside entity with which the Inner Setting feels some degree of affinity or competition (e.g., market competitors, other settings in the same network, a highly regarded institution). In competitive markets, Inner Settings may be more likely to implement new innovations (Frambach & Schillewaert, 2001).

The pressure to implement can be particularly strong for late adopters (Walston et al., 2001). If competitors or colleagues are all using an innovation, individuals and settings may feel compelled to do so too. This is referred to as “mimetic pressure” or “inter-organizational norm-setting” (Greenhalgh, Robert, et al., 2004). This pressure directly influences adoption decisions but can also trickle down to implementation if individuals are attuned to practices of outside entities.

#### 3. Performance-Measurement Pressure

*Quality or benchmarking metrics or established service goals drive implementation and/or delivery of the innovation.*

Performance measurement can influence implementation and/or delivery of the innovation (Mendel et al., 2008). This construct includes formal mechanisms of performance accountability, audit and feedback, outcome goals (Raghavan et al., 2008), benchmarking (Nilsen & Bernhardsson, 2019), and public reporting (Nilsen & Bernhardsson, 2019).

The original CFIR elaborated further on this construct, stating that the threat or reality of public reporting may motivate Inner Settings, especially late-adopters, to implement an innovation in an effort not to look bad compared to their competitors (see Market Pressure). However, public reporting can also have a negative influence if there is an adversarial relationship between the reporting entity and the Inner Setting. In this context, people may cover-up (compliant implementation) (Klein & Sorra, 1996) or engage in “box-checking” rather than true committed use.

## INNER SETTING DOMAIN

***Inner Setting:*** *The setting in which the innovation is implemented, e.g., hospital, school, city. There may be multiple Inner Settings and/or multiple levels within the Inner Setting, e.g., unit, classroom, team.*

The Inner Setting is often the unit of analysis in an implementation study and can be divided into multiple levels (McEachern et al., 2019) to account for teams or units (Miake-Lye et al., 2020; Safaeinili et al., 2020). For example, Safaeinili et al. adapted the CFIR to accommodate three embedded levels: 1) pilot clinics, 2) peer clinics, and 3) the larger health system (Safaeinili et al., 2020). Constructs A – D below capture persistent general characteristics of the Inner Setting (e.g., Relational Connections), while Constructs E – K are specific to implementation and/or delivery of the innovation (e.g., Access to Knowledge & Information).

The original CFIR elaborated further on this domain, stating that the Inner Setting may be composed of tightly or loosely coupled entities (e.g., a loosely affiliated medical center and outlying contracted clinics or tightly integrated service lines within a health system). The Inner Setting is an active interacting force and not just a backdrop in implementation (Sue Dopson & Louise Fitzgerald, 2006). Objective descriptions of the Inner Setting may include its size, age, maturity, and specialization (the uniqueness of the niche or market for the organization’s products or services); each of these features may influence implementation (Greenhalgh, Robert, et al., 2004). Size and age are sometimes used as proxy measures and may be negatively associated with implementation when bureaucratic structure is increased as a result (Van de Ven, 1986; Walston et al., 2001).

### Structural Characteristics

*Infrastructure components support functional performance of the Inner Setting. Use this construct to capture themes related to Structural Characteristics that are not included in the subconstructs below.*

The original CFIR provided a broad description of the role of Structural Characteristics. Much support for the role of structural characteristics comes from Damanpour’s seminal research into organizational innovation (Damanpour, 1991). Several dimensions of structural characteristics have been found to have significant associations with implementation, though many of these characteristics have had mixed effects, most likely because they interact with other features of the Inner Setting (Frambach & Schillewaert, 2001).

Regarding quantitative measurement of this construct: In a systematic review of quantitative measures related to implementation, Dorsey et al. identified 13 measures (Dorsey et al., 2021). Using PAPERS criteria of measurement quality with an aggregate scale ranging from -9 to +36 (Lewis, Mettert, Stanick, et al., 2021), four (30.77%) of the measures could not be scored and scores for the remaining measures ranged from -2 to +6. Themes within the measures included objective descriptors of the Inner Setting (e.g., age, size), physical infrastructure, and work infrastructure. Results indicate the need for continued development of high-quality measures.

#### Physical Infrastructure

*Layout and configuration of space and other tangible material features support functional performance of the Inner Setting.*

Layout and configuration of space and other tangible material features can support or hinder functional performance of the Inner Setting (Ashok et al., 2018; Dy et al., 2015).

#### 2. Information Technology Infrastructure

*Technological systems for tele-communication, electronic documentation, and data storage, management, reporting, and analysis support functional performance of the Inner Setting.*

Several published adaptations of the original CFIR and recommendations highlighted the importance of the Information Technology Infrastructure (Ashok et al., 2018; Dy et al., 2015; Yuan et al., 2019). Yuan et al. found that a uniform information system played a key role for family physicians who used it to access complete health information; while the presence of a uniform system promoted coordination between specialists and led to higher quality health management of patients, the absence of a uniform system led to duplication of work and lower efficiency (Yuan et al., 2019).

#### 3. Work Infrastructure

*Organization of tasks and responsibilities within and between individuals and teams, and general staffing levels, support functional performance of the Inner Setting.*

A multi-country analysis of contextual features by Squires et al. includes a construct specific to work structures (Squires et al., 2019), described as the “arrangement of tasks, responsibilities, and resources within and between the various teams in […] settings, and delegation of tasks among supervisors and subordinates.” This includes “the arrangement of schedules, shifts, and on-call duties, the order of work tasks and procedures, and the management of workloads (Squires et al., 2019).” General staffing (e.g., chronic understaffing) and turnover are included in this construct because of their significant effects on task allocation. General staffing levels indicate level of scarce (relatively limited) or slack (relatively abundant) resources in terms of time availability to take on new implementation. Slack resources are thought to promote absorptive capacity (a potential antecedent assessment related to implementation readiness – refer to the CFIR Outcomes Addendum for more detail (Damschroder et al., 2022)) because of the increased ability to absorb failure (Damanpour, 1991; Singh & Lumsden, 1990) or take on new initiatives. However, in a meta-analysis, slack resources were not a significant influence, perhaps because of the lack of distinction between different types of slack resources (Damanpour, 1991). This is differentiated from time allocations specifically for people to participate in implementation or delivery of the innovation (see Individuals: Characteristics: Opportunity).

The original CFIR elaborated on this by describing social architecture: how large numbers of people are clustered into smaller groups and differentiated, and how the independent actions of these differentiated groups are coordinated for functional performance (Thompson et al., 2003). When teams are stable (i.e., members remain with the team for an adequate period of time; there is low turnover), implementation is more likely to be successful (Edmondson et al., 2001). Functional differentiation is the internal division of labor where coalitions of professionals are formed into differentiated units. The number of units/departments represents diversity of knowledge in an organization. The degree of specialization (the number of different occupational types or specialties in a setting) can have a positive relationship with implementing change when the knowledge base is increased (Kimberly & Evanisko, 1981). Related to social architecture, the number of departments that participate in decision-making is positively associated with effective implementation (Aiken et al., 1980; Baldridge & Burnham, 1975; Damanpour, 1991; Greenhalgh, Robert, et al., 2004); centralization (the dispersion or concentration of decision-making autonomy) has mixed effects depending on the study (Damanpour, 1991) and the stage of innovation (initial v. implementation stage) (Dewar & Dutton, 1986). In a recent study in the Veterans Health Administration, centralized decision-making and the associated bureaucratic structures delayed implementation completion (Nevedal et al., 2020).The degree of vertical integration (the number of hierarchical levels in departments or units) has a mixed relationship with implementation (Aiken et al., 1980; Damanpour, 1991; Hull et al., 2019). Administrative intensity (the ratio of managers to total employees) has a positive relationship with implementation (Damanpour, 1991). Clear role definitions (e.g., physician and non-physician roles) positively influences implementation (Bodenheimer, 2002; Bodenheimer et al., 2002). The PARHiS framework asserts that clearly defined physical, social, cultural, structural, and system boundaries contributes to innovation uptake (A. Kitson et al., 1998; Rycroft-Malone, Kitson, et al., 2002).

### B. Relational Connections

*There are high quality formal and informal relationships, networks, and teams within and across Inner Setting boundaries (e.g., structural, professional).*

The original CFIR elaborated on this construct by recognizing that research on organizational change has moved beyond simplified measures of Inner Setting structure, and increasingly embraces the complex role that networks have on implementation of innovations (L. A. Fitzgerald & van Eijnatten, 2002). This construct includes Greenhalgh’s intraorganizational networks (Greenhalgh, Robert, et al., 2004). Connections between individuals, units, services, and hierarchies may be strong or weak, formal or informal, tangible or intangible, visible or invisible. There is much interplay between formal structure, informal networks, and communications (see Communications). Assessments may be informed by social networking (Scott, 2000), complexity (Burnes, 2005; L. A. Fitzgerald & van Eijnatten, 2002), or other theories, or a more inductive, grounded approach can be used. Social capital describes the quality and the extent of relationships and includes dimensions of shared vision and information sharing. One component of social capital is the internal bonding of individuals within the Inner Setting (Greenhalgh, Robert, et al., 2004). Complexity theory posits that relationships between individuals may be more important than individual attributes (Plsek & Wilson, 2001), and building these relationships can positively influence implementation (Safran et al., 2006).

More recent literature has acknowledged the key role of teams (Dy et al., 2015; Means et al., 2020) and how ideally, relationships evolve to build a sense of ‘teamness’ or ‘community’ that may contribute to implementation outcomes (Edmondson, 2012; Edmondson et al., 2001). The original CFIR went on to highlight teamwork as an essential “core property” for successful implementation of quality improvement initiatives (Ferlie & Shortell, 2001)(page 287). The more stable teams are (members are able to be with the team for an adequate period of time; low turnover), the more likely implementation will be successful (Edmondson et al., 2001). Teamness is a state and is an essential core property for successful implementation (Ferlie & Shortell, 2001). More recently, Edmondson has described the role of increasingly dynamic teams working in complex systems like healthcare (Edmondson, 2012) (see also, Implementation Process: Teaming).

Greenhalgh et al. cite strong or moderate influence of at least a partial role of Inner Setting networks and internal boundary spanning on implementation (Greenhalgh, Robert, et al., 2004). Strong horizontal and informal networks have a positive relationship with adoption; e.g., physicians are influenced by the spreading of information through their peers (Greenhalgh, Robert, et al., 2004). Coordination across departments and specialties is essential for effective implementation to attenuate the “complex web of sources of power and covert and overt sources of influence” that all contribute to individual decisions about whether to cooperate (Feldstein & Glasgow, 2008 p233). A core principle of complexity theories leads to the idea that the actions of individuals and units affect implementation, positively or negatively, in predicted and unpredicted ways (L. A. Fitzgerald, 2002). Integration to bridge traditional intra-organizational boundaries among individual units is one of five critical elements for driving transformation in patient care (VanDeusen Lukas et al., 2007). Physicians and nurses may struggle with routine (and role) changes that require coordination of activities and sharing of information across professions or units (Klein et al., 2001).

Regarding quantitative measurement of this construct: In a systematic review of quantitative measures related to implementation, Dorsey et al. identified 29 measures (Dorsey et al., 2021). These measures relate to both Relational Connections and Communications (see below) in the updated CFIR. Using PAPERS criteria of measurement quality with an aggregate scale ranging from -9 to +36 (Lewis, Mettert, Stanick, et al., 2021), five (17.24%) of the measures could not be scored. The Texas Christian University Organizational Readiness for Change (Lehman et al., 2002) achieved the highest score of 10. Results indicate the need for continued development of high-quality measures.

### Communications

*There are high quality formal and informal information sharing practices within and across Inner Setting boundaries (e.g., structural, professional).*

The original CFIR elaborated that regardless of how an Inner Setting is structurally organized, the importance of communication across the setting is clear. This construct includes Greenhalgh’s intraorganizational communications (Greenhalgh, Robert, et al., 2004). Greenhalgh et al. cite strong or moderate influence of intraorganizational communication on implementation outcomes (Greenhalgh, Robert, et al., 2004). Communication failures are involved with the majority of sentinel events in US hospitals – most often between physicians and nurses (Pronovost et al., 2006). High quality formal communications contribute to effective implementation (Simpson & Dansereau, 2007). Strong intra-organizational communications are positively associated with implementation as it pushes decision-making to the front-line teams or individuals (Greenhalgh, Robert, et al., 2004; Meyers et al., 1999). Making staff feel welcome (good assimilation through communication), peer collaboration, open feedback and review among peers and across hierarchical levels, clear communication of mission and goals, and informal communication quality all contribute to effective implementation (Simpson & Dansereau, 2007).

See the last paragraph for Relational Connections above, for an overview of quantitative measures related to Communications.

### D. Culture

*There are shared values, beliefs, and norms across the Inner Setting. Use this construct to capture themes related to Culture that are not included in the subconstructs below.*

The original CFIR had extensive elaboration on Culture, including a general description of culture concepts and a more in-depth description of one concept of culture, the Competing Values Framework (CVF). Broadly, culture includes the norms, values, and basic assumptions in a setting (Gershon et al., 2004). Culture is often viewed as relatively stable, socially constructed, and subconscious (Martin, 2002). However, culture is not defined consistently in the literature (Gershon et al., 2004) and many definitions exist for culture (Martin, 2002). Some researchers have a relatively narrow definition of culture, while other researchers incorporate nearly every construct related to the Inner Setting. Culture and climate can be interchangeable across studies, depending on the definition used (Martin, 2002). One review identified 54 different definitions for organizational climate (Gershon et al., 2004).

Often, change efforts are targeted at visible, objective aspects of an organization that include work tasks, structures, and processes. Despite variation in use and definition, culture has been shown (Helfrich, Li, et al., 2007; Shortell et al., 2001) or theorized (A. Kitson et al., 1998; Rycroft-Malone, Kitson, et al., 2002) to have significant influence on implementation effectiveness. One explanation for why so many implementations fail centers on the failure to change the less tangible organizational assumptions, thinking, or culture (van Eijnatten & Galen, 2002). Individuals within a setting impart organizational culture to new members, and culture influences how people within a setting relate to one another (also see Relational Connections) and their work environment (Helfrich, Li, et al., 2007).

The CVF was originally developed by Quinn and Rohrbaugh (Quinn & Rohrbaugh, 1981) and has been used in healthcare (Shortell et al., 2001) and in the VA (Helfrich, Li, et al., 2007). It is an example of a “variable definition” approach to culture: a quantitative measure that purports to capture key aspects of the complicated dynamics of culture. Often measures of culture are elicited from senior leaders in the organization – not from non-supervisors. The CVF characterizes organizations along two dimensions, each representing a basic challenge that every organization must resolve to function effectively. The first set of competing values is the degree to which an organization emphasizes central control over processes versus decentralization and flexibility (see also Structural Characteristics: Work Infrastructure. The second set of competing values is the trade-off between focus on its own internal environment and processes versus the external environment and relationships with outside entities. Four archetypical organizational cultures arise: 1) team culture (high internal focus with high flexibility (aka personal)); 2) hierarchical culture (high internal focus with high control (aka formalized and structured)); 3) entrepreneurial culture (high external focus with high flexibility (aka dynamic and entrepreneurial)); and 4) rational culture (high external focus with high control (aka production oriented)) (Helfrich, Li, et al., 2007; Shortell et al., 2001). These “archetypes” are not mutually exclusive. In one study, CVF culture was not found to be influential in the number of evidence-based practices used by healthcare organizations (Shortell et al., 2001). Formalization is negatively associated with innovation because of lack of flexibility and/or low acceptance of new ideas (Damanpour, 1991) and can foster continuance of status quo (Klein et al., 2001). A “balanced” culture with respect to the CVF (how close organizations are to 25-25-25-25% on each of the four archetypical quadrants using a Herfindahl-type measure) contributes to perceptions of team effectiveness and the number of changes implemented (though not the depth of change) (Shortell et al., 2004).

Regarding quantitative measurement of this construct: In a systematic review of quantitative measures related to implementation, Powell et al. identified 21 measures (Powell et al., 2021). Using PAPERS criteria of measurement quality with an aggregate scale ranging from -9 to +36 (Lewis, Mettert, Stanick, et al., 2021), the Organizational Social Context – Culture Scale (Glisson et al., 2008) had the highest score of 11 followed by the Organizational Description Questionnaire (Parry & Proctor-Thomson, 2001) with a score of 9. Thirty-six measures were also identified for organizational climate. Of the 31 measures that were scored, The Texas Christian University Program Training Needs Survey (Simpson, 2002) had the highest score of 13, followed closely by The Organizational Social Context—Climate (Glisson et al., 2008) with a score of 12, and The Organizational Climate Measure (Patterson et al., 2005) with a score of 9. Results indicate the need for continued development of high-quality measures.

#### 1. Human Equality-Centeredness

*There are shared values, beliefs, and norms about the inherent equal worth and value of all human beings.*

Equity must be considered in implementation work (Leeman et al., 2019; Means et al., 2020); shared values, beliefs, and norms about the inherent equal worth and value of all human beings may contribute to implementation outcomes as well as equity in implementation (Gosepath, 2021). Justice in the Inner Setting is based on perceptions of distributive and procedural fairness (Greenberg, 1990).

#### 2. Recipient-Centeredness

*There are shared values, beliefs, and norms around caring, supporting, and addressing the needs and welfare of recipients.*

Prioritizing recipient needs within the Inner Setting is important (Dy et al., 2015; Godbee et al., 2020). While the original CFIR highlighted patient centered care, the updated CFIR expands on the patient role to include any recipient who benefits from products or services generated by the Inner Setting. The original CFIR highlighted the key role of improving the health and well-being of patients as the foundational mission of all healthcare entities; many calls have gone out for settings to be more patient centered (Institute of Medicine, 2001; Kochevar & Yano, 2006; Trumbo et al., 2019; Varsi et al., 2015). Patient-centered settings are more likely to implement change effectively (Oswald et al., 2019; Shortell et al., 2004). PRISM delineates six elements that can help guide evaluation of the extent to which patients are at the center of organizational processes and decisions: patient choices are provided, patient barriers are addressed, transition between program elements is seamless, complexity and costs are minimized, and patients have high satisfaction with service and access, and receive feedback (Feldstein & Glasgow, 2008). Patient-centeredness is also at the heart of patient safety culture (Nieva & Sorra, 2003).

#### 3. Deliverer-Centeredness

*There are shared values, beliefs, and norms around caring, supporting, and addressing the needs and welfare of deliverers.*

Deliverer-Centeredness captures the importance of addressing the needs of deliverers, and aligns with the expansion of the “Triple Aim” (Berwick et al., 2008) (enhancing patient experience, improving population health, reducing costs) to the “Quadruple Aim,” which adds an aim to improve the work-life and well-being of clinicians and staff (Bodenheimer & Sinsky, 2014). There is an extensive literature on the important role of employee well-being (Bakker, 2015; Ilies et al., 2015). Physician burnout and personal-well-being have a potentially bi-directional or reinforcing relationship with capacity for change (Goldberg et al., 2021; Rotenstein & Johnson, 2020; Williams et al., 2018). An Agency for Healthcare Research and Quality innovation adoption guide includes questions about potential workplace inequities as potential barriers to implementation (Brach et al., 2008). The organizational social context measure, developed by Glisson et al., includes constructs related to psychological climate (perception of the psychological influence of work environment) as potential influences on implementation outcomes.

#### 4. Learning-Centeredness

*There are shared values, beliefs, and norms around psychological safety, continual improvement, and using data to inform practice.*

Learning culture is a prominent theory in healthcare, especially as systems press forward to become “learning systems” (Ashok et al., 2018; Dy et al., 2015; Harrison & Shortell, 2021; Institute of Medicine (IOM), 2013). Ideally, continuous learning is occurring throughout the Inner Setting with visible evidence of engaged process improvement (including historical evidence of previous change initiatives (Breimaier et al., 2015)), use of data to inform change, and the necessary relational environment (Edmondson, 2012; Lapré & Nembhard, 2011; Miake-Lye et al., 2020). Shared values, beliefs, and norms around psychological safety, continual improvement, and using data to inform practice (Guise et al., 2018; Harrison & Shortell, 2021; Institute of Medicine (IOM), 2013; Lapré & Nembhard, 2011) support and enable employee skill development through increased experience implementing innovations (Breimaier et al., 2015). This culture creates a collective learning mindset (Edmondson, 2012) and increases the setting’s absorptive capacity for new knowledge and methods (Greenhalgh, Robert, et al., 2004).

The original CFIR’s learning climate construct has been moved under Culture in the updated CFIR because of the lack of agreement on culture versus climate concepts. As with the other Culture subconstructs, Learning-Centeredness can be assessed as a broad Inner Setting culture or as a specific micro-system climate.

The original CFIR elaborated further on this construct, stating that learning-centeredness manifests through several behaviors and perceptions: a) leaders express their own fallibility and need for team member assistance and input; b) team members feel that they are essential, valued, and knowledgeable partners in implementation; c) individuals feel psychologically safe to try new methods; and d) there is sufficient time and space for reflective thinking and evaluation (Klein et al., 2001; Leeman et al., 2007; Nembhard & Edmonson, 2006).

The degree to which an organization demonstrates “learning” will vary across sub-groups, and manifestations of these attributes may have a stronger influence than a general measure of learning in the setting more broadly (Edmondson et al., 2001). The literature on team learning has emphasized the setting’s role in creating the climate to enable learning and fostering collaboration within and between cross-disciplinary teams (Edmondson, 2002) (also see Relational Connections and Process: Teaming).

However, there is no agreement on precisely how to operationalize this construct. Despite this, we can make some generalizations. In a positive learning climate, individuals are not constrained by failure and psychological safety is promoted. Psychological safety has predicted engagement in quality improvement work (Nembhard & Edmonson, 2006). Having the time and space for reflective thinking and evaluation (see also, Process: Reflecting and Evaluating) is another important characteristic, at least in part, because it promotes learning from past successes and failures to inform future implementations (Edmondson et al., 2001; Helfrich, Weiner, et al., 2007). Developing a culture that promotes learning is a “core property” that health care organizations need for on-going quality improvement (Ferlie & Shortell, 2001 p287). A learning culture is an important contributor for increasing absorptive capacity for new knowledge: the ability of an organization to fully assimilate innovations (Greenhalgh, Robert, et al., 2004). Greenhalgh et al. include learning organization culture within their concept of absorptive capacity of new knowledge along with existing knowledge and skills (tacit and explicit), and within proactive leadership (Greenhalgh, Robert, et al., 2004).

Quantitative measurement instruments are available for measuring an organization’s “learning” capability (Goh & Richards, 1997; Templeton et al., 2002). Additionally, in a systematic review of quantitative measures related to implementation, Powell et al. identified two measures for learning climate (Powell et al., 2021). Using PAPERS criteria of measurement quality with an aggregate scale ranging from -9 to +36 (Lewis, Mettert, Stanick, et al., 2021), The Ramsey Learning Climate Measure (Ramsey et al., 2015) was rated the highest with a score of 6. Results indicate the need for continued development of high-quality measures.

### E. Tension for Change

*The current situation is intolerable and needs to change.*

The original CFIR highlighted that an intolerable situation where a need for change is acutely felt can be an important determinant of implementation outcomes (Greenhalgh, Robert, et al., 2004; Meyer & Goes, 1988; Simpson & Dansereau, 2007; VanDeusen Lukas et al., 2007). An acute sense of the need for change may trigger designing an innovation internally (see Innovation Source).

Effective communication can foster tension for change by building dissatisfaction with status quo as well as announcing a change, cultivating commitment, and reducing resistance (Greenhalgh, Robert, et al., 2004). When individuals have first-hand experience with the problem, implementation is more likely to be successful (Gustafson et al., 2003). It is difficult to create a tension for change when none exists.

Regarding quantitative measurement of this construct: In a systematic review of quantitative measures related to implementation, Powell et al. identified two measures (Powell et al., 2021). Using PAPERS criteria of measurement quality with an aggregate scale ranging from -9 to +36 (Lewis, Mettert, Stanick, et al., 2021), both measures had scores of 2. Results indicate the need for continued development of high-quality measures.

### F. Compatibility

*The innovation fits with workflows, systems, and processes.*

The original CFIR elaborated by saying that this construct includes the tangible fit between the innovation and existing workflows and systems (Greenhalgh, Robert, et al., 2004; Klein & Sorra, 1996); compatibility between innovation and delivery context has a positive association with implementation (Aubert & Hamel, 2001; Greenhalgh, Robert, et al., 2004; Klein & Sorra, 1996) and is a key driver of sustained outcomes (Chambers et al., 2013; Lennox et al., 2018; von Thiele Schwarz et al., 2019).

Helfrich et al. found that perceived fit with core competencies and experience was an important predictor of successful implementation (Helfrich, Weiner, et al., 2007). In contrast, changes seen as incompatible with current conditions will be resisted (Gustafson et al., 2003). The radicalness and magnitude of difference between the innovation and status quo, i.e., the disruptiveness of the innovation, may influence implementation (Greenhalgh, Robert, et al., 2004; R. P. Grol et al., 2007); radical innovations or innovations affecting core work processes may require reorientation and produce fundamental changes in the Inner Setting, (Greenhalgh, Robert, et al., 2004; R. P. Grol et al., 2007)

Regarding quantitative measurement of this construct: In a systematic review of quantitative measures related to implementation, Powell et al. identified six measures (Powell et al., 2021). Using PAPERS criteria of measurement quality with an aggregate scale ranging from -9 to +36 (Lewis, Mettert, Stanick, et al., 2021), measures had scores ranging from -1 to 5. Results indicate the need for continued development of high-quality measures.

### G. Relative Priority

*Implementing and delivering the innovation is important compared to other initiatives.*

The original CFIR elaborated on this construct, stating that the importance of implementing and delivering the innovation relative to other initiatives can affect implementation outcomes.(Feldstein & Glasgow, 2008; Klein et al., 2001; Klein & Sorra, 1996). If employees perceive that implementation is a key organizational priority (promoted, supported, and cooperative behaviors rewarded), then implementation climate will be strong (Klein et al., 2001). When relative priority is high, employees regard the innovation as an important priority rather than a distraction from their "real work" (Klein et al., 2001). The higher the relative priority of implementing an innovation, the more effective the implementation is likely to be (Helfrich, Weiner, et al., 2007; Klein et al., 2001). The ability of an organization to fully implement may be a function of how many other initiatives or changes have been rolled out in the recent past, which may lead to being overwhelmed with yet another implementation (Greenhalgh, Robert, et al., 2004; Gustafson et al., 2003); as a consequence, implementation may become a low priority.

Regarding quantitative measurement of this construct: In a systematic review of quantitative measures related to implementation, Powell et al. identified two measures (Powell et al., 2021). Using PAPERS criteria of measurement quality with an aggregate scale ranging from -9 to +36 (Lewis, Mettert, Stanick, et al., 2021), the two measures had scores of -1 and 3. Results indicate the need for continued development of high-quality measures.

### H. Incentive Systems

*Tangible and/or intangible incentives and rewards and/or disincentives and punishments support implementation and delivery of the innovation.*

The original CFIR described “incentives and rewards” that include extrinsic incentives such as goal-sharing awards, performance reviews, promotions, and raises in salary as well as less tangible incentives such as increased stature or respect (Helfrich, Weiner, et al., 2007; Klein et al., 2001). Tangible and/or intangible incentives and rewards, disincentives and punishments, or performance feedback can help to support implementation and delivery of the innovation (Balliet et al., 2011; Clark & Wilson, 1961; Helfrich, Weiner, et al., 2007; Klein et al., 2001) and is affirmed by more recent literature (Tagliabue et al., 2020).

Financial incentives and performance evaluations are important for reinforcing beliefs that behavior will lead to desirable results (Gustafson et al., 2003; Leeman et al., 2007). Strong incentives were found to be positively associated with implementation effectiveness (Helfrich, Weiner, et al., 2007; Klein et al., 2001). Well-designed incentives can bolster the degree to which new behaviors are positively or negatively valued, which heightens intention to change, a precursor to actual change (Gershon et al., 2004). A four-item “reward system” subscale is included as part of the Competing Values Framework measure of culture, and it was found that the number of different types of compensation incentives used is positively associated with comprehensiveness of the use of best practices by healthcare organizations (Shortell et al., 2001).

Regarding quantitative measurement of this construct: In a systematic review of quantitative measures related to implementation, Powell et al. identified three measures (Powell et al., 2021). Using PAPERS criteria of measurement quality with an aggregate scale ranging from -9 to +36 (Lewis, Mettert, Stanick, et al., 2021), only one measure was assessed with a score of 5. Results indicate the need for continued development of high-quality measures.

### I. Mission Alignment

*Implementing and delivering the innovation is in line with the overarching commitment, purpose, or goals in the Inner Setting.*

The original CFIR highlighted the importance of aligning the innovation with the Inner Setting mission (Kochevar & Yano, 2006; Simpson & Dansereau, 2007; VanDeusen Lukas et al., 2007) and other concurrent initiatives (Wagner et al., 2017).

Helfrich et al. found that perceived fit with mission was an important predictor of successful implementation (Helfrich, Weiner, et al., 2007). This alignment requires being well-informed and understanding the goals related to an innovation (Helfrich, Weiner, et al., 2007). From an organizational perspective, the degree to which goals (aligned with mission) are clearly communicated, acted upon, and measured, monitored, and reported is important for successful implementation (see also Engaging and Communications) (VanDeusen Lukas et al., 2007). Among the most effective ways to engage key individuals (e.g., leaders) is to have a change effort that is aligned with and contributes to achieving organizational goals (VanDeusen Lukas et al., 2007). A shared sense of responsibility as well as understanding organizational goals and believing task decisions are based on those goals, may contribute to implementation success (Simpson & Dansereau, 2007). The Chronic Care Model emphasizes the importance of relying on multiple methods of evaluation and feedback about achieving mission, including clinical, performance, economic evaluations, and experience (Bodenheimer, 2002; Bodenheimer et al., 2002).

Regarding quantitative measurement of this construct: In a systematic review of quantitative measures related to implementation, Powell et al. identified three measures for original CFIR’s Goals and Feedback (Powell et al., 2021). Using PAPERS criteria of measurement quality with an aggregate scale ranging from -9 to +36 (Lewis, Mettert, Stanick, et al., 2021), scores for two assessed measures ranged from 1 to 4. Results indicate the need for continued development of high-quality measures.

### J. Available Resources

*Resources are available to implement and deliver the innovation. Use this construct to capture themes related to Available Resources that are not included in the subconstructs below.*

Provision of funding, space, and materials and equipment are key for implementation and delivery of the innovation (Ashok et al., 2018; Dy et al., 2015; Leeman et al., 2019). In addition, Flottorp et al.’s checklist for implementation lists availability of resources that include financial, facilities, equipment and supplies, (Flottorp et al., 2013). See Individuals: Characteristics: Opportunity for staffing allocations that are specific to implementing or delivering the innovation.

The original CFIR elaborated on Available Resources, stating that the level of resources dedicated for implementation is positively associated with implementation (Denis et al., 2002; Leeman et al., 2007; Meyers et al., 1999; Perrin et al., 2006; Pronovost et al., 2006; Rabin et al., 2008) but is not necessarily sufficient to guarantee success (Stetler, 2001).

Regarding quantitative measurement of this construct: In a systematic review of quantitative measures related to implementation, Weiner et al. identified 19 measures (Weiner et al., 2020). Using PAPERS criteria of measurement quality with an aggregate scale ranging from -9 to +36 (Lewis, Mettert, Stanick, et al., 2021), 14 measures (73.68%) had sufficient information for assessment and scores ranged from -2 to +8. The Texas Christian University Program Training Needs Survey (Simpson, 2002) had the highest score. Results indicate the need for continued development of high-quality measures.

#### 1. Funding

*Funding is available to implement and deliver the innovation.*

The original CFIR noted that financial resources is a partial mediator between management support and implementation policy and procedures (Klein et al., 2001).

#### 2. Space

*Physical space is available to implement and deliver the innovation.*

Squires et al. define space as a resource as “the presence/absence, design, maintenance, and allocation of areas that are properly equipped, clean, and of sufficient size and number for the provision of health care in a facility” (see also Structural Characteristics: Physical Infrastructure) (Squires et al., 2019 p3 Additional File 2).

#### 3. Materials & Equipment

*Supplies are available to implement and deliver the innovation.*

### K. Access to Knowledge & Information

*Guidance and/or training is accessible to implement and deliver the innovation.*

The accessibility of guidance, training, and education related to the innovation and its implementation are critical to successful implementation and delivery of the innovation (Dy et al., 2015; Greenhalgh, Robert, et al., 2004).

The original CFIR further elaborated on the importance of the ease of access to digestible information about the innovation and how to incorporate the innovation into work tasks (Greenhalgh, Robert, et al., 2004; Helfrich, Weiner, et al., 2007; Klein et al., 2001; Wallin et al., 2006). The number of different knowledgeable occupational types or specialties who are involved with the implementation is positively associated with effective implementation (Wallin et al., 2006). When timely on-the-job training is available, especially at the team level, implementation is more likely to be successful (Greenhalgh, Robert, et al., 2004). Education, training, and access to information about the innovation are all key strategies to move deliverers from unengaged to fully committed users of the innovation (R. P. Grol et al., 2007).

Regarding quantitative measurement of this construct: In a systematic review of quantitative measures related to implementation, Weiner et al. identified six measures (Weiner et al., 2020). Using PAPERS criteria of measurement quality with an aggregate scale ranging from -9 to +36 (Lewis, Mettert, Stanick, et al., 2021), 5 measures (83.33%) had sufficient information for assessment and scores ranged from -1 to +6. The Structured Interview of Evidence Use (Palinkas et al., 2016) had the highest score. Results indicate the need for continued development of high-quality measures.

## IV. INDIVIDUALS DOMAIN

***Individuals:*** *The roles and characteristics of individuals.*

This domain highlights the importance of the roles and characteristics of individuals involved with implementing, delivering, and/or receiving the innovation (Ashok et al., 2018; Barwick et al., 2020; Breimaier et al., 2015; Dy et al., 2015; Kerins et al., 2020; Moretto et al., 2019; Varsi et al., 2015).

Users may find it helpful to consider a matrix of roles and their characteristics; coding the relevant characteristics for each role enables users to explore differences in characteristics across types of roles, which is crucial for adapting implementation strategies and the innovation (see Adapting).

| **Roles:** | **Characteristics:** | | | |
| --- | --- | --- | --- | --- |
|  | Need | Capability | Opportunity | Motivation |
| 1. High-level Leaders |  |  |  |  |
| 1. Mid-level Leaders |  |  |  |  |
| 1. Opinion Leaders |  |  |  |  |
| 1. Implementation Facilitators |  |  |  |  |
| 1. Implementation Leads |  |  |  |  |
| 1. Implementation Team Members |  |  |  |  |
| 1. Other Implementation Support |  |  |  |  |
| 1. Innovation Deliverers |  |  |  |  |
| 1. Innovation Recipients |  |  |  |  |

### ROLES SUBDOMAIN

This subdomain includes the roles of individuals involved with implementing, delivering, and/or receiving the innovation. Terms for implementation and innovation roles are used inconsistently and sometimes synonymously in the literature (Greenhalgh, Robert, et al., 2004); as a result, it is important for each user to clearly define roles for each project. Each of the roles listed below may reside in the Inner or Outer Setting. For example, blended implementation facilitation approaches involve Implementation Facilitators who may be affiliated with a centralized entity in the Outer Setting and who partner with an Implementation Lead in the Inner Setting (S. N. Smith et al., 2020).

#### Leaders

The updated CFIR divides formal leadership roles into two levels (see below). Commitment of individuals (see also Characteristics: Motivation) in formal leadership roles at multiple levels is a significant and frequently assessed determinant of implementation outcomes (Kirk et al., 2015). Commitment, involvement, and accountability (see also Characteristics: Motivation and Opportunity) of leaders and managers have a significant influence on the success of implementation (Klein et al., 2001; Meyers et al., 1999; VanDeusen Lukas et al., 2007). Anything less than wholehearted support from leaders, dooms implementation to failure (Repenning, 2002). Leadership support in terms of commitment and active interest leads to a stronger implementation climate which is, in turn, related to implementation effectiveness; this association is strengthened, the more users are required to work together to implement (Helfrich, Weiner, et al., 2007; Klein et al., 2001). Committed leaders have managerial patience (taking a long-term view rather than short-term) to allow time for the oft-inevitable reduction in productivity until the innovation takes hold (Klein et al., 2001) and can be important conduits to help persuade other individuals via interpersonal channels and by modeling norms (see Culture: Learning-Centeredness) associated with implementing an innovation (Leeman et al., 2007). Leaders are important for their ability to network (see also Relational Connections) and negotiate for resources (see also Available Resources), allocating time (see also Characteristics: Opportunity) and priority (see also Relative Priority).

#### A. High-level Leaders

*Individuals with a high level of authority, including key decision-makers, executive leaders, or directors.*

High-level Leaders include leaders with the authority to dedicate resources and to make decisions about whether to adopt, implement, and or/sustain the innovation (see Opportunity) (Dy et al., 2015).

The involvement of leaders and managers (Klein et al., 2001; Moretto et al., 2019; VanDeusen Lukas et al., 2007) is often critical to implementation success (see Motivation). Different levels of leadership may have differential effects on implementation success, thus the updated CFIR distinguishes between levels of leadership (see Mid-level Leaders below) (Ilott et al., 2012).

Regarding quantitative measurement of this construct (see also Characteristics: Motivation): In a systematic review of quantitative measures related to implementation, Weiner et al. identified 24 measures (Weiner et al., 2020). Using PAPERS criteria of measurement quality with an aggregate scale ranging from -9 to +36 (Lewis, Mettert, Stanick, et al., 2021), 17 measures (70.83%) had sufficient information for assessment and scores ranged from -1 to +14. The Implementation Leadership Scale (Aarons et al., 2014) had the highest score of 14. Results indicate the need for continued development of high-quality measures.

#### B. Mid-level Leaders

*Individuals with a moderate level of authority, including leaders supervised by a high-level leader who supervise others.*

Mid-level managers are a key link between strategic decisions from High-level Leaders and the people who must execute implementation and accomplish delivery of the innovation (S. Birken et al., 2018; S. A. Birken & Currie, 2021). Mid-level Leaders can include clinical leaders who often lead implementation efforts (Ilott et al., 2012) and/or direct supervisors of Implementation Leads and others involved in implementation. Mid-level leaders are benefited by having skills in mediating between high-level strategy in the Inner Setting and day-to-day activities and in diffusing, selling, and synthesizing information related to the innovation and its implementation (see Capability) (S. Birken et al., 2018). These leaders are more likely to support implementation if they believe that doing so will promote their own Inner Setting goals (see also Inner Setting: Mission Alignment) and if they feel involved in discussions about the implementation (Meyers et al., 1999).

See High-level Leaders for information on potential quantitative measures.

#### C. Opinion Leaders

*Individuals with informal influence on the attitudes and behaviors of others.*

Opinion Leaders have informal influence on the attitudes and behaviors of people involved with implementing or delivering the innovation (Flodgren et al., 2011; Greenhalgh, Robert, et al., 2004; E. Rogers, 2003). Waltz et al. reported on one study that found that informing opinion leaders was the fifth most commonly recommended implementation strategy by participating implementation experts (Waltz et al., 2019).

The original CFIR elaborated on this role further by acknowledging that opinion leaders can exert a strong negative or positive effect (Locock et al., 2001). The role and definition of opinion leaders is varied and complex. Locock et al. highlighted two types of opinion leaders: experts and peers (Locock et al., 2001). Expert opinion leaders exert influence through their authority and status (see Opportunity) (Greenhalgh, Robert, et al., 2004). Peer opinion leaders exert influence through their representativeness and credibility (Greenhalgh, Robert, et al., 2004). Implementation Facilitators or Leads may use Opinion Leaders in a social system as “lieutenants” in diffusion activities (E. Rogers, 2003). Opinion leaders can lose the respect of their peers if they come to be regarded as a professional change agent (E. Rogers, 2003; L. Rogers et al., 2020). The effect of Opinion Leaders on promoting use of innovations is mixed based on a review of randomized control trials ranging from -6% to +25% in improving behaviors of healthcare professionals (Doumit et al., 2007).

Regarding quantitative measurement of this construct: In a systematic review of quantitative measures related to implementation, Dorsey et al. identified five measures (Dorsey et al., 2021). Using PAPERS criteria of measurement quality with an aggregate scale ranging from -9 to +36 (Lewis, Mettert, Stanick, et al., 2021), three (60%) of the measures could not be scored, one had a score of zero and the other a score of two. Results indicate the need for continued development of high-quality measures.

#### D. Implementation Facilitators

*Individuals with subject matter expertise who assist, coach, or support implementation.*

Implementation Facilitators are individuals with subject matter expertise who assist, mentor, coach, or support implementation (S. N. Smith et al., 2020). The original CFIR elaborated further, stating that the PARiHS framework describes facilitators who are task-oriented versus holistic, the latter approach being valued more highly than the former (Rycroft-Malone, Kitson, et al., 2002). Ideally, “the role of the appropriately prepared facilitator, along with the team(s) they are working with, is to construct a programme of change that meets individual and team learning needs” (A. L. Kitson et al., 2008)(p 22). Implementation Facilitators may include any individual who provides guidance to the Implementation Leads or Teams (Ritchie et al., 2020; Solberg et al., 2021). Implementation Facilitators can play an integral role throughout implementation, formally influencing or facilitating innovation decisions in a desirable direction. External facilitators usually have professional training in a technical field related to organizational change science or in the technology being introduced into the organization (Ritchie et al., 2020). This role includes outside researchers who may be implementing a multi-site innovation study and other formally appointed individuals from the Outer Setting, e.g., a facilitator from a corporate or regional office or a hired consultant. Strong Implementation Facilitators have characteristics of empathy, curiosity, commitment, critical thinking, and advancing equity (Metz et al., 2020) and skills in five overarching areas: building relationships; changing processes; transferring knowledge and skills for continued learning; planning and leading; assessing people, process, and outcomes (see Characteristics) (Ritchie et al., 2020).

#### E. Implementation Leads

*Individuals who lead efforts to implement the innovation.*

Implementation Leads may emerge organically out of a grassroots (bottom-up) initiative to e.g., improve use of an innovation (Bonawitz et al., 2020; Damschroder, Banaszak-Holl, et al., 2009). Alternatively, Implementation Leads may be identified through top-down assignment. Ilot et al. found that “none of the instigators” of implementation were formally appointed in their cross-case comparison study, though some ultimately assumed that role (Ilott et al., 2012). Individuals who volunteer to lead may be more effective than those who were assigned the role (Bonawitz et al., 2020).

The term Implementation Lead is used to focus on individuals who are leading the implementation effort. These individuals take on the role as coordinator, project manager, team leader, or other similar responsibility. These leaders may or may not have dedicated time allocation to the role (see Opportunity). The term champion may be used to describe the person leading implementation (Miech et al., 2018); it is important to distinguish the role of champion versus championing behavior that can be exhibited by many of the listed implementation roles (see Motivation). Implementation Leads benefit from having project management skills, including critical thinking, influence, motivation, grit, conscientiousness, negotiation, participatory leadership style, and problem-solving (see Characteristics) (Barron & Barron, n.d.-a; Bonawitz et al., 2020; Müller & Turner, 2010).

The original CFIR elaborated on this role further by noting that ideally, Implementation Leads dedicate themselves to supporting, marketing, and ‘driving through an [implementation]’ (Greenhalgh, Glenn Robert, et al., 2004), overcoming indifference or resistance that the innovation may provoke in an organization. Ideally, Implementation Leads bring a high degree of passion, creativity, and willingness to take risks in accomplishing implementation goals (see Characteristics) (Maidique, 1980). There is strong to moderate support for the role of Implementation Leads on implementation outcomes, (Greenhalgh, Robert, et al., 2004; Helfrich, Weiner, et al., 2007; Miech et al., 2018; E. Rogers, 2003) though a more recent review found mixed impacts (Santos et al., 2022).

Regarding quantitative measurement of this construct: In a systematic review of quantitative measures related to implementation, Dorsey et al. did not identify any measures (search terms included implementation leader, coordinator, project manager, or team leader), but they did identify five measures related to champions (search terms included champion, transformational leader, campaigner, promoter, proponent, or supporter) (Dorsey et al., 2021). Using PAPERS criteria of measurement quality with an aggregate scale ranging from -9 to +36 (Lewis, Mettert, Stanick, et al., 2021), scores for the three champion measures that could be assessed ranged from -1 to +5. Results indicate the need for continued development of high-quality measures.

#### F. Implementation Team Members

*Individuals who collaborate with and support the Implementation Leads to implement the innovation, ideally including Innovation Deliverers and Recipients.*

Implementation Team Members include individuals who directly or indirectly participate in implementation and support the Implementation Leads. Implementation teams can play a critical role in implementation (Dy et al., 2015; Klein & Sorra, 1996; Means et al., 2020; Sue Dopson & Louise Fitzgerald, 2006), because Implementation Leads are not as effective alone (Miech et al., 2018). The original CFIR elaborated that engaging team members tasked with implementing an innovation (or to be “first users”), is an often-overlooked part of implementation; implementation teams ideally include deliverers and recipients. It is vital that Implementation Team Members are carefully and thoughtfully selected or respectfully encouraged to volunteer (Edmondson et al., 2001; Greenhalgh, Robert, et al., 2004; Pronovost et al., 2008) (see also Teaming, Engaging). The positive influence of having the “right people in the right seats” (Collins, 2009) is strong; having the wrong people or missing key opportunities to engage important individuals on the team can have negative influence on implementation success.

#### G. Other Implementation Support

*Individuals who support the Implementation Leads and/or Implementation Team Members to implement the innovation.*

Other key roles in implementation include individuals who assist the Implementation Leads and Team Members with implementation. These individuals can perform many different functions, including providing technical assistance for information technology, human resources, contracting, etc. It can also include integrators who help to build relationships between Inner Settings (Dy et al., 2015).

#### H. Innovation Deliverers

*Individuals who are directly or indirectly delivering the innovation.*

Dy et al. (Dy et al., 2015) include administrators, clinicians, and others who deliver or support delivery of the innovation (within and outside the Inner Setting). Users are strongly encouraged to use the published CFIR Outcomes Addendum to guide conceptualization of this role because Deliverers can play multiple roles, e.g., implementation team members, opinion leaders, or implementation leads, in addition to delivering the innovation (Damschroder et al., 2022).

#### I. Innovation Recipients

*Individuals who are directly or indirectly receiving the innovation.*

Innovation Recipients include anyone expected to benefit from implementation of the innovation. It is important to center recipients to help ensure their needs (see Process: Assessing Needs) are prioritized (see Inner Setting: Culture-Recipient-centeredness) (Dy et al., 2015; Godbee et al., 2020). Types of recipients have included, but are not limited to, community health workers, outreach teams, nurses, community members within communities (Naidoo et al., 2018), teachers, parents, students, food service staff (Norman et al., 2015; Okamoto et al., 2020; Tabak & Moreland-Russell, 2015) in schools, or farmers and extension service individuals in farming (Tinc et al., 2018). Other terms used for Innovation Recipients have included consumers or clients. Innovation Recipients are determined by the goals and focus for implementing an innovation.

The original CFIR acknowledged that within healthcare, many theories of research uptake or implementation acknowledge the importance of accounting for patient (recipient) characteristics (Feldstein & Glasgow, 2008; Graham & Logan, 2004; Rycroft-Malone, Kitson, et al., 2002). Users are strongly encouraged to use the published CFIR Outcomes Addendum to guide conceptualization of this role because recipients can play multiple roles, e.g., implementation team members, in addition to receiving the innovation (Damschroder et al., 2022).

### CHARACTERISTICS SUBDOMAIN

This subdomain includes the characteristics of individuals involved with implementing, delivering, and/or receiving the innovation. It includes characteristics related to professional skills and capabilities, autonomy, and level of involvement (Ashok et al., 2018; Dy et al., 2015; Moretto et al., 2019). Some users combine use of the CFIR with the Theoretical Domains Framework (TDF) (S. A. Birken et al., 2017), which was developed with the intent “…to simplify and integrate a plethora of behavior change theories and make theory more accessible to, and usable by, other disciplines” (Cane et al., 2012). The TDF contains 84 behavior change-related constructs, organized into 14 domains. TDF domains are, in turn, mapped to an even more highly synthesized representation called the COM-B system (Michie et al., 2011, 2014). The COM-B was developed as a simplified system by which to acknowledge key domains related to behavior change based on consensus of behavioral theorists and a principle of criminal law defining specific prerequisites for volitional behavior. Three of the updated Characteristics are based on this COM-B system. The COM-B posits that the broad categories of *Capability* (e.g., skills), *Opportunity* (e.g., autonomy), and *Motivation* (e.g., commitment) shape behavior (Michie et al., 2011). We encourage users to draw on this extensive work and the work of other individual-level behavior change scientists (e.g., Theory of Planned Behavior (Ajzen, 2011) or the Social Ecological Theory (Stokols, 1996)) when more detailed theories are needed. Alternatively, users may rely on role-specific theories, for example, facilitation (Albers et al., 2020; Metz et al., 2020) and project management (Barron & Barron, n.d.-b; Müller & Turner, 2010) theories relevant for Implementation Facilitators and Implementation Leads; leadership (Albers et al., 2020; Metz et al., 2020) theories relevant for High- and Mid-level Leaders.

#### Need

*The individual(s) has deficits related to survival, well-being, or personal fulfillment, which will be addressed by implementation and/or delivery of the innovation.*

Aims, wishes, and needs are important to assess for all constituents (Breimaier et al., 2015) (see also Process: Assessing Needs), and the level of awareness of recipient and deliverer needs is an important implementation determinant (Dy et al., 2015; Godbee et al., 2020). Within healthcare delivery settings, consideration of patient needs must be integral to any implementation that seeks to improve patient outcomes (Institute of Medicine, 2001).

#### Capability

*The individual(s) has interpersonal competence, knowledge, and skills to fulfill Role.*

Michie et al. include psychological and physical ability (Michie et al., 2011) within Capability. Intrapersonal competence, knowledge, and skills to fulfill roles are important for successful implementation (Ashok et al., 2018; Dy et al., 2015). Past experiences with implementation and/or the innovation helps build capability (King et al., 2019). Capabilities including personal traits of competence and learning style are important for tailoring training strategies (Barwick et al., 2020; Greenhalgh, Robert, et al., 2004).

The original CFIR elaborated further saying that the competence of individuals to judge the effectiveness of an innovation is facilitated by their understanding of underlying principles that justify using the innovation (E. Rogers, 2003). Skilled use and delivery of the innovation is a key outcome metric for effective implementation (Klein & Sorra, 1996), but assessments of skill can also be a key implementation determinant; see the CFIR Outcomes Addendum for more detail about the nuanced but important distinctions between determinants and outcomes (Damschroder et al., 2022). Capability is important to assess at individual and sub-group levels to assess quality of implementation and prospects for sustainability. If knowledge, for example through training (see Inner Setting: Access to Knowledge and Information), is not obtained prior to an individual having to use the innovation, rejection and discontinuance are likely (Klein et al., 2001). When knowledge can be codified and transferred across contexts, implementation is more likely to be successful (Greenhalgh, Robert, et al., 2004).

Self-efficacy, confidence in one’s ability to make the change, has been widely studied and is among the most common individual measures in theories of individual change (Bandura, 1977; R. P. Grol et al., 2007; US DHHS-National Cancer Institute, 2005). Individuals with high confidence in their capability are more likely to embrace the innovation and exhibit committed use even in the face of obstacles. If individuals are not confident in their ability to use the innovation or experience a level of failure early-on, they may not be motivated to persist in the face of challenges that may arise (US DHHS-National Cancer Institute, 2005).

Regarding quantitative measurement of this construct: In a systematic review of quantitative measures related to implementation, Stanick et al. identified 104 measures plus 28 subscale measures of knowledge and beliefs about the innovation and 24 measures plus 16 subscale measures of self-efficacy (Stanick et al., 2021). Using PAPERS criteria of measurement quality with an aggregate scale ranging from -9 to +36 (Lewis, Mettert, Stanick, et al., 2021), The Texas Christian University Training Needs Survey (Simpson, 2002) achieved the highest measurement score of 13 out of a maximum quality score of 36 for knowledge and beliefs. The Counselor Activity Self-Efficacy Scales (CASES) (Lent et al., 2003) achieved the highest PAPERS score for self-efficacy, scoring 15. These findings indicate the need for continued development of high-quality measures.

#### Opportunity

*The individual(s) has availability, scope, and power to fulfill Role.*

Michie et al. broadly define opportunity as “all the factors that lie outside the individual that make the behavior possible or prompt it” (Michie et al., 2011). In the updated CFIR, themes related to this construct are specific to implementing and delivering an innovation in the Inner Setting, including staff availability and sufficient time allocation, autonomy, and control to fulfill the role (Ashok et al., 2018; Dy et al., 2015; Means et al., 2020; Moretto et al., 2019). The quality of support provided by the Inner Setting to individuals is positively associated with implementation (Ovretveit, 2002).

The original CFIR provided further elaboration on this construct. Allocation of time by the organization is a key component of Opportunity; individuals involved in implementation will be more effective if they have dedicated time rather than as a distraction on top of other job duties (Feldstein & Glasgow, 2008). Implementation will be more effective when key individuals dedicate time and energy and are empowered and supported by their organization in their efforts to implement and deliver the innovation (Brach et al., 2008; Feldstein & Glasgow, 2008; Fixsen, 2007). However, Inner Settings may have “slack resources” (see also Inner Setting: Work Infrastructure) that enables people to “squeeze” time on top of their regular duties, to implement the innovation without noticeable unintended impacts. Ideally, leaders empower Innovation Deliverers and Implementation Leads and Teams by providing autonomy from rules, procedures, and systems of the organization so they can establish creative solutions to existing problems and generate support from other members of the organization.

#### Motivation

*The individual(s) is committed to fulfilling Role.*

Motivation includes brain processes that energize and direct behavior (Michie et al., 2011) and commitment, the act of binding oneself to a course of action intellectually and/or emotionally (Cane et al., 2012). This construct includes commitment of individuals to fulfill their role (Ashok et al., 2018; Dy et al., 2015). Perceptions of the commitment of leaders was captured in the original CFIR as part of the Inner Setting, but the updated CFIR recognizes the importance of capturing this theme for all individual roles. Enthusiastic use of an innovation is reflected by a positive affective response to the innovation (Klein & Sorra, 1996). Klein et al. define implementation outcomes based on measures of enthusiastic versus compliant use (Klein & Sorra, 1996); thus, care should be taken to clearly capture these concepts as determinants of implementation versus implementation outcomes; refer to the CFIR Outcomes Addendum for more detail (Damschroder et al., 2022). Often, subjective opinions obtained from peers based on personal experiences are convincing and help to generate enthusiasm (Pronovost et al., 2006). The converse is true as well, creating a negative source of active or passive resistance (Meyers et al., 1999; Saint et al., 2009). The degree to which new behaviors are positively or negatively valued heightens intention to change, which is a precursor to actual change (Gershon et al., 2004).

The following paragraphs provide further elaboration for individual-level constructs from the original CFIR that are included as themes within the new Motivation construct.

Championing as a behavior can be an important indicator of Motivation (Bonawitz et al., 2020; Miech et al., 2018). Individuals exhibiting this level of commitment are actively involved with the implementation and willing to risk informal status and reputation because they believe so strongly in the innovation (Schon, 1963). Such highly committed, championing behavior may occur across roles. For example, deliverers are most effective when they can defend and develop cross-functional coalitions of individuals who strongly believe in the innovation and are able to articulate the benefits in a way to move other individuals to fully embrace the innovation.

Individual stage of change reflects the phase an individual is in, as they progresses toward skilled, enthusiastic, and sustained use of the innovation (R. P. Grol et al., 2007; Klein et al., 2001). Prochaska’s trans-theoretical model characterizes stages of change as pre-contemplation, contemplation, preparation, and action and maintenance (Prochaska & Velicer, 1997). Rogers’ diffusion theory delineates five stages (E. Rogers, 2003). Grol et al. describe a five-stage model with ten sub-stages based on their synthesis of the literature (R. P. Grol et al., 2007).

How individuals perceive their organization (Inner Setting) and their relationship and commitment to the Inner Setting may affect willingness fully engage in implementation efforts or to use the innovation (Abraham, 2000; Cummings et al., 2007; Estabrooks et al., 2007; Greenberg, 1990). How strongly organizational identity is taken on by individuals may bolster implementation or delivery of the innovation (Pearce & Ensley, 2004; A. C. Smith et al., 1983). Within the Inner Setting, the alignment between the meaning individuals attach to the innovation versus the meaning communicated by upper management has a strong influence on whether key individuals will commit to the innovation (Greenhalgh, Robert, et al., 2004). For example, an innovation that leadership believes will improve performance may be perceived as a threat to autonomy in treatment decisions by physicians. Meaning in this context can be negotiated and reframed through discussions across organizational networks (Greenhalgh, Robert, et al., 2004).

Regarding quantitative measurement for themes related to Motivation, in a systematic review of quantitative measures designed to assess individual characteristics in implementation, Stanick et al. found 2 measures of stage-of-change (Stanick et al., 2021) and 7 measures plus 3 subscale measures of individual identification with the organization. Using PAPERS criteria of measurement quality with an aggregate scale ranging from -9 to +36 (Lewis, Mettert, Stanick, et al., 2021), only one measure could be scored for stage of change and it received a 4 out of a maximum quality score of 36. The Work Environment Scale (Insel & Moos, 1974) achieved the highest PAPERS score for identification with the organization, scoring 7. These findings indicate the need for continued development of high-quality measures.

## IMPLEMENTATION PROCESS DOMAIN

***Implementation Process:*** *The activities and strategies used to implement the innovation.*

Approaches aimed at sustained implementation approaches vary widely (Lennox et al., 2018). Activities can be accomplished in any order, but the Dynamic Sustainability Framework describes an initial implementation phase that emphasizes getting structures and processes in place according to protocol (including first-order changes that include strategies and tasks to get the innovation initially running day-to-day; e.g., problem-solving, staffing allocations), ideally followed by a longer-term phase of sustained incremental optimization to increasingly improve fit of the innovation with context (including second-order changes to continuously improve by teams with a focus on increasingly deeply embedded processes to establish new routines (Chambers et al., 2013)). This approach helps to ensure sustained change (Reed et al., 2018).

The original CFIR elaborated further by highlighting that theories abound in how implementation (or change) should be enacted, embodied by theories of total quality management, integrated care, complexity theory, organizational learning, and others (R. P. Grol et al., 2007). Essential activities of the implementation process common across organizational change models and aligned with advances in implementation science are included in this domain. These activities may be accomplished formally or informally on a spectrum from bottom-up grassroots or top-down mandated change efforts. Often changes are made in a spiral, stop-and-start, or incremental approach to implementation (Van de Ven et al., 1999). Constructs broadly reflect the plan-do-study-act (adapt/abandon) (PDSA) approach to incremental testing and implementation (Institute for Healthcare Improvement, 2003; Perla et al., 2013): Planning, Doing, Reflecting and Evaluating, and then Adapting in response to the evaluation. Ideally, each activity is revisited, expanded, refined, and re-evaluated as needed, throughout the course of initial and sustained implementation.

Regarding quantitative measurement of this domain: In a systematic review of quantitative measures related to implementation, Dorsey et al. identified four measures (Dorsey et al., 2021). Using PAPERS criteria of measurement quality with an aggregate scale ranging from -9 to +36 (Lewis, Mettert, Stanick, et al., 2021), The Implementation Phases Inventory (Bradshaw et al., 2009) had the highest score (10), followed closely by the Veterans Health Administration Continuous Quality Improvement Degree of Implementation Survey (V. A. Parker et al., 1999) with a score of 9. Results indicate the need for continued development of high-quality measures.

### A. Teaming

*Join together, intentionally coordinating and collaborating on interdependent tasks, to implement the innovation.*

Researchers have noted the important influence of team capabilities, social relationships, teamwork, and morale in accomplishing their goals (see also Implementation Team Members and Characteristics) (Chan et al., 2011; A. L. Parker et al., 2019; L. Rogers et al., 2020; Sarkies et al., 2020; Spitzer-Shohat et al., 2018), which are encapsulated within Edmondson’s concept of teaming (Edmondson, 2012). Working with teams, rather than relying a “hero-model” where motivated individuals do all or most of the work, is an important ingredient for sustained change (Doyle et al., 2013; Edmondson, 2012; Miake-Lye et al., 2020). Edmondson describes levels of maturity in teaming: 1) the need for teaming is recognized; 2) individuals communicate; 3) steps and hand-offs are coordinated; 4) reciprocal and interdependent action unfolds; 4) teams take time and space to reflect; 5) teaming mindset in psychologically safe spaces is adopted (Edmondson, 2012).

Strategies to build coalitions and conduct local consensus discussions (key activities in teaming) are among the most frequently recommended strategies to address implementation barriers (Waltz et al., 2019). The original CFIR asserted that any one role cannot function in isolation; relationships between individuals can be more important than individual roles or individual characteristics (Plsek & Wilson, 2001).

### Assessing Needs

*Collect information about priorities, preferences, and needs of people. Use this construct to capture themes related to Assessing Needs that are not included in the subconstructs below.*

Assessing the needs of both recipients and deliverers to guide the implementation process is an important determinant to implementation success (Ashok et al., 2018; Dy et al., 2015) as well as equity in implementation.

#### Innovation Deliverers

*Collect information about the priorities, preferences, and needs of deliverers to guide implementation and delivery of the innovation.*

Assessing deliverer needs facilitates the “Quadruple Aim,” which includes improving the work-life and well-being of clinicians and staff (Bodenheimer & Sinsky, 2014).

#### Innovation Recipients

*Collect information about the priorities, preferences, and needs of recipients to guide implementation and delivery of the innovation.*

Assessing recipient needs facilitates patient-centered care and patient safety culture within healthcare delivery (Nieva & Sorra, 2003) and is included in implementation process frameworks, e.g., Getting to Outcomes (Wandersman et al., 2000).

### C. Assessing Context

*Collect information to identify and appraise barriers and facilitators to implementation and delivery of the innovation.*

Assessing context is foundational within implementation science (Damschroder, Aron, et al., 2009; Nilsen & Bernhardsson, 2019; J. D. Smith et al., 2020; Wierenga et al., 2013) and ideally utilizes a determinant framework to guide the assessment (Damschroder, 2020; Nilsen, 2015; Nilsen & Bernhardsson, 2019). Assessments should consider all salient contextual factors — both modifiable and non-modifiable. Workarounds can be developed for non-modifiable factors, and strategies can be targeted to modify factors (e.g., increase the knowledge of deliverers about the innovation).

### D. Planning

*Identify roles and responsibilities, outline specific steps and milestones, and define goals and measures for implementation success in advance.*

The fundamental objective of planning is to design a course of action to promote effective implementation by building local capacity for using the innovation, collectively and individually (Mendel et al., 2008); this construct includes contingency planning (Dy et al., 2015), goal-setting (see also Reflecting and Evaluation for monitoring progress toward set goals), selecting strategies, and occurs within context of incremental implementation approaches or testing cycles.

The original CFIR elaborated on this construct, stating that regardless of the degree of complexity of the innovation, simple, clear, and detailed implementation plans, schedules, and task assignments contribute to successful implementation (Gustafson et al., 2003). The specific steps in plans should be based on the underlying theories or models used to promote change at organization and individual levels (R. P. Grol et al., 2007). For example, the Institute for Healthcare Improvement (Institute for Healthcare Improvement, 2003, 2005), Grol et al. (R. Grol et al., 2005), and Glisson and Schoenwald (Glisson & Schoenwald, 2005) describe comprehensive approaches to implementation on which implementation plans can be developed. However, these theories prescribe different sets of activities because they were developed in different contexts (though commonalities exist as well). Grol et al. list 14 different bodies of theories for changing behaviors in social or organizational contexts (R. P. Grol et al., 2007), and Estabrooks et al. list 18 different models of organizational innovation (Estabrooks et al., 2006). Thus, the content of plans will vary depending on the theory or model being used to guide implementation and the context within which implementation will occur.

Setting goals and identifying metrics or measures to track progress are integral aspects planning (Greenhalgh, Robert, et al., 2004; E. Rogers, 2003). Planned measures should include implementation and innovation outcomes that are important to key constituencies (von Thiele Schwarz et al., 2019), including at a minimum, leaders, deliverers, and recipients (Damschroder et al., 2022). Goals should be specific, measurable, attainable, relevant, and timely (the SMART rubric) (Brach et al., 2008); goal planning includes documenting objectives, benchmarks, and timeline with consideration of feasibility and adequacy (Dy et al., 2015). Note that the degree to which monitoring and evaluation occurs is captured in Reflecting and Evaluating.

Regarding quantitative measurement of this construct: In a systematic review of quantitative measures related to implementation, Dorsey et al. identified five measures (Dorsey et al., 2021). Using PAPERS criteria of measurement quality with an aggregate scale ranging from -9 to +36 (Lewis, Mettert, Stanick, et al., 2021), The Community Leader Survey (Planning) (Valente et al., 2007) had the highest score of seven. Results indicate the need for continued development of high-quality measures.

### E. Tailoring Strategies

*Choose and operationalize implementation strategies to address barriers, leverage facilitators, and fit context.*

Implementation strategies are chosen and tailored to address findings from Assessing Needs and Assessing Context (Powell et al., 2017). The Expert Recommendations for Implementing Change (ERIC) provides a list of strategies (Powell et al., 2015); Waltz et al. reported recommendations from implementation experts about which strategies might best address each CFIR barrier (Waltz et al., 2019); this can provide an initial starting point to select strategies. There are many approaches for tailoring strategies (Powell et al., 2017), including implementation mapping (Fernandez et al., 2019), which can be used to operationalize chosen strategies and then reported following published guidelines (Proctor et al., 2013).

### F. Engaging

*Attract and encourage participation in implementation and/or the innovation. Use this construct to capture themes related to Engaging that are not included in the subconstructs below.*

This construct includes attracting and involving appropriate individuals — specifically deliverers and recipients – in the implementation and use of the innovation through a combined strategy of social marketing, education, role modeling (Cruess et al., 2008), training, and other similar activities (see also Assessing Needs). Engaging deliverers and recipients of an innovation is an often overlooked part of implementation (Pronovost et al., 2008), but doing so helps ensure sustained change (Doyle et al., 2013; Lennox et al., 2018, 2020). As a result, it is important to identify and engage deliverers and recipients early and often (Breimaier et al., 2015). The original CFIR asserted that if supporters of the innovation outnumber and are better strategically positioned than the opponents, the implementation is more likely to be successful (Greenhalgh, Robert, et al., 2004).

Regarding quantitative measurement of this construct: In a systematic review of quantitative measures related to implementation, Dorsey et al. identified one measure (Dorsey et al., 2021). Using PAPERS criteria of measurement quality with an aggregate scale ranging from -9 to +36 (Lewis, Mettert, Stanick, et al., 2021), The single measure scored 2 for predictive validity; no other information on validity was available. Results indicate the need for continued development of high-quality measures.

#### 1. Innovation Deliverers

*Attract and encourage deliverers to serve on the implementation team and/or to deliver the innovation.*

Engaging potential deliverers in meaningful problem-solving is one of five interactive elements critical to transform patient care (VanDeusen Lukas et al., 2007).

#### 2. Innovation Recipients

*Attract and encourage recipients to serve on the implementation team and/or participate in the innovation.*

Engaging individuals early in implementation who share similar socioeconomic, professional, educational, and cultural backgrounds with intended recipients is more likely to lead to higher participation in the innovation (Dopp et al., 2019; Ilott et al., 2012).

### G. Doing

*Implement in small steps, tests, or cycles of change to trial and cumulatively optimize delivery of the innovation.*

Doing includes using iterative approaches such as Plan-Do-Study-Act change cycles or incremental steps instead of implementing all components in all planned areas within a specified period (Ashok et al., 2018; Dy et al., 2015). The original CFIR elaborated further, stating that taking an incremental approach leads to breaking the innovation down into manageable parts that can be implemented incrementally (Damanpour, 1991). The ability to implement an innovation incrementally (sometimes referred to as divisibility (R. P. Grol et al., 2007)) can help decrease perceptions of innovation complexity and thus implementation difficulty. Thus, more complex innovations especially benefit from incremental implementation (Damanpour, 1991; Grossman, 1970; Normann, 1971). In addition, incremental approaches allow deliverers to have enough time to do their work and to learn new skills associated with the new innovation (Helfrich, Weiner, et al., 2007). Successes in early increments of the implementation help increase confidence, give an opportunity to adjust the innovation or implementation (see Adapting), and gain new “believers.” Usability testing (with deliverers and recipients) promotes successful adaptation of the innovation (Feldstein & Glasgow, 2008). Dry runs (simulations or practice sessions) to allow team members to learn how to use the innovation before going live (Edmondson et al., 2001) is also beneficial.

Doing also includes trialing, which allows for time to build experience and expertise, reflect upon and test the innovation (Rycroft-Malone, Kitson, et al., 2002), gain confidence, and build an environment of psychological safety (Edmondson et al., 2001). The ability to trial is a key feature of the PDSA approach, allowing users increase coordination to manage interdependence (see also Trialability) (Leeman et al., 2007; Rabin et al., 2008). Implementers or deliverers need to be able to stop the innovation and reverse its effects if it causes problems or is ineffective (Feldstein & Glasgow, 2008). Results from trials will provide needed information about how best to implement to other units to minimize workflow disruption.

### H. Reflecting & Evaluating

*Collect and discuss quantitative and qualitative information about the success of implementation and/or the innovation. Use this construct to capture themes related to Reflecting & Evaluating that are not included in the subconstructs below.*

Though less attention has been paid historically to the need for group and personal reflection, more recent literature is acknowledging its key role in strong teaming and team-building (Edmondson, 2012). Dedicating time for reflecting or debriefing before, during, and after implementation is one way to promote shared learning and improvements (Edmondson et al., 2001). The original CFIR asserted that these times of reflection help foster a learning climate – one in which a successful implementation can be ingrained into institutional memory and help improve the odds for future implementations (Edmondson et al., 2001; Simpson & Dansereau, 2007). Even failures, when reflected upon in an effective way, can lead to future success when the root causes are uncovered with psychologically safety (Klein & Sorra, 1996); failure is key for strengthening learning within organizations (Lapré & Nembhard, 2011).

Timely availability to data for monitoring, evaluation, and process improvement is important (Dy et al., 2015). The original CFIR highlighted that data to support reflection and evaluation includes quantitative and qualitative feedback about the experience, progress, and quality of implementation efforts. Evaluation includes traditional forms of feedback, such as reports and graphs, as well as qualitative feedback and anecdotal stories of success (US DHHS-National Cancer Institute, 2005). Feedback on progress toward those goals or objectives is a key behavior change technique in many individual behavior change theories and models (Carey et al., 2018; US DHHS-National Cancer Institute, 2005) and has strong to moderate evidence supporting implementation at an organizational level (Greenhalgh, Robert, et al., 2004). One review found that effects of using audit and feedback mechanisms to improve practices can lead to small to moderate effects (Jamtvedt et al., 2006), a finding confirmed by a more recent review (Ivers et al., 2012). It is hard for people to continue the work without sufficient feedback that is tightly coupled to goals that are important to them (Hysong et al., 2006). Reviewing progress toward goals allows people to assess whether the innovation is creating value (Jamtvedt et al., 2006).

Regarding quantitative measurement of this construct: In a systematic review of quantitative measures related to implementation, Dorsey et al. identified five measures (Dorsey et al., 2021). Using PAPERS criteria of measurement quality with an aggregate scale ranging from -9 to +36 (Lewis, Mettert, Stanick, et al., 2021), three were assessed with score ranging from 2 to 8. The Community Leader Survey (Prevention Activity Progress Subscale) (Valente et al., 2007) had the highest score of eight. Results indicate the need for continued development of high-quality measures.

#### 1. Implementation

*Collect and discuss quantitative and qualitive information about the success of implementation.*

This construct includes tracking progress towards achieving implementation goals and milestones using rigorous and pragmatic monitoring and evaluation methods (Greenhalgh, Robert, et al., 2004; E. Rogers, 2003)

#### 2. Innovation

*Collect and discuss quantitative and qualitative information about the success of the innovation. that indicates the degree to which innovation outcomes are achieved.*

This construct includes monitoring and optimizing outcomes for key constituencies (Damschroder et al., 2022; von Thiele Schwarz et al., 2019) using rigorous and pragmatic monitoring and evaluating methods.

### I. Adapting

*Modify the innovation and/or the Inner Setting for optimal fit and integration into work processes.*

There is extensive literature that emphasizes the key role of adaptations to ensure optimal fit between the innovation and setting(s) within which it is being implemented and delivered (Chambers et al., 2013; Chambers & Norton, 2016; Greenhalgh, Robert, et al., 2004; Hill et al., 2018; Kerins et al., 2020; Lennox et al., 2018; Moore et al., 2021; Stirman et al., 2019; von Thiele Schwarz et al., 2019; Wells et al., 2020). Most of the literature focuses on adapting the innovation but adaptations of the setting (e.g., workflows) are often needed as well, to optimize fit (Chambers et al., 2013; Hill et al., 2018; Kerins et al., 2020; Wells et al., 2020) and achieve optimal sustained outcomes (von Thiele Schwarz et al., 2019). Adapting includes determining and documenting the types of adaptations made, ideally following an adaptation framework (Stirman et al., 2019). We refer users to above cited articles for further guidance on best practices for adapting.

# References

Aarons, G. A., Ehrhart, M. G., & Farahnak, L. R. (2014). The implementation leadership scale (ILS): Development of a brief measure of unit level implementation leadership. *Implementation Science*, *9*(1), 45. https://doi.org/10.1186/1748-5908-9-45

Aarons, G. A., Hurlburt, M., & Horwitz, S. M. (2011). Advancing a Conceptual Model of Evidence-Based Practice Implementation in Public Service Sectors. *Administration and Policy in Mental Health and Mental Health Services Research*, *38*(1), 4–23. https://doi.org/10.1007/s10488-010-0327-7

Abraham, R. (2000). Organizational cynicism: Bases and consequences. *Genet Soc Gen Psychol Monogr*, *126*(3), 269–292.

Aiken, M., Bacharach, S. B., & French, J. L. (1980). Organizational Structure, Work Process, and Proposal Making in Administrative Bureaucracies. *Academy of Management Journal*, *23*(4), 631–652. https://doi.org/10.5465/255553

Ajzen, I. (2011). The theory of planned behaviour: Reactions and reflections. *Psychology & Health*, *26*(9), 1113–1127. https://doi.org/10.1080/08870446.2011.613995

Albers, B., Metz, A., & Burke, K. (2020). Implementation support practitioners – a proposal for consolidating a diverse evidence base. *BMC Health Services Research*, *20*(1), 368. https://doi.org/10.1186/s12913-020-05145-1

Albrecht, L., Archibald, M., Arseneau, D., & Scott, S. D. (2013). Development of a checklist to assess the quality of reporting of knowledge translation interventions using the Workgroup for Intervention Development and Evaluation Research (WIDER) recommendations. *Implementation Science*, *8*(1), 52. https://doi.org/10.1186/1748-5908-8-52

Ashok, M., Hung, D., Rojas-Smith, L., Halpern, M. T., & Harrison, M. (2018). Framework for Research on Implementation of Process Redesigns: *Quality Management in Health Care*, *27*(1), 17–23. https://doi.org/10.1097/QMH.0000000000000158

Aubert, B. A., & Hamel, G. (2001). Adoption of smart cards in the medical sector: *Social Science & Medicine*, *53*(7), 879–894. https://doi.org/10.1016/S0277-9536(00)00388-9

Bakker, A. B. (2015). Towards a multilevel approach of employee well-being. *European Journal of Work and Organizational Psychology*, *24*(6), 839–843. https://doi.org/10.1080/1359432X.2015.1071423

Baldridge, J. V., & Burnham, R. A. (1975). Organizational Innovation: Individual, Organizational, and Environmental Impacts. *Administrative Science Quarterly*, *20*(2), 165. https://doi.org/10.2307/2391692

Balliet, D., Mulder, L. B., & Van Lange, P. A. M. (2011). Reward, punishment, and cooperation: A meta-analysis. *Psychological Bulletin*, *137*(4), 594–615. https://doi.org/10.1037/a0023489

Bandura, A. (1977). Self-efficacy: Toward a unifying theory of behavioral change. *Psychol Rev*, *84*(2), 191–215.

Barnsley, J., Lemieux-Charles, L., & McKinney, M. M. (1998). Integrating Learning into Integrated Delivery Systems: *Health Care Management Review*, *23*(1), 18–28. https://doi.org/10.1097/00004010-199801000-00003

Barron, M., & Barron, A. (n.d.-a). Project Management Areas of Expertise. In *Project Management*. sue. Retrieved March 23, 2022, from https://cnx.org/contents/XpF315mY@11.6:_nDfs3nk@2/Project-Management-Areas-of-Expertise

Barron, M., & Barron, A. (n.d.-b). Project Management Areas of Expertise. In *Project Management*. https://cnx.org/contents/XpF315mY@11.6:_nDfs3nk@2/Project-Management-Areas-of-Expertise

Barwick, M., Barac, R., Kimber, M., Akrong, L., Johnson, S. N., Cunningham, C. E., Bennett, K., Ashbourne, G., & Godden, T. (2020). Advancing implementation frameworks with a mixed methods case study in child behavioral health. *Translational Behavioral Medicine*, *10*(3), 685–704. https://doi.org/10.1093/tbm/ibz005

Berta, W. B., & Baker, R. (2004). Factors that Impact the Transfer and Retention of Best Practices for Reducing Error in Hospitals: *Health Care Management Review*, *29*(2), 90–97. https://doi.org/10.1097/00004010-200404000-00002

Berwick, D. M., Nolan, T. W., & Whittington, J. (2008). The Triple Aim: Care, Health, And Cost. *Health Affairs*, *27*(3), 759–769. https://doi.org/10.1377/hlthaff.27.3.759

Birken, S. A., & Currie, G. (2021). Using organization theory to position middle-level managers as agents of evidence-based practice implementation. *Implementation Science*, *16*(1), 37. https://doi.org/10.1186/s13012-021-01106-2

Birken, S. A., Powell, B. J., Presseau, J., Kirk, M. A., Lorencatto, F., Gould, N. J., Shea, C. M., Weiner, B. J., Francis, J. J., Yu, Y., Haines, E., & Damschroder, L. J. (2017). Combined use of the Consolidated Framework for Implementation Research (CFIR) and the Theoretical Domains Framework (TDF): A systematic review. *Implementation Science : IS*, *12*(1), 2. https://doi.org/10.1186/s13012-016-0534-z

Birken, S., Clary, A., Tabriz, A. A., Turner, K., Meza, R., Zizzi, A., Larson, M., Walker, J., & Charns, M. (2018). Middle managers’ role in implementing evidence-based practices in healthcare: A systematic review. *Implementation Science*, *13*(1), 149. https://doi.org/10.1186/s13012-018-0843-5

Bodenheimer, T. (2002). Improving Primary Care for Patients With Chronic Illness. *JAMA*, *288*(14), 1775. https://doi.org/10.1001/jama.288.14.1775

Bodenheimer, T., & Sinsky, C. (2014). From Triple to Quadruple Aim: Care of the Patient Requires Care of the Provider. *The Annals of Family Medicine*, *12*(6), 573–576. https://doi.org/10.1370/afm.1713

Bodenheimer, T., Wagner, E. H., & Grumbach, K. (2002). Improving Primary Care for Patients With Chronic Illness: The Chronic Care Model, Part 2. *JAMA*, *288*(15), 1909. https://doi.org/10.1001/jama.288.15.1909

Bonawitz, K., Wetmore, M., Heisler, M., Dalton, V. K., Damschroder, L. J., Forman, J., Allan, K. R., & Moniz, M. H. (2020). Champions in context: Which attributes matter for change efforts in healthcare? *Implementation Science*, *15*(1), 62. https://doi.org/10.1186/s13012-020-01024-9

Brach, C., N. Lenfestey, A. Roussel, J. Amoozegar, & A. Sorensen. (2008). *Will It Work Here? A Decisionmaker’s Guide to Adopting Innovations*. Agency for Healthcare Research & Quality (AHRQ). https://www.ahrq.gov/innovations/will-work/index.html

Bradshaw, C. P., Debnam, K., Koth, C. W., & Leaf, P. (2009). Preliminary Validation of the Implementation Phases Inventory for Assessing Fidelity of Schoolwide Positive Behavior Supports. *Journal of Positive Behavior Interventions*, *11*(3), 145–160. https://doi.org/10.1177/1098300708319126

Brehem, J., & Rahn, W. (1997). Individual Level Evidence for the Causes and Consequences of Social Capital. *American Journal of Political Science*, *41*(3), 999–1023.

Breimaier, H. E., Heckemann, B., Halfens, R. J. G., & Lohrmann, C. (2015). The Consolidated Framework for Implementation Research (CFIR): A useful theoretical framework for guiding and evaluating a guideline implementation process in a hospital-based nursing practice. *BMC Nursing*, *14*(1), 43. https://doi.org/10.1186/s12912-015-0088-4

Burnes, B. (2005). Complexity theories and organizational change. *International Journal of Management Reviews*, *7*(2), 73–90. https://doi.org/10.1111/j.1468-2370.2005.00107.x

Burns, L. R., & Wholey, D. R. (1993). Adoption and abandonment of matrix management programs: Effects of organizational characteristics and interorganizational networks. *Academy of Management Journal. Academy of Management*, *36*(1), 106–138.

Butler, M., Epstein, R. A., Totten, A., Whitlock, E. P., Ansari, M. T., Damschroder, L. J., Balk, E., Bass, E. B., Berkman, N. D., Hempel, S., Iyer, S., Schoelles, K., & Guise, J.-M. (2017). AHRQ series on complex intervention systematic reviews—paper 3: Adapting frameworks to develop protocols. *Journal of Clinical Epidemiology*, *90*, 19–27. https://doi.org/10.1016/j.jclinepi.2017.06.013

Cane, J., O’Connor, D., & Michie, S. (2012). Validation of the theoretical domains framework for use in behaviour change and implementation research. *Implementation Science*, *7*(1), 37. https://doi.org/10.1186/1748-5908-7-37

Carey, R. N., Connell, L. E., Johnston, M., Rothman, A. J., de Bruin, M., Kelly, M. P., & Michie, S. (2018). Behavior Change Techniques and Their Mechanisms of Action: A Synthesis of Links Described in Published Intervention Literature. *Annals of Behavioral Medicine*. https://doi.org/10.1093/abm/kay078

Carroll, C., Patterson, M., Wood, S., Booth, A., Rick, J., & Balain, S. (2007). A conceptual framework for implementation fidelity. *Implement Sci*, *2*(1), 40.

Chambers, D. A., Glasgow, R. E., & Stange, K. C. (2013). The dynamic sustainability framework: Addressing the paradox of sustainment amid ongoing change. *Implementation Science*, *8*(1), 117.

Chambers, D. A., & Norton, W. E. (2016). The Adaptome: Advancing the Science of Intervention Adaptation. *Am J Prev Med*, *51*(4 Suppl 2), S124-31. https://doi.org/10.1016/j.amepre.2016.05.011

Chan, K. S., Hsu, Y.-J., Lubomski, L. H., & Marsteller, J. A. (2011). Validity and usefulness of members reports of implementation progress in a quality improvement initiative: Findings from the Team Check-up Tool (TCT). *Implementation Science*, *6*(1), 115. https://doi.org/10.1186/1748-5908-6-115

Clark, P. B., & Wilson, J. Q. (1961). Incentive Systems: A Theory of Organizations. *Administrative Science Quarterly*, *6*(2), 129. https://doi.org/10.2307/2390752

Collins, J. (2009). Good to Great—(Why Some Companies Make the Leap and others Don’t). *NHRD Network Journal*, *2*(7), 102–105. https://doi.org/10.1177/0974173920090719

Cruess, S. R., Cruess, R. L., & Steinert, Y. (2008). Role modelling—Making the most of a powerful teaching strategy. *Bmj*, *336*(7646), 718–721.

Cummings, G. G., Estabrooks, C. A., Midodzi, W. K., Wallin, L., & Hayduk, L. (2007). Influence of Organizational Characteristics and Context on Research Utilization. *Nursing Research*, *56*(4), S24–S39. https://doi.org/10.1097/01.NNR.0000280629.63654.95

Curran, G. M. (2020). Implementation science made too simple: A teaching tool. *Implementation Science Communications*, *1*, 27. https://doi.org/10.1186/s43058-020-00001-z

Damanpour, F. (1991). Organizational Innovation: A Meta-Analysis of Effects of Determinants and Moderators. *The Academy of Management Journal*, *34*(3), 555–590.

Damschroder, L. J. (2020). Clarity out of chaos: Use of theory in implementation research. *Psychiatry Research*, *283*, 112461.

Damschroder, L. J., Aron, D. C., Keith, R. E., Kirsh, S. R., Alexander, J. A., & Lowery, J. C. (2009). Fostering implementation of health services research findings into practice: A consolidated framework for advancing implementation science. *Implementation Science: IS*, *4*, 50. https://doi.org/10.1186/1748-5908-4-50

Damschroder, L. J., Banaszak-Holl, J., Kowalski, C. P., Forman, J., Saint, S., & Krein, S. L. (2009). The role of the champion in infection prevention: Results from a multisite qualitative study. *Quality & Safety in Health Care*, *18*(6), 434–440. https://doi.org/10.1136/qshc.2009.034199

Damschroder, L. J., Reardon, C. M., Opra Widerquist, M. A., & Lowery, J. (2022). Conceptualizing outcomes for use with the Consolidated Framework for Implementation Research (CFIR): The CFIR Outcomes Addendum. *Implementation Science*, *17*(1), 7. https://doi.org/10.1186/s13012-021-01181-5

Denis, J.-L., Hébert, Y., Langley, A., Lozeau, D., & Trottier, L.-H. (2002). Explaining Diffusion Patterns for Complex Health Care Innovations: *Health Care Management Review*, *27*(3), 60–73. https://doi.org/10.1097/00004010-200207000-00007

Dewar, R. D., & Dutton, J. E. (1986). The Adoption of Radical and Incremental Innovations: An Empirical Analysis. *Management Science*, *32*(11), 1422–1433.

Dopp, A. R., Narcisse, M.-R., Mundey, P., Silovsky, J. F., Smith, A. B., Mandell, D., Funderburk, B. W., Powell, B. J., Schmidt, S., Edwards, D., Luke, D., & Mendel, P. (2020). A scoping review of strategies for financing the implementation of evidence-based practices in behavioral health systems: State of the literature and future directions. *Implementation Research and Practice*, *1*, 263348952093998. https://doi.org/10.1177/2633489520939980

Dopp, A. R., Parisi, K. E., Munson, S. A., & Lyon, A. R. (2019). A glossary of user-centered design strategies for implementation experts. *Translational Behavioral Medicine*, *9*(6), 1057–1064. https://doi.org/10.1093/tbm/iby119

Dopson, S., FitzGerald, L., Ferlie, E., Gabbay, J., & Locock, L. (2010). No magic targets! Changing clinical practice to become more evidence based. *Health Care Management Review*, *35*(1), 2–12. https://doi.org/10.1097/HMR.0b013e3181c88e79

Dorsey, C. N., Mettert, K. D., Puspitasari, A. J., Damschroder, L. J., & Lewis, C. C. (2021). A systematic review of measures of implementation players and processes: Summarizing the dearth of psychometric evidence. *Implementation Research and Practice*, *2*, 263348952110024. https://doi.org/10.1177/26334895211002474

Doumit, G., Gattellari, M., Grimshaw, J., & O’Brien, M. A. (2007). Local opinion leaders: Effects on professional practice and health care outcomes. *The Cochrane Database of Systematic Reviews*, *1*, CD000125. https://doi.org/10.1002/14651858.CD000125.pub3

Doyle, C., Howe, C., Woodcock, T., Myron, R., Phekoo, K., McNicholas, C., Saffer, J., & Bell, D. (2013). Making change last: Applying the NHS institute for innovation and improvement sustainability model to healthcare improvement. *Implementation Science*, *8*(1), 127. https://doi.org/10.1186/1748-5908-8-127

Dy, S. M., Ashok, M., Wines, R. C., & Rojas Smith, L. (2015). A Framework to Guide Implementation Research for Care Transitions Interventions: *Journal for Healthcare Quality*, *37*(1), 41–54. https://doi.org/10.1097/01.JHQ.0000460121.06309.f9

Edmondson, A. C. (2002). The Local and Variegated Nature of Learning in Organizations: A Group-Level Perspective. *Organization Science*, *13*(2), 128–146. https://doi.org/10.1287/orsc.13.2.128.530

Edmondson, A. C. (2012). *Teaming: How organizations learn, innovate, and compete in the knowledge economy*. Jossey-Bass.

Edmondson, A. C., Bohmer, R. M., & Pisana, G. P. (2001). Disrupted routines: Team learning and new technology implementation in hospitals. *Administrative Science Quarterly*, *46*(4), 685–716.

Estabrooks, C. A., Midodzi, W. K., Cummings, G. G., & Wallin, L. (2007). Predicting research use in nursing organizations: A multilevel analysis. *Nurs Res*, *56*(4 Suppl), S7-23.

Estabrooks, C. A., Thompson, D. S., Lovely, J. J., & Hofmeyer, A. (2006). A guide to knowledge translation theory. *J Contin Educ Health Prof*, *26*(1), 25–36.

Evans, W. D., & Hastings, G. (Eds.). (2008). *Public health branding: Applying marketing for social change*. Oxford University Press. https://www.google.com/books/edition/Public_Health_Branding/Voox6-zMQTsC?hl=en&gbpv=0

Feldstein, A. C., & Glasgow, R. E. (2008). A practical, robust implementation and sustainability model (PRISM) for integrating research findings into practice. *Jt Comm J Qual Patient Saf*, *34*(4), 228–243.

Ferlie, E. B., & Shortell, S. M. (2001). Improving the quality of health care in the United Kingdom and the United States: A framework for change. *Milbank Q*, *79*(2), 281–315.

Fernandez, M. E., ten Hoor, G. A., van Lieshout, S., Rodriguez, S. A., Beidas, R. S., Parcel, G., Ruiter, R. A. C., Markham, C. M., & Kok, G. (2019). Implementation Mapping: Using Intervention Mapping to Develop Implementation Strategies. *Frontiers in Public Health*, *7*, 158. https://doi.org/10.3389/fpubh.2019.00158

Fitzgerald, L. A. (2002). Chaos: The lens that transcends. *Journal of Organizational Change Management*, *15*(4), 339–358. https://doi.org/10.1108/09534810210433665

Fitzgerald, L. A., & van Eijnatten, F. M. (2002). Reflections: Chaos in organizational change. *Journal of Organizational Change Management*, *15*(4), 402–411.

Fitzgerald, L., & Dopson, S. (2006). Knowledge, credible evidence, and utilization. In *Knowledge to action? Evidence-based health care in context* (p. 223). Oxford University Press.

Fixsen, D. L. (2007). *Implementation Research: A Synthesis of the Literature*. University of South Florida, Louis de la Parte Florida Mental Health Institute.

Flodgren, G., Parmelli, E., Doumit, G., Gattellari, M., O’Brien, M. A., Grimshaw, J., & Eccles, M. P. (2011). Local opinion leaders: Effects on professional practice and health care outcomes. *Cochrane Database of Systematic Reviews*. https://doi.org/10.1002/14651858.CD000125.pub4

Flottorp, S. A., Oxman, A. D., Krause, J., Musila, N. R., Wensing, M., Godycki-Cwirko, M., Baker, R., & Eccles, M. P. (2013). A checklist for identifying determinants of practice: A systematic review and synthesis of frameworks and taxonomies of factors that prevent or enable improvements in healthcare professional practice. *Implementation Science*, *8*(1), 35. https://doi.org/10.1186/1748-5908-8-35

Frambach, R. T., & Schillewaert, N. (2001). Organizational innovation adoption: A multi-level framework of determinants and opportunities for future research. *Journal of Business Research*, *55*(2), 163–176.

Gershon, R., Stone, P. W., Bakken, S., & Larson, E. (2004). Measurement of Organizational Culture and Climate in Healthcare. *Journal of Nursing Administration*, *34*(1), 33–40.

Gittell, R. J., & Vidal, A. (1998). *Community organizing: Building social capital as a development strategy*. Sage Publications.

Gladwell, M. (2006). *The tipping point how little things can make a big difference*. Little, Brown : Hachette Book Group. https://eza.udesa.edu.ar/login?url=https://search.ebscohost.com/login.aspx?direct=true&db=nlebk&AN=762990&lang=es&site=ehost-live

Glisson, C., Landsverk, J., Schoenwald, S., Kelleher, K., Hoagwood, K. E., Mayberg, S., & Green, P. (2008). Assessing the Organizational Social Context (OSC) of Mental Health Services: Implications for Research and Practice. *Adm Policy Ment Health*, *35*(1–2), 98–113.

Glisson, C., & Schoenwald, S. K. (2005). The ARC organizational and community intervention strategy for implementing evidence-based children’s mental health treatments. *Ment Health Serv Res*, *7*(4), 243–259.

Godbee, K., Gunn, J., Lautenschlager, N. T., & Palmer, V. J. (2020). Refined conceptual model for implementing dementia risk reduction: Incorporating perspectives from Australian general practice. *Australian Journal of Primary Health*, *26*(3), 247. https://doi.org/10.1071/PY19249

Goh, S., & Richards, G. (1997). Benchmarking the learning capability of organizations. *European Management Journal*, *15*(5), 575–583. https://doi.org/10.1016/S0263-2373(97)00036-4

Goldberg, D. G., Soylu, T. G., Kitsantas, P., Grady, V. M., Elward, K., & Nichols, L. M. (2021). Burnout among Primary Care Providers and Staff: Evaluating the Association with Practice Adaptive Reserve and Individual Behaviors. *Journal of General Internal Medicine*, *36*(5), 1222–1228. https://doi.org/10.1007/s11606-020-06367-z

Gosepath, S. (2021). Equality. In E. N. Zalta (Ed.), *The Stanford Encyclopedia of Philosophy* (Summer 2021). Metaphysics Research Lab, Stanford University. https://plato.stanford.edu/archives/sum2021/entries/equality/

Graham, I. D., & Logan, J. (2004). Innovations in knowledge transfer and continuity of care. *Canadian Journal of Nursing Research*, *36*(2), 89–103.

Greenberg, J. (1990). Organizational justice: Yesterday, today, and tomorrow. *Journal of Management*, *16*(2), 399–432.

Greenhalgh, T., Glenn Robert, Paula Bate, Olympia Kyriakidou, Fraser Macfarlane, & Richard Peacock. (2004). *How to Spread Good Ideas* (p. 424). National Co-ordinating Centre for NHS Service Delivery and Organisation R & D.

Greenhalgh, T., Jackson, C., Shaw, S., & Janamian, T. (2016). Achieving Research Impact Through Co‐creation in Community‐Based Health Services: Literature Review and Case Study. *The Milbank Quarterly*, *94*(2), 392–429. https://doi.org/10.1111/1468-0009.12197

Greenhalgh, T., Robert, G., Macfarlane, F., Bate, P., & Kyriakidou, O. (2004). Diffusion of innovations in service organizations: Systematic review and recommendations. *Milbank Q*, *82*(4), 581–629.

Greenhalgh, T., Wherton, J., Papoutsi, C., Lynch, J., Hughes, G., A’Court, C., Hinder, S., Fahy, N., Procter, R., & Shaw, S. (2017). Beyond Adoption: A New Framework for Theorizing and Evaluating Nonadoption, Abandonment, and Challenges to the Scale-Up, Spread, and Sustainability of Health and Care Technologies. *Journal of Medical Internet Research*, *19*(11), e367. https://doi.org/10.2196/jmir.8775

Grol, R. P., Bosch, M. C., Hulscher, M. E., Eccles, M. P., & Wensing, M. (2007). Planning and studying improvement in patient care: The use of theoretical perspectives. *Milbank Q*, *85*(1), 93–138.

Grol, R., Wensing, M., & Eccles, M. (2005). *Improving Patient Care: The Implementation of Change in Clinical Practice*. Elsevier.

Grossman, J. B. (1970). The Supreme Court and Social Change: A Preliminary Inquiry. *American Behavioral Scientist*, *13*(4), 535–551. https://doi.org/10.1177/000276427001300405

Guise, J.-M., Savitz, L. A., & Friedman, C. P. (2018). Mind the Gap: Putting Evidence into Practice in the Era of Learning Health Systems. *Journal of General Internal Medicine*, *33*(12), 2237–2239. https://doi.org/10.1007/s11606-018-4633-1

Gustafson, D. H., Sainfort, F., Eichler, M., Adams, L., Bisognano, M., & Steudel, H. (2003). Developing and testing a model to predict outcomes of organizational change. *Health Serv Res*, *38*(2), 751–776.

Harrison, M. I., & Shortell, S. M. (2021). Multi‐level analysis of the learning health system: Integrating contributions from research on organizations and implementation. *Learning Health Systems*, *5*(2). https://doi.org/10.1002/lrh2.10226

Harvey, G., & Kitson, A. (2015). PARIHS revisited: From heuristic to integrated framework for the successful implementation of knowledge into practice. *Implementation Science*, *11*(1), 33. https://doi.org/10.1186/s13012-016-0398-2

Helfrich, C. D., Li, Y.-F., Mohr, D. C., Meterko, M., & Sales, A. E. (2007). Assessing an organizational culture instrument based on the Competing Values Framework: Exploratory and confirmatory factor analyses. *Implementation Science: IS*, *2*, 13. https://doi.org/10.1186/1748-5908-2-13

Helfrich, C. D., Weiner, B. J., McKinney, M. M., & Minasian, L. (2007). Determinants of implementation effectiveness: Adapting a framework for complex innovations. *Med Care Res Rev*, *64*(3), 279–303.

Hill, J. N., Locatelli, S. M., Bokhour, B. G., Fix, G. M., Solomon, J., Mueller, N., & LaVela, S. L. (2018). Evaluating broad-scale system change using the Consolidated Framework for Implementation Research: Challenges and strategies to overcome them. *BMC Research Notes*, *11*(1), 560. https://doi.org/10.1186/s13104-018-3650-9

Ho, M., Livingston, P., Bould, M. D., Nyandwi, J. D., Nizeyimana, F., Uwineza, J. B., & Urquhart, R. (2019). Barriers and facilitators to implementing a regional anesthesia service in a low-income country: A qualitative study. *Pan African Medical Journal*, *32*. https://doi.org/10.11604/pamj.2019.32.152.17246

Hoffmann, T. C., Glasziou, P. P., Boutron, I., Milne, R., Perera, R., Moher, D., Altman, D. G., Barbour, V., Macdonald, H., Johnston, M., Lamb, S. E., Dixon-Woods, M., McCulloch, P., Wyatt, J. C., Chan, A.-W., & Michie, S. (2014). Better reporting of interventions: Template for intervention description and replication (TIDieR) checklist and guide. *BMJ (Clinical Research Ed.)*, *348*, g1687. https://doi.org/10.1136/bmj.g1687

Hohmeier, K. C., Wheeler, J. S., Turner, K., Vick, J. S., Marchetti, M. L., Crain, J., & Brookhart, A. (2019). Targeting adaptability to improve Medication Therapy Management (MTM) implementation in community pharmacy. *Implementation Science*, *14*(1), 99. https://doi.org/10.1186/s13012-019-0946-7

Hull, L., Goulding, L., Khadjesari, Z., Davis, R., Healey, A., Bakolis, I., & Sevdalis, N. (2019). Designing high-quality implementation research: Development, application, feasibility and preliminary evaluation of the implementation science research development (ImpRes) tool and guide. *Implementation Science : IS*, *14*(1), 80. https://doi.org/10.1186/s13012-019-0897-z

Hysong, S. J., Best, R. G., & Pugh, J. A. (2006). Audit and feedback and clinical practice guideline adherence: Making feedback actionable. *Implementation Science*, *1*(1), 9. https://doi.org/10.1186/1748-5908-1-9

Ilies, R., Aw, S. S. Y., & Pluut, H. (2015). Intraindividual models of employee well-being: What have we learned and where do we go from here? *European Journal of Work and Organizational Psychology*, *24*(6), 827–838. https://doi.org/10.1080/1359432X.2015.1071422

Ilott, I., Gerrish, K., Booth, A., & Field, B. (2012). Testing the Consolidated Framework for Implementation Research on health care innovations from South Yorkshire: Testing the CFIR on health care innovations. *Journal of Evaluation in Clinical Practice*, n/a-n/a. https://doi.org/10.1111/j.1365-2753.2012.01876.x

Insel, P. M., & Moos, R. H. (1974). *Work environment scale*. Consulting Psychologists Press.

Institute for Healthcare Improvement. (2003). *The Breakthrough Series: IHI’s Collaborative Model for Achieving Breakthrough Improvement* (Innovation Series 2003, p. 20). Institute for Healthcare Improvement.

Institute for Healthcare Improvement. (2005). *Going Lean in Health Care* (IHI Innovation Series White Paper).

Institute of Medicine. (2001). *Crossing the Quality Chasm: A New Health System for the 21st Century*. National Academy Press.

Institute of Medicine (IOM). (2013). *Best Care at Lower Cost: The Path to Continuously Learning Health Care in America* (p. 13444). National Academies Press. https://doi.org/10.17226/13444

Ivers, N., Jamtvedt, G., Flottorp, S., Young, J. M., Odgaard-Jensen, J., French, S. D., O’Brien, M. A., Johansen, M., Grimshaw, J., & Oxman, A. D. (2012). Audit and feedback: Effects on professional practice and healthcare outcomes. *Cochrane Database of Systematic Reviews*. https://doi.org/10.1002/14651858.CD000259.pub3

Jamtvedt, G., Young, J. M., Kristoffersen, D. T., O’Brien, M. A., & Oxman, A. D. (2006). Does telling people what they have been doing change what they do? A systematic review of the effects of audit and feedback. *Quality and Safety in Health Care*, *15*(6), 433–436. https://doi.org/10.1136/qshc.2006.018549

Katz, D., & Kahn, R. L. (1966). *The social psychology of organizations*. Wiley.

Kerins, C., McHugh, S., McSharry, J., Reardon, C. M., Hayes, C., Perry, I. J., Geaney, F., Seery, S., & Kelly, C. (2020). Barriers and facilitators to implementation of menu labelling interventions from a food service industry perspective: A mixed methods systematic review. *International Journal of Behavioral Nutrition and Physical Activity*, *17*(1), 48. https://doi.org/10.1186/s12966-020-00948-1

Kimberly, J. R., & Evanisko, M. J. (1981). Organizational innovation: The influence of individual, organizational, and contextual factors on hospital adoption of technological and administrative innovations. *Academy of Management Journal. Academy of Management*, *24*(4), 689–713.

King, E. S., Moore, C. J., Wilson, H. K., Harden, S. M., Davis, M., & Berg, A. C. (2019). Mixed methods evaluation of implementation and outcomes in a community-based cancer prevention intervention. *BMC Public Health*, *19*(1), 1051. https://doi.org/10.1186/s12889-019-7315-y

Kirk, M. A., Kelley, C., Yankey, N., Birken, S. A., Abadie, B., & Damschroder, L. (2015). A systematic review of the use of the Consolidated Framework for Implementation Research. *Implementation Science*, *11*(1), 72. https://doi.org/10.1186/s13012-016-0437-z

Kirsh, S. R., Lawrence, R. H., & Aron, D. C. (2008). Tailoring an intervention to the context and system redesign related to the intervention: A case study of implementing shared medical appointments for diabetes. *Implement Sci*, *3*, 34. https://doi.org/1748-5908-3-34 [pii] 10.1186/1748-5908-3-34

Kitson, A., Harvey, G., & McCormack, B. (1998). Enabling the implementation of evidence based practice: A conceptual framework. *Quality and Safety in Health Care*, *7*(3), 149–158. https://doi.org/10.1136/qshc.7.3.149

Kitson, A. L., Rycroft-Malone, J., Harvey, G., McCormack, B., Seers, K., & Titchen, A. (2008). Evaluating the successful implementation of evidence into practice using the PARIHS framework: Theoretical and practical challenges. *Implement Sci*, *3*(1), 1.

Klein, K. J., Conn, A. B., & Sorra, J. S. (2001). Implementing computerized technology: An organizational analysis. *Journal of Applied Psychology*, *86*(5), 811–824.

Klein, K. J., & Sorra, J. S. (1996). The Challenge of Innovation Implementation. *The Academy of Management Review*, *21*(4), 1055–1080.

Kochevar, L. K., & Yano, E. M. (2006). Understanding health care organization needs and context. Beyond performance gaps. *J Gen Intern Med*, *21 Suppl 2*, S25-9.

Lapré, M. A., & Nembhard, I. M. (2011). Inside the Organizational Learning Curve: Understanding the Organizational Learning Process. *Foundations and Trends® in Technology, Information and Operations Management*, *4*(1), 1–103. https://doi.org/10.1561/0200000023

Leana, C. R., & Pil, F. K. (2006). Social Capital and Organizational Performance: Evidence from Urban Public Schools. *Organization Science*, *17*(3), 353–366. https://doi.org/10.1287/orsc.1060.0191

Leeman, J., Baernholdt, M., & Sandelowski, M. (2007). Developing a theory-based taxonomy of methods for implementing change in practice. *J Adv Nurs*, *58*(2), 191–200.

Leeman, J., Baquero, B., Bender, M., Choy-Brown, M., Ko, L. K., Nilsen, P., Wangen, M., & Birken, S. A. (2019). Advancing the use of organization theory in implementation science. *Preventive Medicine*, *129*, 105832. https://doi.org/10.1016/j.ypmed.2019.105832

Lehman, W. E. K., Greener, J. M., & Simpson, D. D. (2002). Assessing organizational readiness for change. *Journal of Substance Abuse Treatment*, *22*(4), 197–209. https://doi.org/10.1016/S0740-5472(02)00233-7

Lengnick-Hall, R., Gerke, D. R., Proctor, E. K., Bunger, A. C., Phillips, R. J., Martin, J. K., & Swanson, J. C. (2022). Six practical recommendations for improved implementation outcomes reporting. *Implementation Science*, *17*(1), 16. https://doi.org/10.1186/s13012-021-01183-3

Lengnick-Hall, R., Willging, C., Hurlburt, M., Fenwick, K., & Aarons, G. A. (2020). Contracting as a bridging factor linking outer and inner contexts during EBP implementation and sustainment: A prospective study across multiple U.S. public sector service systems. *Implementation Science*, *15*(1), 43. https://doi.org/10.1186/s13012-020-00999-9

Lennox, L., Linwood-Amor, A., Maher, L., & Reed, J. (2020). Making change last? Exploring the value of sustainability approaches in healthcare: a scoping review. *Health Research Policy and Systems*, *18*(1), 120. https://doi.org/10.1186/s12961-020-00601-0

Lennox, L., Maher, L., & Reed, J. (2018). Navigating the sustainability landscape: A systematic review of sustainability approaches in healthcare. *Implementation Science*, *13*(1), 27. https://doi.org/10.1186/s13012-017-0707-4

Lent, R. W., Hill, C. E., & Hoffman, M. A. (2003). Development and validation of the Counselor Activity Self-Efficacy Scales. *Journal of Counseling Psychology*, *50*(1), 97–108. https://doi.org/10.1037/0022-0167.50.1.97

Lewin, S., Hendry, M., Chandler, J., Oxman, A. D., Michie, S., Shepperd, S., Reeves, B. C., Tugwell, P., Hannes, K., Rehfuess, E. A., Welch, V., Mckenzie, J. E., Burford, B., Petkovic, J., Anderson, L. M., Harris, J., & Noyes, J. (2017). Assessing the complexity of interventions within systematic reviews: Development, content and use of a new tool (iCAT_SR). *BMC Medical Research Methodology*, *17*(1), 76. https://doi.org/10.1186/s12874-017-0349-x

Lewis, C. C., Mettert, K. D., Stanick, C. F., Halko, H. M., Nolen, E. A., Powell, B. J., & Weiner, B. J. (2021). The psychometric and pragmatic evidence rating scale (PAPERS) for measure development and evaluation. *Implementation Research and Practice*, *2*, 263348952110373. https://doi.org/10.1177/26334895211037391

Lewis, C. C., Mettert, K., & Lyon, A. R. (2021). Determining the influence of intervention characteristics on implementation success requires reliable and valid measures: Results from a systematic review. *Implementation Research and Practice*, *2*, 263348952199419. https://doi.org/10.1177/2633489521994197

Locock, L., Dopson, S., Chambers, D., & Gabbay, J. (2001). Understanding the role of opinion leaders in improving clinical effectiveness. *Social Science & Medicine*, *53*(6), 745–757. https://doi.org/10.1016/S0277-9536(00)00387-7

Maidique, M. A. (1980). Entrepeneurs, champions and technological innovation. *Sloan Management Review*, *21*, 59–76.

Martin, J. (2002). *Organizational culture: Mapping the terrain*. Sage Publications.

McEachern, B. M., Jackson, J., Yungblut, S., & Tomasone, J. R. (2019). Barriers and Facilitators to Implementing Exercise is Medicine Canada on Campus Groups. *Health Promotion Practice*, *20*(5), 751–759. https://doi.org/10.1177/1524839919830923

McHugh, S., Dorsey, C. N., Mettert, K., Purtle, J., Bruns, E., & Lewis, C. C. (2020). Measures of outer setting constructs for implementation research: A systematic review and analysis of psychometric quality. *Implementation Research and Practice*, *1*, 263348952094002. https://doi.org/10.1177/2633489520940022

Means, A. R., Kemp, C. G., Gwayi-Chore, M.-C., Gimbel, S., Soi, C., Sherr, K., Wagenaar, B. H., Wasserheit, J. N., & Weiner, B. J. (2020). Evaluating and optimizing the consolidated framework for implementation research (CFIR) for use in low- and middle-income countries: A systematic review. *Implementation Science*, *15*(1), 1–19. https://doi.org/10.1001/jamasurg.2017.5565

Mendel, P., Meredith, L. S., Schoenbaum, M., Sherbourne, C. D., & Wells, K. B. (2008). Interventions in organizational and community context: A framework for building evidence on dissemination and implementation in health services research. *Adm Policy Ment Health*, *35*(1–2), 21–37.

Merlo, G., Page, K., Zardo, P., & Graves, N. (2019). Applying an Implementation Framework to the Use of Evidence from Economic Evaluations in Making Healthcare Decisions. *Applied Health Economics and Health Policy*, *17*(4), 533–543. https://doi.org/10.1007/s40258-019-00477-4

Metz, A., Louison, L., Burke, K., & Ward, C. (2020). *Implementation Support Practitioner Profile: Guiding principles and core competencies for implementation practice* (p. 18). National Implementation Research Network. https://nirn.fpg.unc.edu/resources/implementation-support-practitioner-profile

Meyer, A. D., & Goes, J. B. (1988). Organizational Assimilation of Innovations: A Multilevel Contextual Analysis. *Academy of Management Journal*, *31*(4), 897–923. https://doi.org/10.5465/256344

Meyers, P. W., Sivakumar, K., & Nakata, C. (1999). Implementation of Industrial Process Innovations: Factors, Effects, and Marketing Implications. *Journal of Product Innovation Management*, *16*(3), 295–311. https://doi.org/10.1111/1540-5885.1630295

Miake-Lye, I. M., Delevan, D. M., Ganz, D. A., Mittman, B. S., & Finley, E. P. (2020). Unpacking organizational readiness for change: An updated systematic review and content analysis of assessments. *BMC Health Services Research*, *20*(1), 106. https://doi.org/10.1186/s12913-020-4926-z

Michie, S., Atkins, L., & West, R. (2014). *The behaviour change wheel: A guide to designing interventions*. Silverback.

Michie, S., van Stralen, M. M., & West, R. (2011). The behaviour change wheel: A new method for characterising and designing behaviour change interventions. *Implementation Science : IS*, *6*, 42. https://doi.org/10.1186/1748-5908-6-42

Miech, E. J., Rattray, N. A., Flanagan, M. E., Damschroder, L., Schmid, A. A., & Damush, T. M. (2018). Inside help: An integrative review of champions in healthcare-related implementation. *SAGE Open Medicine*, *6*, 205031211877326. https://doi.org/10.1177/2050312118773261

Moecker, R., Terstegen, T., Haefeli, W. E., & Seidling, H. M. (2021). The influence of intervention complexity on barriers and facilitators in the implementation of professional pharmacy services – A systematic review. *Research in Social and Administrative Pharmacy*, *17*(10), 1651–1662. https://doi.org/10.1016/j.sapharm.2021.01.013

Moore, G., Campbell, M., Copeland, L., Craig, P., Movsisyan, A., Hoddinott, P., Littlecott, H., O’Cathain, A., Pfadenhauer, L., Rehfuess, E., Segrott, J., Hawe, P., Kee, F., Couturiaux, D., Hallingberg, B., & Evans, R. (2021). Adapting interventions to new contexts—The ADAPT guidance. *BMJ*, n1679. https://doi.org/10.1136/bmj.n1679

Moretto, N., Comans, T. A., Chang, A. T., O’Leary, S. P., Osborne, S., Carter, H. E., Smith, D., Cavanagh, T., Blond, D., & Raymer, M. (2019). Implementation of simulation modelling to improve service planning in specialist orthopaedic and neurosurgical outpatient services. *Implementation Science*, *14*(1), 78. https://doi.org/10.1186/s13012-019-0923-1

Moullin, J. C., Dickson, K. S., Stadnick, N. A., Rabin, B., & Aarons, G. A. (2019). Systematic review of the Exploration, Preparation, Implementation, Sustainment (EPIS) framework. *Implementation Science*, *14*(1), 1. https://doi.org/10.1186/s13012-018-0842-6

Moullin, J. C., Sabater-Hernández, D., & Benrimoj, S. I. (2016). Qualitative study on the implementation of professional pharmacy services in Australian community pharmacies using framework analysis. *BMC Health Services Research*, *16*(1), 439. https://doi.org/10.1186/s12913-016-1689-7

Müller, R., & Turner, R. (2010). Leadership competency profiles of successful project managers. *International Journal of Project Management*, *28*(5), 437–448. https://doi.org/10.1016/j.ijproman.2009.09.003

Naidoo, N., Zuma, N., Khosa, N. S., Marincowitz, G., Railton, J., Matlakala, N., Jobson, G. A., Igumbor, J. O., McIntyre, J. A., Struthers, H. E., & Peters, R. P. H. (2018). Qualitative assessment of facilitators and barriers to HIV programme implementation by community health workers in Mopani district, South Africa. *PLOS ONE*, *13*(8), e0203081. https://doi.org/10.1371/journal.pone.0203081

Nembhard, I. M., & Edmonson, A. C. (2006). Making it safe: The effects of leader inclusiveness and professional status on psychological safety and improvement efforts in health care teams. *Journal of Organizational Behavior*, *27*, 941–966.

Nevedal, A. L., Reardon, C. M., Jackson, G. L., Cutrona, S. L., White, B., Gifford, A. L., Orvek, E., DeLaughter, K., White, L., King, H. A., Henderson, B., Vega, R., & Damschroder, L. (2020). Implementation and sustainment of diverse practices in a large integrated health system: A mixed methods study. *Implementation Science Communications*, *1*(1), 61. https://doi.org/10.1186/s43058-020-00053-1

Nieva, V. F., & Sorra, J. (2003). Safety culture assessment: A tool for improving patient safety in healthcare organizations. *Quality & Safety in Health Care*, *12 Suppl 2*, ii17-23. https://doi.org/10.1136/qhc.12.suppl_2.ii17

Nilsen, P. (2015). Making sense of implementation theories, models and frameworks. *Implementation Science*, *10*(1), 53.

Nilsen, P., & Bernhardsson, S. (2019). Context matters in implementation science: A scoping review of determinant frameworks that describe contextual determinants for implementation outcomes. *BMC Health Services Research*, *19*(1), 189. https://doi.org/10.1186/s12913-019-4015-3

Norman, Å., Nyberg, G., Elinder, L. S., & Berlin, A. (2015). One size does not fit all–qualitative process evaluation of the Healthy School Start parental support programme to prevent overweight and obesity among children in disadvantaged areas in Sweden. *BMC Public Health*, *16*(1), 37. https://doi.org/10.1186/s12889-016-2701-1

Normann, R. (1971). Organizational Innovativeness: Product Variation and Reorientation. *Administrative Science Quarterly*, *16*(2), 203. https://doi.org/10.2307/2391830

Okamoto, S. K., Helm, S., Chin, S. K., Hata, J., Hata, E., & Okamura, K. H. (2020). The implementation of a culturally grounded, school‐based, drug prevention curriculum in rural Hawai‘i. *Journal of Community Psychology*, *48*(4), 1085–1099. https://doi.org/10.1002/jcop.22222

Oswald, J. M., Boswell, J. F., Smith, M., Thompson-Brenner, H., & Brooks, G. (2019). Practice–research integration in the residential treatment of patients with severe eating and comorbid disorders. *Psychotherapy*, *56*(1), 134–148. https://doi.org/10.1037/pst0000180

Ovretveit, J. (2002). Quality collaboratives: Lessons from research. *Quality and Safety in Health Care*, *11*(4), 345–351. https://doi.org/10.1136/qhc.11.4.345

Palinkas, L. A., Garcia, A. R., Aarons, G. A., Finno-Velasquez, M., Holloway, I. W., Mackie, T. I., Leslie, L. K., & Chamberlain, P. (2016). Measuring Use of Research Evidence: The Structured Interview for Evidence Use. *Research on Social Work Practice*, *26*(5), 550–564. https://doi.org/10.1177/1049731514560413

Parker, A. L., Forsythe, L. L., & Kohlmorgen, I. K. (2019). TeamSTEPPS®: An evidence‐based approach to reduce clinical errors threatening safety in outpatient settings: An integrative review. *Journal of Healthcare Risk Management*, *38*(4), 19–31. https://doi.org/10.1002/jhrm.21352

Parker, V. A., Wubbenhorst, W. H., Young, G. J., Desai, K. R., & Charns, M. P. (1999). Implementing Quality Improvement in Hospitals: The Role of Leadership and Culture. *American Journal of Medical Quality*, *14*(1), 64–69. https://doi.org/10.1177/106286069901400109

Parry, K. W., & Proctor-Thomson, S. B. (2001). Testing the validity and reliability of the Organizational Description Questionnaire (ODQ). *International Journal of Organisational Behaviour*, *4*(3), 111–124.

Pearce, C. L., & Ensley, M. D. (2004). A reciprocal and longitudinal investigation of the innovation process: The central role of shared vision in product and process innovation teams (PPITs). *Journal of Organizational Behavior*, *25*(2), 259–278.

Perla, R. J., Provost, L. P., & Parry, G. J. (2013). Seven Propositions of the Science of Improvement: Exploring Foundations. *Quality Management in Health Care*, *22*(3), 170–186. https://doi.org/10.1097/QMH.0b013e31829a6a15

Perrin, K. M., Burke, S. G., O’Connor, D., Walby, G., Shippey, C., Pitt, S., McDermott, R. J., & Forthofer, M. S. (2006). Factors contributing to intervention fidelity in a multi-site chronic disease self-management program. *Implement Sci*, *1*, 26.

Pettigrew, A. M., Woodman, R. W., & Cameron, K. S. (2001). Studying Organizational Change and Development: Challenges for Future Research. *Academy of Management Journal*, *44*(4), 697–713. https://doi.org/10.5465/3069411

Pinnock, H., Barwick, M., Carpenter, C. R., Eldridge, S., Grandes, G., Griffiths, C. J., Rycroft-Malone, J., Meissner, P., Murray, E., Patel, A., Sheikh, A., & Taylor, S. J. C. (2017). Standards for Reporting Implementation Studies (StaRI) Statement. *BMJ*, i6795. https://doi.org/10.1136/bmj.i6795

Plsek, P. E., & Greenhalgh, T. (2001). Complexity science: The challenge of complexity in health care. *Bmj*, *323*(7313), 625–628.

Plsek, P. E., & Wilson, T. (2001). Complexity, leadership, and management in healthcare organisations. *Bmj*, *323*(7315), 746–749.

Powell, B. J., Beidas, R. S., Lewis, C. C., Aarons, G. A., McMillen, J. C., Proctor, E. K., & Mandell, D. S. (2017). Methods to Improve the Selection and Tailoring of Implementation Strategies. *The Journal of Behavioral Health Services & Research*, *44*(2), 177–194. https://doi.org/10.1007/s11414-015-9475-6

Powell, B. J., McMillen, J. C., Proctor, E. K., Carpenter, C. R., Griffey, R. T., Bunger, A. C., Glass, J. E., & York, J. L. (2012). A compilation of strategies for implementing clinical innovations in health and mental health. *Med Care Res Rev*, *69*(2), 123–157. https://doi.org/10.1177/1077558711430690

Powell, B. J., Mettert, K. D., Dorsey, C. N., Weiner, B. J., Stanick, C. F., Lengnick-Hall, R., Ehrhart, M. G., Aarons, G. A., Barwick, M. A., Damschroder, L. J., & Lewis, C. C. (2021). Measures of organizational culture, organizational climate, and implementation climate in behavioral health: A systematic review. *Implementation Research and Practice*, *2*, 263348952110188. https://doi.org/10.1177/26334895211018862

Powell, B. J., Waltz, T. J., Chinman, M. J., Damschroder, L. J., Smith, J. L., Matthieu, M. M., Proctor, E. K., & Kirchner, J. E. (2015). A refined compilation of implementation strategies: Results from the Expert Recommendations for Implementing Change (ERIC) project. *Implementation Science*, *10*(1), 21.

Prochaska, J. O., & Velicer, W. F. (1997). The transtheoretical model of health behavior change. *Am J Health Promot*, *12*(1), 38–48.

Proctor, E. K., Powell, B. J., & McMillen, J. C. (2013). Implementation strategies: Recommendations for specifying and reporting. *Implementation Science*, *8*(1), 139. https://doi.org/10.1186/1748-5908-8-139

Pronovost, P. J., Berenholtz, S. M., Goeschel, C. A., Needham, D. M., Sexton, J. B., Thompson, D. A., Lubomski, L. H., Marsteller, J. A., Makary, M. A., & Hunt, E. (2006). Creating high reliability in health care organizations. *Health Serv Res*, *41*(4 Pt 2), 1599–1617.

Pronovost, P. J., Berenholtz, S. M., & Needham, D. M. (2008). *Translating evidence into practice: A model for large scale knowledge translation*. *337*(oct06_1), a1714-. https://doi.org/10.1136/bmj.a1714

Quinn, R. E., & Rohrbaugh, J. (1981). A Competing Values Approach to Organizational Effectiveness. *Public Productivity Review*, *5*(2), 122. https://doi.org/10.2307/3380029

Rabin, B. A., Brownson, R. C., Haire-Joshu, D., Kreuter, M. W., & Weaver, N. L. (2008). A glossary for dissemination and implementation research in health. *J Public Health Manag Pract*, *14*(2), 117–123.

Raghavan, R., Bright, C. L., & Shadoin, A. L. (2008). Toward a policy ecology of implementation of evidence-based practices in public mental health settings. *Implementation Science*, *3*(1), 26. https://doi.org/10.1186/1748-5908-3-26

Ramsey, A. T., van den Berk-Clark, C., & Patterson Silver Wolf (Adelv unegv Waya), D. A. (2015). Provider-agency fit in substance abuse treatment organizations: Implications for learning climate, morale, and evidence-based practice implementation. *BMC Research Notes*, *8*(1), 194. https://doi.org/10.1186/s13104-015-1110-3

Reed, J. E., Howe, C., Doyle, C., & Bell, D. (2018). Simple rules for evidence translation in complex systems: A qualitative study. *BMC Medicine*, *16*(1), 92. https://doi.org/10.1186/s12916-018-1076-9

Repenning, N. P. (2002). A Simulation-Based Approach to Understanding the Dynamics of Innovation Implementation. *Organization Science*, *13*(2), 109–127. https://doi.org/10.1287/orsc.13.2.109.535

Ritchie, M. J., Parker, L. E., & Kirchner, J. E. (2020). From novice to expert: A qualitative study of implementation facilitation skills. *Implementation Science Communications*, *1*(1), 25. https://doi.org/10.1186/s43058-020-00006-8

Rogers, E. (2003). *Diffusion of innovations: 5th ed.* Free Press.

Rogers, L., De Brún, A., & McAuliffe, E. (2020). Defining and assessing context in healthcare implementation studies: A systematic review. *BMC Health Services Research*, *20*(1), 591. https://doi.org/10.1186/s12913-020-05212-7

Rosenthal, A., Stover, E., & Haar, R. J. (2021). Health and social impacts of California wildfires and the deficiencies in current recovery resources: An exploratory qualitative study of systems-level issues. *PLOS ONE*, *16*(3), e0248617. https://doi.org/10.1371/journal.pone.0248617

Rotenstein, L. S., & Johnson, A. K. (2020). Taking Back Control—Can Quality Improvement Enhance The Physician Experience? *Health Affairs Blog*.

Rycroft-Malone, J., Harvey, G., Kitson, A., McCormack, B., Seers, K., & Titchen, A. (2002). Getting evidence into practice: Ingredients for change. *Nurs Stand*, *16*(37), 38–43.

Rycroft-Malone, J., Kitson, G., Harvey, B., McCormack, K., Seers, A. T., & C. Estabrooks. (2002). Ingredients for change: Revisiting a conceptual framework. (Viewpoint). *Quality and Safety in Health Care*, *11*(2), 174–180.

Safaeinili, N., Brown‐Johnson, C., Shaw, J. G., Mahoney, M., & Winget, M. (2020). CFIR simplified: Pragmatic application of and adaptations to the Consolidated Framework for Implementation Research (CFIR) for evaluation of a patient‐centered care transformation within a learning health system. *Learning Health Systems*, *4*(1). https://doi.org/10.1002/lrh2.10201

Safran, D. G., Miller, W., & Beckman, H. (2006). Organizational dimensions of relationship-centered care. Theory, evidence, and practice. *J Gen Intern Med*, *21 Suppl 1*, S9-15.

Saint, S., Christine P. Kowalski, Jane Banaszak-Holl, Jane Forman, Laura Damschroder, & Sarah L. Krein. (2009). How Active Resisters and Organizational Constipators Affect Health Care–Acquired Infection Prevention Efforts. *The Joint Commission Journal on Quality and Patient Safety*, *35*(5), 239–246.

Santos, W. J., Graham, I. D., Lalonde, M., Demery Varin, M., & Squires, J. E. (2022). The effectiveness of champions in implementing innovations in health care: A systematic review. *Implementation Science Communications*, *3*(1), 80. https://doi.org/10.1186/s43058-022-00315-0

Sarkies, M., Long, J. C., Pomare, C., Wu, W., Clay-Williams, R., Nguyen, H. M., Francis-Auton, E., Westbrook, J., Levesque, J.-F., Watson, D. E., & Braithwaite, J. (2020). Avoiding unnecessary hospitalisation for patients with chronic conditions: A systematic review of implementation determinants for hospital avoidance programmes. *Implementation Science*, *15*(1), 91. https://doi.org/10.1186/s13012-020-01049-0

Schon, D. A. (1963). Champions for radical new inventions. *Harvard Business Review*, *41*, 77–86.

Scott, J. (2000). *Social Network Analysis: A Handbook* (2nd ed.). Sage Publications.

Shortell, S. M., Marsteller, J. A., Lin, M., Pearson, M. L., Wu, S. Y., Mendel, P., Cretin, S., & Rosen, M. (2004). The role of perceived team effectiveness in improving chronic illness care. *Med Care*, *42*(11), 1040–1048.

Shortell, S. M., Zazzali, J. L., Burns, L. R., Alexander, J. A., Gillies, R. R., Budetti, P. P., Waters, T. M., & Zuckerman, H. S. (2001). Implementing evidence-based medicine: The role of market pressures, compensation incentives, and culture in physician organizations. *Medical Care*, *39*(7 Suppl 1), I62-78.

Simpson, D. D. (2002). A conceptual framework for transferring research to practice. *Journal of Substance Abuse Treatment*, *22*(4), 171–182.

Simpson, D. D., & Dansereau, D. F. (2007). Assessing Organizational Functioning as a Step Toward Innovation. *NIDA Science & Practice Perspectives*, *3*(2), 20–28.

Singh, J. V., & Lumsden, C. J. (1990). Theory and Research in Organizational Ecology. *Annual Review of Sociology*, *16*, 161–195. JSTOR.

Smith, A. C., DW Organ, & JP Near. (1983). Organizational Citizenship Behavior: Its Nature and Antecedents. *Journal of Applied Psychology*, *68*(4), 653–663.

Smith, J. D., Li, D. H., & Rafferty, M. R. (2020). The Implementation Research Logic Model: A method for planning, executing, reporting, and synthesizing implementation projects. *Implementation Science*, *15*(1), 84. https://doi.org/10.1186/s13012-020-01041-8

Smith, S. N., Liebrecht, C. M., Bauer, M. S., & Kilbourne, A. M. (2020). Comparative effectiveness of external vs blended facilitation on collaborative care model implementation in slow‐implementer community practices. *Health Services Research*, *55*(6), 954–965. https://doi.org/10.1111/1475-6773.13583

Solberg, L. I., Kuzel, A., Parchman, M. L., Shelley, D. R., Dickinson, W. P., Walunas, T. L., Nguyen, A. M., Fagnan, L. J., Cykert, S., Cohen, D. J., Balasubramanaian, B. A., Fernald, D., Gordon, L., Kho, A., Krist, A., Miller, W., Berry, C., Duffy, D., & Nagykaldi, Z. (2021). A Taxonomy for External Support for Practice Transformation. *The Journal of the American Board of Family Medicine*, *34*(1), 32–39. https://doi.org/10.3122/jabfm.2021.01.200225

Spitzer-Shohat, S., Shadmi, E., Goldfracht, M., Key, C., Hoshen, M., & Balicer, R. D. (2018). Evaluating an organization-wide disparity reduction program: Understanding what works for whom and why. *PLOS ONE*, *13*(3), e0193179. https://doi.org/10.1371/journal.pone.0193179

Squires, J. E., Aloisio, L. D., Grimshaw, J. M., Bashir, K., Dorrance, K., Coughlin, M., Hutchinson, A. M., Francis, J., Michie, S., Sales, A., Brehaut, J., Curran, J., Ivers, N., Lavis, J., Noseworthy, T., Vine, J., Hillmer, M., & Graham, I. D. (2019). Attributes of context relevant to healthcare professionals’ use of research evidence in clinical practice: A multi-study analysis. *Implementation Science*, *14*(1), 52. https://doi.org/10.1186/s13012-019-0900-8

Stanick, C. F., Halko, H., Mettert, K., Dorsey, C., Moullin, J., Weiner, B., Powell, B., & Lewis, C. C. (2021). Measuring characteristics of individuals: An updated systematic review of instruments’ psychometric properties. *Implementation Research and Practice*, *2*, 263348952110004. https://doi.org/10.1177/26334895211000458

Stetler, C. B. (2001). Updating the Stetler Model of research utilization to facilitate evidence-based practice. *Nursing Outlook*, *49*(6), 272–279.

Stirman, S. W., Baumann, A. A., & Miller, C. J. (2019). The FRAME: an expanded framework for reporting adaptations and modifications to evidence-based interventions. *Implementation Science*, *14*(1), 1–10.

Stokols, D. (1996). Translating Social Ecological Theory into Guidelines for Community Health Promotion. *American Journal of Health Promotion*, *10*(4), 282–298. https://doi.org/10.4278/0890-1171-10.4.282

Sue Dopson, & Louise Fitzgerald. (2006). The active role of context. In S. Dopson & L. Fitzgerald (Eds.), *Knowledge to action? Evidence-based health care in context* (p. 223). Oxford University Press.

Tabak, R. G., & Moreland-Russell, S. (2015). Food Service Perspectives on National School Lunch Program Implementation. *Health Behavior and Policy Review*, *2*(5), 362–371. https://doi.org/10.14485/HBPR.2.5.4

Tagliabue, M., Sigurjonsdottir, S. S., & Sandaker, I. (2020). The effects of performance feedback on organizational citizenship behaviour: A systematic review and meta-analysis. *European Journal of Work and Organizational Psychology*, *29*(6), 841–861. https://doi.org/10.1080/1359432X.2020.1796647

Templeton, G. F., Lewis, B. R., & Snyder, C. A. (2002). Development of a measure for the organizational learning construct. *Journal of Management Information Systems*, *19*(2), 175–218.

Teplensky, J. D., Pauly, M. V., Kimberly, J. R., Hillman, A. L., & Schwartz, J. S. (1995). Hospital adoption of medical technology: An empirical test of alternative models. *Health Services Research*, *30*(3), 437–465.

The AIMD Writing/Working Group, Bragge, P., Grimshaw, J. M., Lokker, C., & Colquhoun, H. (2017). AIMD - a validated, simplified framework of interventions to promote and integrate evidence into health practices, systems, and policies. *BMC Medical Research Methodology*, *17*(1), 38. https://doi.org/10.1186/s12874-017-0314-8

Thompson, J., Scott, W., & Zald, M. (2003). *Organizations in Action: Social Science Bases of Administrative Theory*. Transaction Publishers.

Tiderington, E., Ikeda, J., & Lovell, A. (2020). Stakeholder Perspectives on Implementation Challenges and Strategies for Moving On Initiatives in Permanent Supportive Housing. *The Journal of Behavioral Health Services & Research*, *47*(3), 346–364. https://doi.org/10.1007/s11414-019-09680-6

Tinc, P. J., Gadomski, A., Sorensen, J. A., Weinehall, L., Jenkins, P., & Lindvall, K. (2018). Applying the Consolidated Framework for implementation research to agricultural safety and health: Barriers, facilitators, and evaluation opportunities. *Safety Science*, *107*, 99–108. https://doi.org/10.1016/j.ssci.2018.04.008

Trumbo, S. P., Iams, W. T., Limper, H. M., Goggins, K., Gibson, J., Oliver, L., Leverenz, D. L., Samuels, L. R., Brady, D. W., & Kripalani, S. (2019). Deimplementation of Routine Chest X-rays in Adult Intensive Care Units. *Journal of Hospital Medicine*, *14*(2), 83–89. https://doi.org/10.12788/jhm.3129

Tucker, A. L., Nembhard, I. M., & Edmondson, A. C. (2007). Implementing New Practices: An Empirical Study of Organizational Learning in Hospital Intensive Care Units. *Management Science*, *53*(6), 894–907. https://doi.org/10.1287/mnsc.1060.0692

US DHHS-National Cancer Institute. (2005). *Theory at a Glance: A guide for health promotion practice 2nd Edition*. https://cancercontrol.cancer.gov/sites/default/files/2020-06/theory.pdf

Valente, T. W., Chou, C. P., & Pentz, M. A. (2007). Community Coalitions as a System: Effects of Network Change on Adoption of Evidence-Based Substance Abuse Prevention. *American Journal of Public Health*, *97*(5), 880–886. https://doi.org/10.2105/AJPH.2005.063644

Van de Ven, A. H. (1986). Central Problems in the Management of Innovation. *Management Science*, *32*(5), 590–607. https://doi.org/10.1287/mnsc.32.5.590

Van de Ven, A. H., Polley, D. E., Garud, R., & Vandataraman, S. (1999). *The Innovation Journey*. Oxford University Press.

van Eijnatten, F. M., & Galen, M. (2002). Chaos, dialogue and the dolphin’s strategy. *Journal of Organizational Change Management*, *15*(4), 391–401.

VanDeusen Lukas, C., Holmes, S. K., Cohen, A. B., Restuccia, J., Cramer, I. E., Shwartz, M., & Charns, M. P. (2007). Transformational change in health care systems: An organizational model. *Health Care Management Review*, *32*(4), 309–320. https://doi.org/10.1097/01.HMR.0000296785.29718.5d

Varsi, C., Ekstedt, M., Gammon, D., & Ruland, C. M. (2015). Using the Consolidated Framework for Implementation Research to Identify Barriers and Facilitators for the Implementation of an Internet-Based Patient-Provider Communication Service in Five Settings: A Qualitative Study. *Journal of Medical Internet Research*, *17*(11), e262. https://doi.org/10.2196/jmir.5091

von Thiele Schwarz, U., Aarons, G. A., & Hasson, H. (2019). The Value Equation: Three complementary propositions for reconciling fidelity and adaptation in evidence-based practice implementation. *BMC Health Services Research*, *19*(1), 868. https://doi.org/10.1186/s12913-019-4668-y

Wagner, D. J., Durbin, J., Barnsley, J., & Ivers, N. M. (2017). Beyond quality improvement: Exploring why primary care teams engage in a voluntary audit and feedback program. *BMC Health Services Research*, *17*(1), 803. https://doi.org/10.1186/s12913-017-2765-3

Wallin, L., Estabrooks, C. A., Midodzi, W. K., & Cummings, G. G. (2006). Development and Validation of a Derived Measure of Research Utilization by Nurses: *Nursing Research*, *55*(3), 149–160. https://doi.org/10.1097/00006199-200605000-00001

Walston, S. L., Kimberly, J. R., & Burns, L. R. (2001). Institutional and economic influences on the adoption and extensiveness of managerial innovation in hospitals: The case of reengineering. *Med Care Res Rev*, *58*(2), 194–228; discussion 229-33.

Waltz, T. J., Powell, B. J., Fernández, M. E., Abadie, B., & Damschroder, L. J. (2019). Choosing implementation strategies to address contextual barriers: Diversity in recommendations and future directions. *Implementation Science*, *14*(1), 42. https://doi.org/10.1186/s13012-019-0892-4

Wandersman, A., Imm, P., Chinman, M., & Kaftarian, S. (2000). Getting to outcomes: A results-based approach to accountability. *Evaluation and Program Planning*, *23*(3), 389–395. https://doi.org/10.1016/S0149-7189(00)00028-8

Weiner, B. J., Mettert, K. D., Dorsey, C. N., Nolen, E. A., Stanick, C., Powell, B. J., & Lewis, C. C. (2020). Measuring readiness for implementation: A systematic review of measures’ psychometric and pragmatic properties. *Implementation Research and Practice*, *1*, 263348952093389. https://doi.org/10.1177/2633489520933896

Wells, R., Breckenridge, E. D., & Linder, S. H. (2020). Wellness project implementation within Houston’s Faith and Diabetes initiative: A mixed methods study. *BMC Public Health*, *20*(1), 1050. https://doi.org/10.1186/s12889-020-09167-6

Wensing, M., Sales, A., Armstrong, R., & Wilson, P. (2020). Implementation science in times of Covid-19. *Implementation Science*, *15*(1), 42, s13012-020-01006–x. https://doi.org/10.1186/s13012-020-01006-x

Wierenga, D., Engbers, L. H., Van Empelen, P., Duijts, S., Hildebrandt, V. H., & Van Mechelen, W. (2013). What is actually measured in process evaluations for worksite health promotion programs: A systematic review. *BMC Public Health*, *13*(1), 1190. https://doi.org/10.1186/1471-2458-13-1190

Williams, N. J., Ehrhart, M. G., Aarons, G. A., Marcus, S. C., & Beidas, R. S. (2018). Linking molar organizational climate and strategic implementation climate to clinicians’ use of evidence-based psychotherapy techniques: Cross-sectional and lagged analyses from a 2-year observational study. *Implementation Science*, *13*(1), 85. https://doi.org/10.1186/s13012-018-0781-2

Yuan, S., Wang, F., Li, X., Jia, M., & Tian, M. (2019). Facilitators and barriers to implement the family doctor contracting services in China: Findings from a qualitative study. *BMJ Open*, *9*(10), e032444. https://doi.org/10.1136/bmjopen-2019-032444
